# Optical Phenomena in Molecule-Based Magnetic Materials

**DOI:** 10.1021/acs.chemrev.3c00840

**Published:** 2024-04-30

**Authors:** Jakub J. Zakrzewski, Michal Liberka, Junhao Wang, Szymon Chorazy, Shin-ichi Ohkoshi

**Affiliations:** † Faculty of Chemistry, Jagiellonian University, Gronostajowa 2, 30-387 Krakow, Poland; ‡ Doctoral School of Exact and Natural Sciences, Jagiellonian University, Lojasiewicza 11, 30-348 Krakow, Poland; § Department of Materials Science, Faculty of Pure and Applied Science, University of Tsukuba, 1-1-1 Tonnodai, Tsukuba, Ibaraki 305-8573, Japan; # Department of Chemistry, School of Science, The University of Tokyo, 7-3-1 Hongo, Bunkyo-ku, Tokyo 113-0033, Japan

## Abstract

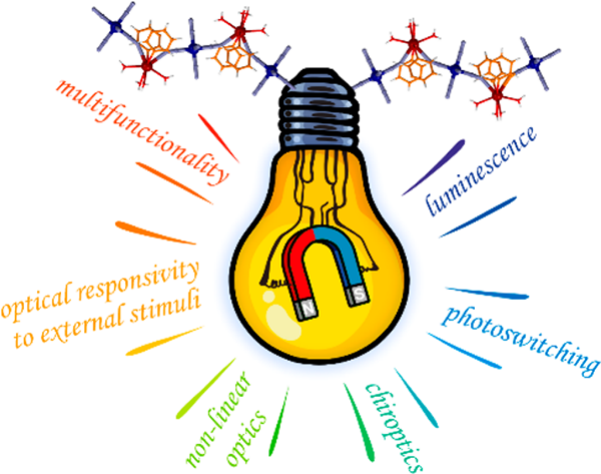

Since the last century, we have witnessed the development of molecular magnetism which deals with magnetic materials based on molecular species, i.e., organic radicals and metal complexes. Among them, the broadest attention was devoted to molecule-based ferro-/ferrimagnets, spin transition materials, including those exploring electron transfer, molecular nanomagnets, such as single-molecule magnets (SMMs), molecular qubits, and stimuli-responsive magnetic materials. Their physical properties open the application horizons in sensors, data storage, spintronics, and quantum computation. It was found that various optical phenomena, such as thermochromism, photoswitching of magnetic and optical characteristics, luminescence, nonlinear optical and chiroptical effects, as well as optical responsivity to external stimuli, can be implemented into molecule-based magnetic materials. Moreover, the fruitful interactions of these optical effects with magnetism in molecule-based materials can provide new physical cross-effects and multifunctionality, enriching the applications in optical, electronic, and magnetic devices. This Review aims to show the scope of optical phenomena generated in molecule-based magnetic materials, including the recent advances in such areas as high-temperature photomagnetism, optical thermometry utilizing SMMs, optical addressability of molecular qubits, magneto-chiral dichroism, and opto-magneto-electric multifunctionality. These findings are discussed in the context of the types of optical phenomena accessible for various classes of molecule-based magnetic materials.

## 1 Introduction

Molecule-based magnetic materials are functional solids exhibiting an extensive range of magnetic properties,^1^ for instance, room-temperature permanent magnetization,^2^ which is broadly utilized in technology and industry employing classical magnets based on metals, metal oxides, or intermetallics.^3−5^ However, contrary to them, molecule-based magnetic materials are constructed from well-defined molecular components, such as paramagnetic organic radicals or metal ions coordinating organic and inorganic ligands.^6,7^ Thus, they take advantage of a molecular building blocks approach enabling the selection of proper functional molecular precursors generating desired physical properties that are not easily accessible in the typical high-temperature synthesis of traditional metal- or metal-oxide-based magnets.^8,9^ Moreover, the construction of magnetic materials from molecules opens the routes for unique magnetic phenomena,^1,10−12^ not observed in their conventional analogs, as well as for extreme miniaturization of the related electronic and magnetic nanodevices as their magnetic effects can originate from the set of several molecules or even a single molecule.^10,13,14^ This methodology was also found successful in other branches of materials science where molecular ferroelectrics,^15,16^ luminophores,^17,18^ nonlinear optical materials,^19,20^ or ionic/electrical conductors,^21,22^ based on organic molecules and metal complexes, have appeared to be promising alternatives for the more conventional inorganic solids primarily utilized in the related optical, electronic, and magnetic devices.^23−25^

The field of molecular magnetism originates from the investigation of magnetic properties of paramagnetic metal complexes and organic radicals, as well as magnetic coupling between these spin centers.^26−28^ The nature and strength of the magnetic interactions between the molecular spins were found designable which leads to the synthesis of molecule-based materials revealing long-range magnetic ordering of tunable critical temperatures.^6,29−35^ Moreover, the proper selection of magnetic metal ions and supporting ligands leads to the molecule-based materials exhibiting the magnetically ordered state at room temperature, as exemplified by the V^II/III^–[Cr^III^(CN)_6_]^3–^ Prussian Blue analogs (PBAs), the V^II^(TCNE)_*x*_ coordination polymer (CP), and the Cr^II^–pyrazine metal–organic framework (MOF).^2,36−38^ It was simultaneously found that some transition metal ions, mainly of the d^4^–d^7^ valence configuration, can exhibit the spin crossover (SCO) effect between the high-spin (HS) and low-spin (LS) states when placed in the coordination sphere of the intermediate ligand field strength.^39−41^ Such a HS–LS transition can be induced using a series of external stimuli, including temperature, light, pressure, or electric field, and lead to pronounced changes in magnetic, optical, mechanical, and electrical properties.^42−44^ All these features opened the broad branch of molecular magnetism dealing with the design of spin transition materials, particularly employing Fe^II/III^, Mn^III^, and Co^II^ complexes, for sensing and data storage applications.^40,42,45−48^ Furthermore, the stimuli-responsive molecule-based materials exhibiting intermetallic electron transfer aroused considerable interest as such a process was found to be sometimes accompanied by the spin transition phenomenon.^49,50^ The resulting electron transfer-coupled spin transition (ETCST), named also charge transfer-induced spin transition (CTIST), offers the efficient switching of physical properties by external stimuli, analogous to the SCO systems, ensuring often high cooperativity of the related phase transition which is crucial in magnetic memory applications.^51−53^ Since the 1990s, the gradually increasing interest in the molecular magnetism field is shifted toward the design and investigation of strongly magnetically anisotropic metal complexes exhibiting the effect of slow relaxation of magnetization.^10,54−56^ This leads to the magnetic hysteresis loop of the molecular origin appearing below the so-called blocking temperature (*T*_B_), which opens the pathway toward data storage devices realized at the remarkable scale of single molecules.^56−58^ This phenomenon was primarily found in the polynuclear coordination clusters based on transition metal ions, such as the families of {Mn^III/IV^_12_} and {Fe^III^_4_} molecules, showing the high spin ground states appearing due to the magnetic coupling between metal centers of the non-negligible magnetic anisotropy.^54,59−61^ The resulting single-molecule magnets (SMMs) were further constructed using the polynuclear heterometallic d–d or d–f discrete systems,^62−64^ as well as the clusters based purely on the f-block metal ions.^65^ The analogous effect of slow magnetic relaxation of a molecular origin was also detected for isolated one-dimensional coordination polymers, named single-chain magnets (SCMs), bearing magnetically anisotropic d- or f-block metal centers efficiently coupled through bridging ligands,^66−70^ often with the assistance of bridging organic radicals.^66,67,69^ In these regards, the last two decades proved that high-performance SMMs can be even built of mononuclear complexes of transition,^71−73^ lanthanide,^55,56,74−80^ or actinide metal ions.^81,82^ Among them, a particularly huge magnetic anisotropy, resulting from the combination of large spin-orbit coupling (SOC) and a smaller, yet critical, crystal-field (CF) effect, was generated for the complexes of trivalent lanthanide ions, such as Dy^3+^, Tb^3+^, and Er^3+^.^74−79^ This produced the best-performance SMMs exhibiting *T*_B_ values exceeding the liquid nitrogen temperature that brings the whole class of molecular nanomagnets closer to the applications in memory devices;^56^ however, the subsequent task of processing them into devices is truly challenging.^14,57,83^ The SMMs, along with some relative magnetic molecules, are also investigated as active units in spintronic devices.^84−86^ At the same time, the quest for even better SMMs is continued, which includes, among others, playing with the magnetic coupling between two closely attached lanthanide centers^87,88^ as well as the experimental and theoretical studies of the crucial factors governing the magnetic relaxation processes, especially from the viewpoint of the interactions between the spin and the crystal lattice.^89,90^ Among other concepts emerging in molecular magnetism, it is worth noting the usage of magnetic molecules, including mononuclear complexes or spin centers in coordination clusters or even MOFs, with two- or multi-level electronic structures as molecular objects for encoding qubits or qudits.^12,91−94^ This opens possible applications of magnetic molecules in quantum computing and simulations.^95,96^

Molecule-based magnetic systems were found to be sensitive to external chemical and physical stimuli.^1,97−100^ In the context of processing into electronic or magnetic devices, it is often considered a disadvantage when compared with usually robust conventional magnets based on metals or metal oxides.^3,4^ However, the proper control over the interaction between the molecular material and the external stimulus leads to the fruitful tuning and switching of magnetic properties which open the pathways for sensing and data storage applications.^101,102^ In this regard, various external stimuli, ranging from physical ones, such as temperature, electric field, pressure, mechanical force, and electromagnetic radiation, to chemical ones, such as humidity, solvent vapors, gases, acids, or bases, and other guest molecules, were employed in switching of the magnetic phenomena in molecule-based materials.^103−107^ This was achieved by playing not only with spin transition and electron transfer processes, which are intrinsically governed by external stimuli but also working with the materials showing magnetic ordering,^108^ as well as molecular nanomagnets, including SCMs and SMMs.^109,110^ In the most advanced cases, the combinations of at least two magnetic phenomena are explored, e.g., the photoinduced spin transition or electron transfer can be linked with the magnetic coupling leading to the spin ordering which gives the remarkable molecule-based photomagnets exhibiting photoswitchable magnetic memory effect.^52,111^ The quest for molecule-based magnetic materials switchable by external stimuli contributes to the more general research on stimuli-responsive molecular materials, which was recognized also in the context of switching such physical phenomena as electronic or ionic conductivity, gas sorption, or ferroelectricity.^112−117^ Besides the realization of the molecular-level switching of physical properties, in the case of magnetic molecular materials, the external stimuli were employed in the post-synthetic optimization of the desired physical effects, e.g., the increase of the critical temperature of magnetic ordering either by the controlled desolvation in the dynamic systems named molecular magnetic sponges or by the hydrostatic pressure.^108,118−120^

Molecule-based magnetic materials provide also an efficient molecular platform for the implementation of multifunctionality defined as the presence of a few different physical functionalities in a single-phase system.^121,122^ Multifunctionality is desired in the pursuit of highly miniaturized advanced devices realizing multiple functions simultaneously and can be achieved by the construction of composite materials built of well-distinguished functional components, e.g., magnetic metal-oxide nanoparticles covered by luminescent quantum dots.^123−125^ However, it was proved that multifunctionality can be generated at the molecular level using the predesigned molecular building blocks bearing defined physical properties that are bound into the single phase which results in their combination of, e.g., chirality with ferroelectricity, super-ionic conductivity with porosity, electronic conductivity with catalytic activity, etc.^126−130^ In these regards, various magnetic phenomena were combined with diverse physical functionalities, such as super-ionic and electronic conductivity,^131,132^ nonlinear optical,^133^ luminescent,^134^ and chiroptical effects,^135^ porosity,^136^ pyro-, piezo-, and ferroelectricity,^137,138^ electrochemical activity,^139^ and also others.^121,122,140^ In some material systems, these properties simply co-exist but they can interact with each other giving new physical cross-effects, such as magneto-chiral dichroism (MChD) being the result of chirality and magnetism in the designed material.^121,141,142^ The products of multifunctionality are particularly rich when the generated physical effects are combined not only with the magnetic effects but also with the sensitivity to external stimuli leading to multifunctional molecular magnetic switches.^143−145^

All the above-introduced aspects of molecule-based magnetic materials indicate that this emerging group of functional materials reveals great potential, especially, in the context of rich multifunctionality and multi-stimuli-responsive switching of physical properties. Both these research pathways are realized when linking molecular magnetism with optical phenomena. The research on diverse optical effects in functional organic, inorganic, or hybrid solids is the vast branch of materials science, with the recent broad scientific interest devoted to unique optical phenomena in molecular materials which, similarly to molecule-based magnetic materials, are based on organic molecules and/or metal complexes.^146−148^ Among the groups of such optical phenomena, one can count light absorption, especially in the visible range of the electromagnetic spectrum related to electronic transitions, which are efficiently switched by external stimuli.^149,150^ The resulting chromic materials are controlled by multiple stimuli including chemical ones (chemochromism, or its variations, e.g., solvatochromism, vapochromism, halochromism, and others)^151−153^ and the series of physical ones such as temperature (thermochromism),^154^ light irradiation (photochromism),^155^ electric field (electrochromism),^156^ pressure (piezochromism),^157^ mechanical action (mechanochromism),^158^ etc., as well as their combinations, e.g., photoelectrochromism or chemophotochromism.^159,160^ They found broad applications in sensors, displays, memory devices, optical transistors, biological probes, security techniques, etc.^155,161−163^ It was presented that light irradiation can not only lead to changes in light absorption but also may induce the switching of many other physical properties, including such critical ones as ionic conductivity,^113^ ferroelectricity,^116^ permanent porosity,^164^ or magnetism.^111^ The resulting photoswitchable materials are considered attractive candidates for multiple applications, e.g., in memory devices, intelligent gas or energy storage, and smart electronic systems.^165−168^ They could be constructed using typical inorganic solids, such as metal oxides;^169^ however, the photoinduced physical phenomena were found to be particularly efficient for molecular materials offering many photosensitive organic molecules and, further metal complexes.^170,171^

Among other optical materials, extensive research works are focused on the design and investigation of the solids exhibiting luminescence that is the light emission occurring due to the absorption of photons (photoluminescence),^172,173^ electric current (electroluminescence),^174^ chemical reaction (chemiluminescence),^175^ ionizing radiation (radioluminescence),^176^ or mechanical action (mechanoluminescence).^177^ They found vital applications ranging from light-emitting diodes (LEDs), optical communication, and photovoltaics, to sensors of physical and chemical stimuli, anticounterfeiting as well as bioimaging tools.^175,178−180^ Luminescence can be realized by playing with the emissive electronic transitions generated in inorganic solids, such as oxides or fluorides,^181,182^ organic molecules and frameworks,^183,184^ as well as complexes of lanthanides or transition metals, including both heavy elements as well as earth abundant 3d metals,^18,185−187^ coordination polymers, including metal–organic frameworks (MOFs),^188,189^ and molecular organic-inorganic hybrids.^190^ The vast part of their applications is related to the construction of sensors which is due to the sensitivity of these materials to external stimuli, especially for molecule-based systems.^191^ In this regard, the stimuli-responsive luminescent materials are the emissive analogs of chromic materials, often named fluorochromic systems.^192^ Thus, they take advantage of the switching of light emission by external stimuli, e.g., solvent molecules (solvatofluorochromism, often called solvatochromic luminescence),^193^ temperature (thermofluorochromism),^194^ electric field (electrofluorochromism),^195^ pressure (piezofluoro-chromism),^196^ or mechanical action (mechanofluorochromism).^197^ Photochromic luminescence was also realized; in this case, as the light irradiation can be used both as a trigger to switch the state of a material and as the generator of the output signal; thus, to employ such luminescent photochromism in optical devices, the usage of two different wavelengths playing two above-mentioned roles is the most convenient, yet challenging, solution.^198,199^

The broad research area within optical materials is also occupied by the materials able to exhibit various nonlinear optical (NLO) properties such as two-photon absorption, second and higher-order harmonic generation (SHG and others), or the optical Kerr effect.^200−202^ These physical phenomena usually occur for the solids crystallizing in the non-centrosymmetric space groups and are considered for multiple applications in nanophotonic devices, including laser systems, sensors, and the characterization of other functional materials.^203−205^ Recent years brought a large increase in the interest in novel NLO materials, especially those based on organic molecules, metal complexes, and MOFs or molecular perovskites.^205−208^ The application horizon of the NLO materials is further broadened when the influence of a magnetic field is added resulting in the nonlinear magneto-optical effects, such as magnetization-induced SHG (MSHG).^209^ This arises from the more general interest in the investigation of physical interaction between light and magnetic matter providing such physical phenomena as the magneto-optic Kerr effect (MOKE) used in magnetic media and microscopy.^210^ On the other hand, the construction of chiral materials becomes the efficient route for the next-generation optical effects, so-called chiroptical ones.^211,212^ They range from optical rotation, optical rotatory dispersion (ORD), and circular dichroism (CD), which are the effects particularly useful in the studies of biological molecules and chemical sensors,^213,214^ up to circularly polarized luminescence (CPL) which enables the control over the polarization of the emission light for applications in CP-OLEDs (circularly polarized organic light-emitting diodes),^215^ optical information processing,^216^ and advanced optical sensors.^217^ The molecule-based materials, e.g., based on lanthanide complexes or expanded organic systems, are now broadly investigated for the strong and tunable CPL signal.^218,219^ These optical functionalities do not complete the research on optical materials as the branch of photonics focusing on various aspects of generation, manipulation, and detection of light is intensively developed using diverse solid state systems, including MOFs and other molecule-based systems, and their applications, in such areas as medicine, photovoltaics, lasers, light detection, or information processing, are ubiquitous.^220−223^

The separate sub-field of the research on optical materials is related to the exploration of multifunctional systems combining a few linear and nonlinear optical phenomena.^224,225^ This trend can be also exemplified by families of stimuli-responsive materials revealing both the light absorption and emission switchable by external stimuli, including those showing several types of chromic responses or the luminescence of diverse sources.^226,227^ The emerging pathway consists also of the quest for acentric and chiral optical materials linking NLO or chiroptical properties with advanced luminescent effects as well as the responsivity to external stimuli providing such coupling phenomena as the chemically switchable SHG or the photoswitchable CPL.^228−230^

From the above introduction, it appears that both molecular magnetism as well as the field related to the design of optical materials, including molecule-based ones, are emerging areas in materials science. Almost since the rise of the interest in molecule-based magnetic materials, their optical properties were investigated and the correlations between the optical and magnetic features were explored, e.g., the color change upon the thermal spin transition.^231,232^ Further, the variety of physical phenomena related to light-matter interactions was implemented into molecule-based magnetic materials, which include the switching of magnetism by light,^231−233^ the construction of magnets from luminescent molecular building blocks,^234^ or the syntheses of chiral and non-centrosymmetric molecule-based magnetic materials for chiroptical as well as nonlinear optical and magneto-optical effects.^209,235,236^ These efforts were greatly intensified in the last fifteen years opening a series of new magneto-optical correlations,^237^ advanced stimuli-responsive systems,^238^ and unique examples of rich multifunctionality.^143,239^ In this review, we will summarize the achievements in the generation of optical phenomena in molecule-based magnetic materials focusing on the types of accessible optical effects and the recent advances presented in the literature, however, the background of the pioneering works on light-matter interactions in molecule-based magnetic materials is also shown. Besides this introduction, this scope of the review is realized in six thematic sections focusing on various types of optical phenomena in molecule-based magnetic materials, including thermochromism, photoswitching effects, luminescence, chirality- and non-centrosymmetricity-related optical phenomena, switching of optical effects by non-light external stimuli, and the extended optical multifunctionality in the systems combining a few above-listed physical properties. In these sections, we discuss the optical phenomena from the viewpoints of their implementation into different classes of molecule-based magnetic materials, including magnetically ordered systems, spin transition and electron transfer materials, and molecular nanomagnets. The functional potential in linking the optical phenomena with the magnetic ones in each of these classes is emphasized, which is presented together with the deeper discussions on the selected hot topics in the research on related opto-magnetic molecule-based materials. In the Summary, Conclusions, and Perspectives section, we comment on the most promising research pathways in the research on novel optical phenomena in molecule-based magnetic materials, underlying the expected future breakthroughs and the more general perspectives for this emerging sub-field of chemistry and materials science.

## 2 Thermal Switching of Light Absorption in Molecule-Based Magnetic Materials

Thermochromic materials change light absorption properties upon temperature change, which usually concerns the systems for which the absorbed light belongs to the visible region of an electromagnetic spectrum.^240−242^ Then the heating-cooling cycles lead to the material’s color modification; however, the recent scientific and technological interest was also broadened towards, e.g., the NIR absorption thermochromic effect.^243,244^ In general, thermochromism is observed in a variety of (a) inorganic materials, such as metal oxides, iodides, or chlorides, as well as metal alloys, (b) transition metal complexes, (c) organic–inorganic hybrid salts, (d) organic materials, including those containing specific functional groups, e.g., spiropyrans, fluoranthene, triaryl-methane, etc., and (e) the related liquid crystalline and conjugated polymers.^154,240−249^ Depending on the type of the functional material system, the optical thermoresponse is realized through multiple mechanisms including (i) intramolecular electron or proton transfer in organic or hybrid materials, (ii) ring opening or interconversion of stereoisomeric forms of organic molecules, (iii) crystal transformation in organic materials as well as inorganic solids, (iv) band gap energy change in inorganic semiconductors, (v) changes in crystal field effect, coordination geometry, or coordination number in compounds containing transition metal ions, especially those embedded in metal complexes.^240−249^ This results in the great tunability of the thermochromic property which enables a broad set of its applications covering smart material coatings for window glass surfaces,^244,245,250^ thermoresponsive dyes, pigments, paints, inks, cement, textiles, etc.,^251−253^ as well as thermosensors,^254^ color-indicators,^255^ and anti-counterfeiting systems.^256^ In the context of molecule-based magnetic materials, thermochromism was found to be almost inextricably connected with the appearance of thermally induced spin transition and electron transfer phenomena.^44,257^ This leads to the efficient coupling between the thermal switching of magnetic properties and the concomitant switching of optical absorption. The two following sections (2.1 and 2.2) will summarize these thermally induced opto-magnetic switching effects, together with the resulting application potential of thermochromic molecular magnetic materials.

### 2.1 Spin Crossover Materials Exhibiting Switchable Light Absorption

The spin crossover (SCO) phenomenon is the transition between the high-spin (HS) and low-spin (LS) states that exist for the transition metal ions of the d^4^–d^7^ valence configurations depending on the ligand field strength. In the intermediate ligand field, which is characteristic of the selected metal center, the SCO effect can occur using an external stimulus, e.g., temperature, light, or pressure. The distribution of electrons on the d orbitals differs for the HS and LS states which determine the variable physical properties, including magnetic (e.g., total spin and the resulting magnetic moment) and optical (e.g., the set of allowed and forbidden d–d electronic transitions governing the UV–vis light absorption) properties.^39−48,258^ As a result, from the beginning of the research on the SCO phenomenon in transition metal complexes or clusters, and related materials, the occurrence of externally induced, i.e., classically by cooling/heating the sample, spin transition effect was observed not only in the magnetic measurements but also in the color change and the related modification of optical absorption spectra.^39−41,259−261^ Therefore, the SCO systems are considered good candidates for thermochromic materials with their respective broad application potential.^262,263^

The pioneering works on the SCO effect and its influence on both magnetic moment and optical absorption were realized using Fe(II) centers which reveal the thermal spin transition when embedded in the relatively convenient coordination environment ensured by imine-type N-donor ligands, such as pyridine or triazole derivatives, often also accompanied by N-donor anionic ligands, e.g., NCX^–^ (X = S, Se) or NC^–^, the latter usually provided by attached cyanido-containing metalloligands.^259−261,264−266^ Such coordination sphere can be achieved using a rather classical coordination chemistry approach; moreover, the SCO effect on octahedral Fe(II) sites of the d^6^ valence configuration leads to the dramatic change in physical properties as the HS phase of the t_2g_^4^e_g_^2^ configuration is paramagnetic, usually very weakly colored (all of the d–d electronic transitions responsible for the visible light absorption are strongly forbidden), and exhibits relatively long Fe–ligand distances due to the two electrons occupying the antibonding e_g_ orbitals, while the LS phase of the t_2g_^6^ configuration is diamagnetic, often deeply colored (due to the series of only partially forbidden d–d electronic transitions), and exhibits much shorter Fe–ligand distances. As a result, the SCO-active Fe(II) complexes are not only broadly achievable but also reveal the most pronounced switching of numerous physical properties, including magnetic and optical, as well as mechanical or dielectric ones; thus, the related switching effects are still under intensive investigation.^258,267−269^ In the context of designing materials based on the SCO systems, an enormous development has been observed in the recent decade when many different types of molecular systems from this family were successfully transferred into functional nanomaterials.^270,271^ Although in most cases upon the formation of nanomaterials, bulk properties of the system are modified, the most crucial point lies in the preservation of its integrity and related SCO-activity. Among successful strategies, one may list the synthesis of SCO nanoparticles, core-shell heterostructures, thin film nanocrystalline phases, or even molecule-based monolayers.^271−275^ Moreover, when deposited on the surface, the SCO-active systems can modify the properties of the substrate upon the occurrence of the spin transition stimulated by the change of temperature, irradiation, and other factors, while the output is registered in the form of electrical or optical response.^276−281^ When combining two magnetic phases into heterostructures, the impact of SCO occurring in the first component upon the properties of the second was noticed.^282^ Generalizing to some extent, from the point of the application horizon, the materials showing the near-room-temperature spin crossover effect are the most suitable for sensing, including temperature sensing in solution, sensing of solvent vapors and gases, or mechanical strain sensing in the solid phase.^283−286^ Then the related spin transition may or even should occur within a low-cooperativity process, to observe rather gradual changes within the optical absorption or the emission in the case of luminescent SCO materials. On the other hand, for the design of electronic devices, mostly relying on the modified conductivity between the HS and LS states, the presence of thermal SCO hysteresis, in the best case wide and repeatable within several thermal cycles, is required, which can be ensured by strong elastic interactions within the framework.^287^ From this viewpoint, the deposition of single molecules on surfaces becomes a difficult issue as the interaction of a molecule with the substrate tends to strongly modify or even quench the SCO activity. However, some examples of even photoswitching of single SCO molecules on surfaces were reported up to date, which is considered an important step toward future spintronic devices involving molecule-based magnetic materials.^102,288^

The development of SCO devices, as well as the related systems obtained primarily in bulk or the solution, requires proper characterization techniques, which drove the advances in SQUID magnetometry, ^57^Fe Mössbauer spectroscopy, microscopy methods, ultrafast absorption techniques, and others.^289−293^ Moreover, to rationalize the observed SCO characteristics and to be able to rationally design next-generation systems, a thorough structure–property analysis of a rich library of SCO compounds remains a hot topic in this field.^294−296^ However, among the basic and most successful methods used to characterize spin-transition materials, the thermal variation of the electronic absorption in the UV–vis region is still a commonly employed technique, which is due to the simplicity of the optical output in terms of possible applications.

**Figure 1:**
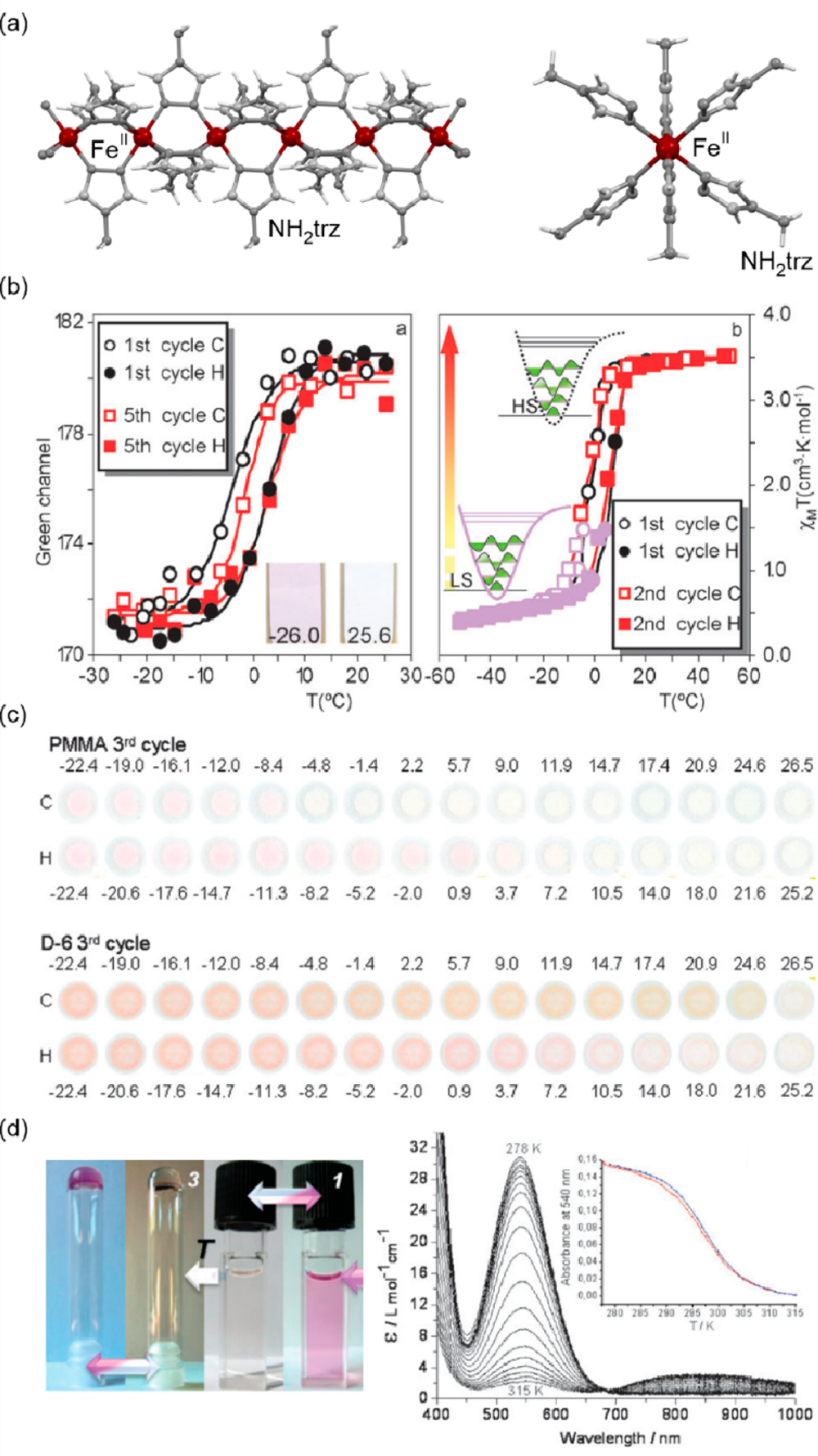
Representative structural fragments of [Fe^II^(NH_2_trz)_3_](NO_3_)_2_·2H_2_O (NH_2_trz = 4-amino-4H-1,2,4-triazole) coordination polymers (a),^298^ thermal variation of the color intensity of the analogous chains with BF_4_^–^ ions, depicted by green channel histogram modification and shown together with the thermal hysteresis loops of the *χ*_M_*T* product (b), the series of photos for the thermochromic behavior of the BF_4_-containing compound dispersed in the PMMA polymer and polyurethane hydrogel D-6 (C = cooling, H = heating) (c), and the photos of high- and low-temperature phases of the analogous chains with tosylate counter-ions obtained as the gel and the colloidal suspension of nanoparticles, shown together the related variable temperature absorption spectra and the thermal dependence of the absorbance at 540 nm (d). Parts (b) and (c) were reproduced from ref (306) with permission from the Royal Society of Chemistry. Part (d) was reproduced from ref (307) with permission from the Royal Society of Chemistry.

As for demonstration of the potential of Fe(II) complexes in the synchronous thermal switching of both magnetism and light absorption, it is worth using one of the richest family of SCO compounds which are based on Fe(II) centers and N-donor triazole-type organic ligands. It was found that the simplest 1,2,4-triazole (trz), as well as its various derivatives (R-trz, with the R-group substituting the H-atom on the first position), produce one-dimensional (1-D) coordination polymers with Fe(II) centers that can be roughly described by the general formula of [Fe^II^(trz-ligand)_3_](X)_2_ (X = various inorganic or organic counter-ions) (Figure 1a).^257,297−302^ The resulting coordination environment of the Fe(II) sites provides the intermediate ligand fields giving the SCO effect occurring in the temperature range close to room temperature which makes them perfect candidates for applications in various devices. Their SCO performance, including the spin transition cooperativity leading to the thermal hysteresis loop as well as the height of transition temperature, can be modulated by modifying the R-group and counter-ions. The great cooperativity achievable for this class of SCO compounds was the basis of their application in memory devices operating around room temperature.^257,303,304^ The related thermochromism was also broadly recognized as usually pink-to-violet LS phase undergoes discoloration upon transition to the HS phase which happens together with the transition from the diamagnetic ground state to the paramagnetic one (Figure 1b).^263,305,306^ Moreover, it was reported that Fe(II)–triazole coordination polymers can be processed into nanomaterials, including thin films and nanoparticles, the latter dispersed in various media with the preservation of the SCO property.^305−309^ Therefore, they were shown to be great precursors for functional thermochromic nanomaterials showing the temperature sensing ability realized by tracing the material’s color or features of the related absorption spectrum, e.g., absorbance at the selected wavelength (Figure 1cd).^306,307^ The SCO-active Fe(II)–triazole coordination polymers were further employed for diverse composite systems and their thermochromic functionality was demonstrated.^308,309^ The range of related functional materials systems was, even more, broadened when the more sophisticated triazole derivatives were used.^310−314^ For instance, the triazole derivatives with long aliphatic chains gave rise to thermochromic liquid crystals based on Fe(II)-based coordination chains,^312^ the polytriazole ligands produced SCO-active Fe(II) coordination layers that could be exfoliated toward thermochromic films with controlled nanometer-scale thickness,^313^ while the tridentate triazole-type ligands provided mononuclear Fe(II) complexes processable to thermochromic thin films by a vacuum deposition method.^314^

**Figure 2:**
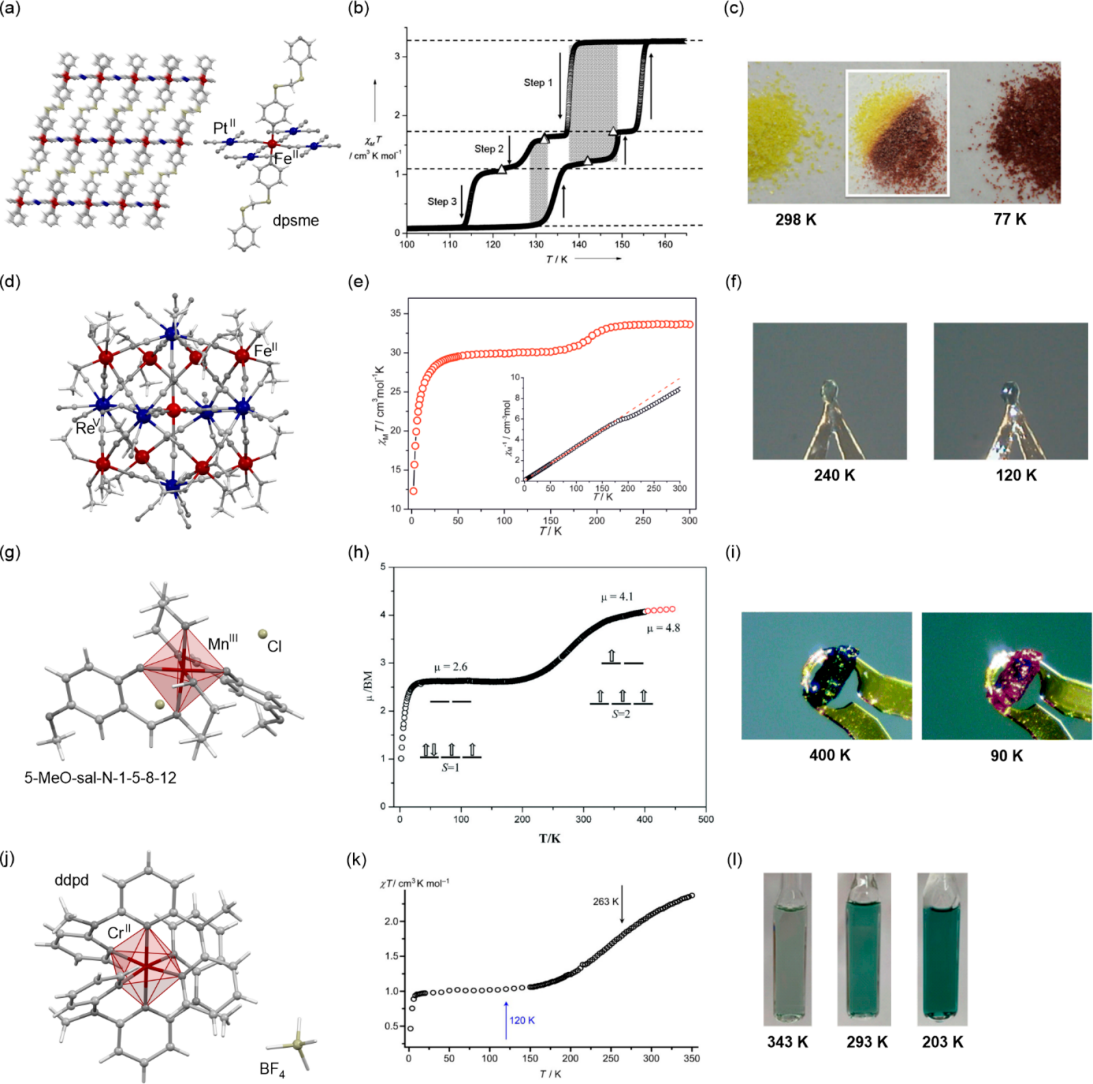
The structural views on {[Fe^II^(dpsme)][Pt^II^(CN)_4_]}·2/3(dpsme)·*x*EtOH·*y*H_2_O (dpsme = 4,4′-di(pyridylthio)methane) coordination framework (a),^315^ shown with the temperature dependence of the *χ*_M_*T* product (b) and photos of the powder sample at indicated temperatures (c), the structure of {Fe^II^_9_(MeOH)_8_[Re^V^(CN)_8_]_6_}·10MeOH coordination cluster (d),^322^ shown with the temperature dependences of the *χ*_M_*T* product and the *χ*_M_^–1^ value (e) and photos of the selected single crystal at indicated temperatures (f), the structural view on [Mn^III^(5-MeO-sal-*N*-1-5-8-12)]Cl (5-MeO-sal-*N*-1-5-8-12 = a Schiff base ligand derived from 5-methoxysalicyl-aldehyde and *N*,*N*′-bis(3-aminopropyl)ethylenediamine) molecular material (g),^331^ presented with the temperature dependence of its magnetic moment (h) and photos of the selected single crystal at indicated temperatures (i), the structure of [Cr^II^(ddpd)_2_](BF_4_)_2_ (ddpd = *N*,*N*′-dimethyl-*N*,*N*′-dipyridine-2-yl-pyridine-2,6-diamine) molecular material (j),^334^ shown together with the temperature dependence of the *χ*_M_*T* product (k) and the photos of the acetonitrile solution of this Cr(II) complex at indicated temperatures (l). Parts (b) and (c) were adapted with permission from ref (315). Copyright 2012 John Wiley & Sons. Parts (e) and (f) were adapted with permission from ref (322). Copyright 2015 John Wiley & Sons. Parts (h) and (i) were reproduced from ref (331) with permission from the Royal Society of Chemistry. Parts (k) and (l) were adapted with permission from ref (334). Copyright 2020 John Wiley & Sons.

The thermal switching of optical absorption occurring upon the spin transition was demonstrated not only for the family of Fe(II)–triazole coordination systems but also for many other Fe(II) complexes and the related coordination frameworks. For instance, the dramatic color change, often from yellow to dark red, was observed for SCO-active Hofmann-type metal–organic frameworks (MOFs) which are typically built of di- or tetracyanidometallates, [M(CN)_*x*_]^*n*−^ (M = Ag^I^, Au^I^, when *x* = 2; M = Pd^II^, Pt^II^, when *x* = 4) and Fe(II) complexes bearing N-donor imine-type ligands, such as pyridine derivatives, or their bridging *N*,*N*-bidentate analogs.^315−321^ In this family of compounds, the thermochromic response is accompanied by the thermal SCO effect of diverse thermal courses, which are represented in magnetic characteristics and range from gradual, weakly cooperative to multi-step, hysteretic behavior, depending on the applied molecular linkers and guest molecules (Figure 2a–c).^315−321^ Using other ligands and metalloligands occupying the coordination sphere of Fe(II) complexes, it was achievable to induce various thermochromic characteristics, including the diverse temperature ranges related to tunable spin transition temperatures as well as diverse colors of the samples, especially in the typically deeply colored LS state.^101,322−327^ The latter tuning effect can be generated even for the incomplete SCO phenomenon as exemplified by nanosized {Fe^II^_9_(MeOH)_8_[Re^V^(CN)_8_]_6_}·10MeOH coordination clusters in which only the single central Fe(II) site undergoes the thermal spin transition, but this is sufficient to provide the large thermochromic response from weakly greenish HS phase to dark blue LS phase (Figure 2d–f).^322^

Besides intensively investigated SCO-active Fe(II) complexes, other transition metal complexes were also found to demonstrate the conjunction of the thermal switching of magnetism and optical absorption due to the spin transition effect.^328−338^ In contrast to Fe(II) centers, the SCO-active Fe(III) complexes (d^5^ valence configuration) do not usually show strong thermochromic response as the broad visible light absorption usually remains for both HS and LS states.^328−330^ A few examples of more impressive thermochromism were detected for less studied cases of SCO-active Mn(III) centers (d^4^ valence configuration) embedded in the mixed coordination environment of N- and O-donor atoms ensured, e.g., by Schiff-base ligands (Figure 2g–i).^331−333^ The great potential for exploration of thermochromism in more exotic spin crossover complexes was further suggested by the heating-induced discoloration of deep green LS phase of Cr(II) centers (d^4^ valence configuration) coordinating two *N*,*N*,*N*-tridentate polypyridine-type ligands (Figure 2j–l).^334^ It is worth noting that in both said cases of Mn(III) and Cr(II) complexes, the spin transition cooperativity is usually limited leading to a rather gradual course of thermal SCO;^331−334^ thus, these systems are promising candidates for optical temperature sensing in the broad working temperature range, including the room-temperature region. The other transition metal complexes, which exhibit the thermal SCO in the non-trivial coordination environment, such as Mn^II^, Co^III^, or Co^II^ (d^5^, d^6^, and d^7^ valence configurations, respectively), can also demonstrate the analogous potential in the thermochromic behavior.^46,336−338^

**Figure 3:**
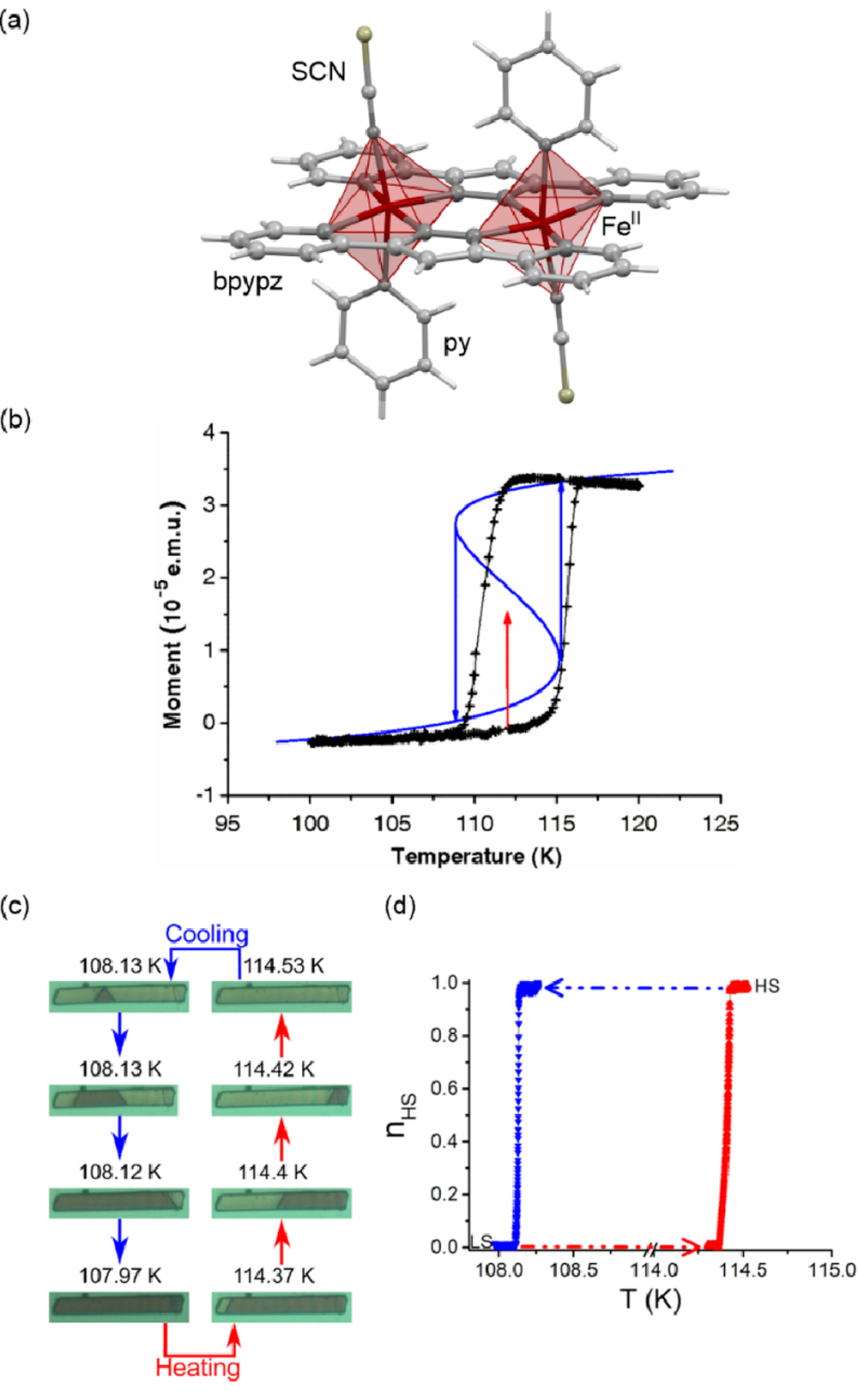
The structure of {[Fe^II^(NCS)(py)]_2_(bpypz)_2_} (bpypz = 3,5-bis(2-pyridyl)pyrazolate; py = pyridine) molecules (a),^339^ the temperature dependence of magnetic moment for the NCSe-analog, shown with the calculated hysteresis loop (blue line) (b), the related optical microscopy snapshots of the crystal along the course of the thermal hysteresis (c), and the temperature dependence of the HS fraction derived from optical density analysis (d). Part (b) was reproduced with permission from ref (340). Copyright 2013 American Physical Society. Parts (c) and (d) were adapted with permission from ref (343). Copyright 2018 American Chemical Society.

Valuable works on thermochromism originating from the thermal spin crossover were presented by K. Boukheddaden and co-workers. They presented the optical studies on the single crystals of dinuclear {[Fe^II^(NCS)(py)]_2_(bpypz)_2_} (bpypz = 3,5-bis(2-pyridyl)pyrazolate) molecules undergoing the abrupt hysteretic thermal SCO (Figure 3ab), focusing on the propagation of thermal spin transition on the needle crystals using optical microscopy.^339−343^ The single-crystal optical studies reproduced the spin transition effect demonstrated primarily by magnetic measurements (Figure 3c,d).^339,340,343^ Moreover, these studies showed that the color change connected with the SCO effect proceeds upon changing the temperature through single-domain nucleation and propagation; in some cases, two equivalent and symmetric HS–LS interface orientations were found. Their propagation velocity was close to a few-to-several μm/s level. The interface could be tuned using the intensity of the irradiation beam of the microscope within a photothermal effect. As a result, the reversible driving of the translational and rotational degrees of freedom of the HS–LS interface was achieved. The translational motion was proved to be accompanied by a crystal’s length change while the rotation of the interface between two stable angles is controlled by the crystal’s bending. The latter effect opens the perspective in the development of robust stress sensors using the switchable interface. The HS–LS front can be also governed by a weak modulated laser intensity providing the ability for the SCO photocontrol inside the thermal hysteresis, an important feature in the aspect of memory devices and switches.^340,342,343^

### 2.2 Electron Transfer Magnetic Materials Exhibiting Switchable Light Absorption

When concerning the switching of the magnetic moment by external stimuli, such as temperature, in molecular materials, heterometallic coordination systems revealing an electron transfer process appear as one of the most promising alternatives. Besides a few types of materials where the electron transfer process between two metal centers, usually linked by a bridging ligand, simply leads to the change in the respective oxidation states, without large structural transformation,^344,345^ there is a continuously growing group of compounds demonstrating a more impressive electron transfer-coupled spin transition (ETCST), alternatively named a charge transfer-induced spin transition (CTIST).^11,49,50,346−351^ Such property was primarily found for cyanido-bridged coordination frameworks based on Co^II^ and Fe^III^ metal centers, i.e. A^I^_x_{Co^II^_y_[Fe^III^(CN)_6_]}·*n*H_2_O (A^I^ = alkali metal ions) Prussian blue analogs (PBAs), and then transferred to bimetallic molecular systems.^11,49,233,347−351^ When these metal ions are embedded in the proper coordination environment and bridged by cyanido ligands, they can reveal the cooling-induced transition from the high-temperature (HT) phase containing the high-spin Co^II^ (HS, *S* = 3/2) and low-spin Fe^III^ (LS, *S* = 1/2) sites (the latter due to the presence of strong-ligand-field C-bonded cyanido ligands) to the low-temperature (LT) phase containing the low-spin Co^III^ (LS, *S* = 0) and low-spin Fe^II^ (LS, *S* = 0) sites. One can easily notice that the combined effect of an electron transfer and spin transition leads to a dramatic change in magnetic properties as the paramagnetic-to-diamagnetic ground state transition is observed.^11,49,347−351^ Similarly to the SCO effect, the ETCST phenomenon provides a drastic change in metal-ligand bond lengths generating the significant expansion/contraction of the crystal lattice. The light absorption is also strongly affected as different electronic transitions are accessible for the metal centers of specific oxidation and spin states in the HT and LT phases. Moreover, the Laporte-allowed metal-to-metal charge transfer (MMCT) electronic transitions, responsible for strong visible-to-NIR absorption bands, are observed for the ETCST materials, and their energies are often different for the HT and LT phases. Therefore, thermochromism is expected to be, in principle, even more efficient when playing with the ETCST systems rather than with the SCO materials presented in the previous section. These MMCT bands were also the basis of the application of ETCST systems in the context of photoswitching of physical properties, which was first recognized for the above-mentioned cyanido-bridged Co^II^–Fe^III^ networks (see section 3).^233,351,352^ The limited family of ETCST systems, similar to the SCO complexes, was also studied from the point of their transfer into the nanoscale. Although finite clusters or molecules in this context are rarely exploited, well-known three-dimensional cyanido-bridged frameworks, i.e., Prussian blue analogs, were studied to form nanoparticles and the related core@shell heterostructures. By these means, an additional possibility to modify the ETCST phenomenon by the particle size control and core/shell selection, and to generate photoinduced magnetization for nanosized objects appeared as an alternative to the SCO complexes.^353−357^

**Figure 4:**
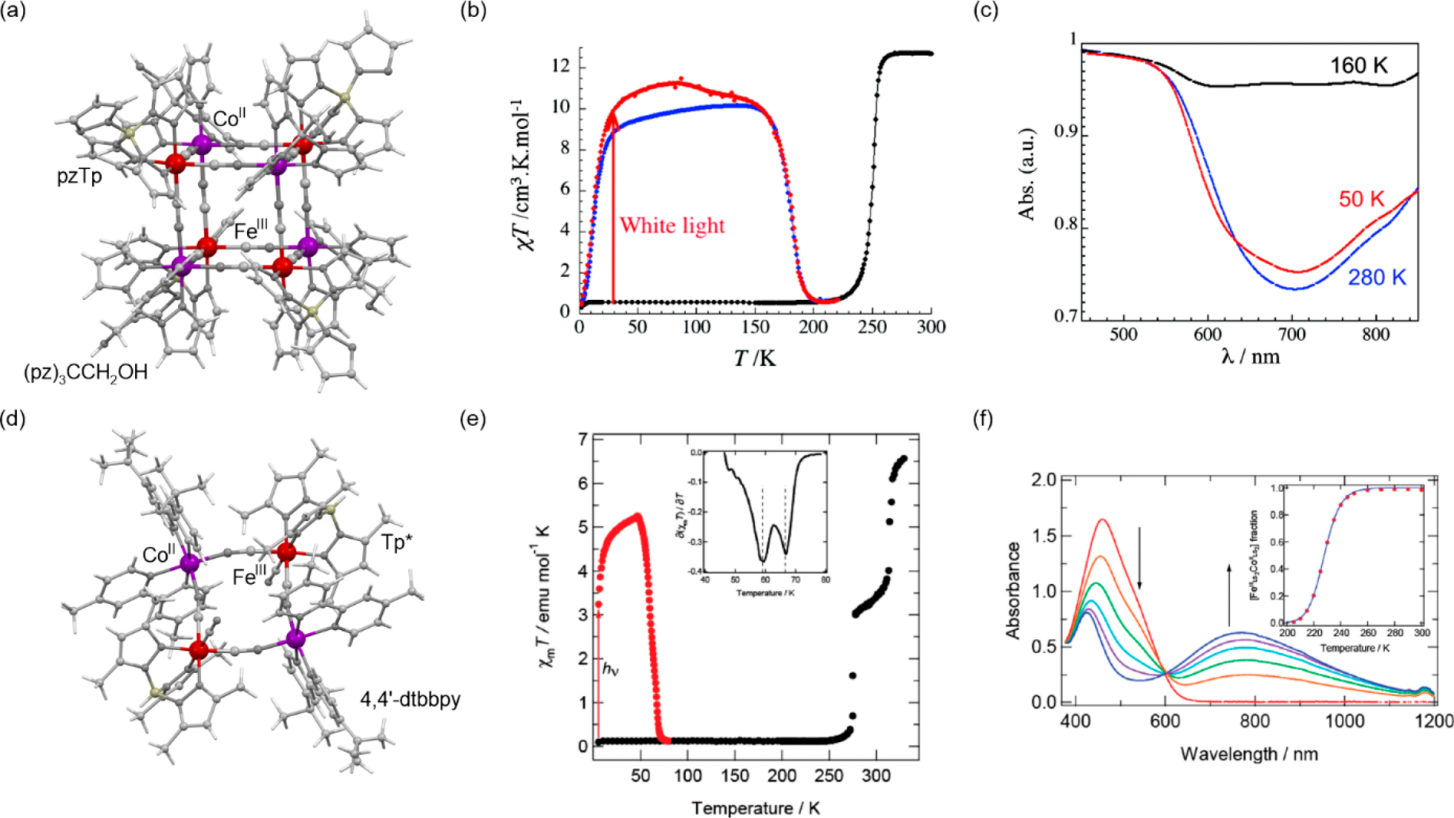
The structure of {[(pzTp)Fe^III^(CN)_3_]_4_[Co^II^(pz)_3_CCH_2_OH]_4_}(ClO_4_)_4_·13dmf·4H_2_O (pzTp = tetrapyrazolylborate; (pz)_3_CCH_2_OH = tris-2,2,2-(1-pyrazoyl)ethanol) molecular cube (a),^51^ shown with its temperature dependence of the *χ*_M_*T* product, including also the results of a photomagnetic experiment with white light (b), and surface light absorption spectra at three indicated temperatures (c), the structure of {[Co^II^_2_Fe^III^_2_(CN)_6_(Tp*)_2_(dtbbpy)_4_]}(PF_6_)_2_·2MeOH (Tp* = hydrotris(3,5-dimethylpyrazol-1-yl)borate, dtbbpy = 4,4′-di-*tert*-butyl-2,2′-bipyridine) molecular square (d),^358^ presented with its temperature dependence of the *χ*_M_*T* product, including also the curve after photoirradiation at low temperatures (e), and temperature-variable UV–vis-NIR absorption spectra, together with the temperature dependence of the [Co^II,HS^_2_Fe^III,LS^_2_] fraction estimated by the analyses of the temperature variable absorption intensity at 770 nm (f). Parts (b) and (c) were adapted with permission from ref (51). Copyright 2008 American Chemical Society. Parts (e) and (f) were adapted with permission from ref (358). Copyright 2011 American Chemical Society.

Despite these promising expectations, the studies on the thermochromic phenomena and related applications of ETCST/CTIST materials are limited when compared with SCO systems. Among archetypical Co–Fe PBAs, for a Na_0.37_{Co^II^_1.37_[Fe^III^(CN)_6_]_0.89_[Fe^II^(CN)_6_]_0.11_}·4.8H_2_O framework, the presence of thermally induced ETCST was reported, which is accompanied by the subtle changes within the UV–vis absorption spectra between 290 and 50 K.^49^ As at both temperatures the obtained optical absorption bands are rather broad, which is partially related to the mixed-valence character of the metal centers, Co–Fe PBAs were barely exploited from this viewpoint. However, the thermal switching of visible-to-NIR light absorption was presented quite frequently for CT-active Co^II^–Fe^III^ systems with a decreased coordination dimensionality (Figure 4).^51,358^ For instance, D. Li et al. reported the {[(pzTp)Fe^III^(CN)_3_]_4_[Co^II^(pz)_3_CCH_2_OH]_4_}(ClO_4_)_4_·13dmf·4H_2_O (pzTp = tetrapyrazolylborate; (pz)_3_CCH_2_OH = tris-2,2,2-(1-pyrazoyl)ethanol, Figure 4a) cuboidal clusters, undergoing the abrupt thermal ETCST effect below 250 K, which was further found to be photoreversible at low temperatures (Figure 4b). The surface absorption spectra revealed the appearance of a strong and broad absorption band at ca. 700 nm which was assigned to the new MMCT transition occurring between Co^III,LS^ and Fe^II,LS^ centers formed in the LT phase (Figure 4c). Interestingly, on further cooling the sample below 100 K, this band started to disappear again; thus, at 50 K, the light absorption properties are similar to those detected for the HT phase. This unusual behavior was explained by the returning conversion from the Co^III,LS^–Fe^II,LS^ pair to the original Co^II,HS^–Fe^III,LS^ pair upon the irradiation by light needed to gather the spectra.^51^

The distinct thermochromism related to the ETCST effect was presented by M. Nihei et al. for {[Co^II^_2_Fe^III^_2_(CN)_6_(Tp*)_2_(dtbbpy)_4_]}(PF_6_)_2_·2MeOH (Tp* = hydrotris(3,5-dimethyl pyrazol-1-yl)borate, dtbbpy = 4,4′-di-*tert*-butyl-2,2′-bipyridine) square molecules (Figure 4d). They exhibit two-step abrupt thermal ETCST in the 250–350 K range, also partially reversible by light at low temperatures (Figure 4e). The related thermochromism could be traced upon cooling the butyronitrile solution of these molecular objects (Figure 4f). Similarly to the previous case, this compound exhibits the cooling-induced appearance of the strong broad band ranging from 600 to almost 1100 nm, which can be ascribed to the MMCT transition within the generated LT phase. Simultaneously, the strong band centered around 450 nm gradually disappears upon cooling; thus, it was assigned to the analogous CT band between Co^II,HS^ and Fe^III,LS^ centers formed in the HT phase. The absorbance at the selected wavelength of 770 nm shows an abrupt increase in the 240–210 K range, which indicates the occurrence of the ETCST in the solution leading to a thermochromic effect.^358^ Analogous thermal switching of light absorption was found in the substantial set of other CT-active Co^II^–Fe^III^ frameworks.^348,352,359−363^

The thermally activated intermetallic electron transfer connected with the spin transition within the ETCST process was also presented for heterometallic d–d hexacyanidometallate-based coordination systems exploring Fe^II/III^–Os^III/II^ and Co^II/III^–Os^III/II^ redox pairs but thermochromism related to their broad absorption was not precisely investigated.^364,365^ On the other hand, the distinct thermal switching of light absorption, visualized by a cooling-induced color change from red to blue, was found for the unique case of Ni^II^–[Fe^III^(CN)_6_]^3–^ systems undergoing the electron transfer phase transition to the LT phase containing Ni^III^ and Fe^II^ centers. This effect happens only in the hydrated form of a supramolecular framework while the dehydration of the sample blocks this thermally controlled process (see section 6.1).^366^ The analogous electron transfer effect, occurring without the subsequent spin transition, was demonstrated for the deeply investigated family of multifunctional Rb^I^_*x*_{Mn^II/III^[Fe^III/II^(CN)_6_]_(*x*+2)/3_}·H_2_O Prussian blue analogs (PBAs).^367−372^ They undergo a highly cooperative charge transfer phase transition from the HT phase containing Mn^II,HS^ and Fe^III,LS^ centers to the LT phase containing Mn^III,HS^ and Fe^II,LS^ centers, the latter accompanied by the crucial strong Jahn-Teller distortion within Mn^III,HS^ complexes. Besides the variety of magnetic, mechanical, electrical, and nonlinear optical properties affected by this cooling-induced transition, light absorption is also distinguishable for the HT and LT phases, especially from the viewpoint of the overall absorption intensity.^367^ Therefore, these systems are promising thermochromic materials, which was not yet fully explored because the main attention was devoted to the research on their visible-light-induced reversible photomagnetism (see section 3.2).^367,373^

**Figure 5:**
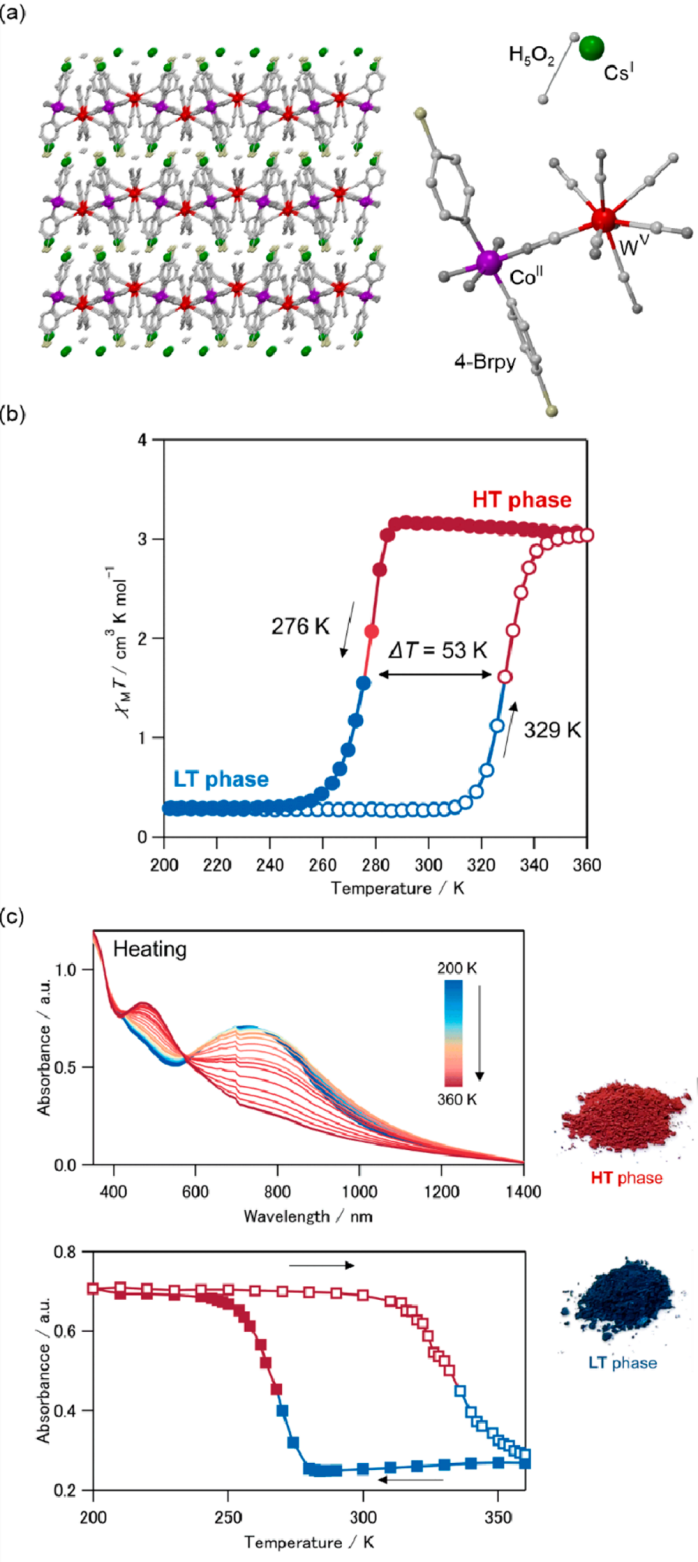
The structure of Cs^I^_0.1_(H_5_O_2_)_0.9_{[Co^II^(4-Brpy)_2.3_][W^V^(CN)_8_]} (4-Brpy = 4-bromo-pyridine) layered coordination polymer (a),^377^ its thermal hysteresis loop on the temperature dependence of the *χ*_M_*T* product (b), and the related thermal changes in the UV–vis-NIR absorption spectra shown together with the temperature dependence of the absorbance at 760 nm and the photos of the high- (HT) and low-temperature (LT) phases of the material (c). Parts (b) and (c) were reproduced from ref (377) with permission from the Royal Society of Chemistry.

In 2003, the first thermal ETCST/CTIST effect outside the family of hexacyanidometallate-based coordination assemblies was presented. In the related article of Y. Arimoto et al., heterometallic cyanido-bridged Cs^I^{[Co^II^(3-cyanopyridine)_2_][W^V^(CN)_8_]}·H_2_O layered framework was shown to exhibit the cooling-induced transition from the HT phase containing the Co^II,HS^ (*S* = 3/2) and W^V^ (*S* = 1/2) pair to the LT phase containing the Co^III,LS^ (*S* = 0) and W^IV^ (*S* = 0) pair.^346^ In the analogous way to the CT-active Co^II^–Fe^III^ frameworks, the resulting thermal transition leads to both the paramagnet-to-diamagnet switching as well as the concomitant change of the sample’s color. The modified light absorption properties were explained by the appearance of novel MMCT absorption bands in the LT phase which was further applied for the photoinduced magnetization effect. Throughout the next twenty years, the analogous CTIST effect was found in a few other examples of Co^II^–W^V^ coordination systems; however, their thermochromism was only roughly pointed out.^52,374−376^ In most cases, the HT-to-LT phase transition leads to the color change from red (or violet) to blue which was mainly ascribed to the cooling-induced appearance of the new Co^III,LS^–W^IV^ MMCT band. Very recently, K. Nakamura et al. presented more precise studies on the thermal switching of light absorption in the Cs^I^_0.1_(H_5_O_2_)_0.9_{[Co^II^(4-Brpy)_2.3_][W^V^(CN)_8_]} (4-Brpy = 4-bromopyridine) layered coordination polymer exhibiting the highly cooperative ETCST around the room temperature (Figure 5ab).^377^ The cooling of the red-colored HT phase leads to the gradual appearance of the new Co^III,LS^–W^IV^ MMCT band around 770 nm while the higher energy absorption, assignable to the Co^II,HS^–W^V^ MMCT, synchronously disappears which provides the color change to deep blue for the LT phase (Figure 5c). In this compound, the high transition cooperativity gives the pronounced thermal hysteresis loop in magnetic measurements, which was also reflected in the light absorption property. Therefore, the room-temperature bistability could be perfectly observed both in magnetic and optical studies.^377^ The utility of the Co^II^–W^V^ redox pair in the generation of the ETCST effect was also transferred into nanosized pentadecanuclear clusters of the {Co^II^_9_[W^V^(CN)_8_]_6_} core;^378,379^ however, in this case, the admixture of intracluster Fe^II^ centers was needed to induce the charge transfer phase transition. As a result, broad absorption bands, representing the mixture of Fe^II/III^–W^V/IV^ and Co^II/III^–W^V/IV^ MMCT transitions, were observed, providing a less pronounced thermochromism.

In this context, even broader absorption bands and their rather subtle thermal switching were demonstrated for the other class of electron transfer molecular materials, named valence tautomeric (VT) complexes.^380−386^ These systems are usually constructed of cobalt ions and redox-active organic ligands from the dioxolene group. In the HT phase, the Co^II,HS^ centers coordinate paramagnetic semiquinonate (SQ) ligands, while, upon the cooling-induced metal-to-ligand electron transfer, the LT phase consisting of the Co^III,LS^ centers that coordinate diamagnetic catecholate (cat) ligands is formed. Thus, the VT transition leads to the spin transition on Co sites giving the distinct change in magnetic properties.^380−386^ The synchronous switching of light absorption is observed mainly due to the thermal modulation of the charge transfer electronic transitions occurring between the metal center and the ligand. However, the related thermochromism is usually moderate, especially in comparison to the SCO and Co–Fe/Co–W CTIST systems.^384−386^

On the other hand, the VT transition cooperativity is typically weak which provides the gradual and often near-room-temperature course of the thermally-induced spin transition effect. This makes the VT compounds promising candidates for thermochromism-related applications, e.g., absorption-based temperature sensors. Another class of magnetic molecule-based materials is composed of homometallic electron-transfer systems, especially those based on the Fe(II)/Fe(III) pairs, which are recognized for their advanced redox activity in solution.^387^ The related Fe(II)/Fe(III) ratio can be tuned by the change of the temperature within the usually gradual thermal ET effect. Thus, the good potential in thermochromism is also expected to be uncovered;^388^ however, the difficulty in the realization of this pathway can appear due to the very broad absorption bands originating from the intervalence charge transfer transitions.

## 3 Photoswitching of Molecule-Based Magnetic Materials

For stimuli-responsive materials, optical control over the physical features has immense advantages that govern the continuous development of this field in the context of a wide range of future applications.^389−394^ Light as a physical stimulus enables to amend the crystal structure of the material at the molecular level, but owing to the specified power, energy, and even polarization of the selected radiation, allows to precisely excite the electronic states involved in such a process, which stays in contrast to the change of temperature. Differently from electric current/field, hydrostatic pressure, or mechanical stress, employing light stimulus does not require to have direct contact with the material, while its cost efficiency is much better than that of the magnetic field. At the same time as all physical stimuli, electromagnetic radiation has a much wider scope of possible applications in comparison to chemical ones (pH, solvent vapors, gases, anions, small molecules, etc.).^395^ Due to the recent development of efficient light sources, such as femtosecond lasers and high-power light-emitting diodes, within the last two decades, one can observe increasing scientific attention toward studies of photophysical and photochemical processes with photoswitchable systems serving as a future alternative for various devices of everyday use and technological applications.^396−398^ This includes optical memory devices,^399−402^ data encryption,^403−405^ logic gates,^406−408^ optical communication,^409^ switchable sensors as well as biomaterials,^410−414^ and materials for photocatalysis.^415−418^

Light, being often orthogonal to different physicochemical stimuli, especially well suits the construction of dual-property or multi-stimuli-responsive materials.^419−422^ In terms of the molecule-based magnetic materials it was found suitable to photocontrol diverse magnetic phenomena.^97^ Some of them were described in the previous section, such as the spin-crossover effect of d^4^–d^7^ metal complexes or the electron transfer-coupled spin transition (ETCST) effect between d-block metal centers.^50,423^ Moreover, chemists working with photoswitchable magnetic materials often employ photochromic organic molecules, serving as ligands or linkers for spin-bearing metal ions and less often even radicals.^97,171^ In this aspect, the use of molecules showing photocyclization, such as dithienylethene group,^424−427^ or photoisomerization, e.g., azobenzene derivatives, seems to be currently the most efficient synthetic strategy.^428−430^

In terms of photoresponsive materials, an important aspect is the reproducibility of the initial state. A common relaxation of the photoexcited state occurs due to the network vibrations, thus often the metastable state at a certain temperature relaxes to the ground one upon heating. The photoinduced transformations within molecule-based systems are usually related to the specific electronic transitions of the optical absorption spectra, thus the related photochromic behavior in some cases may be reversed by employing a different wavelength of irradiation. It is noteworthy to mention the occurrence of the structural rearrangement in the photoexcited state which for the spin transition and electron-transfer-assisted phenomena is usually limited mainly to the modulation of metal–ligand bonding lengths, while for the extended organic molecules involving photochromic moieties seriously affects the supramolecular network. Therefore, the latter photoactivity is much harder to design in the solid state, as well as the photoreversibility is often limited to some extent. Having that in mind, some mechanisms of efficient photoswitching in the solution were up to now not presented for the crystalline materials.^50,97,171,423−430^

Within this section, the reported photoswitchable molecule-based magnetic materials were divided into three groups related to the nature of the photoswitching effect. In section 3.1, spin-crossover systems showing the light-induced excited spin-state trapping (LIESST) effect are discussed. Charge-transfer systems, including those of the ETCST mechanism, as well as reports explaining the related kinetics, were gathered in section 3.2. The final section (3.3) contains the selected molecule-based systems incorporating photoswitchable organic ligands and molecules tuning their magnetic features. Within each section, the tuning of the slow relaxation of the magnetization effects in molecular nanomagnets was distinguished from the other phenomena to expose the increasing significance of such opto-magnetic coupling.

### 3.1 Optical Switching Related to Photoresponsive Metal Centers

Among molecule-based magnetic materials, spin-crossover (SCO) systems are continuously studied due to their potential applications in many aspects of life and technology (see also section 2).^431−433^ Apart from the SCO-related use of temperature to switch magnetic properties, optical features, dielectric characteristics, and structural parameters, other physical stimuli may be employed for changing the spin state, such as pressure,^434^ magnetic or electric field,^102,145,435^ as well as light.^436^ In a model case, electromagnetic radiation can influence the spin state of the system at a temperature below the SCO transition by exciting the low-spin (LS) state into the high-spin (HS) one, resulting in its metastability within the effect named light-induced excited spin-state trapping (LIESST). While elevating the temperature, the relaxation rate increases, resulting in a reverse transition from HS to LS configuration above the critical temperature, *T*_LIESST_.^423,436^ Another relaxation pathway sometimes present in photoactive SCO systems involves the use of a different light wavelength, quenching the metastable HS state and stabilizing the ground one.^437^ Aiming at memory and spintronic devices, various SCO systems were processed into the nanoscale as thin films or even adsorbed at surfaces or in carbon nanotubes.^102,438^ It was proven that the switching properties within the LIESST mechanism can be maintained in the related nanomaterials.^102,439−445^ The read-out of the spin state in such devices most often employs the change of the conduction between both states of the molecular unit,^102,438,444^ alternatively, the differences in the X-ray absorption spectra are explored.^439,441^ Nevertheless, the most crucial aspect is the stability of the SCO molecules upon deposition in the real device. In this context, different ligands and substrates are studied to optimize the interactions with SCO-active compounds. Despite those recent advances in processing SCO materials into the nanoscale, basic studies involving the synthesis of novel photoswitchable systems and their bulk properties still play a pivotal role. In this section, we make the overview of non-trivial physical effects related to spin state switching by employing light that governs the SCO phenomenon.

#### 3.1.1 Spin Transition Materials Exploring Photoresponsive Metal Centers

While the spin-crossover effect is known for the wide range of complexes of various transition metal ions of d^4^–d^7^ valence configurations, the number of metal ions for which the LIESST effect was reported is quite limited. The broadest family involves Fe(II)-based SCO materials, for which the correlations of LIESST with structural features as well as with the *T*_1/2_ temperature of the thermal spin transition were presented.^423,446,447^ Additionally, among different criteria, the spin-orbit coupling, the enthalpy difference between the HS and LS states, and the transition cooperativity were lately depicted as critical for designing high *T*_LIESST_ values.^436^ Interestingly, the calculations of the energy curves with respect to the metal-ligand distances between Fe(II) and tetrazole ligands were reported to reveal a similar LIESST mechanism when the metallic center was exchanged by Fe(III) one.^437^ This stays in agreement with the fact that Fe(III) complexes are the second group of SCO systems where the LIESST effect was observed. Even though the first example of the Fe(II) complex showing LIESST in the solid state was presented in 1984 by A. Hauser and co-workers,^448^ followed by the report regarding a reverse-LIESST in 1986,^449^ it took 16 years to observe the same effect for a Fe(III) system,^450^ and still such systems are rather scarce.^451^ This is due to the much smaller change in the metal-ligand distance upon changing the spin state, resulting in a much smaller energy barrier between HS and LS configuration energy curves. To induce the efficient LIESST effect, an additional distortion of the coordination octahedron in the metastable excited state was highlighted. Moreover, a constructive role of intermolecular π–π or halogen interactions was suggested.^452,453^ A similar issue concerns other SCO systems, as to the best of our knowledge, none of the reported Mn(II/III), Cr(II), or Co(II/III) SCO-active systems was found to exhibit the LIESST effect.

**Figure 6:**
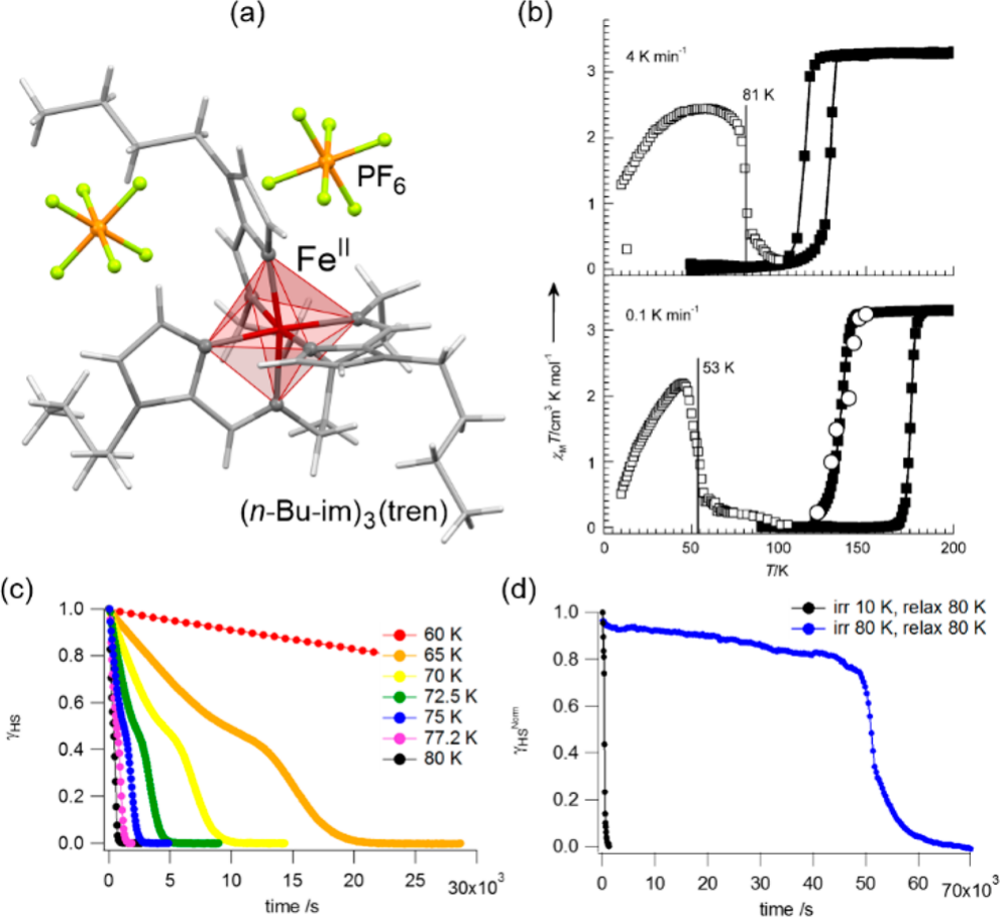
The molecular structure of [Fe^II^(*n*-Bu-im)_3_(tren)](PF_6_)_2_ ((*n*-Bu-im)_3_(tren) = *n-*butyl-imidazoltris(2-ethylamino)amine) coordination system (a),^454^ its temperature dependence of the *χ*_M_*T* product, measured at the rate of 4 K min^–1^ (upper part) and 0.1 K min^–1^ (bottom part), presented together with the *χ*_M_*T*(*T*) curves for the photogenerated at 10 K LIESST phases (b), temperature-variable time evolution of the high-spin Fe(II) fraction, *γ*_HS_, photogenerated within the LIESST effect at 10 K (c), and the comparison between the time evolution of *γ*_HS_ at 80 K for the samples irradiated at 10 K and at 80 K (d). Part (b) was adapted with permission from ref (454). Copyright 2013 John Wiley & Sons. Parts (c) and (d) were adapted with permission from ref (455). Copyright 2018 American Chemical Society.

The influence of subtle structural aspects upon both thermal and light-induced spin-crossover was well demonstrated for iron(II) complexes. In 2013, J. A. Real and co-workers presented an octahedral Fe(II) system, [Fe^II^(*n*-Bu-im)_3_(tren)](PF_6_)_2_ ((*n*-Bu-im)_3_(tren) = *n-*butylimidazoltris(2-ethyl-amino)amine), for which both thermal hysteresis loop and *T*_LIESST_ were strongly affected by the cooling rate of the material (Figure 6).^454^ This was explained by the presence of a structural phase transition, upon lowering the temperature, related to the presence of alkyl groups. The difference in cooling rates resulted in the formation of the modified LS phases with their own LIESST characteristics. Moreover, the same system was reported to exhibit a much longer relaxation time when irradiated at 80 K instead of 10 K.^455^ When the sample was cooled at the rate of 0.1 K/min reaching the LS2 phase, no difference in the relaxation at 45 K was observed wherever the irradiation was performed at this temperature or at 10 K.

On the contrary, for the LS1 phase, obtained by cooling at the rate of 4 K/min, the system photoexcited at 10 K fully relaxes in a few minutes when elevating the temperature above 70 K. However, the LS1 sample irradiated at 80 K remains in its metastable HS state for tens of hours. Photocrystallographic studies revealed that the irradiation at 10 K does not affect the conformation of the butyl groups, which remained ordered for the obtained HS state. For the crystal irradiated at 90 K one of the alkyl groups changes its conformation, while another one was found in a structural disorder. This suggests that the increased metastability at the elevated temperature is connected with an additional energy barrier of the structural reorganization on the relaxation transition path from the HS to the LS state. Moreover, the disordered phase of the HS state can be obtained by fast cooling, and its relaxation rate is similar to the one obtained by photoirradiation at 90 K.^455^

Another example of a structural rearrangement resulting in a high relaxation temperature for the photogenerated HS phase was found for pentagonal bipyramidal [Fe^II^(L_5_)(CN)_2_] (L_5_ = N_3_O_2_ or N_5_ Shiff-base macrocyclic ligand) complexes.^456−460^ For these systems, cyanido ligands are *trans*-positioned, while the macrocyclic ligand occupies the equatorial of the complex. Structural studies revealed, that only four donor atoms of the organic ligand are coordinated in the LS phase to the Fe(II) center, while after reaching the HS state either thermally or after irradiation at cryogenic temperatures, the fifth O-/N-donor atom binds the central ion.^460^ Such change in the first coordination sphere upon spin transition results in *T*_LIESST_ values above 100 K, even when in the whole range below room temperature the complex stays in its LS state.^456,457,459^ On the other hand, distinct modification of the second coordination sphere such as intraligand proton transfer upon thermal and light-induced spin transition was reported for the [Fe^II^(HLCl)_2_](AsF_6_)_2_ system containing *N*′-(di(pyridin-2-yl)methylene)-2-chlorobenzohydrazide (HLCl) ligands.^461^ Upon the LS to HS transformation, the spin state change induces a hydrogen atom relocation from the non-coordinated pyridine ring to the coordinated amine group. Reverse ligand transformation occurs due to thermal quenching of the metastable excited state at ca. 85 K.

An unprecedented example of the photoinduced spin transition appears also for [M^IV^(CN)_8_]^4–^ (M = Mo, W) anions. These complexes do not reveal thermal SCO behavior and remain in their LS state due to the strong ligand field generated by the C-bonded cyanido ligands. However, V. Marvaud and co-workers reported for the {[Zn^II^(tren)_2_]_2_[Mo^IV^(CN)_8_]} molecule a photomagnetic behavior at cryogenic temperatures, and a similar behavior was also found for the [W^IV^(CN)_8_]^4–^-based chain system.^462,463^ The LIESST effect between *S* = 0 and *S* = 1 was proposed, but its reversibility required elevation of the temperature. Later, D. Pinkowicz, C. Mathonière, and co-workers found that even the K_4_[Mo^IV^(CN)_8_]·2H_2_O salt reveals a light-induced change of the magnetic moment upon 405 nm light irradiation.^464^ The mechanism of such behavior was attributed to the photodissociation of one cyanido ligand, resulting in the heptacoordinated paramagnetic [Mo^IV^(CN)_7_]^3–^ ion, which was proven employing photocrystallographic studies. As an analogous paramagnetic [W^IV^(CN)_7_]^3–^ anion was previously isolated,^465^ one may expect a similar mechanism of the light-induced spin changes for both Mo^IV^ and W^IV^ complexes.

**Figure 7:**
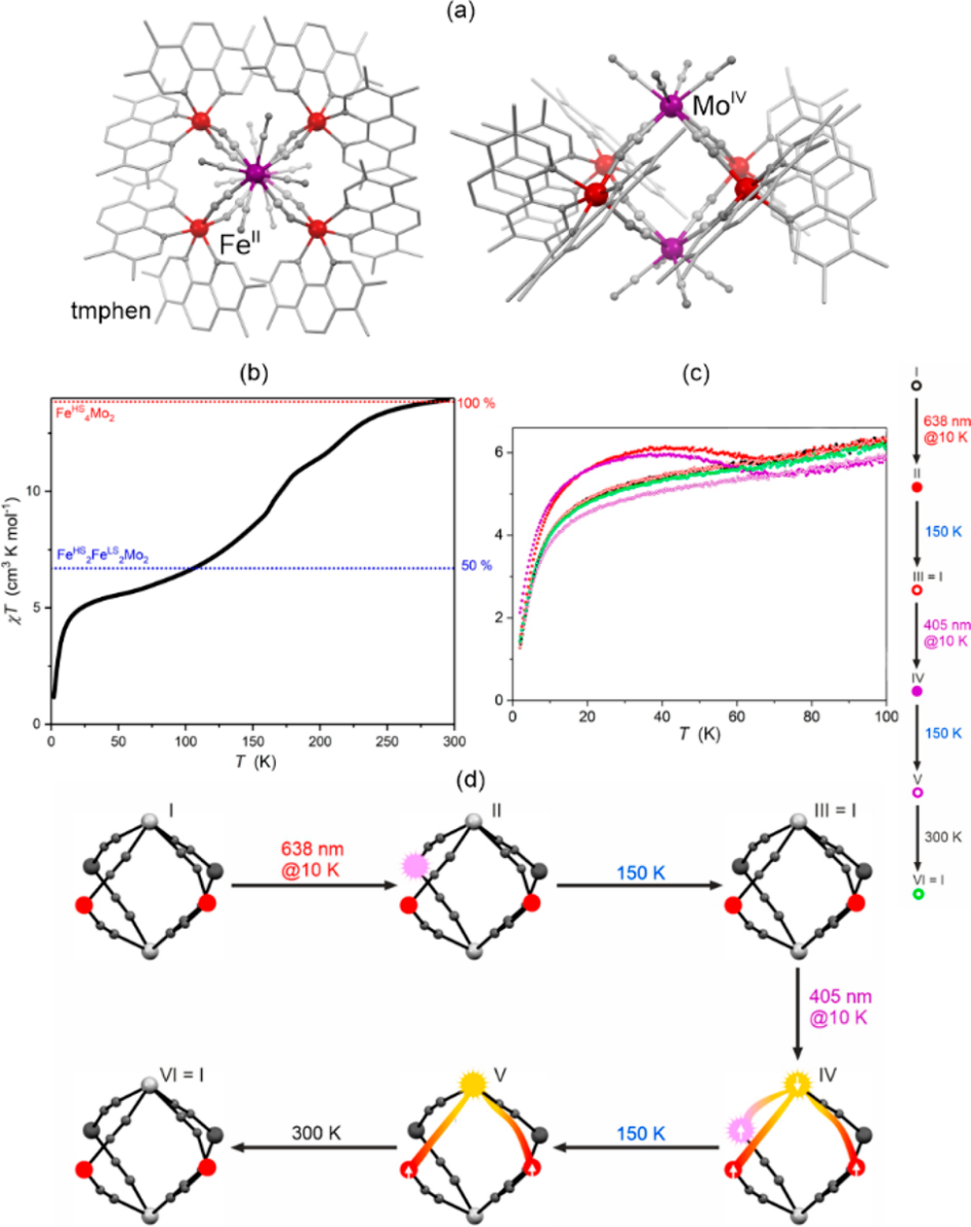
The structural views on {[Fe^II^(tmphen)_2_]_4_[Mo^IV^(CN)_8_]_2_}·solv (tmphen = 3,4,7,8–tetramethyl-1,10-phenanthroline; solv = crystallization solvent, MeOH/H_2_O) hexanuclear coordination cluster (a),^466^ the temperature dependence of the *χ*_M_*T* product, shown together with the comparison between the *χ*_M_*T*(*T*) curves collected before irradiation and in different parts of the photomagnetic experimental sequence shown on the right (c), and the schematic representation of the site-selective photoswitching effect (d). Parts (b), (c), and (d) were adapted with permission from ref (466). Copyright 2019 American Chemical Society.

Polycyanidometallates of d-block metal ions readily form bimetallic systems with other d- and f-block metal complexes. In this context, octacyanidomolybdate(IV) was used by K. R. Dunbar, D. Pinkowicz, and co-workers to construct octahedral molecules with Fe^2+^ ions and 3,4,7,8–tetramethyl-1,10-phenanthroline ligands (Figure 7).^466^ Each cluster of these systems is built of cyanido-bridged four Fe(II) and two Mo(IV) centers. Upon lowering the temperature an extremely gradual spin-transition on only two Fe(II) sites occurs, which was found similar in the analogs built of W(IV) and Nb(IV) complexes.^466,467^ Upon light irradiation at 10 K, the Mo(IV) system reveals a reasonable increase in magnetic moment. Irradiation with 638 nm light induces LIESST effect on the Fe(II) site that relaxes thermally upon heating to 150 K, while 405 nm laser excites both Fe(II) and Mo(IV) centers, resulting in antiferromagnetic interactions between them. Again, elevating the temperature to 150 K relaxes the Fe(II) center but, to return to the pristine state, annealing at 300 K is necessary due to the higher relaxation temperature for the Mo(IV) center. For the W(IV) congener a similar photomagnetic behavior was observed, but its reversibility was incomplete, while the [Nb^IV^(CN)_8_]^4–^ ions are photostable. For the Mo(IV)-based cluster, overall four magnetically different states are accessible by light and temperature combination.^466^

**Figure 8:**
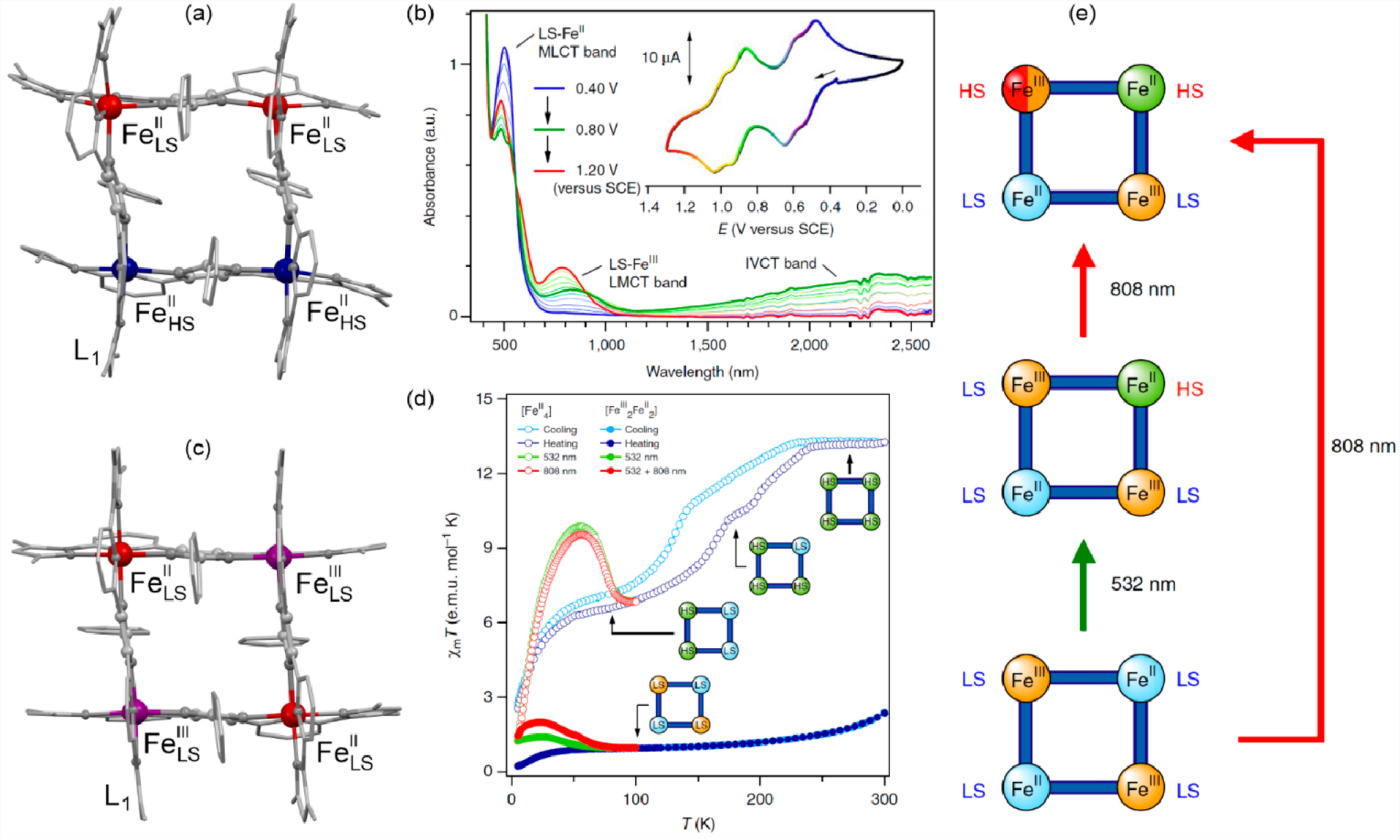
The structure of {[Fe^II^_4_(L_1_)_4_]}(BF_4_)_4_·2MeCN ([Fe^II^_4_]) (HL_1_ = 2-phenyl-4,5-bis{6-(3,5-dimethyl-1H-pyrazol-1-yl)pyrid-2-yl}-1H-imidazole) molecular square at 100 K (a),^469^ electric-potential-variable UV–vis-NIR absorption spectra of the [Fe^II^_4_] molecules in acetonitrile (b), the structure of {[Fe^III^_2_Fe^II^_2_(L_1_)_4_]}(BF_4_)_6_·6MeNO_2_·Et_2_O·4H_2_O ([Fe^III^_2_Fe^II^_2_]) molecular square at 100 K (c),^469^ the related *χ*_M_*T*(*T*) curves for the [Fe^II^_4_] and [Fe^III^_2_Fe^II^_2_] systems in the dark and after photoirradiation at 5 K (d), and the mechanism of the site-selective photoswitching effect for the [Fe^III^_2_Fe^II^_2_] molecular squares (e). Parts (b), (d), and (e) were reproduced with permission from ref (469) under terms of the CC-BY license. Copyright 2014 Springer Nature.

In mixed valence systems containing both iron(II) and iron(III) centers one may expect a similar behavior as both those metal ions can exhibit temperature- and light-induced SCO.^468−470^ However, enforcing SCO for both these centers within a single crystalline material seems hard to achieve. As such, for example, G. Chastanet, M. Halcrow, and co-workers reported a supramolecular cubic architecture of {[Fe^III^(H_2_O)][Fe^II^_8_(μ-L)_12_][BF_4_]_7_} (HL = 4,6-di(pyrazol-1-yl)-1H-pyrimid-2-one or 4,6-di(4-methylpyrazol-1-yl)-1H-pyrimid-2-one) where only the external Fe(II) sites reveal partial SCO behavior activated by temperature and light, while the Fe(III) aqua complexes remain in the HS state within the whole stability range.^468^ Nevertheless, the desired site-selective switching of SCO properties realized for Fe(II)/Fe(III) pairs was achieved by H. Oshio and co-workers.^469^ By using Fe^2+^ ions and deprotonated 2-phenyl-4,5-bis{6-(3,5-dimethyl-1H-pyrazol-1-yl)pyrid-2-yl}-1H-imidazole, a molecular square [Fe^II^_4_] was obtained. It was found to reveal a gradual thermal SCO involving two Fe(II) centers and only one of them being photoactive in the LS state at cryogenic temperatures (Figure 8). However, the cyclic voltammetry showed relative stability for the doubly oxidized [Fe^III^_2_Fe^II^_2_] species in the acetonitrile, thus such a system was obtained by oxidizing the pristine one in solution. Below 300 K, the Fe centers of both oxidation states remain at the LS state with only the onset of the spin transition around room temperature. Upon 532 nm light irradiation at 5 K, the [Fe^III^_2_Fe^II^_2_] squares undergo partial LIESST effect involving only one Fe^II^ site. When changing the excitation wavelength to 808 nm one Fe^III^ site also achieves the HS state. The result of light irradiation was also confirmed by the Mössbauer spectroscopy. However, in the case of [Fe^III^_2_Fe^II^_2_] squares similarity between relaxation temperatures of Fe(II) and Fe(III) HS centers, combined with the overlap between MLCT Fe(II) bands and LMCT Fe(III) bands, disabled the observation of the fourth state consisting of LS Fe(II) centers and HS Fe(III) ones.^469^

In a vast number of SCO systems, especially when embedded in the rigid coordination framework, the thermal spin-crossover effect may occur in a step-wise fashion.^315,471−475^ This may be either caused by the structural inequivalence of SCO sites or by the local stiffening of the network upon bond length shortening for the part of metal centers. Similar behavior can be rarely observed within the LIESST effect, as in the already presented case of site-selective switching materials. Light-induced SCO effect can be sometimes found in Hofmann-type metal–organic frameworks (MOFs) composed of Fe(II) sites, tetracyanidometallate(II) ions, [M^II^(CN)_4_]^2–^ (M = Ni, Pd, Pt), and supporting or bridging N-donor ligands.^475^ However, in some cases even with thermal multi-step SCO, the light-induced process involves all the LS iron(II) sites at the same time.^475^ Due to large transition cooperativity, Hofmann clathrates reveal abrupt thermal SCO transitions but their LIESST properties are less frequent. Despite this, highly organized bimetallic systems, such as Hofmann-type MOF of {[Fe^II^(3-Clpy)_2_][Pd^II^(CN)_4_]} and the relative Cs^I^{[Fe^II^(3-CNpy)_2_][Re^V^(CN)_8_]}·H_2_O coordination polymer, were shown to reveal multi-step SCO modulated by temperature, light, and external pressure.^268,476^

**Figure 9:**
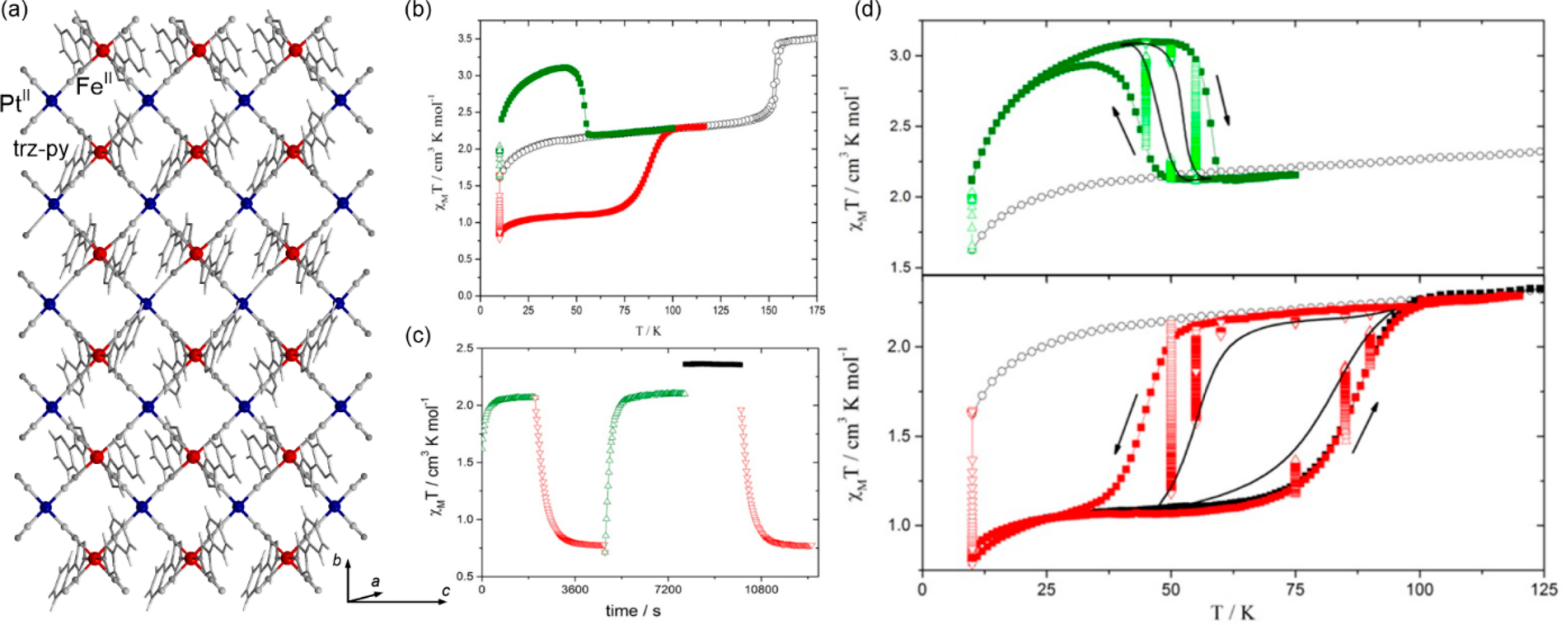
The representative view for the crystal structure of {[Fe^II^(trz-py)_2_][Pt^II^(CN)_4_]}·3H_2_O (trz-py = 4-(2-pyridyl)-1,2,4,4H-triazole) layered coordination framework (a),^477^ its temperature dependence of the *χ*_M_*T* product, presented with the *χ*_M_*T*(*T*) curves for the phases photogenerated at 10 K using 510 nm (green points) and 830 nm (red points) light irradiation (b), the time evolution of the *χ*_M_*T* product at 10 K under successive irradiation with 510 and 830 nm light (c), and the *χ*_M_*T*(*T*) curves measured under 510 nm (top) and 830 nm (bottom) light irradiation, shown with the respective quasi-static hysteresis loops (black lines) (d). Parts (b), (c), and (d) were adapted with permission from ref (477). Copyright 2016 American Chemical Society.

In some spin-crossover systems, due to the presence of antiferroelastic interactions, only one cooperative SCO transition is present while the remaining Fe(II) sites are at the HS state. Such a system can be triggered to undergo light-induced reverse-LIESST. Such behavior was reported by S. Triki and co-workers for the {[Fe^II^(trz-py)_2_][Pt^II^(CN)_4_]}·3H_2_O (trz-py = 4-(2-pyridyl)-1,2,4,4H-triazole) Hofmann-type network (Figure 9).^477^ At cryogenic temperatures, the so-called HS-LS state achieved by sample cooling can be either irradiated to the HS-HS state with 510 nm light or to the LS-LS state with 830 nm wavelength. Under continuous irradiation, both the HS-LS to HS-HS and HS-LS to LS-LS transitions were found to exhibit large cooperativity effects visible in thermal hysteresis loops. The same irradiation wavelengths can also induce the transitions directly between the LS-LS and the HS-HS states below ca. 50 K.^477^

The related behavior was reported for a similar Hofmann-type MOF, {[Fe^II^(3phOH-trz)_2_][Pt^II^(CN)_4_]}·2H_2_O (3phOH-trz = 4-(3-hydroxyphenyl)-1,2,4-triazole), which, within the whole temperature range, remain at the HS state of Fe(II) centers and the reverse-LIESST unveil its hysteretic SCO behavior.^478^ Interestingly, multi-stable Hofmann networks can reveal much more complex behavior as presented for a {[Fe^II^(isoq)_2_][Au^I^(CN)_2_]_2_} (isoq = isoquinoline) layered framework by Z.-P. Ni, M.-L. Tong, and co-workers.^479^ Upon cooling, half of the Fe(II) fraction changes from HS to LS between 150 and 130 K, and the resulting HS-LS state remains stable down to 10 K. Then the 532 nm and 830 nm irradiation induces the formation of the HS-HS and LS-LS configurations, respectively. However, when the photogenerated HS-HS state is heated above *T*_LIESST_ to ca. 80 K, the magnetic moment falls below the level of the HS-LS phase and slowly decays, while the system reaches the LS-LS configuration over time. Then the heating curve shows a two-step SCO that recovers the HS-HS state and initial thermal SCO properties. Such behavior was called Light-Assisted Spin State Annealing (LASSA) mechanism. Moreover, another relaxation pathway to the LS-LS state was unveiled by thermally quenching the system down to ca. 80 K. Despite, that the HS-HS to HS-LS transition occurs instantly, another relaxation is activated and the system goes to the LS-LS state over time. The second mechanism was described as Temperature-Assisted Spin State Annealing (TASSA). Overall, a multi-stable system with different conversion pathways was obtained showing a reversible behavior. This confirms the enormous role of cooperative interactions, crucial to describe the mechanisms of SCO complexes embedded in highly-dimensional coordination networks.^479^

Very lately the hidden hysteretic behavior was also found for Fe(III) SCO systems.^480^ In 2019, P. Harding, D. J. Harding, and co-workers reported SCO behavior of the [Fe^III^(naphEen)_2_]I (naphEen = (1-{[2-(ethylamino)ethylimino]-methyl}-2-naphthol)) assembly that undergoes a cooperative phase transition related to one half on the Fe(III) centers.^480^ This system shows only a small LIESST effect under 650 nm irradiation but the 830 nm or 980 nm light induces a significant reverse-LIESST effect, which allows registering a hidden hysteresis loop under light irradiation. Later, the same group reported the [Fe^III^(qsal-I)_2_]NO_3_·2MeOH (qsal-I = 4-iodo-2-[(8-quinolylimino)methyl]phenolate) system showing bidirectional light switching, accompanied by the change of the SCO behavior upon the crystallization solvent exchange to EtOH.^481^

**Figure 10:**
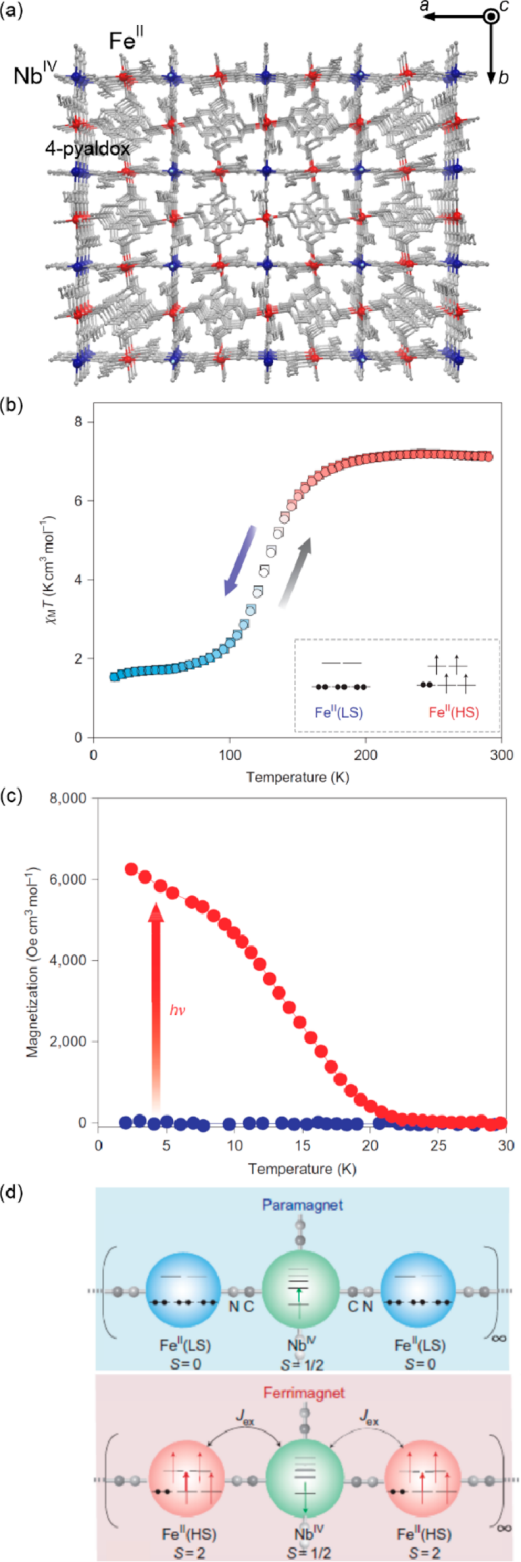
The representative view on {[Fe^II^(4-pyaldox)_4_]_2_[Nb^IV^(CN)_8_]}·2H_2_O (4-pyaldox = 4-pyridinealdoxime) coordination network (a),^111^ its temperature dependence of the *χ*_M_*T* product in the broad temperature range (b) and the limited low-temperature region before and after the 473 nm light irradiation (c), and the schematic illustration of the ferrimagnetic ordering occurring due to the light-induced SCO in this network (d). Parts (b), (c), and (d) were reproduced with permission from ref (111) under terms of the CC-BY license. Copyright 2011 Springer Nature.

While the Hofmann clathrates built of [M^II^(CN)_4_]^2–^ ions usually reveal large cooperativity of thermal SCO effect, the respective networks composed of [M^IV^(CN)_8_]^4–^ ions show rather gradual transitions even when the three-dimensional cyanido-bridged scaffold is formed.^325,482^ However, the combination of Fe^2+^ ions, 4-pyridinealdoxime, and paramagnetic (*S* = 1/2) octacyanidoniobate(IV) moieties generated the {[Fe^II^(4-pyridinealdoxime)_4_]_2_[Nb^IV^(CN)_8_]}·2H_2_O network, showing SCO on the 3/4 of Fe(II) sites (Figure 10).^111^ Therefore, at cryogenic temperatures, this system behaves as a typical paramagnet. Then the 473 nm light irradiation dramatically increases the magnetization, due to the generation of high-spin Fe(II) sites coupling magnetically by cyanido bridges with Nb(IV) centers. Upon irradiation, the appearance of the hysteresis is observed with *T*_c_ around 20 K, but, to recover the pristine state, heating above *T*_LIESST_ is necessary due to the absence of the reverse-LIESST effect. Similar behavior was found in another three-dimensional network, {[Fe^II^(4-methylpyridine)_4_]_2_[Nb^IV^(CN)_8_]}·2H_2_O, upon the 543 nm light irradiation, and the 785 nm light wavelength induces partial reverse-LIESST effect, affecting the size of the hysteresis loop and lowering magnetic ordering temperature from ca. 15 K to ca. 12 K.^483^ In an attempt to achieve a light-induced magnetization, Fe(II) centers were lately combined with diamagnetic [Mo^IV^(CN)_8_]^4–^ and [W^IV^(CN)_8_]^4–^ anions and 4,4′-bipyridine *N*,*N*′-dioxide bridging ligands.^484^ Even though all Fe(II) centers remained at the HS state down to 2 K showing uncoupled paramagnetism, the 450 nm light irradiation induces spin change on the M(IV) centers leading to the combination of ferro- and antiferromagnetic interactions. Another cyanido-bridged network reported by D. Pinkowicz and co-workers, {[Mn^II^(imidazole)(H_2_O)_2_]_2_[W^IV^(CN)_8_]}·4H_2_O lacks the photoinduced magnetic behavior in its hydrated form.^485^ However, after its dehydration a large increase of magnetic signal is observed after irradiation with 450 nm light at 10 K, accompanied by the appearance of a magnetic hysteresis loop even up to 90 K.

**Figure 11:**
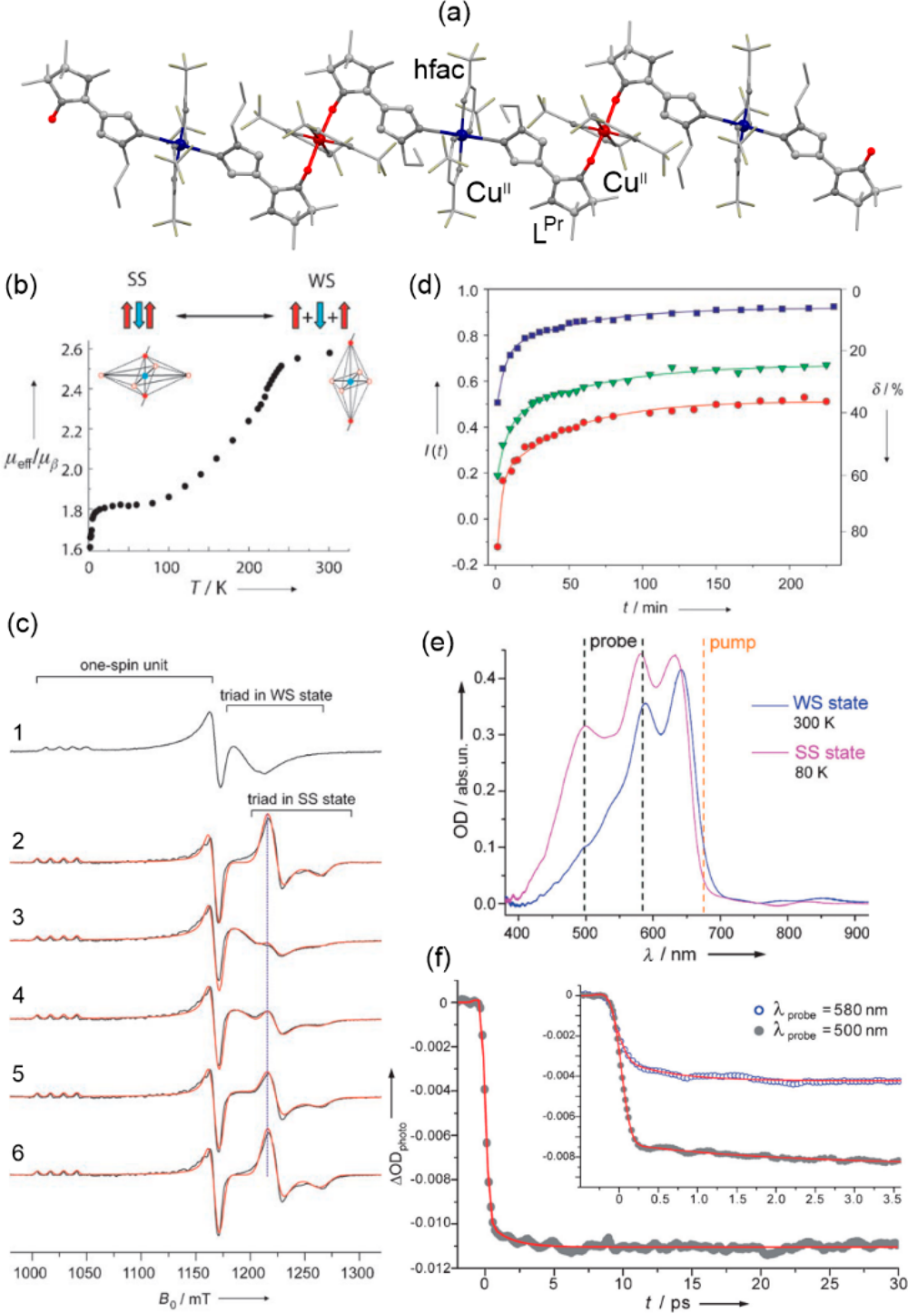
The structure of [Cu^II^(hfac)_2_L^Pr^] (hfac = hexafluoroacetylacetonate; L^Pr^ = propyl-substituted nitronyl nitroxide ligand) coordination chain (a),^487^ the temperature dependence of its effective magnetic moment (b), continuous-wave EPR spectra of this material at 210 K (marked as 1), at 7 K (2), immediately after 900 nm light irradiation at 7 K (3), 5 min (4) and 210 min (5) after irradiation, at 7 K after thermal quenching at 20 K (6) (c), recovery curves of the 1216 mT EPR signal after 900 nm light irradiation at 7 K (red), 10 K (green), and 13 K (blue) (d), optical absorption spectra at 300 and 80 K (e), and time dependence of the transient absorption of this compound in PVC probed at 500 nm after 675 nm pump excitation at 90 K (f). Parts (b), (c), and (d) were adapted with permission from ref (487). Copyright 2008 John Wiley & Sons. Parts (e) and (f) were adapted with permission from ref (489). Copyright 2014 John Wiley & Sons.

A different non-trivial example of a light-induced spin-state switching effect involves the use of exchange-coupled spin triads composed of Cu(II) centers and two nitroxide groups, that appear for the family of the [Cu^II^(hfac)_2_L^R^] (L^R^ = R-substituted nitronyl nitroxide ligand) chain systems.^486^ Some of them are considered breathing crystals due to the thermally induced rearrangements observable in magnetic behavior. At high temperatures, the exchange coupling between the spin centers is small, leading to a weakly coupled spin (WS) state. Upon cooling, the shortening of the distance between spin carriers may appear due to the rotation of the elongated, by the Jahn-Teller effect, axis of the octahedral Cu(II) complex. Such structural feature changes the effective coupling interaction, converting the spin triad to the strongly coupled spin (SS) state at low temperatures. Owing to such behavior, the [Cu^II^(hfac)_2_L^Pr^] (hfac = hexafluoroacetylacetonate; L^Pr^ = propyl-substituted nitronyl nitroxide ligand, Figure 11a–d) system was found to undergo light-induced transformation between SS and WS states upon 900 nm light irradiation at cryogenic temperatures, which was studied by the continuous wave EPR technique on the sample mixed with glycerol.^487^ The light-induced changes and kinetics of this transition were also studied for thin film samples using optical absorption spectroscopy and time-resolved EPR technique (Figure 11e,f).^488,489^ Light-induced SS to WS transition is also observed for the [Cu^II^(hfac)_2_L^i-Pr^] (L^i-Pr^ = isopropyl-substituted nitronyl nitroxide ligand) analog, where the thermal spin transition does not appear. Additionally, the temperature-variable FTIR experiment under photoirradiation revealed different orientations of propyl groups between WS states obtained thermally and by light irradiation, which may influence the relaxation of the photoexcited state.^490,491^ The latest results of the density of states calculations attribute the irradiation band to the LMCT states, and suggest the possibility of light-induced spin switching for all members of the family of Cu(II)-based breathing crystals.^492^

#### 3.1.2 Molecular Nanomagnets Built of Photoresponsive Metal Centers

As discussed in the previous section, the light-induced spin-crossover phenomenon may induce exchange coupling between photogenerated high-spin (HS) metal centers and the accompanying light-insensitive paramagnetic centers, and ultimately, in that way, a long-range magnetic ordering can be obtained.^111^ One may expect that for systems with lower dimensionality, such as coordination clusters or chains, the transition to the HS state may induce the appearance of slow relaxation of the magnetization effect due to magnetic coupling leading to single-molecule magnet (SMM) or single-chain magnet (SCM) behavior. Even for the isolated SCO-active Fe(II) complexes light irradiation and the related *S* = 0 to *S* = 2 transition may induce the SMM behavior as reported by R. Clérac, J. M. Smith, and co-workers for a four-coordinated Fe(II) complex,^493^ and by R. Clérac, J. R. Long, and co-workers, for the octahedral one.^494^

**Figure 12:**
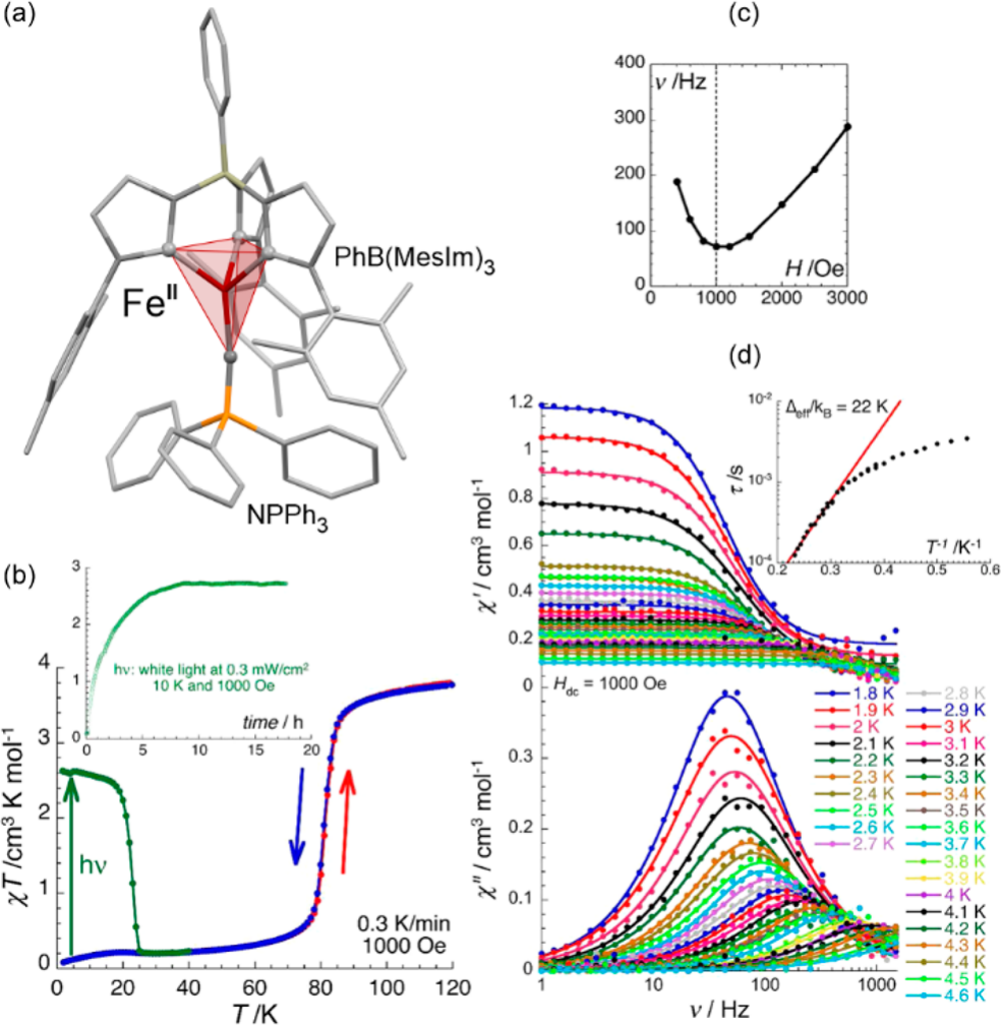
The structure of [PhB(MesIm)_3_Fe^II^(NPPh_3_)] (PhB(MesIm)_3_ = a bulky tris(carbene) borate ligand) metal complex (a),^493^ the temperature dependence of the *χ*_M_*T* product, shown together with the time evolution of the *χ*_M_*T* product during irradiation (the inset) and the *χ*_M_*T*(*T*) curve corresponding to thermal relaxation of the photogenerated state (b), the field dependence of the relaxation time at 2.3 K measured under continuous white light irradiation (c), and the temperature-variable *ac* magnetic characteristics under the 1000 Oe *dc* magnetic field and continuous white light irradiation (d), shown with the temperature dependence of the resulting relaxation time (the inset). Parts (b), (c), and (d) were adapted with permission from ref (493). Copyright 2013 American Chemical Society.

The first example, [PhB(MesIm)_3_Fe^II^(NPPh_3_)] (PhB(MesIm)_3_ = a bulky tris(carbene)borate ligand, Figure 12) system reveals thermal SCO with *T*_1/2_ = 81 K, accompanied by the change of both magnetic moment and optical refractivity.^493^ Upon white light irradiation at 10 K, the *χ*_M_*T* product increases as a consequence of the LIESST effect, which is accompanied by the drop of the absolute refractivity at 970 nm. Increasing the temperature results in relaxation at ca. 23 K, but even at a few kelvins, the photogenerated state decay is significant. Therefore, both field- and temperature-variable relaxation dynamics were studied under continuous low-power white light irradiation.

At zero *dc* field, no relaxation was detected, but a small field of 1 kOe allowed to quench the quantum tunneling of magnetization (QTM) effect. The temperature dependence of the relaxation time for the photoirradiated state revealed the course of the *χ*_M_”(*υ*) maxima which is typical for SMMs, and the related energy barrier for the magnetization reversal extracted using the Arrhenius law reaches 22 K.^493^ For the six-coordinated [Fe^II^(ptz)_6_](BF_4_)_2_ (ptz = 1-propyltetrazole) system, the thermal SCO appears at ca. 130 K.^494^ If the material is cooled slowly, the symmetry of the [Fe^II^(ptz)_6_]^2+^ cations changes from *D*_3h_ to *C*_i_, while upon thermal quenching the higher symmetry is retained for the LS state. Both structural phases show the LIESST effect upon 505 nm light irradiation with a ca. 10 K difference of *T*_LIESST_ temperatures, however, only the excited, higher-symmetry *D*_3h_ phase reveals slow relaxation of magnetization effect with an energy barrier of 15 cm^–1^ under *dc* field of 2 kOe. The observed SMM effect originates from the generation of magnetically anisotropic *S* = 2 Fe(II) complexes under 505 nm light irradiation, and it can be switched off using the 850 nm light wavelength. For the analogous [Fe^II^(mtz)_6_](CF_3_SO_3_)_2_ system with a methyl-substituted tetrazole ligand, only partial thermal SCO above 150 K was reported.^495^ The remaining HS centers reveal SMM behavior, which can be quenched by the reverse-LIESST effect using the 650–900 nm light, and then recovered within the LIESST effect by 500–650 nm light irradiation.

**Figure 13:**
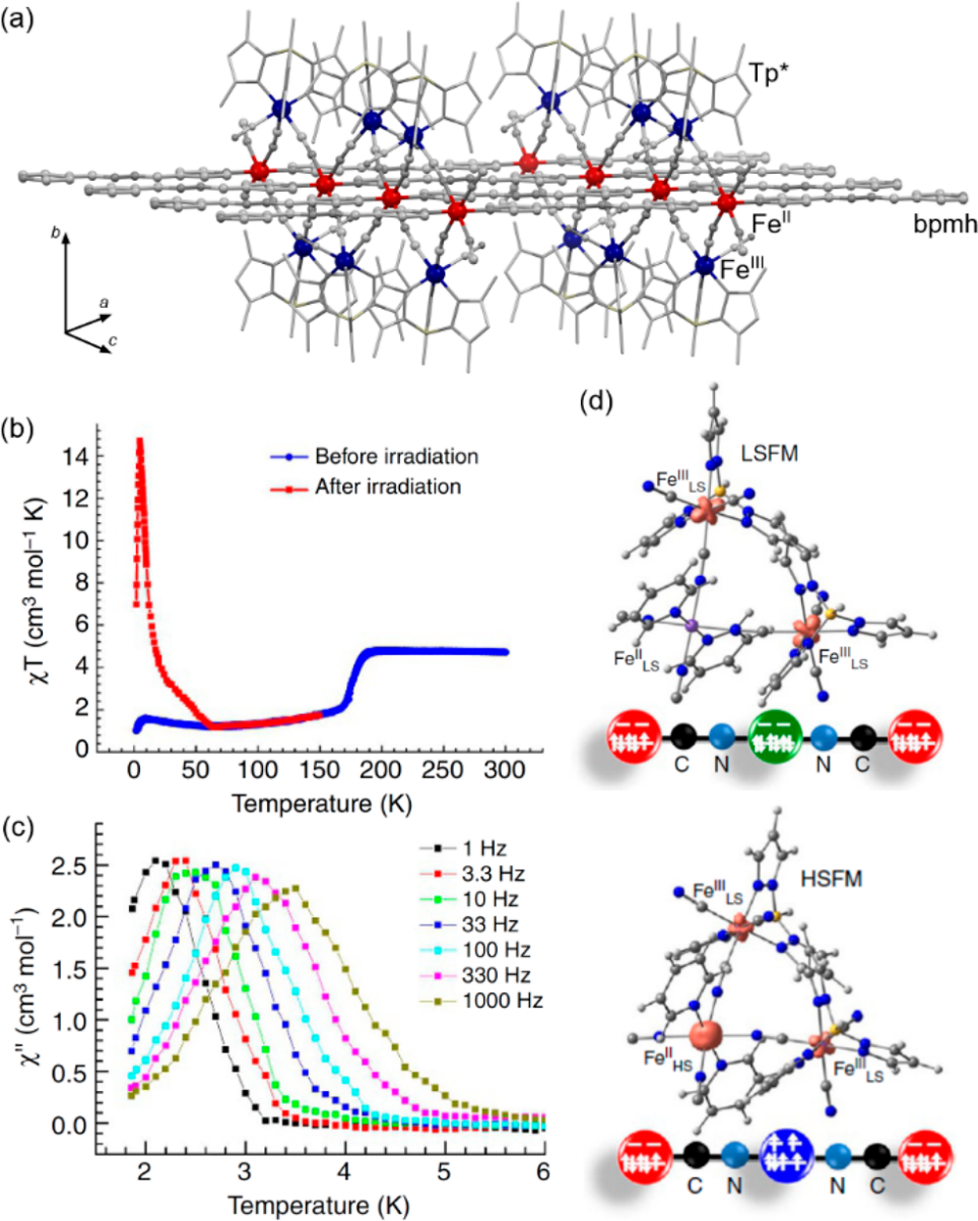
The structural view on {[Fe^II^(bpmh)]_2_[Fe^III^(Tp*)(CN)_3_]}·2H_2_O (bpmh = *N*,*N*′-bis-pyridin-4-ylmethylene hydrazine; Tp* = hydrotris(3,5-dimethylpyrazolyl)borate) layers (a),^496^ the temperature dependence of the *χ*_M_*T* product before and after the 473 nm light irradiation at 5 K (b), the temperature dependences of the *ac* magnetic susceptibility after irradiation (c), and the schematic representation of the ground ferromagnetic triplet state (LSFM) and the excited ferromagnetic septet state (HSFM) (d). Parts (b), (c), and (d) were reproduced with permission from ref (496) under terms of the CC-BY license. Copyright 2013 Springer Nature.

Through combining the iron(II) centers with *N*,*N*′-bis-pyridin-4-ylmethylene hydrazine (bpmh) and paramagnetic low-spin [Fe^III^Tp*(CN)_3_]^2–^ (Tp* = hydrotris(3,5-dimethylpyrazolyl)borate) complexes, a layered coordination system, {[Fe^II^(bpmh)]_2_[Fe^III^Tp*(CN)_3_]}·2H_2_O, showing the SCO behavior was obtained (Figure 13).^496^ Mössbauer spectra analysis revealed a complete HS to LS spin transition down to 5 K for Fe(II) centers. At this temperature, the 473 nm light irradiation results in a strong increase of magnetization due to the LIESST effect. The *χ*_M_*T* product plots before and after irradiation bifurcate at ca. 62 K, and a maximum of the *χ*_M_*T* product is present after irradiation at 4.6 K, suggesting the presence of exchange interactions between light-induced HS Fe(II) centers and paramagnetic (*S* = 1/2) LS Fe(III) complexes, within the chains. Magnetization versus field course after irradiation suggested ferromagnetic exchange between HS Fe(II) and LS Fe(III) complexes bridged by cyanido ligands, resulting in a single-chain magnet (SCM) behavior. The observed *ac* relaxation dynamics at zero *dc* field were used to elucidate the energy barrier of 43 K. The analysis of the *ac* data disclaimed the presence of the spin-glass behavior, as well as further coupling between the separated chains by the organic ligands. DFT studies supported the analysis of the exchange interactions that are ferromagnetic both in the weakly coupled triplet state given by two LS Fe(III) sites and after irradiation within strongly coupled septet state involving two LS Fe(III) complexes mediated by HS Fe(II) sites. Despite the presence of the SCM behavior after irradiation, no magnetic bistability in the form of magnetization versus field hysteresis loop was reported.^496^

To enhance the SCM behavior after irradiation, a similar system was built of Fe(II) centers, [Fe^III^Tp^Pz^(CN)_3_]^2–^ (Tp^Pz^ = tetrakis(pyrazolyl)borate) moieties, and asymmetric organic ligands, 4-pyridine-4-ylmethyleneamino-1,2,4-triazole (Pmat).^497^ The resulting water-solvated system, {[Fe^II^(Pmat)]_2_[Fe^III^Tp^Pz^(CN)_3_]}·12H_2_O contains Fe(II) centers with a distorted coordination environment of four N-bonded cyanido ligands, one triazole N atom, and one pyridine N atom, while the {[Fe^II^(Pmat)]_2_[Fe^III^Tp^Pz^(CN)_3_]}·4MeOH solvate binds either two triazole or two pyridine ends of the ligands, and can be easily desolvated to the {[Fe^II^(Pmat)]_2_[Fe^III^Tp^Pz^(CN)_3_]} phase. Both solvates reveal a complete gradual SCO on the Fe(II) sites, while for the desolvated phase only half of the *S =* 2 centers reach the LS state. Upon irradiation of the water solvate with 808 nm light, a wide magnetic hysteresis appears at 2 K of the intrachain origin, i.e., the SCM behavior. The desolvated phase also shows the LIESST effect on LS Fe(II) sites but the photo-generated phase shows only the SCM behavior visible in the *ac* magnetic data.^497^

**Figure 14:**
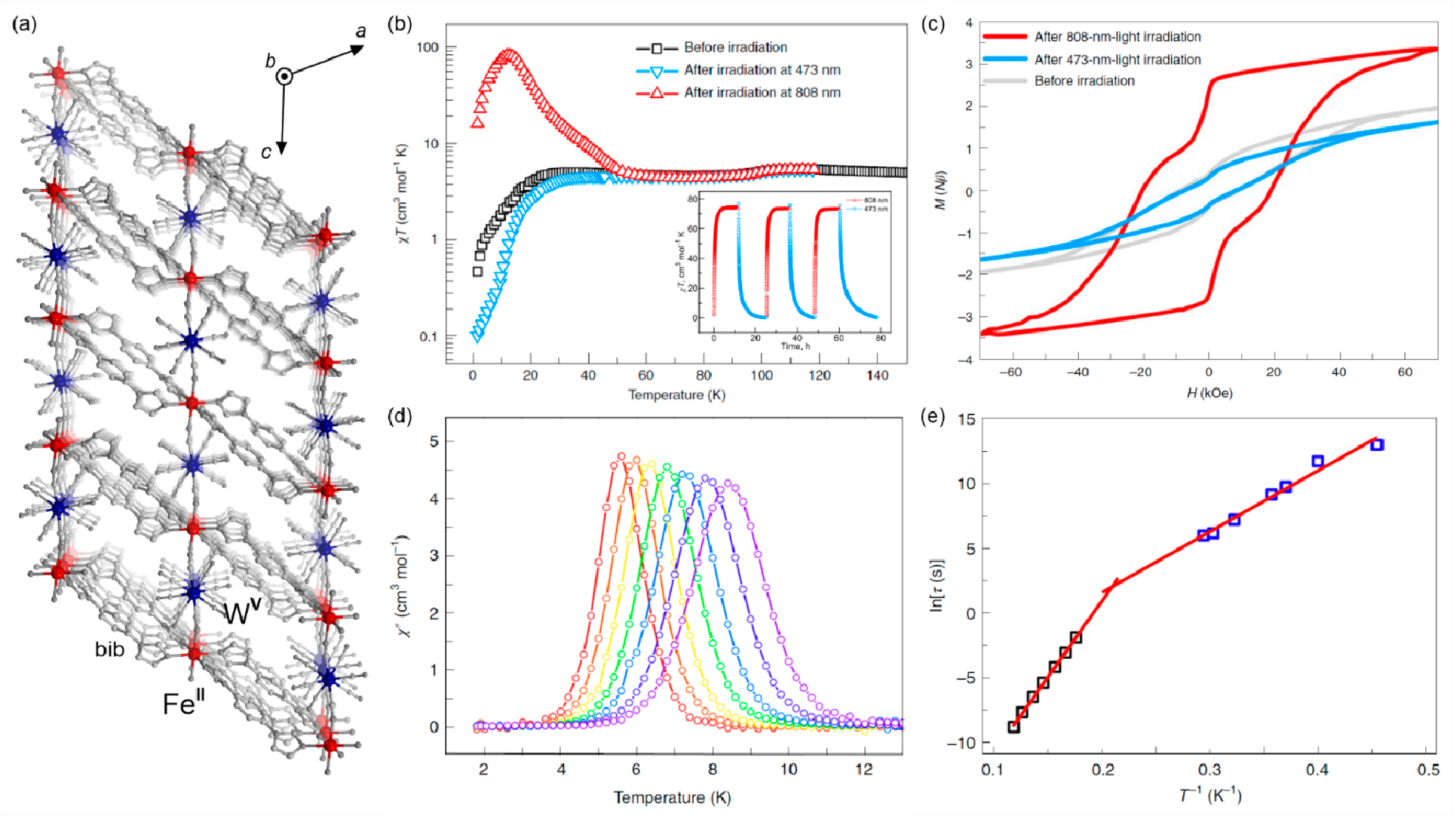
The representative view on (bibH){[Fe^II^(bib)_2_][W^V^(CN)_8_]}·MeOH (bib = 1,4-bis(1H-imidazol-1-yl)benzene) layered framework (a),^498^ the temperature dependence of the *χ*_M_*T* product before and after the 473 or 808 nm light irradiation at 10 K (b), shown together with isothermal cycles of sequential light irradiation (the inset), the field-dependent magnetization curves before and after the 473 or 808 nm light irradiation recorded at 1.8 K (c), the temperature dependences of the imaginary parts of the *ac* susceptibility under zero *dc* field after irradiation with the 808 nm light (d) and the temperature dependence of the resulting magnetic relaxation time after irradiation with the 808 nm light (e). Parts (b), (c), (d), and (e) were reproduced with permission from ref (498) under terms of the CC-BY license. Copyright 2021 Springer Nature.

For none of the coordination systems bridged by [Fe^III^Tp^Pz^(CN)_3_]^2–^ metalloligands, a light deactivation pathway of SCM behavior through the reverse-LIESST was presented. However, bidirectional photoswitching behavior was presented for another cyanido-bridged framework but constructed of paramagnetic [W^V^(CN)_8_]^3–^ anions.^498^ In 2021, Y.-S. Meng, T. Liu, and co-workers reported the synthesis of (bibH){[Fe^II^(bib)_2_][W^V^(CN)_8_]}·MeOH (bib = 1,4-bis(1H-imidazol-1-yl)benzene) heterometallic assembly, where the bib molecules serve as molecular linkers between cyanido-bridged Fe(II)–W(V) chains, as well as additional cations in their mono-protonated form (Figure 14).^498^ The course of the temperature dependence of the *χ*_M_*T* product reveals only a partial thermal SCO effect of the Fe(II) centers of ca. 28%, as found from ^57^Fe Mössbauer spectra. At the lowest temperatures, the value of the *χ*_M_*T* product drops significantly, which was attributed to the cumulative effect of single-ion anisotropy of the remaining HS Fe(II) centers and the appearance of antiferromagnetic interactions between cyanido-bridged chains. At 1.8 K, the pristine system shows a small magnetic hysteresis loop with a remnant magnetization of 0.39 Nβ and a coercive field of 9 kOe. Upon 808 nm light irradiation at 10 K, the *χ*_M_*T* product increases due to the LIESST effect of LS Fe(II) centers, while the hysteresis loop at 1.8 K reaches 19 kOe of coercive field and 2.6 Nβ value of remnant magnetization. When the pristine system is treated with 473 nm light, the reverse-LIESST is observed for the part of the thermally non-switchable HS Fe(II) centers. The same light wavelength induces the reverse-LIESST for the photogenerated HS Fe(II) fraction. Such behavior allows one to switch the magnetization both ways by alternating light irradiation. The *ac* magnetic studies were performed before and after irradiation to study the relaxation behavior. For the pristine system, the extracted energy barrier is 96(3) K, but the values of correlation length of 1.5 and the effective Curie constant of 3.5(1) cm^3^mol^–1^K suggest the SMM behavior. After irradiation, the energy barriers can be distinguished into 117(1) K for the infinite-size region, and 47(3) K for the finite-size region. The correlation length of 55.7 after irradiation confirms the SCM behavior. At 3.3 K, this system irradiated with 473 nm light lacks magnetic hysteretic behavior while after 808 nm irradiation hysteresis remains present at this temperature. This enables to completely switch of the magnetic memory effect related to the SCM behavior. Molecular squares built of SCO Fe(II) complexes and [W^V^(CN)_8_]^3–^ anions were also presented, showing the LIESST effect.^499^ However, a possible SMM behavior was not found presumably due to the low photoconversion and/or low anisotropy of the photogenerated system.

**Figure 15:**
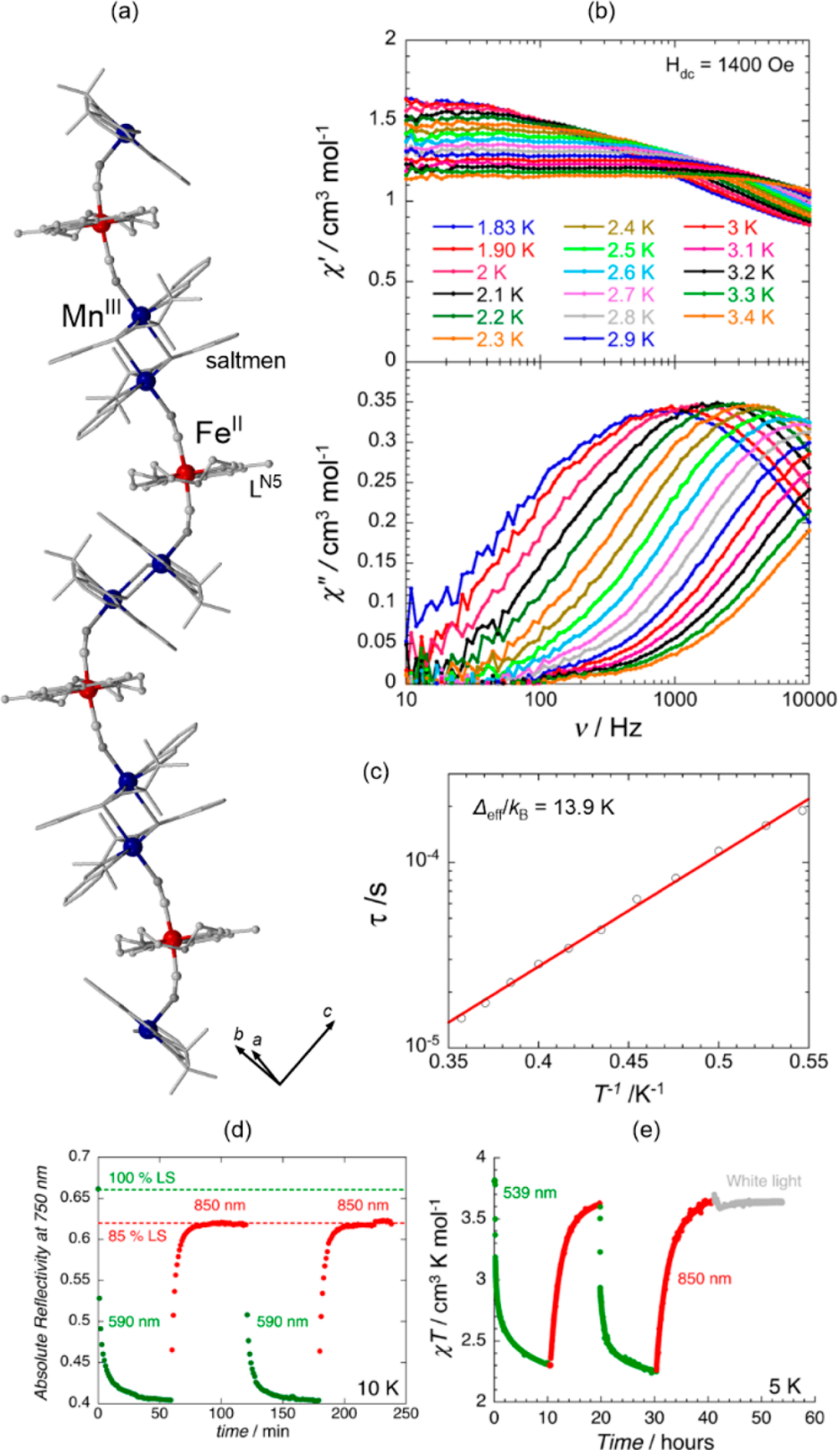
The structure of {[Mn^III^(saltmen)]_2_[Fe^II^(L^N5^)(CN)_2_]}(ClO_4_)_2_·0.5C_4_H_10_O·0.5H_2_O (saltmen = *N*,*N*′-(1,1,2,2-tetramethylethylene)bis(salicylideneiminate); L^N5^ = 2,13-dimethyl-3,6,9-12,18-pentaazabicyclo[12.3.1]octadeca-1(18),2,12,14,16-pentaene) coordination chain (a),^500^ the temperature-variable frequency dependencies of the *ac* magnetic susceptibility (b) and the related temperature dependence of the relaxation time before the photoirradiation experiment (c), the isothermal cycles of sequential light irradiation at 10 K followed by the absolute refractivity at 750 nm (d), and the isothermal cycles of sequential light irradiation at 5 K followed within photomagnetic experiment tracing the value of the *χ*_M_*T* product (e). Parts (b), (c), (d), and (e) were adapted with permission from ref (500). Copyright 2013 American Chemical Society.

A completely different influence of the light-induced SCO upon slow relaxation of the magnetization properties was observed for a system built of {Mn_^III^2_} SMMs linked to Fe(II) complexes, {[Mn^III^(saltmen)]_2_[Fe^II^(L^N5^)(CN)_2_]}(ClO_4_)_2_·*n*solv (saltmen = *N*,*N*′-(1,1,2,2-tetramethylethylene)bis(salicylideneiminate); L^N5^ = 2,13-dimethyl-3,6,9-12,18-pentaazabicyclo[12.3.1]octadeca-1(18),2,12,14,16-pentaene, Figure 15).^500^ For this compound, the [Fe^II^(L^N5^)(CN)_2_] moiety remains in its LS Fe(II) state within the whole temperature range below 300 K. Down to 1.8 K, the *χ*_M_*T*(*T*) curve can be described by contributions from dinuclear magnetic {Mn^III^_2_} units well separated by the Fe(II) diamagnetic complexes within the chain. The dynamic (*ac*) magnetic studies revealed slow relaxation of the magnetization effect of a molecular origin upon the application of an external *dc* field. The energy barrier for the Orbach relaxation extracted from the temperature dependence of the relaxation time under *H*_dc_ = 1400 Oe reaches only 13.9 K but is typical for the isolated binuclear Mn(III)-based SMMs. As the [Fe^II^(L^N5^)(CN)_2_], despite the lack of thermal SCO, exhibits the LIESST effect accompanied by the change of the coordination number, the chain system was irradiated with white light at 10 K and a change of the absolute refractivity at 750 nm was recorded. Upon alternating irradiation with 590 nm and 850 nm light, the changes of the refractivity were found reversible after the first step, as the reverse-LIESST was found only accessible for 85% of photogenerated HS centers. Photomagnetic measurements using the 539 nm light revealed the decrease of the *χ*_M_*T* product after irradiation at 5 K, resulting from the generation of *S* = 2 Fe(II) HS centers coupling antiferromagnetically with {Mn^III^_2_} units. The thermal relaxation of photoinduced centers occurs at 54 K, but the 830 nm light at 5 K can also induce an increase of the *χ*_M_*T* product, thus confirming the results of refractivity measurements. Using photomagnetic studies similar efficiency of the reverse-LIESST effect was observed; however, alternating light irradiation again proved two-way switching only after the first step.^500^ Light-induced transformation to the HS state of 51% of the Fe(II) centers was found sufficient to turn off the SMM behavior, which stays in contrast to the results for the above-mentioned (bibH){[Fe^II^(bib)_2_][W^V^(CN)_8_]}·MeOH framework.^498^

**Figure 16:**
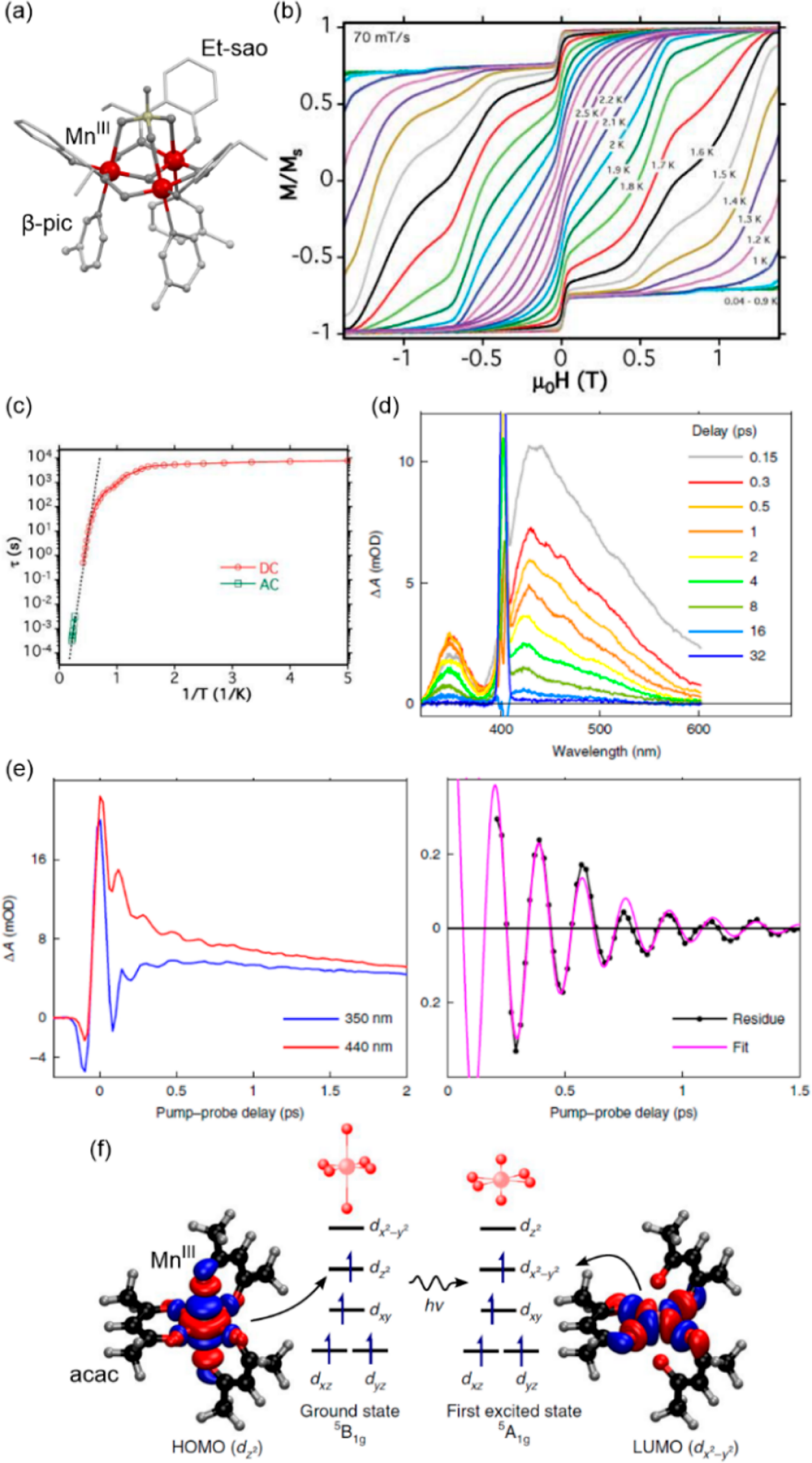
The structure of {[Mn^III^_3_O(Et-sao)_3_(β-pic)_3_(ClO_4_)]} (Et-saoH_2_ = ethyl derivative of salicylaldoxime; β-pic = 2-picolylamine) molecular triangle (a),^502^ the series of its field-dependent magnetization curves at the indicated temperatures (b), the related temperature dependence of the relaxation time (c), the difference spectra at the magic angle, for selected time delays, obtained from the transient absorption data in EtOH after pumping at 400 nm (d), and the kinetic traces for UV and vis bands (left) and associated averaged residues of the global analysis (435–492 nm, right) (e), all shown together with the scheme of the molecular and electronic structure of {[Mn^III^(acac)_3_]} (acac^–^ = acetylacetonate anion) complex for its ground and first excited states, presented for comparison (f). Parts (b) and (c) were reproduced from ref (467) with permission from the Royal Society of Chemistry. Parts (d), (e), and (f) were reproduced with permission from ref (501) under terms of the CC-BY license. Copyright 2020 Springer Nature.

Another method of switching the magnetic state of SMMs was proposed by J. O. Johansson and co-workers using femtosecond laser pulses.^501^ To test such an idea, the dynamics of the triangular {Mn^III^_3_} SMM were studied using ultrafast transient absorption spectroscopy. The magnetic anisotropy of Mn(III) centers is related to the Jahn-Teller (JT) distortion for the *t*_2*g*_^3^*e*_*g*_^3^ electronic configuration.^502^ Femtosecond laser pulses can modulate the JT axis; therefore, in the case of cluster-based Mn(III) systems, one may expect to achieve optical control of magnetic anisotropy and the related SMM behavior by ultrafast photoswitching. The selected {Mn^III^_3_} SMM, {[Mn^III^_3_O(Et-sao)_3_(β-pic)_3_(ClO_4_)]} (Et-saoH_2_ = ethyl derivative of salicylaldoxime; β-pic = 2-picolylamine, Figure 16), is composed of three Mn(III) centers that are arranged in a triangle by μ_3_-oxo bridge and three oxime linkages. The axis of JT distortion for each Mn(III) center goes perpendicular to the triangular plane. For comparison, a simpler [Mn^III^(acac)_3_] (acac^–^ = acetylacetonate anion) complex, was also selected, where the ground ^5^B_1g_ state with elongated JT axis and excited one, ^5^A_2g_, with compressed JT axis may be easily assigned. While the [Mn^III^(acac)_3_] complex does not show significant anisotropy, the {Mn^III^_3_} triangle was reported by E. Brechin and co-workers to reveal a magnetic hysteresis up to 2.5 K.^502^ Both systems were studied in ethanol solutions at room temperature under 400 nm laser pulses, where the excitation involves ligand-field transitions. For [Mn^III^(acac)_3_], four constants were used in a decay model to fit the experimental data of 0.25, 1.0, 5.2, and 400 ps, while, for {Mn^III^_3_}, their number was reduced to three, 0.18, 1.9, and 9 ps. The kinetic traces were analyzed but the extracted oscillator frequencies gave little information about the relaxation mechanisms of the excited states due to the system complexity. However, the analysis of the vibrational wavepacket compared with the Raman spectra revealed, that for [Mn^III^(acac)_3_] change of the JT distortion is observed from axial to equatorial. For the {Mn^III^_3_} triangle, the strong equatorial bonds prevent the switching of the JT distortion, thus forcing the wavepacket motion along the JT axis, resulting in a simpler reaction coordinate and reducing the lifetime of the excited state to 9 ps.^501^

Other mononuclear magnetic Mn(III) species were also recently investigated by transient absorption spectroscopy. For the [Mn^III^(cyclam)(H_2_O)_2_]^3+^ (cyclam = 1,4,8,11-tetraazacyclotetradecane) system, the equatorial organic ligand restricts the expansion within the plane after pulse photoexcitation, shortening the lifetime of the excited, axially compressed state compared to [Mn^III^(acac)_3_] complex.^503^ Moreover, the family of [Mn^III^(terpy)X_3_] (terpy = 2,2′:6′,2′′-terpyridine; X = F, Cl, N_3_) complexes showing the switching effect of JT axis was found to reveal longer dephasing times than [Mn^III^(acac)_3_] complex, which was correlated with the limited number of low-frequency vibrational modes.^504^

**Figure 17:**
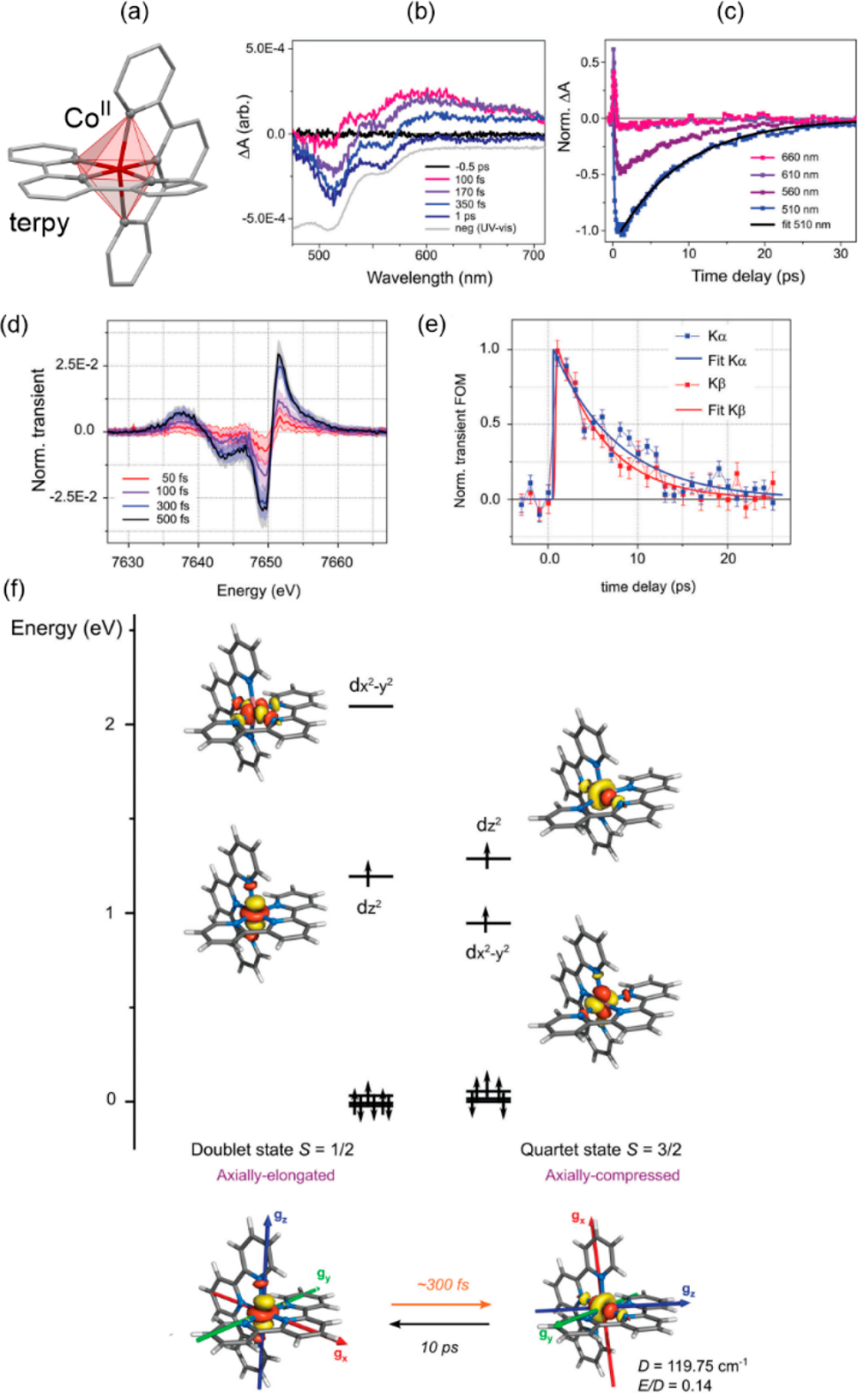
The structure of [Co^II^(terpy)_2_]^2+^ (terpy = 2,2';6',2"-terpyridine) metal complex (a),^505^ its transient optical absorption spectra in water excited at 400 nm, shown for selected time delays (b), and their normalized kinetics at different wavelengths (c), the related normalized K*β* transient difference profiles of X-ray emission observed for different pump-probe delays after optical excitation at 400 nm (d), K*α*1 and K*β* kinetics tracking the lifetime of the quartet HS state (e), and the calculated SA-CASSCF orbitals for the doublet and quartet structures (f). Parts (b), (c), (d), (e), and (f) were adapted with permission from ref (505). Copyright 2023 John Wiley & Sons.

Apart from Mn(III) complexes, lately the Co(II)-based molecular nanomagnet, [Co^II^(terpy)_2_]^2+^, was studied from the point of ultrafast photoswitching in solution (Figure 17).^505^ Transient absorption spectra in water were collected under 400 nm femtosecond pulses and the relative absorbance kinetics were followed at four selected wavelengths of the visible region, showing a single-exponential decay for the lowest-excited metastable state with a lifetime of 6.4(4) ps. The [Co^II^(terpy)_2_]^2+^ system was also investigated by femtosecond X-ray emission spectroscopy in water.

The photoemission at K*α* and K*β* lines was followed after 400 nm optical excitation for different pump-probe delays in a sub-ps timescale and show that the excitation induces a spin transition from the LS (*S =* 1/2) doublet state to the metastable HS (*S* = 3/2) state. The K*α* and K*β* lines decay with time-constants of 7.5(1.3) ps and 5.4(6) ps, respectively, which agrees with the results of transient absorption spectra analysis. The *ab initio* calculations indicated the transition from the doublet ground state to the excited quartet state as accompanied by the geometrical change from the tetragonally elongated octahedron to the tetragonally compressed one, which reorients the axes of the *g* tensor, thus upon light irradiation magnetic anisotropy is switched. Such ultrafast switching behavior is stated as general for Co(II) complexes.^505^ For the Fe(II)-analog, the [Fe^II^(terpy)_2_]^2+^ complex with a *D*_2h_ distorted LS ground state achieves the HS manifold after pulse excitation in solution, with a reduced *D*_2_ symmetry due to the JT effect.^506^

### 3.2 Optical Switching Related to Electron Transfer Processes

For single spin centers, the effect upon photoirradiation usually persists at relatively low temperatures due to the fast relaxation pathway for the metastable state, when numerous phonon modes are thermally activated. To some extent, as discussed in the previous section, the energy barrier for the related transformation from the metastable state to the ground one can be improved by increasing structural reorganization after photoexcitation. Another method involves systems in which after light irradiation the absorbed energy is utilized for the electron transfer process to occur between redox-active chemical moieties instead of the spin transition on a single center.^507^ Such phenomenon, called charge transfer transition, may appear between different atoms or groups within a single organic entity,^508^ metal center and ligand or its part,^509^ as well as between redox-active metal complexes forming heterometallic coordination or supramolecular assemblies.^351,510^ High-temperature photoswitching based on electron transfer may be employed for photomagnetic devices, but it is also explored for light harvesting,^511^ switchable catalytic materials or electronic devices,^512−516^ and photochromic luminophores.^517^ Among ligand-based electron transfer systems, some attention is devoted to viologen-based coordination polymers and molecules, which reveal photoinduced optical changes upon light irradiation at room temperature.^515−520^ Another class of expanded systems employs the redox activity of metallofullerenes.^513,521^ Next, lanthanide(III)-hexacyanidoferrate(III) systems in some cases reveal photochromism accompanied by magnetic moment changes due to presumable photoinduced ligand-to-metal charge transfer (LMCT),^522−525^ appearing even at room temperature.^526,527^ Although similar cyanido complexes, such as nitroprusside anion, [Fe^II^(CN)_5_(NO)]^2–^, were initially thought to reveal photoinduced MLCT,^528,529^ pentacyanidonitrosylmetallates (among them, a nitroprusside ion) are now recognized for the mechanism of photoisomerization of nitrosyl ligands, which leads toward modification of structural, optical, or even electrical features.^113,116,530,531^ In addition, for the family of compounds built of copper(II) and octacyanidometallates(IV), a mechanism of photoinduced Cu(II)-to-Mo(IV)/W(IV) electron transfer was proposed.^532−536^ As described in the previous section, light irradiation for the latter cyanido complexes can induce photodissociation, nevertheless, for some Cu(II)-to-Mo(IV) systems the electron transfer mechanism is thoroughly proven by X-ray absorption spectroscopy techniques.^122,536−540^ In general, a considerable number of binuclear coordination systems reveal electron transfer property; although, in only some of them, a switching pathway involves light stimulus.^50^ In this context, the most recognizable effort was devoted toward Co(II/III)-Fe(III/II) systems, where the appearance of electron transfer is accompanied by the change between low and high spin configuration of the Co center, as such results in drastic transition between diamagnetic and paramagnetic (or even magnetically coupled) phases (see also section 2.2. for comparison).^50,351,500^ In the next two sections, we will discuss the representatives of such photomagnetic phenomena obtained within less than the last three decades.

#### 3.2.1 Spin Transition Materials Exploring Photoswitchable Electron Transfer

**Figure 18:**
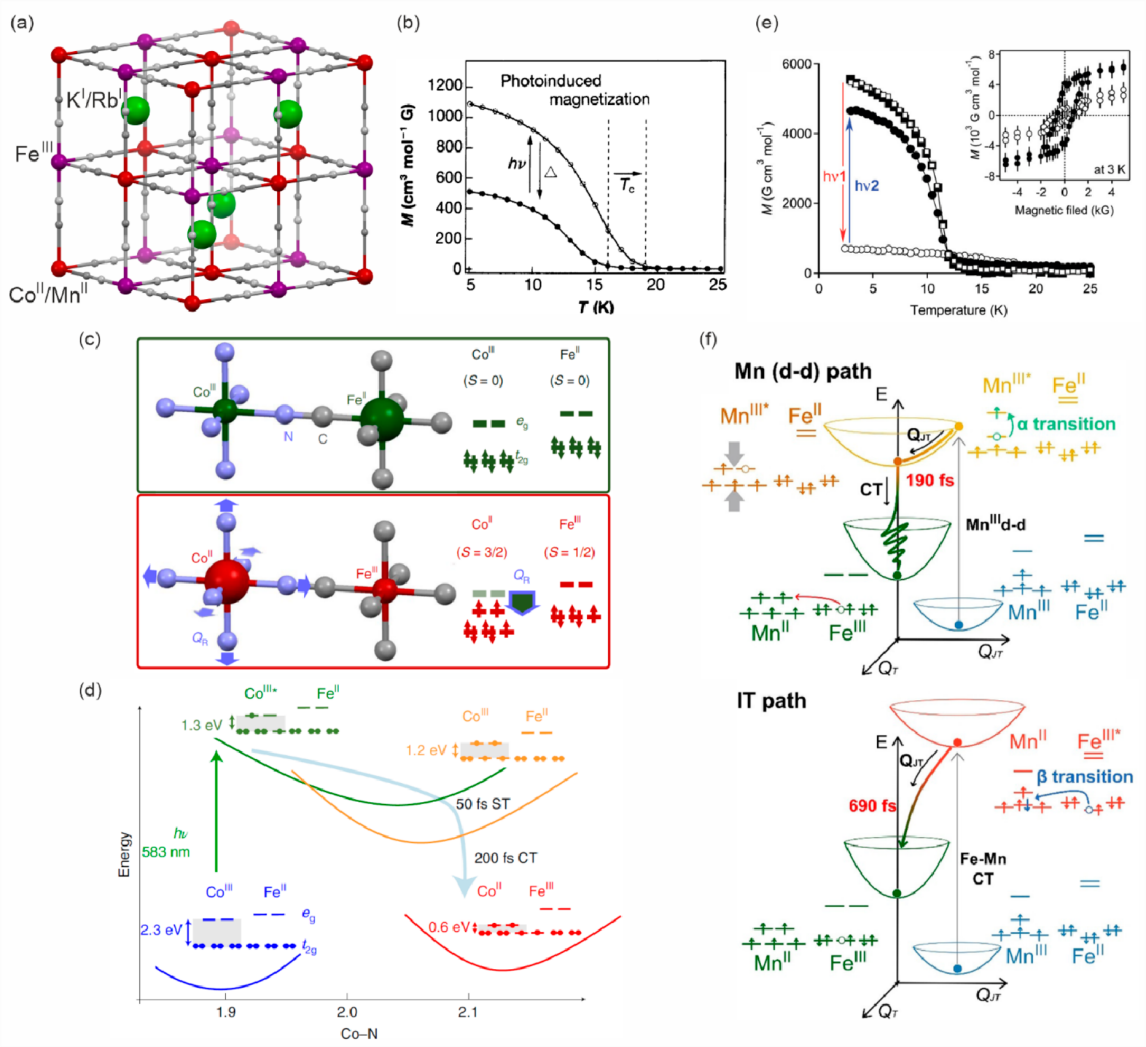
The view on the idealized crystal structure of Prussian blue analogs (PBAs) of the A^I^_*x*_{M^II^_*y*_[Fe^III^(CN)_6_]}·*n*H_2_O (A = alkali metal ion, M = d-block metal ions; here on the examples of A = K and M = Co, as well as A = Rb and M = Mn) type, shown without the visualization of the defects related to the presence of water molecules instead of the part of hexacyanidometallate complexes (a),^541^ the magnetization versus temperature curves before and after red light irradiation for K^I^_0.2_{Co^II^_1.4_[Fe^III^(CN)_6_]}·6.9H_2_O PBA (CoFe PBA) network (b), the molecular unit of the CoFe PBA with the graphical representation of the accessible spin states (c), schematic diagram of the potential energy curves of different key states involved in the electron transfer process in CoFe PBA (d), the magnetization versus temperature curves for Rb_0.88_{Mn^II^[Fe^III^(CN)_6_]_0.96_}·0.5H_2_O PBA (RbMnFe PBA) network, gathered in the dark, after irradiation with the 532 nm light at 3 K, after the reverse photomagnetic effect with the 410 nm light at 3 K, and after thermal relaxation at 180 K, all shown together with the respective magnetic hysteresis loops at 3 K after irradiation with both wavelengths (e), and the schematic representation of the potential energy curves of different key states involved in the electron transfer process within the Mn (d–d excitation) path and the IT (intervalence transfer) path of RbMnFe PBA (f). Part (b) was reproduced with permission from ref (233). Copyright 1996 American Association for the Advancement of Science. Parts (c) and (d) were reproduced with permission from ref (550) under terms of the CC-BY license. Copyright 2021 Springer Nature. Part (e) was adapted with permission from ref (369). Copyright 2008 American Chemical Society. Part (f) was adapted with permission from ref (562). Copyright 2021 John Wiley & Sons.

In the context of the efficient photoswitchable electron transfer phenomenon including its various aspects up to ultrafast pulse photoswitching, it is worth focusing initially on the family of charge transfer systems built of Prussian blue analogs (PBAs) (Figure 18a). At the end of the 20^th^ century, A. Fujishima, K. Hashimoto, and co-workers reported the pioneering photoswitching of magnetization in K^I^_0.2_{Co^II^_1.4_[Fe^III^(CN)_6_]}·6.9H_2_O (CoFe) cubic PBA upon irradiation with red light at 5 K (Figure 18b).^233,541^ The effect of the increased magnetization persisted for several days at 5 K and resulted in increased critical temperature of magnetic ordering from 16 K to 19 K. To restore the initial state, the temperature was raised to 150 K, or the system was irradiated by blue light at 5 K. The explanation of the photomagnetism was then given by IR spectroscopy suggesting a photoinduced change both in valence and spin states for Co and Fe centers. Before irradiation, both LS Co^III^ (*S* = 0) and LS Fe^II^ (*S* = 0) sites, as well as HS Co^II^ (*S* = 3/2) and LS Fe^III^ (*S* = 1/2) are observed (Figure 18c). After red light treatment, the fraction of the latter centers is increased, thus the electron transfer process must have occurred, accompanied by the spin transition on Co sites. After this report, several studies were devoted to the modification of such PBAs towards improved magnetic and photomagnetic characteristics, as well as to the construction of lower dimensionality systems that are built of Co and Fe sites showing the analogous effect.^542−549^ For years, the mechanism of so-called charge transfer-induced spin transition (CTIST), or electron transfer-coupled spin transition (ETCST), was discussed, but only in 2021, M. Cammarata, E. Collet, and co-workers gave insight into the occurring photodynamics using X-ray and optical absorption spectra collected at the femtosecond resolution (Figure 18c,d).^550^ For this purpose, nanocrystals of the cesium-containing analog of the CoFe PBA were used, which exhibit a high relaxation temperature of the photoinduced state of ca. 120 K.^551^ After a combined analysis of XANES spectral changes at the Co and Fe sites K-edges 3 ps after 540 nm photoexcitation and optical density changes for different probe wavelengths, the fast process of 50(10) fs was found solely at the Co site. With the support of TD-DFT calculations, it was ascribed to photoinduced spin transition on the Co site towards the HS Co^III^ (*S* = 2) state, which further drives the charge transfer between metallic centers within 200(10) fs.

Another system from the family of PBAs, Rb^I^_0.88_{Mn^II^[Fe^III^(CN)_6_]_0.96_}·0.5H_2_O (RbMnFe) also reveals a charge transfer transition between magnetic centers, which can be induced and reversibly switched by light at cryogenic temperatures (Figure 18e).^369,552−561^ The low-temperature phase of this system comprises HS Mn^III^ (*S* = 2) and LS Fe^II^ (*S* = 0) centers. The presence of magnetic exchange between Mn^III^ centers results in ferromagnetic ordering below 12 K. After irradiation with 532 nm light, the phase with HS Mn^II^ (*S* = 5/2) and LS Fe^III^ (*S* = 1/2) sites is obtained but it orders antiferromagnetically. Then the 410 nm light irradiation recovers the initial low-temperature state almost quantitively. The charge transfer process for the RbMnFe PBA was also studied using femtosecond light pulses (Figure 18f).^562^ For that purpose, two excitation wavelengths were selected, 445 nm and 580 nm, corresponding to the intervalence (IT) charge transfer and Mn^III^-centered d–d absorption bands, respectively, while time evolution of the optical density charge was collected at 640 nm. The performed analysis revealed two separate processes depending on the excitation light. Upon irradiation of d–d bands, the excitation drives reverse Jahn-Teller distortion, which is followed by charge transfer within 190 fs. The longer process involves the higher energy IT band where the light-induced charge transfer is followed by a spin transition within 690 fs. Photoinduced ETCST was also reported for the {Co^II^_3_[Os^III^(CN)_6_]_2_}·6H_2_O PBA by K. R. Dunbar and co-workers but no related ultrafast excitation studies of the mechanism were presented up to now.^367^ Moreover, for the some of mixed-metal PBAs, the effect of light-induced magnetic pole inversion was reported, as well as such effect was found to occur upon the application of the external pressure.^563−568^

**Figure 19:**
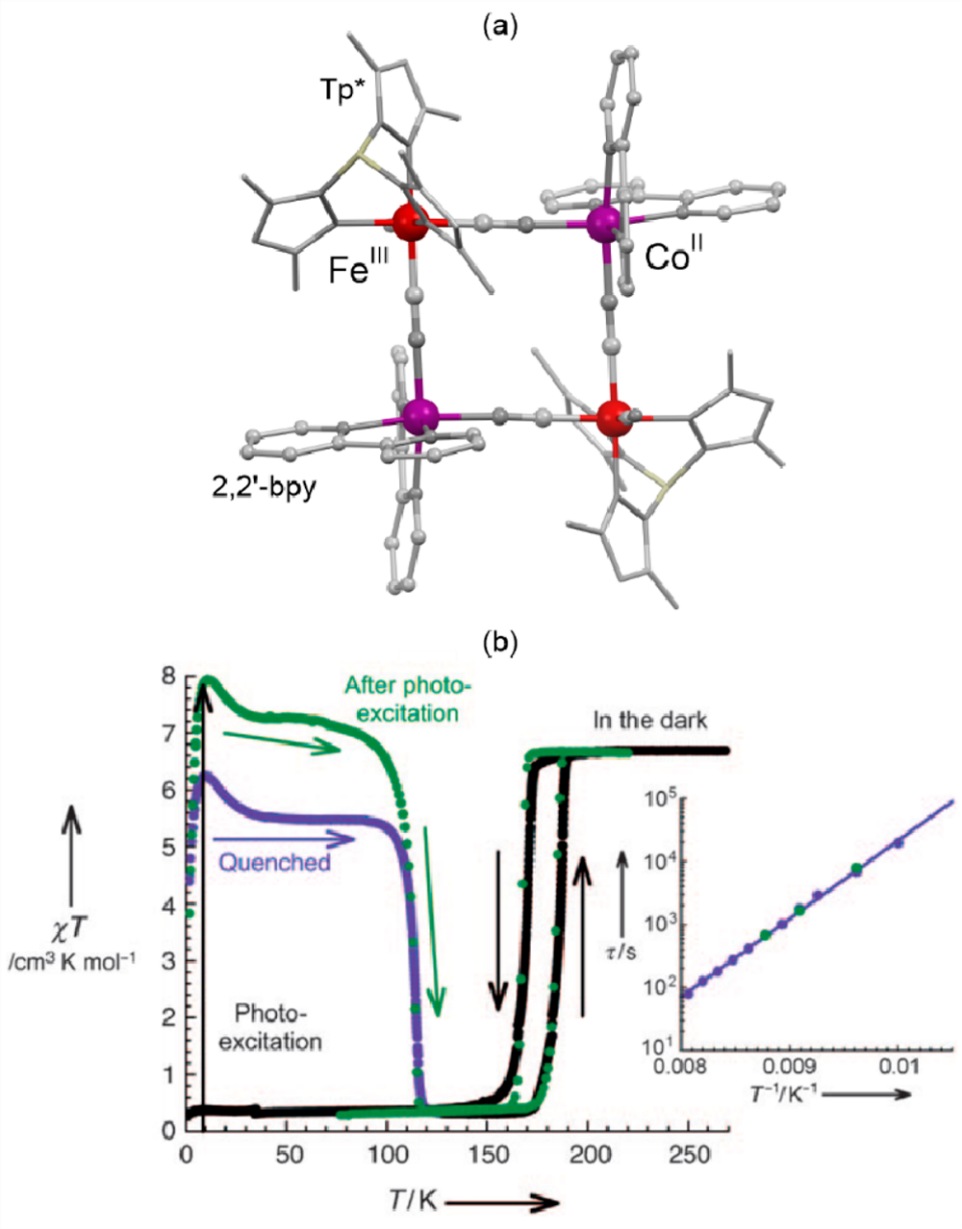
The structure of {[Fe^III^(CN)_3_(Tp*)]_2_[Co^II^(2,2′-bpy)_2_]_2_}(OTf)_2_·4dmf·2H_2_O (OTf^–^ = trifluoromethanesulfonate anion; 2,2′-bpy = 2,2′-bipyridine; Tp* = tris(3,5-dimethyl)pyrazolylborate) molecular squares (a),^348^ the temperature dependences of the *χ*_M_*T* product in the dark, after white light irradiation at 10 K, and for the quickly cooled (quenched) sample (b), shown together with the temperature dependence of the relaxation time of the photogenerated state for the thermally quenched (blue) and irradiated (green) samples (the inset). Part (b) was adapted with permission from ref (348). Copyright 2010 John Wiley & Sons.

Since the original discovery of the photomagnetic effect in the CoFe PBA, some of the work was devoted to obtaining molecular systems based on Co and Fe complexes that will retain temperature- and light-induced charge-transfer properties. For the first time, the temperature-induced ETCST for a molecular system was observed in a {[Co^II^(tmphen)_2_]_3_[Fe^III^(CN)_6_]_2_} (tmphen = 3,4,7,8-tetramethyl-1,10-phenanthroline) cluster in 2004, and much later the presence of the related photoinduced transition was also reported.^11,337,569^ On the other hand, the first example of the photoinduced ETCST for a molecular system was reported in 2007. This was observed for a {[Fe^III^(CN)_3_(pzTp)]_4_[Co^II^(tpmCH_2_OH)]_4_}(ClO_4_)_4_·13dmf·4H_2_O (pzTp = tetra(pyrazolyl)borate; tpmCH_2_OH = 2,2,2-tris(pyrazolyl)ethanol) cubic molecule.^51^ Such a system, upon cooling, reveals an abrupt decrease of *χ*_M_*T* value at ca. 250 K without thermal hysteresis related to an almost complete charge transfer transition from HS Co^II^ and LS Fe^III^ towards LS Co^III^ and LS Fe^II^ centers. Upon white light irradiation at 30 K, the reverse process of charge transfer character is observed, and the resulting metastable state quickly relaxes when the temperature is increased up to 180 K. Since this report, the studies regarding charge-transfer molecular systems with decreased number of metal centers advanced. The next system, presented by R. Clérac, C. Mathonière, S. M. Holmes, and co-workers, composes of cyanido-bridged {[Fe^III^(CN)_3_(Tp*)]_2_[Co^II^(2,2′-bpy)_2_]_2_}(OTf)_2_·4dmf·2H_2_O (OTf^–^ = trifluoromethanesulfonate; 2,2′-bpy = 2,2′-bipyridine; Tp* = tris(3,5-dimethyl)pyrazolylborate) squares (Figure 19a).^348^ Here, the presence of high transition cooperativity resulting in the ca. 20 K-wide thermal charge-transfer hysteresis loop is observed using SQUID magnetometry, surface refractivity, and differential scanning calorimetry. At 10 K, white light irradiation induces the ETCST, and the photogenerated metastable state thermally relaxes near 120 K (Figure 19b). The same relaxation temperature was observed for a thermally quenched high-temperature phase. The isothermal relaxation time for the trapped metastable state changes with the temperature following the Arrhenius law, showing extreme stability of a photoexcited phase at cryogenic temperatures, e.g., 145(15) years at 70 K.^348^

**Figure 20:**
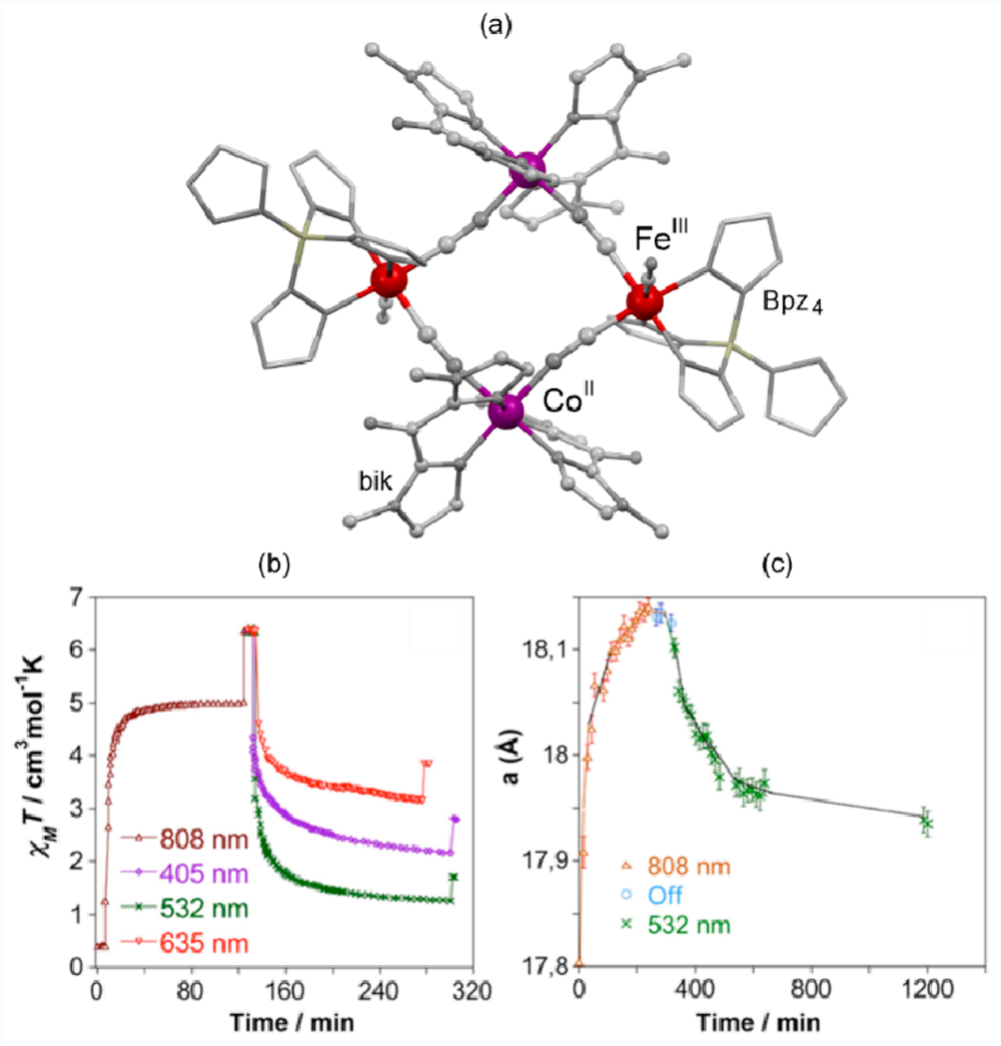
The structural view on {[Fe^III^(Bpz_4_)(CN)_3_]_2_[Co^II^(bik)_2_]_2_}(ClO_4_)_2_·3H_2_O (Bpz_4_ = tetrapyrazolylborate, bik = bis(1-methylimidazol-2-yl)ketone) molecular squares (a),^363^ the time evolution of the *χ*_M_*T* product under irradiation with different indicated light wavelengths at 20 K (b), and the time variation of the *a* unit cell parameter upon successive laser irradiation of 808 and 532 nm at 15 K (c). Parts (b) and (c) were adapted with permission from ref (363). Copyright 2013 American Chemical Society.

Different molecular squares based on Co centers and Fe cyanido complexes showing photoinduced electron transfer were also reported.^352,360,363,570^ One of them, formed from [Fe^III^(Bpz_4_)(CN)_3_]^−^ (Bpz_4_ = tetrapyrazolylborate) and [Co^II^(bik)_2_]^2+^ (bik = bis(1-methylimidazol-2-yl)ketone) complexes (Figure 20a), remain at the ETCST-induced diamagnetic {Fe^II^_2_Co^III^_2_} state over the whole 2–300 K temperature range.^363,570^ Upon white light irradiation at ca. 10 K, this system shows a photoinduced transition to the paramagnetic {Fe^III^_2_Co^II^_2_} state. In the dark, the metastable state relaxes at around 100 K. Moreover, the best results for the photoinduced ETCST were found using the 808 nm light. After saturation of the photomagnetic effect, further irradiation with 532 nm light decreases the *χ*_M_*T* value to the level of ca. 73.5% recovery of the pristine state. Such behavior is expected as the 532 nm light also induces the ETCST effect to the ca. 11% level of conversion starting from the initial state. Nevertheless, changes observed within successive irradiation cycles are repeatable at 20 K using 808 nm and 532 nm light wavelengths (Figure 20b,c).^363^

T. Liu, O. Sato, and co-workers reported a trinuclear system composed of Co and Fe centers, where ETCST changes the polarity of the molecule. The {[Fe^III^(Tp)(CN)_3_]_2_[Co^II^(Meim)_4_]}·6H_2_O (Tp = hydrotris(pyrazolyl)borate; Meim= *N*-methylimidazole) units undergo a hysteretic thermal ETCST, while 532 nm light irradiation at 5 K induces partial electron transfer within LS Co^III^ and LS Fe^II^ pairs, towards HS Co^II^ and LS Fe^III^ state.^571^ Both high-temperature (HT) and low-temperature (LT) phases are described by centrosymmetric space groups as thermal ETCST occurs randomly for only one of the Fe(III) complexes of each molecule. Nevertheless, DFT calculations performed for the HT sextet state and LT doublet state gave the change in the value of the electric dipole moment for each molecule from 0 D to 18.4 D.

**Figure 21:**
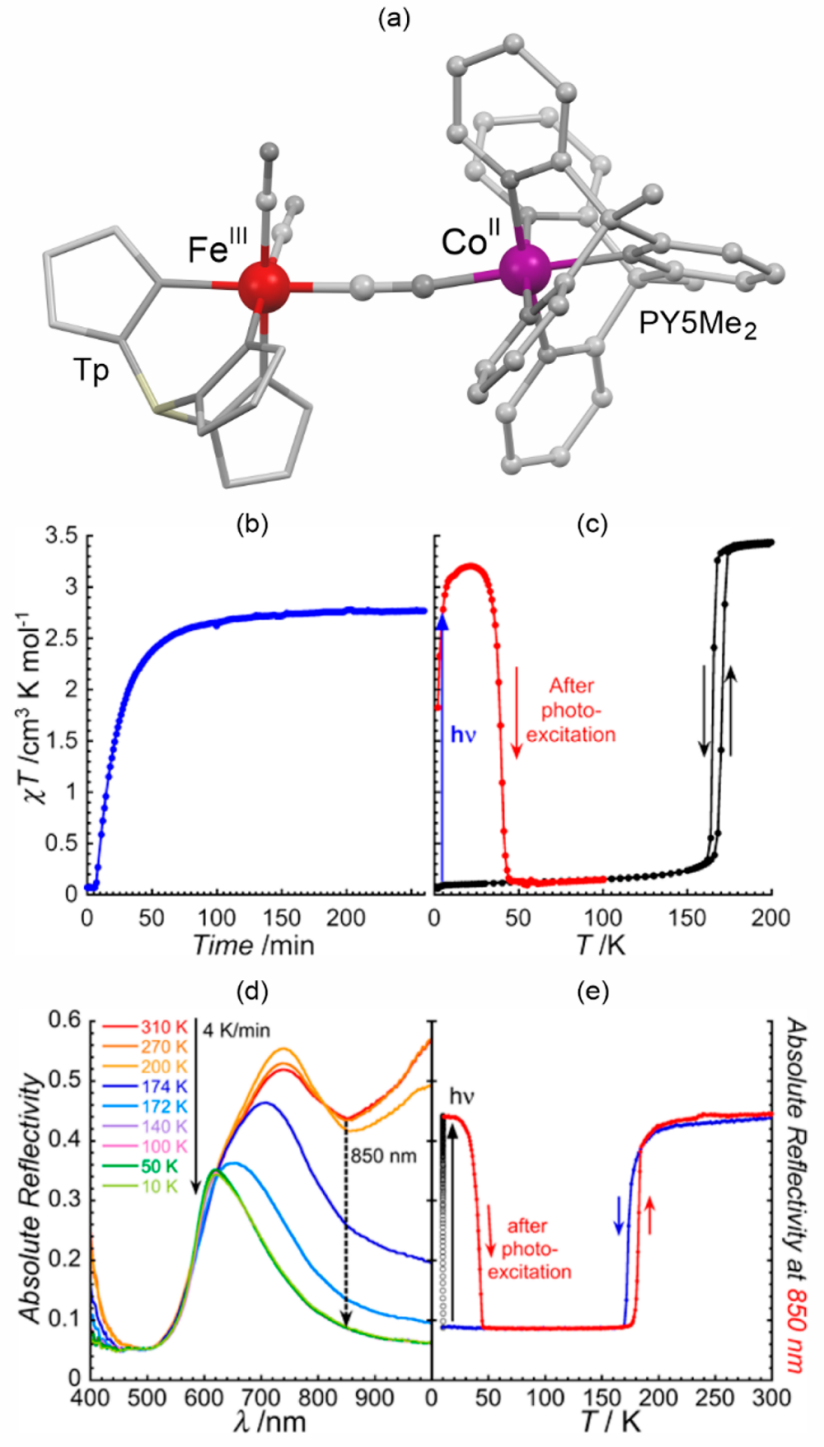
The structure of {[Fe^III^(CN)_3_(Tp)][Co^II^(PY5Me_2_)]}^+^ (Tp = hydridotris(pyrazol-1-yl)borate, PY5Me_2_ = 2,6-bis(1,1-bis(2-pyridyl)ethyl)pyridine) dinuclear molecular cation (a),^361^ the time dependence of the *χ*_M_*T* product under white light irradiation at 10 K (b), the temperature dependence of the *χ*_M_*T* before and after white light irradiation at 10 K (c), the variation of surface refractivity upon cooling from 300 to 10 K (d), and the temperature dependence of absolute refractivity at 850 nm before and after white light irradiation at 10 K. Parts (b), (c), (d), and (e) were adapted with permission from ref (361). Copyright 2014 American Chemical Society.

To further reduce the number of cyanido bridges within a charge-transfer molecular system, thus to obtain a binuclear molecule built of Co and Fe centers, a pentadentate preorganized ligand was employed to block the Co site.^361,572^ Such an approach resulted in the synthesis of the solvated {[Fe^III^(CN)_3_(Tp)][Co^II^(PY5Me_2_)]}(CF_3_SO_3_)·2dmf (Tp = hydridotris(pyrazol-1-yl)borate; PY5Me_2_ = 2,6-bis(1,1-bis(2-pyridyl)ethyl)pyridine, Figure 21a) system, which upon cooling shows only partial charge transfer transition. After the removal of dmf molecules of crystallization by sonification of the crystals in diethyl ether, {[Fe^III^(CN)_3_(Tp)][Co^II^(PY5Me_2_)]}^+^ molecular cation reveal almost complete thermal ETCST within one abrupt step with a narrow thermal hysteresis loop of ca. 5 K. Its irradiation at 10 K with white light increases the *χ*_M_*T* value. The metastable paramagnetic state relaxes at ca. 45 K, which can be followed using either SQUID magnetometry or by following surface refractivity at 850 nm (Figure 21b–e).^361^

**Figure 22:**
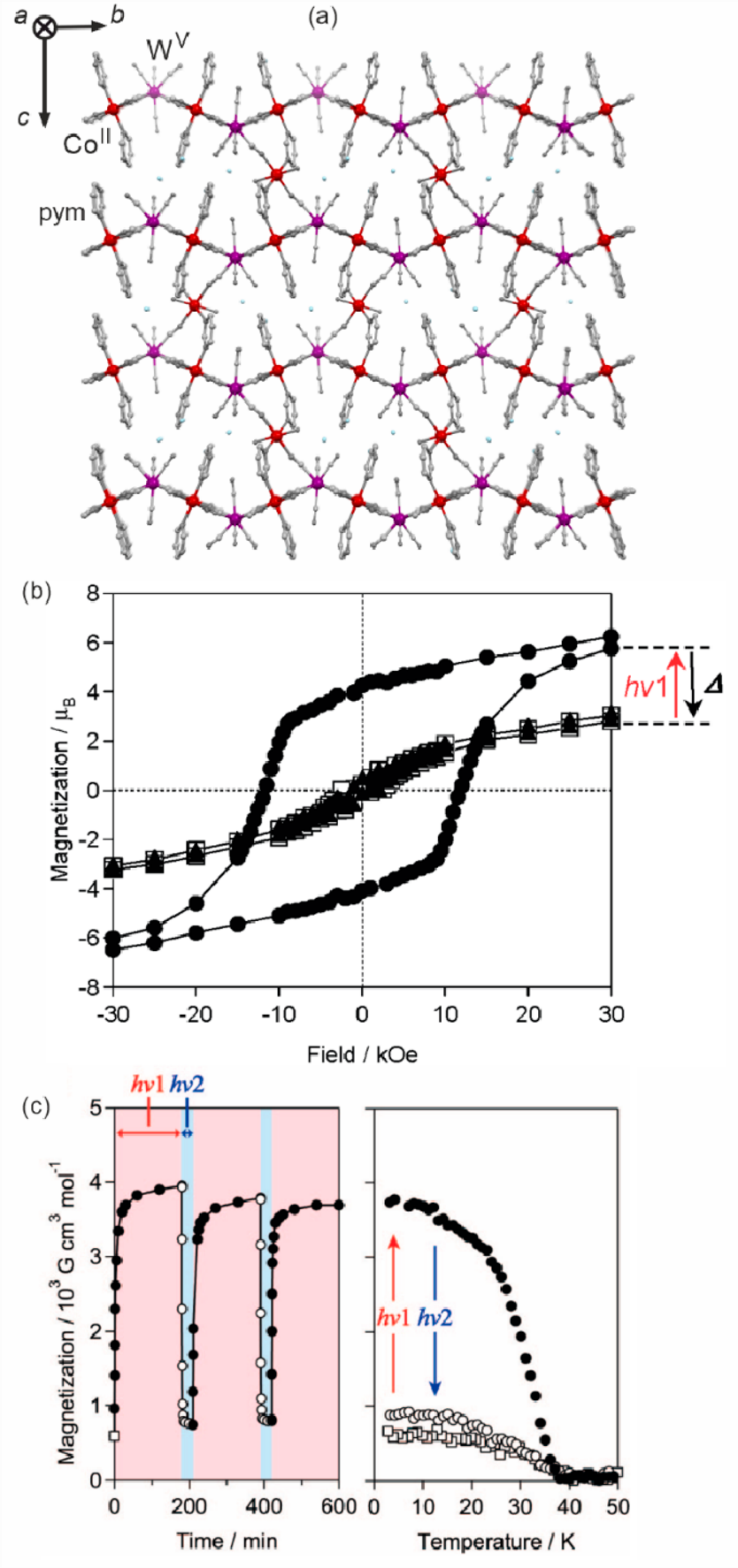
The structure of {[Co^II^(pym)_2_]_2_[Co^II^(H_2_O)_2_][W^V^(CN)_8_]_2_}·4H_2_O (pym = pyrimidine) coordination network (a),^377^ its magnetization versus field curve at 2 K before irradiation, after 120 min of the 840 nm light irradiation, and after thermal relaxation at 150 K (b), and the magnetization versus time curve within the cycles of the successive 840 nm and 532 nm light irradiation at 10 K, shown together with the temperature dependence of magnetization measured in the dark, after irradiation with the 840 nm light, and after the subsequent photoirradiation with the 532 nm light (c). Parts (b) and (c) were adapted with permission from ref (377). Copyright 2008 American Chemical Society.

Similarly to CoFe PBA and its molecular analogs, coordination networks built of Co^II^ centers and octacyanidotungstate(V) ions, [W^V^(CN)_8_]^3–^ can reveal a thermal ETCST phenomenon, accompanied by the presence of photoinduced reverse process at cryogenic temperatures.^573^ Such behavior was first reported in a Cs^I^{[Co^II^(3-cyanopyridine)_2_][W^V^(CN)_8_]}·H_2_O layered system, which upon cooling reveals large thermal hysteresis loop between 167 and 216 K.^346^ The thermal charge transfer transition occurs for 95% of magnetic centers, starting from HS Co^II^ (*S* = 3/2) and paramagnetic W^V^ (*S* = 1/2), and resulting in the low-temperature (LT) phase composed of LS Co^III^ (*S* = 0) and diamagnetic W^IV^ (*S* = 0) centers. Upon irradiation of the LT phase at 5 K with red light, the metastable state of ferromagnetically coupled HS Co^II^ and W^V^ centers is obtained, which upon heating reveals *T*_c_ of ca. 30 K, and then thermally relaxes at 120 K. Another system, a three-dimensional {[Co^II^(pyrimidine)_2_]_2_[Co^II^(H_2_O)_2_][W^V^(CN)_8_]_2_}·4H_2_O coordination framework shows an even higher temperature of magnetic phase transition of 40 K after photoexcitation with 840 nm light (Figure 22).^376,377^ The presence of remaining isolated HS Co^II^ centers at low temperatures results in paramagnetism of the LT phase, while after light irradiation large magnetization versus field hysteresis loop is observed at 2 K with the coercive field of 12 kOe. Although this system can be relaxed to the initial state by thermal treatment (150 K), the paramagnetic phase can be almost fully recovered using 532 nm light irradiation. The next network, {[Co^II^(4-methylpyridine)(pyrimidine)]_2_[Co^II^(H_2_O)_2_][W^V^(CN)_8_]_2_}·4H_2_O, shows an even higher coercive field of 27 kOe after photoirradiation at 2 K, accompanied by higher *T*_c_ value of 48 K.^52^ Moreover, a two-dimensional (H_5_O_2_){[Co^III^(4-bromopyridine)_2_][W^IV^(CN)_8_]} network, that retains the respective oxidation states of metal centers within the whole measured temperature range, upon 785 nm light irradiation at 3 K undergoes the photogenerated ETCST effect toward the Co^II,HS^–W^V^ metastable state relaxing at ca. 80 K.^574^ The intervalence mechanism of the photomagnetic effect was supported by first-principles calculations of the electronic structure, finding reflection in the optical absorption spectra.

#### 3.2.2 Molecular Nanomagnets Revealing Photoswitchable Electron Transfer

Just like the LIESST effect, the photoinduced electron transfer phenomenon may be involved in the construction of molecular nanomagnets. As the ETCST systems are already rare compared to the spin transition materials showing the LIESST effect, and the strong intracluster (for the case of SMMs) or intrachain (for the case of SCMs) magnetic coupling has to be induced after the photoinduced electron transfer which demands the high photoconversion level, it is a challenging task to photogenerate the SMM/SCM systems. As a result, there are only a few examples of ETCST-related photoswitchable molecular nanomagnets.

For a cubic molecular system reported by F. Breher, R. Lescouëzec, and co-workers, K^I^{[Fe^II/III^(Tp)(CN)_3_]_4_[Co^II/III^(pzTp)]_4_} (Tp = hydridotris(pyrazol-1-yl)borate; pzTp = tetrakis(pyrazolyl)borate) only one Co center at room temperature corresponds to HS Co^II^ state, while all other three are at the LS Co^III^ state.^575^ Upon increasing the temperature to 400 K some onset of thermal ETCST can be observed, while its decrease does not affect the HS Co^II^ center. Therefore, at cryogenic temperatures, the system remains paramagnetic and reveals the SMM behavior up to 10 K under 1.8 kOe external field, with an energy barrier of 34 cm^–1^. Upon irradiation with 808 nm light at 20 K, the ECTST is induced and the effect of slow relaxation of magnetization disappears.

**Figure 23:**
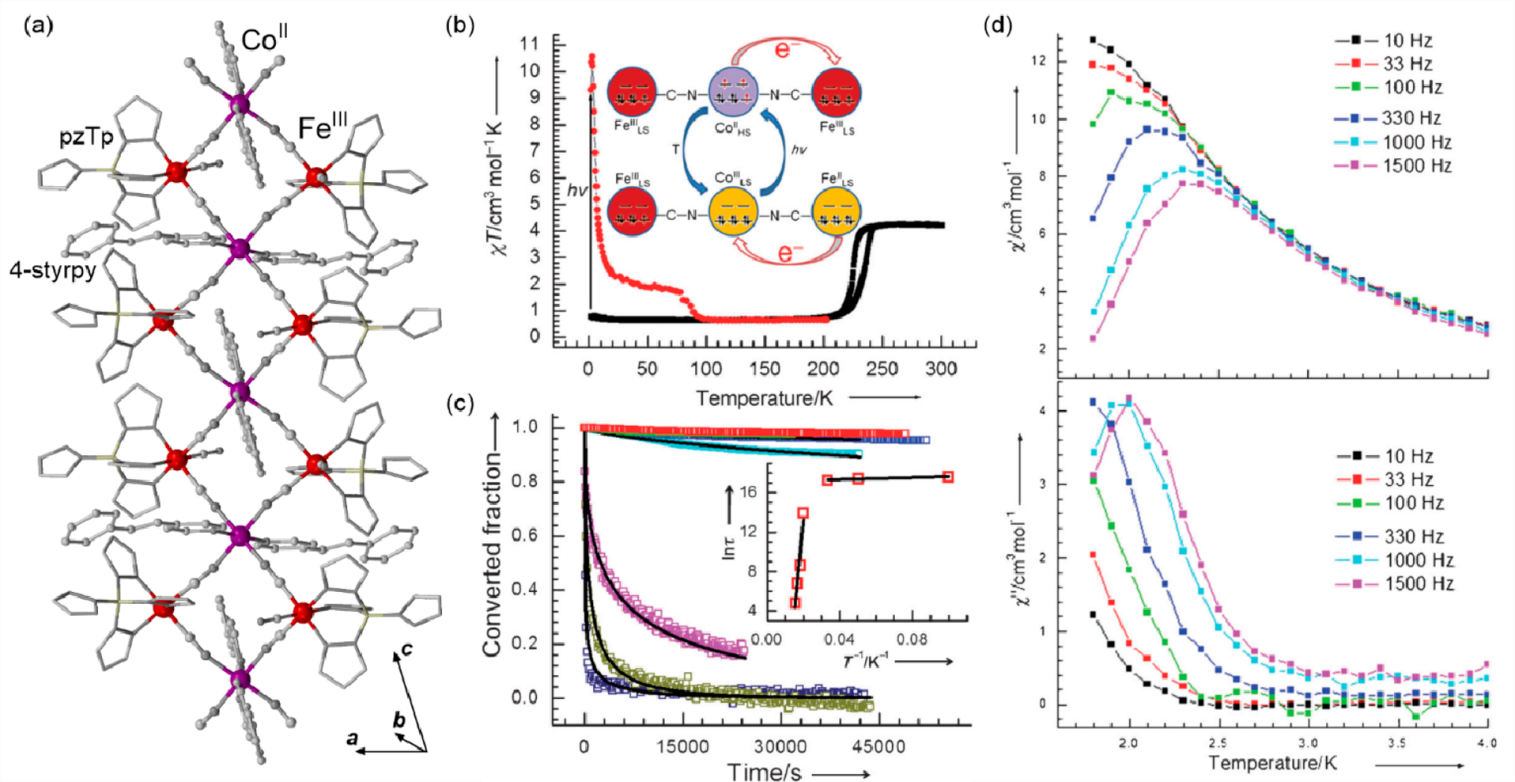
The structure of {[Fe^III^(CN)_3_(pzTp)]_2_[Co^II^(4-styrpy)]}·2H_2_O·2MeOH (pzTp = tetrakis(pyrazolyl)borate; 4-styrpy = 4-styrylpyridine) coordination chain (a),^576^ its temperature dependence of the *χ*_M_*T* product before and after 532 nm light irradiation at 5 K, shown together with the scheme of the observed thermal and photoinduced electron transfer process (b), the relaxation kinetics of the photogenerated fraction at different temperatures (c), and the temperature dependences of the real and imaginary parts of the *ac* magnetic susceptibility under zero *dc* field after irradiation using the 532 nm light (d). Parts (b), (c), and (d) were adapted with permission from ref (576). Copyright 2012 John Wiley & Sons.

In some cases the presence of magnetic coupling between photogenerated HS Co^II^ (*S* = 3/2) and LS Fe^III^ (*S* = 1/2) results in the SCM behavior, when the chain units are sufficiently separated within a sublattice. For example, such behavior was observed for {[Fe^III^(CN)_3_(pzTp)]_2_[Co^II^(4-styrpy)_2_}·2H_2_O·2MeOH (pzTp = tetrakis(pyrazolyl)borate; 4-styrpy = 4-styrylpyridine) system that shows cooperative thermal ETCST effect in the 220–240 K range (Figure 23).^576^ After the 532 nm light irradiation at cryogenic temperatures, the photoinduced ETCST occurs and results in the SCM behavior with an energy barrier of 27 K. The photogenerated state relaxes to the thermodynamically stable diamagnetic one quite readily when the temperature is raised above 60 K.

**Figure 24:**
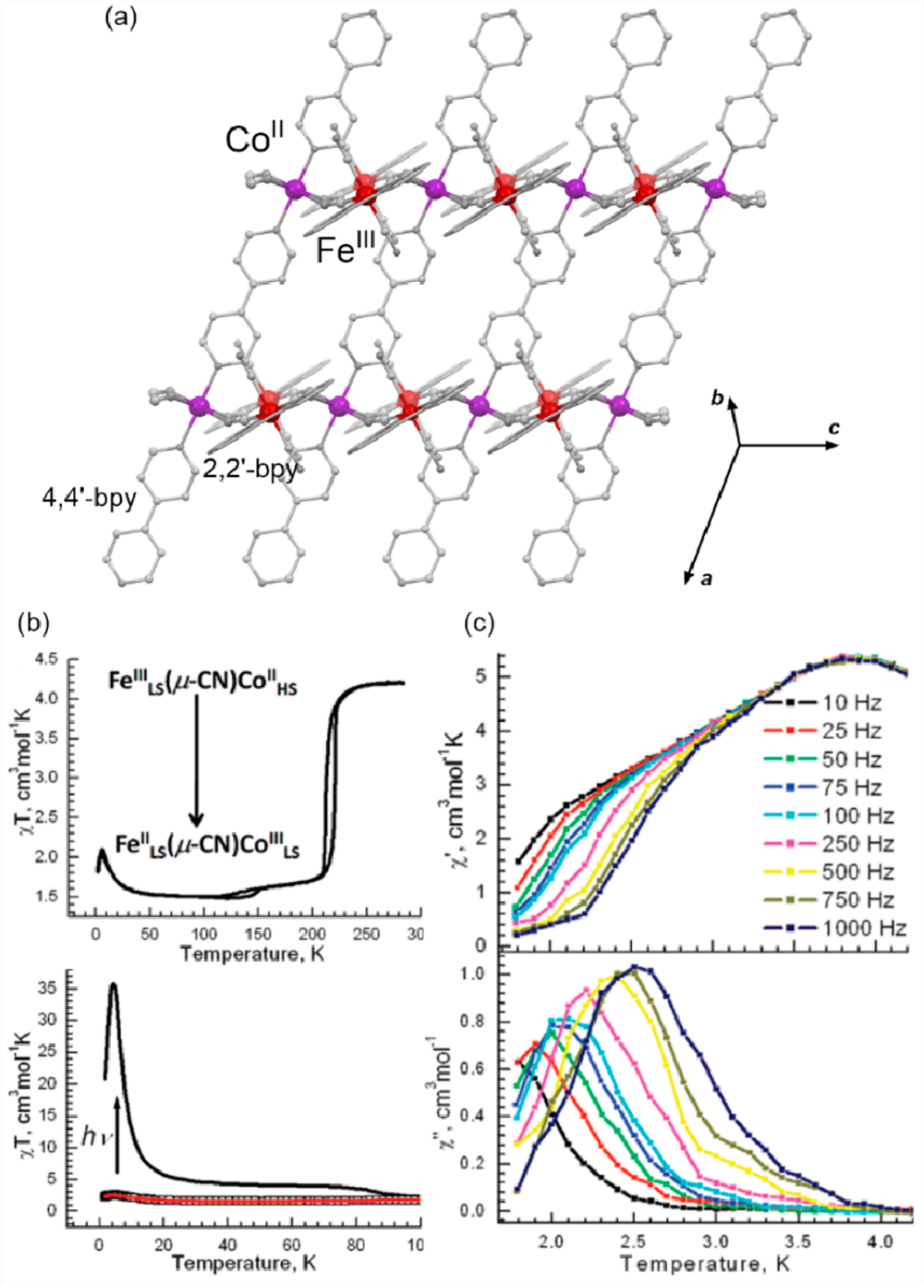
The representative structural view on {[Fe^III^(2,2′-bpy)(CN)_4_]_2_[Co^II^(4,4′-bpy)]}·4H_2_O (2,2′-bpy = 2,2′-bipyridine; 4,4′-bpy = 4,4′-bipyridine) layered coordination polymer (a),^577^ the temperature dependence of the *χ*_M_*T* product in the dark (b, upper part), as well as after the 532 nm light irradiation at 5 K and after thermal relaxation at 150 K (b, bottom part), and the temperature dependences of the real and imaginary parts of the *ac* magnetic susceptibility under zero *dc* field after irradiation with the 532 nm light (c). Parts (b) and (c) were adapted with permission from ref (577). Copyright 2010 American Chemical Society.

Another system, which is built of the {[Fe^III^(2,2′-bpy)(CN)_4_]_2_[Co^II^(4,4′-bpy)]}·4H_2_O (2,2′-bpy = 2,2′-bipyridine; 4,4′-bpy = 4,4′-bipyridine) coordination layers, reveals a thermal ETCST above 200 K but only related to the ca. 2/3 of the present Fe and Co sites (Figure 24).^577^ At cryogenic temperatures, this material reveals a local *χ*_M_*T*(*T*) maximum at 6 K, due to the presence of magnetic interactions within remaining discrete paramagnetic clusters. After 532 nm light irradiation, the paramagnetic HS Co^II^ (*S* = 3/2) and LS Fe^III^ (*S* = 1/2) sites are generated, and upon heating the metastable state relaxes at ca. 100 K. The low-temperature photoinduced phase shows a distinct SCM behavior originating from magnetically coupled Co^II,HS^ and Fe^III,LS^ centers, although, additional antiferromagnetic interactions appear between the cyanido-bridged chains, mediated by the 4,4′-bpy linkers and hydrogen-bonded network. The extracted energy barrier of 29 K is similar to the previous case and this system lacks the magnetization versus field hysteresis loop down to 1.8 K as well before and after irradiation. For both systems, the photoinduced ETCST effect was monodirectional, while the temperature was used to relax the metastable state.^577^ Upon exchanging the 4,4′-bpy bridging ligand for monodentate 4-phenylpyridine (4-phpy), the {[Fe^III^(2,2′-bpy)(CN)_4_]_2_[Co^II^(4-phpy)_2_]}·2H_2_O system composed of zigzag chains was obtained.^109^ When irradiated with 808 nm light at cryogenic temperatures the ETCST phenomenon appears and the resulting metastable phase reveals the SCM behavior. The subsequential irradiation with 532 nm light partially induces the reverse transformation to the LS Co^III^ and LS Fe^II^ states. Such partial transition is sufficient to quench the SCM behavior, thus it can be bidirectionally switched by alternating the irradiation wavelength. Similar behavior was also reported for the {[Fe^III^(CN)_3_(pzTp)]_2_[Co^II^(bpi)_2_]}·MeCN·4H_2_O (pzTp = tetrakis(pyrazolyl)borate; bpi = 1-biphenyl-4-yl-1H-imidazole) chain system.^578^ Moreover, an example of a photoinduced SCM, based on the chiral {[Co^II^((*R*)-pabn)][Fe^III^(Tp)(CN)_3_]}(BF_4_)·MeOH·H_2_O ((*R*)-pabn = (*R*)-*N*(2),*N*(2′)-bis(pyridine-2-ylmethyl)-1,1′-binaphtyl-2,2′-diamine) chain system, showing switchable *dc* electronic conductivity was also presented in the literature by H. Oshio and co-workers but its case will be presented in detail in section 7.4 devoted to extended multifunctionality in optical molecule-based magnetic materials.^238^

### 3.3 Optical Switching Using Photochromic Ligands

A different method for achieving photoswitchable physical properties is based on the use of photoresponsive organic molecules, quite often serving as ligands attached to the d- or f-block metallic centers.^166,170,579^ Photoinduced electron transfer operating for the viologen derivatives is only one example of stimuli-responsive purely organic switches,^515^ but different groups of systems can undergo more pronounced structural changes such as photoisomerization (e.g., azobenzenes, azopyridines),^580−586^ photocyclization (e.g., diarylethenes, spiropyrans),^427,587−597^ and the associated photodissociation,^598,599^ which result in thermally stable photogenerated states at room temperature or above. In basics, every one of these processes can be reversed by different light irradiation but, moving to the solid state, this feature is often easily quenched by supramolecular interactions. However, up to now, numerous studies have been undertaken to achieve light-modulated (bio)sensors,^600^ luminophores,^597^ optical memory devices,^601^ and molecular machines.^602,603^ Recent reports regarding photoswitchable conductors,^112^ separating membranes,^604^ and catalysts^605−607^ also utilize the effect of light-induced structural modifications, broadening the application horizon of this class of molecular materials. Despite the intrinsic switchability of the above-mentioned organic entities, serving as ligands, they also enrich various functionalities of MOF materials.^605^

Aiming at molecular magnetic switches, some ideas of room-temperature photomagnetism were proposed. As a structural change within the first coordination sphere modifies the crystal field strength, the mechanism of room-temperature photoswitching of the spin-state for d-block metal complexes was proposed, which is called ligand-driven light-induced spin change (LD-LISC).^608^ Moreover, post-synthetic modification of the ligand also modifies the redox potential of the complex, thus lately a more complex example of the photoisomerization-induced spin-change excited state (PISCES) effect of the electron transfer transition nature was observed.^609^ However, even for purely paramagnetic or exchange-coupled systems, changing the ligand field should affect the magnetic anisotropy of d- and f- metal ions, while structural reorganization of the ligands modifies the phonon mode scheme of crystalline solids.

Among spin-bearing systems showing light-switchable magnetic properties, some discussion should be given to purely organic compounds. Then instead of d- or f-block metal ions, spin functionality originates from radical units that are accompanied within the molecular structure by photochromic units. As for the organic systems such photochromic processes as photocyclization, photoisomerization, or photodissociation are accompanied by large structural rearrangement, most of the studies were performed in solution, while in the solid state, the photochromic behavior may be quenched due to steric hindrance and by the appearance of inter- or intramolecular interactions. Initially, the studies regarding magnetic photoswitches were devoted to molecules involving nitronyl nitroxides with a diarylethene spacer.^424,610−612^ For such systems the photoreversible reaction affects the magnetic interactions between oxygen radicals changing the *χ*_M_*T*(*T*) course and the EPR spectrum. A different mechanism was presented by B. Feringa and co-workers who presented the two TEMPO (2,2,6,6-tetramethylpiperidine-1-oxyl) radicals bridged by an extended alkene unit.^613^ Using the 312 nm and 365 nm light, this system can be reversibly switched between *E* and *Z* configurations. For the *E* isomer, the magnetic interactions are negligible, while the *Z* state, stabilized by an intramolecular hydrogen bond, reveals strong coupling between radicals, and thus modified EPR spectrum. Different behavior is observed for a heterocyclic bisdithiazolyl radical (*S* = 1/2).^614,615^ At room temperature in the solid state, two radicals form a dimer with hypervalent 4-center S···S–S···S σ-bonds. Upon heating, such dimers undergo a structural transition accompanied by spin change due to the separation of radicals. As shown by optical absorption spectra, the same transformation can be also induced by 650 nm light at 100 K for the thin film samples, and the resulting monoradical phase remains stable up to ca. 220 K. Photomagnetic experiment at 10 K results in a paramagnetic metastable state that shows a gradual increase of *χ*_M_*T* on warming, due to weakening of antiferromagnetic interactions between radicals. At 242 K, the sample relaxes readily to the diamagnetic state, reproducing the original properties of the thin film material. A successful photogeneration of radical systems was presented in glassy matrices of 2-methyltetrahydrofuran at 77 K.^616^ The reported compounds were obtained in the closed form from 1,14-dimethyl-[5]helicene by chemical modifications at the 4,11-positions with O or C(CN)_2_ substituents. At 77 K, both systems are EPR silent but after irradiation with 375 nm and 405 nm light, respectively, the open, diradical forms are generated, showing distinct EPR signals. The light-induced process is reversed by thermal treatment, that is at 140 K and 92 K, respectively.

In the two following sections, we will discuss the role of analogous photoswitchable organic ligands in photoswitching of magnetic and optical phenomena in molecular materials revealing spin transition effects (3.3.1) and those serving as molecular nanomagnets (3.3.2).

#### 3.3.1 Spin Transition Materials Incorporating Photochromic Ligands

**Figure 25:**
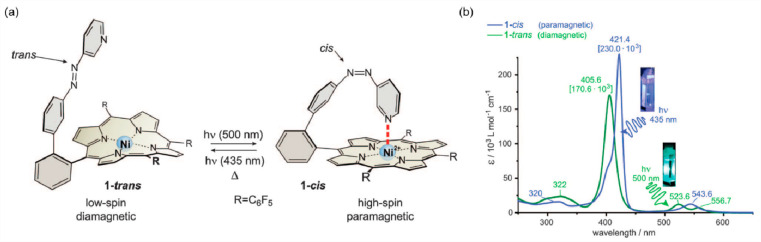
The schematic representation of the reversible light-induced magnetic switching of the azopyridine-functionalized Ni(II)-porphyrin molecular system occurring in solution (a) and the related UV–vis absorption spectra of the *trans* and *cis* isomers of this Ni(II)-porphyrin complex with the indicated light stimuli modulating the spectra (b). Reproduced with permission from ref (617). Copyright 2011 American Association for the Advancement of Science.

Aiming at the photocontrol of the spin transition phenomena beyond the LIESST effect (see section 3.1 above), various organic photoswitchable ligands were employed for the construction of coordination complexes. Among such systems, interesting results were presented in solution for Ni(II) complexes with azopyridine ligands (Figure 25).^617^ Ni(II) centers when embedded in an octahedral crystal field usually reveal the HS state (*S* = 1), while for the related four-coordinated complexes, the LS state (*S* = 0) is favored. For the coordination number of five, the resulting square pyramidal complexes are either HS or LS depending on the nature of the ligands. Therefore, by using the azopyridine-functionalized porphyrin ligand, R. Herges and co-workers achieved photoinduced spin-state switching at room temperature in an acetonitrile solution.^617^ In the as-synthetized Ni(II) complex, the azopyridine adopts the *E* configuration, thus the metallic center with four donor atoms of the porphyrin ring remains at its LS state. After 500 nm light irradiation the azopyridine achieves *Z* isomer, and its N donor atom coordinates the Ni(II) center. The resulting five-coordinated complex has the HS state that is paramagnetic (*S* = 1). The spin-switching has a ca. 65% yield in acetonitrile and a ca. 75% yield in dimethyl sulfoxide. Then upon irradiation with 435 nm light, the original state can be recovered, while the *Z* to *E* isomerization occurs in 97% yield. Another photoswitchable system based on Ni(II) centers was obtained by employing tetrakis(pentafluorophenyl)porphyrin.^618^ Here, a strategy of dissolving the Ni(II) in the solution containing 4,4′-di-*i*Pr-3,3’-azobispyridine to achieve photoswitching at room temperature was used. The azopyridine-type ligand was designed to coordinate the Ni(II) center in its *E* configuration and dissociate after photoconversion to the *Z* form due to steric hindrance. As a consequence, bidirectional switching between the diamagnetic and paramagnetic states of Ni(II) was observed with alternating irradiation at 365 and 455 nm. However, the observed changes oscillate between the level of 20.5% and 68.1% of *S* = 1 state. Similar results were obtained when 4,4′-di-*i*Pr-3,3′-azobispyridine was changed to 4-methyl-3-(3′,5′-di-tert-butylphenyl)azopyridine.^619^ Moreover, the family of Ni(II) complexes with the azopyridine- and azoimidazole-functionalized porphyrin ligands was tested from the point of photoresponsive MRI contrast agents.^620,621^ Additionally, the Fe(III) complex with porphyrin derivative was reported to show spin-state photoswitching between the LS (*S* = 1/2) and HS (*S* = 5/2) states in a dimethyl sulfoxide solution in the presence of 4-methoxy-3-(3′,5′-di-tert-butylphenyl)azopyridine upon alternating 365 and 435 nm light irradiation.^622^

**Figure 26:**
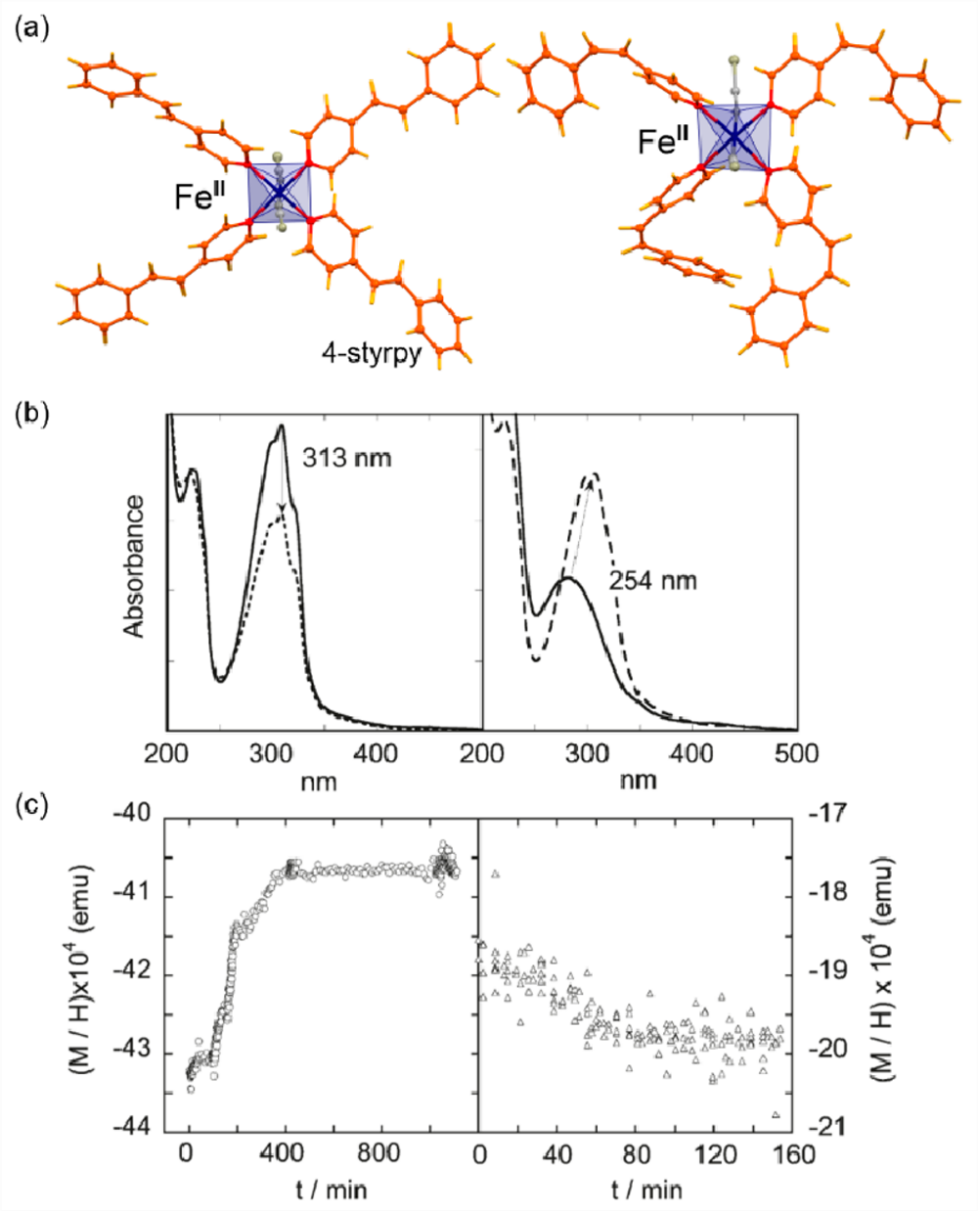
The structures of the all-*trans* (left, *E* isomers) and all-*cis* (right, *Z* isomers) metal complexes of [Fe^II^(4-styrpy)_4_(NCSe)_2_] (4-styrpy = 4-styrylpyridine) (a),^624^ UV–vis absorption spectra at 120 K of PMMA (poly(methyl methacrylate)) thin films filled with all-*trans* (left) and all-*cis* (right) Fe(II) metal complexes before and after irradiation using the indicated wavelengths (b), the time-dependent magnetization over field curves under the 355 nm light irradiation at 130 K for the PMMA thin films filled with the above-mentioned all-*trans* (left) and all-*cis* (right) complexes (c). Parts (b) and (c) were adapted with permission from ref (624). Copyright 2009 American Chemical Society.

As most of the spin-crossover systems are built of Fe(II) centers, their complexes were also intensively studied from the point of ligand-driven light-induced spin change (LD-LISC) effect. For instance, {[Fe^II^(*E-*4-styrpy)_4_(NCBPh_3_)_2_]} and [Fe^II^(*Z*-4-styrpy)_4_(NCBPh_3_)_2_] (4-styrpy = 4-styrylpyridine) complexes were isolated.^623^ The first of them undergoes thermal SCO centered at ca. 190 K, while the second remains at the HS state down to cryogenic temperatures. Both systems were studied in the form of cellulose acetate thin films including Fe(II) complexes at different concentrations, and the thermal SCO properties were retained as shown by optical absorption studies. At 140 K, upon 322 nm light irradiation of the film containing *E-*isomer, the decrease of absorbance was observed. At the same temperature, the film containing *Z*-isomer upon 260 nm light irradiation increases its UV light absorption. Then for the latter, the temperature variable absorption spectra reveal a similar trend as for the non-irradiated *E-*isomer. Another pair of complexes with similar thermal SCO properties, [Fe^II^(*E-*4-styrpy)_4_(NCSe)_2_] and [Fe^II^(*Z*-4-styrpy)_4_(NCSe)_2_], was studied in the form of doped poly(methyl methacrylate) thin films (Figure 26).^624^ Upon 313 nm light irradiation at 120 K *E*-containing film reveals decreasing absorption, while for the *Z*-containing one upon 254 nm light an increase in the absorbance is present. Photomagnetic experiments performed at 130 K using 355 nm light irradiation indicate a strong increase of magnetization for the *E*-dopped thin film and only a weak decrease of the magnetic signal after irradiating the *Z*-dopped analog. The polycrystalline sample [Fe^II^(*Z*-stpy)_4_(NCSe)_2_] upon 532 nm light irradiation changes its color from orange to red, which is also accompanied by the changes within IR absorption spectra.^625^ Additionally, it turns amorphous as shown by powder X-ray diffraction but magnetic properties of the photo-generated phase reveal thermal SCO, as well as the presence of the LIESST effect at 10 K.

For [Fe^II^(3AzoN_4_Py)(MeCN)](BF_4_)_2_ (3AzoN_4_Py = azopyridine-functionalized pentadentate ligand) complex, the coordination of the ligand reduces the efficiency of the *E*-to-*Z* photoisomerization.^626^ Moreover, the LD-LISC effect is not observed even at room temperature. The ultrafast absorption studies revealed that upon irradiation of the ligand bands, an energy transfer towards MLCT and MC (metal-centered) states of Fe(II) occurs, thus reducing the efficiency of ligand switching. Another example of Fe(II) complex based on azobenzene moieties, *E*_100_,*Z*_0_–[Fe^II^(L_ppbd_)_3_](BF_4_)_2_·3H_2_O (L_ppbd_ = phenyl(2-pyridin-2-yl-3H-benzoimidazol-5-yl)diazene), reveal gradual SCO in the solid state and in the acetone near room temperature.^627^ At room temperature, under 365 nm light irradiation of the latter, the complex reveals modified absorption due to partial *E*-to-*Z* isomerization of the ligands accompanied by the LS-to-HS spin change. Those changes can be reversed by 436 nm light. The conversion under 365 nm light was determined as the photoisomerization of only one ligand (*E*_67_,*Z*_33_), while the reverse process was found incomplete (*E*_87_,*Z*_13_).^627^ Photochromic behavior for bis(dipyrazolylstyrylpyridine)iron(II) complexes can be generated both in solid state and solution. The [Fe^II^(*Z*-L_dpsp_)_2_](BF_4_)_2_ (L_dpsp_ = 2,6-di(1H-pyrazol-1-yl)-4-styrylpyridine) complex displays the HS state both at cryogenic and room temperatures.^628^ Upon 436 nm light irradiation in acetone, the [Fe^II^(*E*-L_dpsp_)_2_](BF_4_)_2_ system is formed. Similar behavior is also observed in the solid state, and the sample shows color change, modification of the IR spectra, and gradual SCO upon cooling. Similar organic ligands modified by electron-withdrawing cyano and nitro substituents were also employed for the construction of Fe(II) complexes.^629^ As earlier, the *Z* form of CN-substituted ligand induces a stable HS state over the whole temperature range, while for the NO_2_-substituted system, a thermal SCO is found next to room temperature. After visible light irradiation (*λ* > 420 nm) at room temperature, the obtained systems with organic ligands in *E* configuration reveal similar, gradual SCO behavior. Therefore, for the CN-substituted system, the *χ*_M_*T* is reduced upon irradiation, while for the NO_2_-substituted one, the trend is opposite.

**Figure 27:**
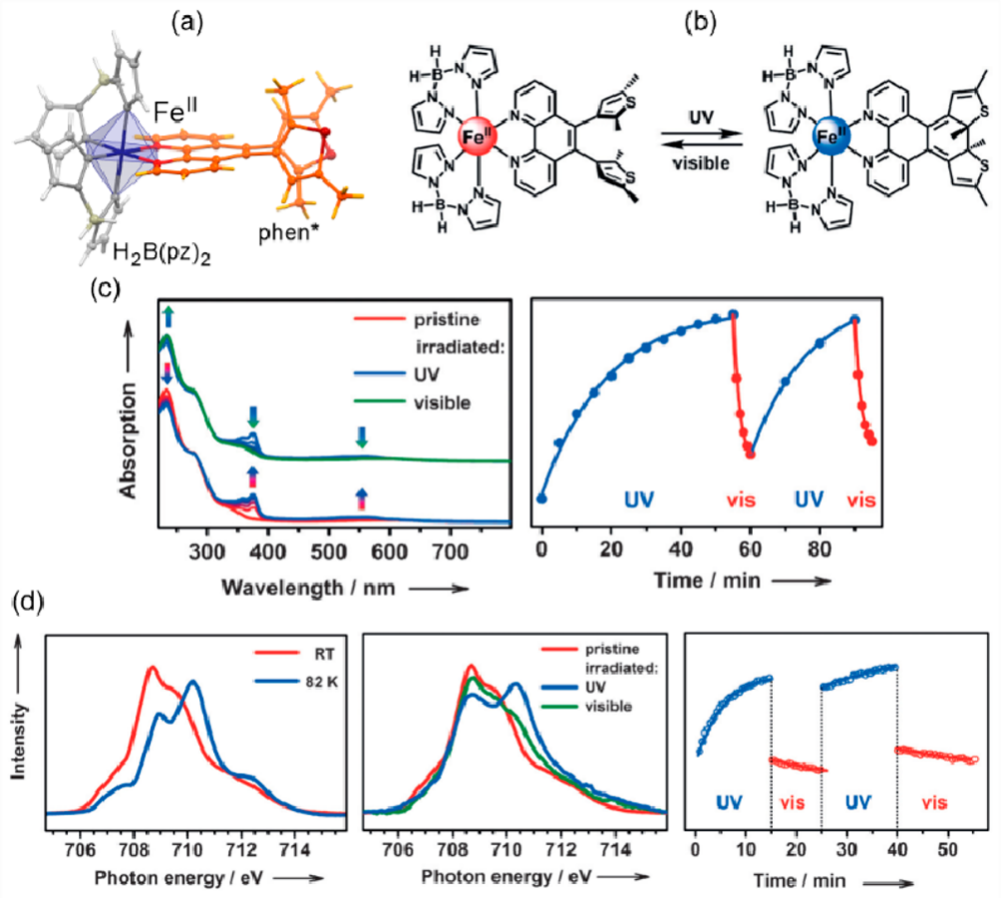
The structure of [Fe^II^(H_2_B(pz)_2_)_2_(phen*)] (pz = 1-pyrazolyl; phen* = 5,6-bis(2,5-dimethylthien-3-yl)-1,10-phenanthroline) metal complex (a),^631^ the schematic representation of the bidirectional switching using photocyclization of the phen* ligand followed by photocycloreversion (b), the UV-induced photocyclization under the 282 nm irradiation and the subsequent photocycloreversion realized by visible light traced by the UV–vis absorption spectra at room temperature, shown together with the cycles of the photoswitching effect investigated following the intensity of the 375 nm absorption band at room temperature (c), and NEXAFS spectra at the FeL_3_ edge at room temperature and at 82 K for the pristine material (left side), room temperature NEXAFS spectra at the FeL_3_ edge for the pristine material and the sample after irradiation with UV or visible light (middle part), shown together with the time dependence of the peak intensity at 710.2 eV in the NEXAFS spectra at the FeL_3_ edge upon alternating irradiation with UV and visible light at room temperature (right side) (d). Parts (b), (c), and (d) were adapted with permission from ref (631). Copyright 2015 John Wiley & Sons.

A different approach towards the LD-LISC system involves the use of diarylethene-based ligands instead of those possessing the photoswitchable double bond. For instance, the reported [Fe^II^(H_2_B(pz)_2_)_2_(phen*)] (pz = 1-pyrazolyl; phen* = 5,6-bis(2,5-dimethyl-3-thienyl)-1,10-phenanthroline) system, upon decreasing the temperature, reveal thermal SCO above ca. 150 K in the solid state (Figure 27a).^425,630,631^ When at 5 K exposed to 637 nm light, the LIESST effect is observed, which can be partially reversed by 808 nm irradiation. At room temperature, upon UV light irradiation in solution, an increased absorption is observed at ca. 380 and ca. 550 nm due to the photocyclization of the organic ligand. Then the photoirradiated solution can be treated with visible light to recover the initial UV–vis spectrum. The temperature-variable absorption spectra in solution before and after UV-light irradiation reveal different courses of the SCO effect. Although at RT, both non-irradiated and irradiated complexes show the HS Fe(II) state, at 173 K clear differences are observed in UV–vis absorption spectra. Room-temperature reversible photosensitivity is found in the solid state, and within several cycles of alternating irradiation closed and open forms of the ligand can be almost quantitively obtained (Figure 27b–d).^631^ The photomagnetic effect was studied at room temperature using NEXAFS spectroscopy. Upon UV irradiation of the powder sample, a similar change of the NEXAFS spectrum is observed in comparison to the one obtained after cooling the sample to 82 K. Furthermore, the use of visible light partially reverses the effect of UV irradiation. The observed changes are attributed to the incomplete LD-LISC effect but some degree of reversibility was postulated.

The same complexes were also used to assemble photochromic SCO thin films on an Au(111) surface.^632^ The XPS spectra revealed, that upon deposition, the resulting 5 nm thin films retain thermally-induced SCO, as well as photoswitching properties after UV light irradiation at room temperature. Later, a sub-monolayer deposit was performed on highly ordered pyrolytic graphite (HOPG).^633^ Again, the thermal SCO property was preserved, however, photomagnetism at room temperature was quenched. Using the *ab initio* calculations, such behavior was rationalized by stabilization of the ligand in its photoinactive form due to absorbate-substrate interactions.

In recent years, an alternative approach to the LD-LISC effect for the Fe(II) SCO complexes was proposed, which consists of the inclusion of photochromic guest molecules in the structural pores of an SCO-active metal–organic framework. This phenomenon called guest-driven light-induced spin change (GD-LISC) was initially studied using Hofmann-type metal–organic frameworks, which are well recognized for their SCO behavior accompanied by the influence of guest molecules upon the transition temperature, its cooperativity, or even the photomagnetic activity towards the LIESST effect.^288^ Firstly, the 3-D {[Fe^II^(bpac)][Pt^II^(CN)_4_]} (bpac = 1,2-bis(4-pyridyl)acetylene) network was loaded with photoisomerizable guest molecules, including (*E*)*-*azobenzene and (*E/Z*)-stilbene.^634^ Although the presence of the specific guest strongly modified both magnetic and photomagnetic properties originating from the presence of the SCO active Fe(II) complexes, the expected photoisomerization of guests in the solid state was quenched not only by steric hindrance and intermolecular interactions but also by the presence of a strong UV-light absorption of the samples, which limits the penetration depth. As a result, the photoconversion was considered negligible at room temperature, and the related impact of light upon the SCO phenomenon was not presented. However, shortly after this pioneering attempt, the first successful realization of the GD-LICS effect was presented by K.-P. Xie et al.^635^ Again, as a host matrix, a Hofmann-type framework was used, which was loaded with (*E*)-azobenzene molecules. The resulting system of {[Fe^II^(bpn)][Ag^I^(CN)_2_]_2_]}·(*E*)-azobenzene (bpn = 1,4-bis(4-pyridyl) naphthalene) reveals a complicated course of the thermal spin transition, where within the *χ*_M_*T*(*T*) curve four distinct SCO steps can be distinguished. Additionally, at the cryogenic region, the LIESST effect was observed; however, such low-temperature excitation does not affect the configuration of the azobenzene molecules. On the contrary, at room temperature, the UV and visible light alternatingly transform the configuration of guests between the *E* and *Z* forms, respectively. Such impact of room-temperature photochromism was studied by the UV–vis and IR absorption spectroscopies, as well as within SQUID magnetic measurements. In the case of the latter, the course of the *χ*_M_*T*(*T*) curve is significantly affected by the room-temperature irradiation, resulting in the presence of a residual Fe(II) HS fraction down to the cryogenic region. Then even after 9 days of room-temperature relaxation in the dark, the magnetic properties of the pristine system are not fully recovered. In contrast, irradiation of the UV-treated sample using the visible light induces a reverse transition towards the *E* configuration of the azobenzene species, which almost fully recovers the *χ*_M_*T*(*T*) curve of the as-synthesized material. Hereby presented bidirectional switching behavior of the SCO characteristics through the activity of guest molecules appears to be a promising pathway toward room-temperature spin state switching, which should be addressed in the future.

An alternative approach for modifying the SCO behavior by light in Hofmann-type networks was lately presented for {[Fe^II^(4-styrpy)_2_][Ag^I^(CN)_2_]_2_} (4-styrpy = 4-styrylpyridine) and {[Fe^II^(2,4-bpe)_2_][Ag^I^(CN)_2_]_2_} (2,4-bpe = *trans*-1-(2-pyridyl)-2-(4-pyridyl)ethylene).^636^ Both systems crystallize as coordination layers, while organic ligands from separate layers face each other due to the π–π interactions. For the first of them, single-step SCO behavior is observed, while the (2,4-bpe)-containing network reveals a two-step transition. Upon irradiation with a high-pressure mercury lamp for a few days, both systems undergo [2+2] photocycloaddition occurring within organic ligands in a single-crystal-to-single-crystal manner. For photoconverted materials, the SCO property is modified, thus for the initially (4-styrpy)-containing assembly the temperature of the spin transition shifts toward lower temperature, while for the second system, the HS to LS transformation is partially hampered and a monotonous drop of *χ*_M_*T* upon cooling is observed. Another example of SCO polymer showing photoswitchability at room temperature also involves the [2+2] photocycloaddition mechanism but of non-coordinated anions.^637^ The [Fe^II^(NH_2_trz)_3_](psca)_2_·2H_2_O (NH_2_trz = 4-amino-1,2,4-triazole; psca = *p*-sulfocinnamic acid) system is based on triazole-bridged coordination chains. At room temperature, the mixed spin-state is observed with a majority of low-spin Fe(II) centers. Upon UV light irradiation at room temperature, the pristine system shows transformation to achieve [Fe^II^(NH_2_trz)_3_](dsta)·2.5H_2_O (dsta = 4,4'-disulfonatetruxillic acid) composition. As a result, the high-spin fraction increases from 29% to 53%.

**Figure 28:**
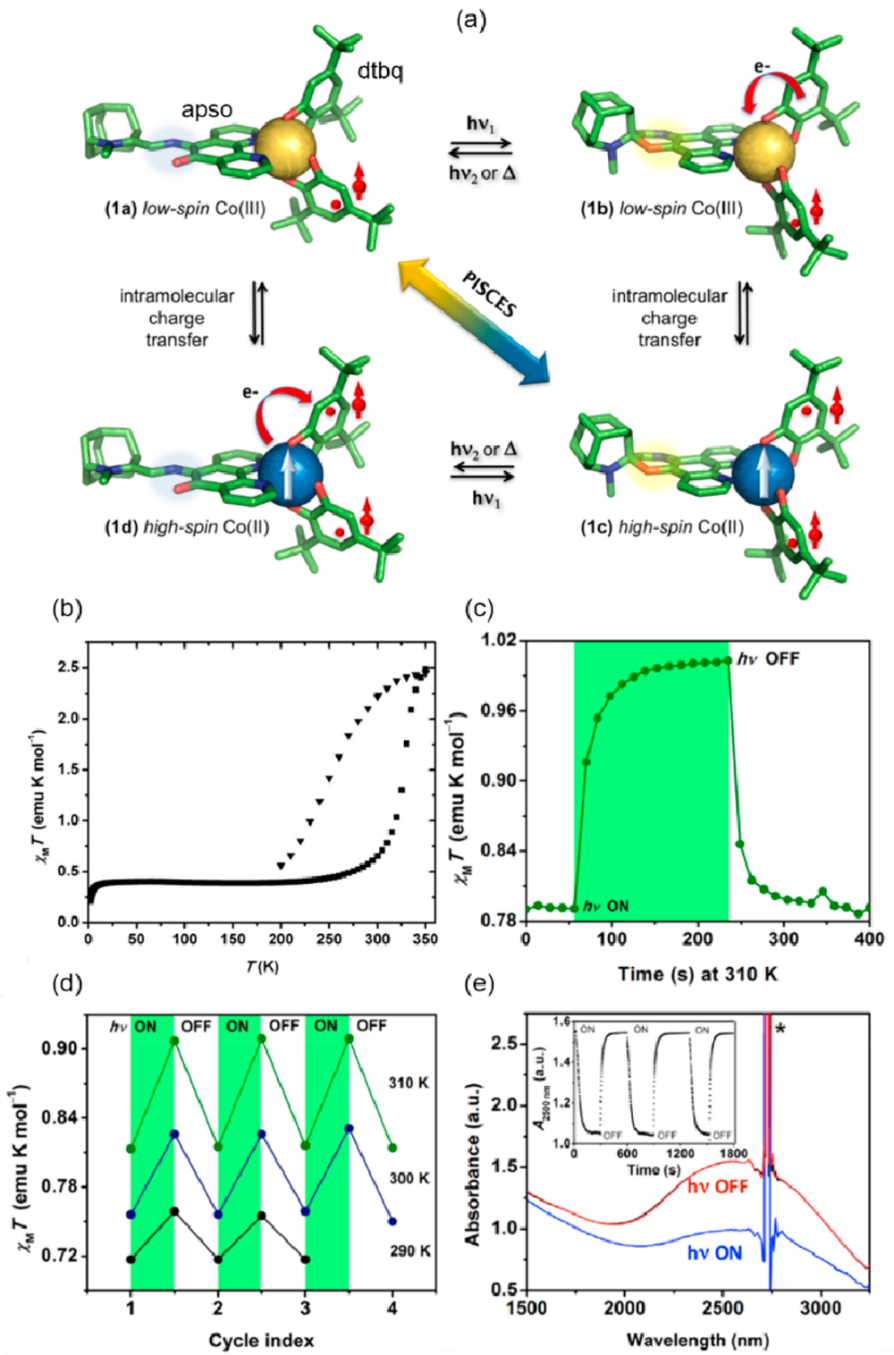
The schematic representation of photoisomerization-induced spin-charge excited-state (PISCES) effect in [Co^III^(dtbq)_2_(apso)] (dtbq = an *o*-benzoquinone derivative; apso = spiro(azahomoadamantyl-phenanthrolinoxazine)) metal complex (a), the temperature dependence of the *χ*_M_*T* product in the solid state (squares) and solution (triangles) (b), the time dependence of the *χ*_M_*T* product under visible light irradiation and its removal for the thin film sample (c), change of the *χ*_M_*T* product over several cycles of white light irradiation followed by thermal relaxation for the thin film sample (d), and the NIR absorption spectra before and after white light irradiation for the thin film sample at 300 K, presented together with the 2500 nm band intensity changes over the several cycles of white light on/off switching (e). Adapted with permission from ref (609). Copyright 2018 American Chemical Society.

Instead of using Fe(II) or Ni(II) spin state switching, photochromic organic ligands can be involved in the formation of Co(III) complexes revealing an electron transfer phenomenon.^609,638^ Such an idea was realized by N. L. Frank and co-workers by assembling spiro(azahomoadamantyl-phenanthrolinoxazine) ligand (apso), Co(II) centers, and an *o*-benzoquinone (dtbq, Figure 28).^609^ The open form of apso ligand favors the LS Co(III) state, thus the formation of a radical on one of the benzoquinone anions. On the other hand, the closed form, which can be generated by photocyclization, induces charge transfer between the second dtbq ligand and the metal center, resulting in the HS Co(II) state. At room temperature in a toluene solution, this system reveals thermal equilibrium between the open and closed form of the ligand and between LS Co(III) and HS Co(II) states. However, the crystallized system contains only LS Co(III) centers and apso ligand in its open form. Magnetic studies on the polycrystalline sample show stability of the LS Co(III) state below room temperature and a paramagnetic signal due to one semiquinonate (*S =* 1/2), while upon heating, at *T*_1/2_ = 325 K, an increase of the *χ*_M_*T* value appears due to formation of HS Co(II) centers (*S* = 3/2) together with the generation of a second semiquinonate ligand. At 350 K, the electron transfer-coupled spin transition does not reach full completeness. The prepared ca. 300 nm thin film samples reveal next to room temperature distinct photomagnetic effect under 550 nm excitation. Then after switching off the light source, thermal relaxation occurs exponentially with a ca. 10 s half-life of the photogenerated species at 310 K. The resulting photoisomerization-induced spin-charge excited state (PISCES) process also induces a change in the optical absorption spectra, which were followed for the thin film in the NIR region at 300 K. Light irradiation decreases ca. 2500 nm band intensity related to CT states. Both magnetic and optical data show reversibility of the PISCES effect.^609^

Ligand-driven switching of electron transfer can be also obtained using a 4-styrylpyridine ligand. For a [Co^III^(dtbq)_2_(*E*-4-styrpy)_2_] (4-styrpy = 4-styrylpyridine) complex in toluene solution gradual electron transfer appears upon cooling with *T*_1/2_ = 299 K, while a modified course with *T*_1/2_ = 287 K is found for the [Co^III^(dtbq)_2_(*Z*-4-styrpy)_2_] system.^639^ The presence of an electron transfer process between Co(III) and benzoquinone derivative can be followed either by the Evans method or electronic absorption spectra. Upon 320 nm light irradiation of toluene solution with a complex containing *E*-4-styrpy at room temperature, an increase of the 750 nm band absorption and decrease of the 2830 nm one is observed, while the magnetic moment increases at the same time. Reverse photoswitching for the solution of *Z*-(4-styrpy)-containing complex is achieved using 272 nm light, resulting in a decreased magnetic moment. Therefore, two-way switching is presented. On the other hand, a photochromic spiropyran-based cation (SP) was incorporated into the [Mn^II^Cr^III^(ox)^_3_^]_n_^*n*–^ ferromagnetic layers, which resulted in the modification of a magnetic hysteresis loop upon 355 nm irradiation in the solid state.^640^

#### 3.3.2 Molecular Nanomagnets Incorporating Photochromic Ligands

Several successful attempts were reported on the introduction of organic photochromic ligands for the construction of molecular nanomagnets. Single-molecule magnets, exploring the magnetic anisotropy of metal centers, require a large degree of photoconversion to change their properties originating mainly from their detailed molecular structure. For this reason, the most recognized photosensitive ligands employed for their construction belong to the family of diarylethenes which can be reversibly switched between their open and closed forms alternatively by UV and visible light irradiation, respectively. As a source of distinct SMM behavior both anisotropic cluster systems, as well as monometallic complexes of lanthanide ions were employed. For instance, the mixed valence system, {[Mn^II/III^_4_(hmp)_6_(dae-c)_2_(H_2_O)_2_]} (ClO_4_)_2_·MeCN·4H_2_O (Hhmp = 2-hydroxymethylpyridine; H_2_dae-c = closed form of 1,2-bis(5-carboxyl-2-methyl-3-thienyl)perfluorocyclopentene), built of [Mn^II^_2_Mn^III^_2_] clusters, upon visible light irradiation at room temperature in the solid state, undergo reversible photochromism.^641^ Due to the dae^2–^ ring opening, the course of the *χ*_M_*T*(*T*) is significantly modified, as well as the shape of the *M*(*H*) butterfly-shaped hysteresis loop at 0.5 K originating from the SMM behavior is amended. Next, the {[Mn^III^(salen)(MeOH)]_2_(dae-c)]}·2MeOH (H_2_salen = 2,2′-ethylenebis(nitrilomethylidene)phenol) assembly shows the off/on switching of the SMM behavior upon alternating visible and UV light irradiation in the solid state.^642^ However, as shown for the {[Mn^III^_2_(saltmen)_2_(dae-o)]} (H_2_saltmen = 2,2′-((1*E*,1′*E*)-((2,3-dimethylbutane-2,3-diyl)bis(azaneylylidene))bis(methaneylylidene))diphenol) and the {[Mn^III^(saltmen)(dae-c)]}·H_2_O·Et_3_N systems, although magnetic properties can vary significantly between SMMs obtained with different ligand photoisomers, the effect of visible or UV light irradiation, respectively, can be negligible.^643^ The dae^2–^ ligands were also used for linking the {[Cu^II^Tb^III^]} units.^644^ Completely different coordination systems were obtained by reacting {[Cu^II^Tb^III^(L_bmsap_)(NO_3_)_3_]} (H_2_L_bsmap_ = 1,3-bis((3-methoxysalicylidene)amino)propane) precursor with opened and closed form of a photochromic bridge. For the open form, tetranuclear molecules are obtained, while the use of H_2_dae-c gives a coordination polymer composed of one-dimensional ladders. Upon irradiation, both systems reveal a photochromic effect, but only for one-dimensional ladders the slow relaxation of magnetization is changed due to the ring opening.

**Figure 29:**
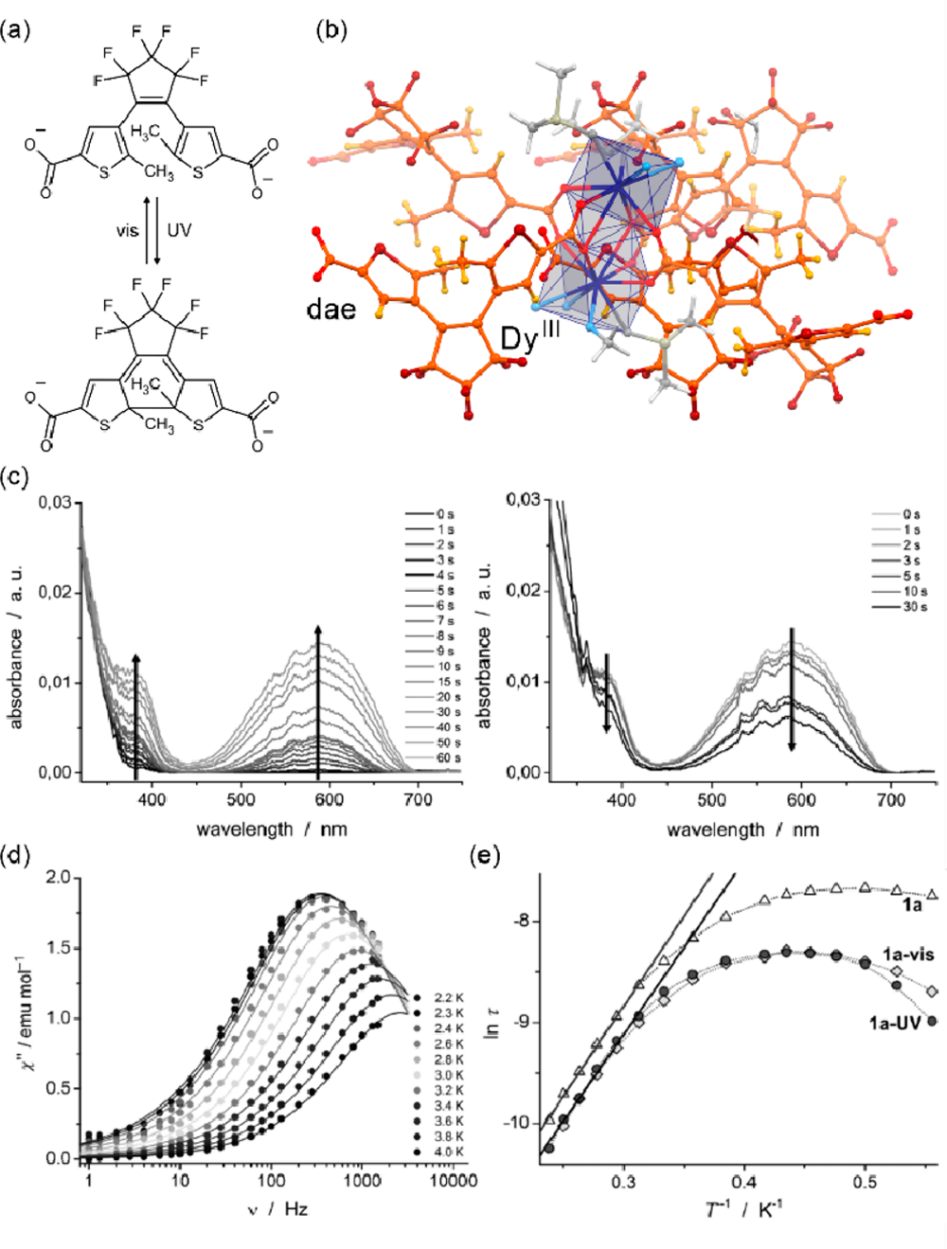
The structure of the 1,2-bis(5-carboxyl-2-methyl-3-thienyl)perfluorocyclopentene (dae^2–^) ligand and its schematic conversion under UV and vis light irradiation (a), the structure of {[Dy^III^_2_(dae)_3_(dmso)_3_(MeOH)]} dinuclear molecule (b),^646^ the solid-state UV–vis absorption spectra of a powdered sample measured upon irradiation using the 365 nm (left side) and 480 nm (right side) light (c), temperature variable frequency dependencies of the imaginary part of the *ac* magnetic susceptibility before irradiation, under 1500 Oe *dc* magnetic field (d), the temperature dependencies of the relaxation times under 1500 Oe *dc* magnetic field for the pristine sample, after irradiation with the 365 nm light, and after irradiation with the 480 nm light (e). Parts (a), (c), (d), and (e) were adapted with permission from ref (646). Copyright 2014 John Wiley & Sons.

By combining Dy(III) or Ho(III) centers with dae^2–^ in its opened form and 2,2′-bipyridine (2,2′-bpy) as chelating ligands, {[Ln^III^_2_(dae-o)_3_(2,2′-bpy)_2_(H_2_O)_2_]}·2EtOH·4H_2_O molecular systems were obtained.^645^ The Dy^III^-based analog reveals slow relaxation of the magnetization effect under the zero *dc* field up to ca. 4 K. However, after irradiation with UV light, the relaxation process at zero *dc* field moves toward higher frequencies, therefore relaxation time could only be followed under the applied field of 1 kOe. The effective energy barrier for magnetization reversal under the external field changes from 21.7 K to 17.1 K, thus suggesting the main role of photoinduced ring closure in modifying the quantum tunneling of magnetization. A similar molecular system, {[Dy^III^_2_(dae-o)_3_(dmso)_3_(MeOH)]}·10MeOH reveals changes within solid-state electronic absorption spectra at room-temperature upon 365 nm light irradiation (Figure 29).^646^ The appearance of the band centered at ca. 590 nm, and the one at ca. 390 nm proves the ring closure in solid state upon irradiation. Further use of 480 nm light reduces their intensity, showing some degree of photoreversibility. The as-synthetized system with the open form of dae^2–^ ligand shows SMM behavior under an applied magnetic field of 1.5 kOe with the energy barrier of 14.2 K. After ring closure the relaxation accelerates but the energy barrier slightly increases to 14.7 K. Then visible light irradiation further modifies slow relaxation dynamics. Although the energy barrier drops down to its original value, the relaxation times remain reduced within the whole temperature range.^646^

Both the above-mentioned systems suggest that the activity of dae^2–^ moiety has little influence on the energy barrier of the spin reversal. The main influence of the ring closure/opening may be found within the Raman two-phonon relaxation or in the rate of the QTM effect. This is especially true for Ln(III) molecular species, where the magnetic relaxation effects are governed by the numerous relaxation mechanisms. Therefore, when searching for large changes in the SMM behavior, one should focus on Ln(III) complexes with large anisotropy, resulting in a magnetic hysteresis of molecular origin. Then not only the shape of the hysteresis may be affected by the modification of the relaxation rates but also the completeness of the photoirradiation process will be less crucial to prove the influence of light upon the magnetic properties.

**Figure 30:**
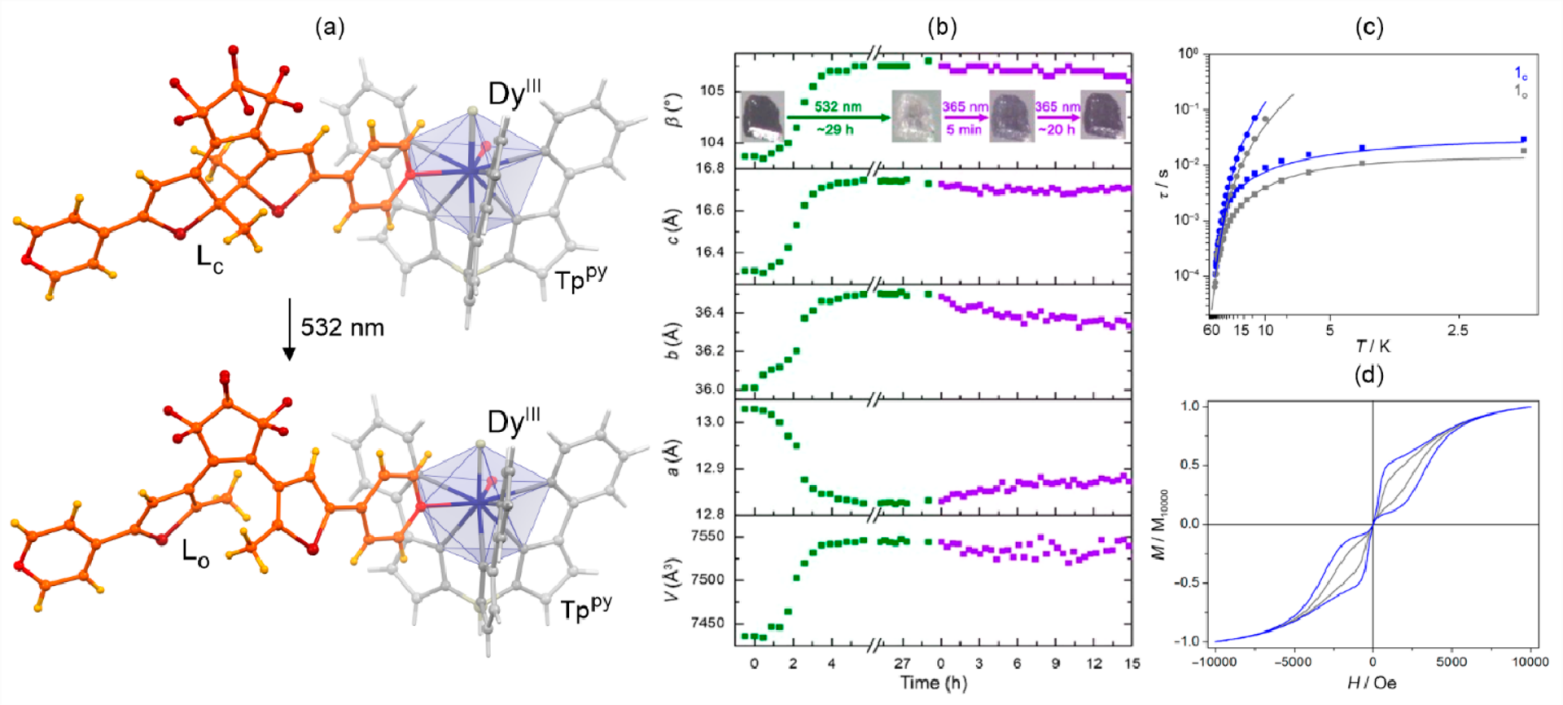
The structure of [Dy^III^F(L_c_)(Tp^py^)]PF_6_ (L_c_ = closed form of bispyridyl dithienylethene derivative; Tp^py^ = tris(3-(2-pyridyl)pyrazolyl)hydroborate) coordination chain and its photoinduced phase with the open form of the bispyridyl dithienylethene ligand (L_o_) (a),^647^ the time evolution of the unit cell parameters upon the 532 nm light irradiation followed by the 365 nm light irradiation at room temperature (b), the temperature dependences of the magnetic relaxation time for this system before and after white light irradiation (c) and the magnetization versus field hysteresis loops at 2 K before and after white light irradiation (d). Parts (b), (c), and (d) were adapted with permission from ref (647). Copyright 2019 American Chemical Society.

Therefore, to obtain large changes in the SMM behavior upon light irradiation, dithienylethene-type ligands were combined with highly-anisotropic [Dy^III^F(Tp^py^)]^+^ (Tp^py^ = tris(3-(2-pyridyl)pyrazolyl)hydroborate) complexes (Figure 30).^647^ The resulting [Dy^III^F(L_c_)(Tp^py^)]PF_6_ (L_c_ = a closed form of bis(2-methyl-5-(4-pydidyl)-3-thienyl)perfluorocyclopentene) coordination chain retains the magnetically axial character of Dy(III) centers originating from the presence of a short Dy–F bond. Upon the visible light irradiation (*λ* = 532 nm), a single-crystal to single-crystal manner of a photoinduced ligand opening occurs. The conversion of the system with the opened form of the ligand toward the closed one can be followed by tracing crystallographic parameters. Single-crystal X-ray analysis also suggests the inability of the photoirradiated system to return to the initial state by UV light irradiation. Both for the closed and the opened forms of dithienylethene ligands, Dy(III) complexes show SMM behavior at zero *dc* field. Upon visible light irradiation, a butterfly-shaped hysteresis loop at 2 K shrinks but does not completely disappear. The temperature dependence of the relaxation time suggests that upon irradiation, the value of the energy barrier for the Orbach process remains stable, while as previously, the changes of quantum tunneling of magnetization influence relaxation dynamics, accompanied by modification of phonon modes involved in the Raman relaxation process.^647^

Upon modification of a photoactive ligand involving the position of N-donor atoms of the pyridine rings towards the 1,2-bis(2-methyl-5-(3-pyridyl)-3-thienyl)perfluorocyclopentene (^3^L_c_), the dimensionality of the system with [Dy^III^F(Tp^py^)]^+^ units is reduced to binuclear molecules.^648^ The resulting {[Dy^III^F(Tp^py^)]_2_(^3^L_c_)_2_}(BArF)_2_ (BArF = tetrakis[3,5-bis(trifluoromethyl)phenyl]borate anion) compound shows photochromism upon visible light irradiation and as its former family member reveals a single-crystal to single-crystal manner of photoinduced transformation. The reverse transformation results in the breaking of the crystals but can be followed using electronic absorption spectroscopy. Visible light irradiation modifies the SMM behavior originating from anisotropic Dy(III) complexes. However, as for the previous case, the ring opening results in faster QTM, here the trend is the opposite. By bridging low-coordinate Er(III) complexes with L_o_ (an open form of bis(2-methyl-5-(4-pydidyl)-3-thienyl)perfluorocyclopentene) ligands into {[Er^III^(BHT)_3_]_3_(L_o_)_2_}·4C_5_H_12_ (BHT = 2,6-di-tert-butyl-4-methylphenolate) coordination chains, reversible photochromic behavior was observed upon alternating UV and visible light irradiation.^649^ Field-induced SMM behavior was observed for both forms of L_o_/L_c_ ligands, with modified characteristics, as exemplified by changes within the field dependencies of magnetic relaxation time.

Photochromic properties of azobenzene ligands were also tested for modulation of SMM characteristics. Among them, the two {Fe^III^}_4_ magnetic clusters, functionalized by different azobenzene derivatives, {[Fe^III^_4_(azo)_2_(dpm)_6_]} and {[Fe^III^_4_(azoMe_2_)_2_(dpm)_6_]} (H_3_azo = 2-(hydroxymethyl)-2-(4-(phenyldiazenyl)phenyl)propane-1,3-diol; H_3_azoMe_2_ = 2-(4-((3,5-di-methylphenyl)diazenyl)phenyl)-2-(hydroxymethyl)propane-1,3-diol; Hdpm = dipivaloylmethane) show reversible changes of their electronic absorption spectra in toluene solution upon alternating 365 nm and visible light irradiation.^650^ Both systems reveal distinct SMM behavior under the applied field of 1 kOe. The {[Fe^III^_4_(azoMe_2_)_2_(dpm)_6_]} system, when dispersed in a polystyrene matrix, reveals subtle but reversible changes in the SMM characteristics. A similar character of photoswitching zero field SMM behavior was presented within a family of {Dy^III^_12_} clusters functionalized by four azobenzene derivatives of salicylic acid.^651^ The molecular system built with the support of 2-hydroxy-5-(*p*-tolyldiazenyl)benzoic acid reveals slow relaxation of magnetization up to 7 K within the frequency region limited to 1–1000 Hz. After 365 nm light irradiation at room temperature, the respective values of relaxation times decrease resulting in distinct SMM behavior up to 5 K. Photoisomerization of the double bond was also employed for modification of SMM behavior of the [Dy^III^(H_2_O)(MC)(OTf)_2_] (MC = chelating merocyanine) complexes dissolved in acetonitrile solution.^652^ As for the {Dy^III^_12_} clusters, the mononuclear system reveals deteriorated relaxation dynamics after irradiation.

**Figure 31:**
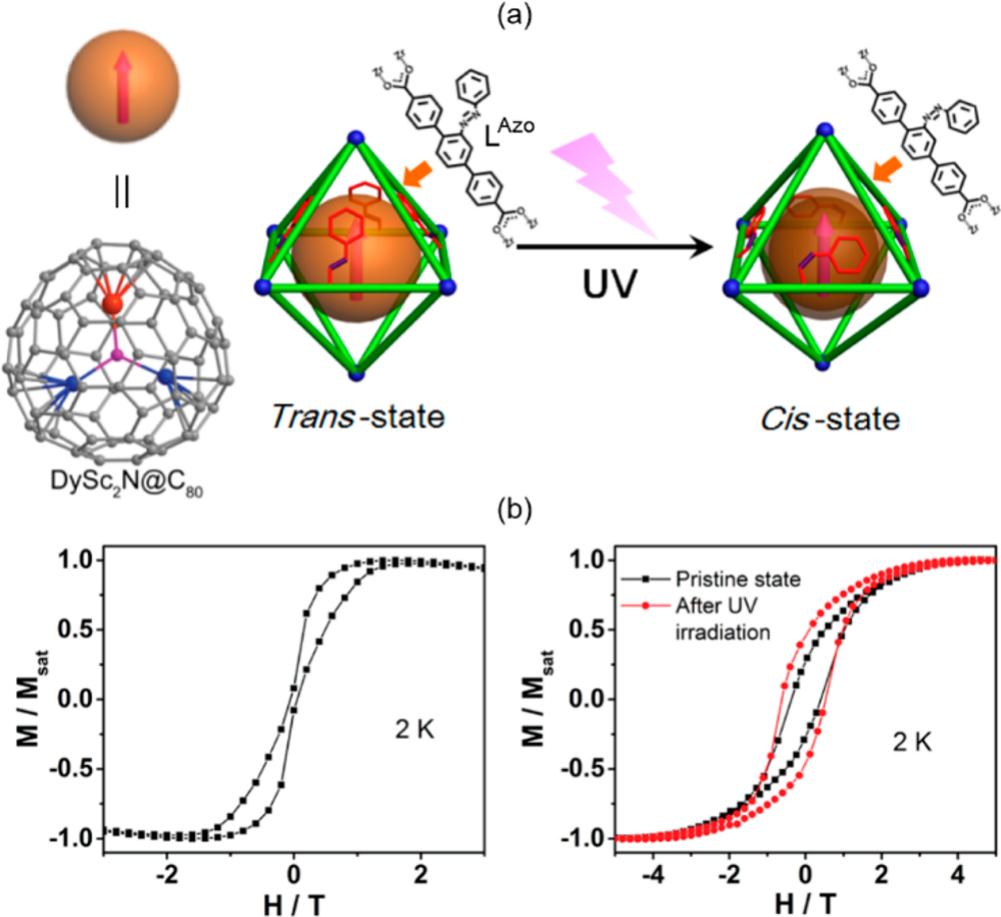
Schematic representation of the ^Azo^MOF, {[Zr^IV^_6_O_4_(OH)_4_(L^Azo^)_6_]} (L^Azo^ = 2′-phenyldiazenyl-1,1′:4′,1″-terphenyl-4,4″-dicarboxylate) with the octahedral pores occupied by {Dy^III^Sc^III^_2_N@C_80_} magnetic fullerenes, and its irradiation with the UV light (a),^654,655^ the related magnetization versus field hysteresis loops at 2 K for {Dy^III^Sc^III^_2_N@C_80_} (b, left) and for this system embedded in the ^Azo^MOF before and after UV light irradiation (b, right). Adapted with permission from ref (655). Copyright 2018 American Chemical Society.

A non-trivial method of photoswitching the magnetic relaxation was presented by T. Wang, C. Wang, and co-workers by introducing {Dy^III^Sc^III^_2_N@C_80_} metallofullerene single-molecule magnets into metal–organic frameworks (Figure 31).^653−655^ Such encapsulation itself modifies SMM characteristics of the pristine materials, especially the shape of the hysteresis loop of a single-ion origin. Proper selection of the MOF matrix enables the introduction of additional features, such as photoresponsivity.^655^ Therefore, Zr(IV) centers and an *E*-2′-benzyldiazenyl-1,1′:4,4′-terphenyl-4,4″-dicarboxylic acid were combined to synthesize the ^Azo^MOF, which contains both small tetrahedral and large octahedral pores.^654^ Upon its immersion in a toluene solution containing {Dy^III^Sc^III^_2_N@C_80_}, metallofullerene is encapsulated into octahedral pores through the formation of π–π interactions. The MOF material both before and after post-synthetic modification shows changes of optical absorption properties upon UV light irradiation at room temperature due to the *E* to *Z* photoisomerization. The pristine system with encapsulated SMMs reveals the *M*(*H*) hysteresis loop at 2 K with a partially quenched QTM effect at zero *dc* field in comparison to the pure {Dy^III^Sc^III^_2_N@C_80_} sample.^656^ Irradiation with UV light at 2 K results in modification of magnetization versus field hysteresis loop, including increased remnant magnetization and coercive field. Such behavior suggests a further reduction of QTM.

Another group of photoswitchable molecular nanomagnets combines lanthanide(III) centers and phosphonate ligands with 9-anthryl substituents which can reveal photocycloaddition in the solid state upon UV light irradiation when involved in π–π interactions with each other in the crystal structure of as-synthesized material.^657−660^ Most of such systems are built of isolated Dy(III) centers and combine slow relaxation of magnetization effect with luminescence properties, thus they will be precisely described in section 7. regarding expanded optical multifunctionality of molecule-based magnetic materials. In this regard, also other strategies toward photoswitchable Ln^III^-based luminescent SMMs were presented which include the introduction of photoactive luminescent 2,4,6-tri(4-pyridyl)-1,3,5-triazine guests into the crystal lattice containing Dy(III) SMMs or the coordination of phosphotungstate ions, showing light-induced electron transfer, to the magnetic Dy(III) centers (see section 7 for details).^661,662^

## 4 Luminescence in Molecule-Based Magnetic Materials

Specific physicochemical properties of luminescent materials, related to their ability to emit light due to the absorption of photons, chemical reaction, exposure to electric current, mechanical action, or ionizing radiation, as well as the sensitivity of this property to external stimuli, are the key elements for technological development.^663−668^ These materials arouse an enormous scientific interest due to their diverse applications for cathode-ray or fluorescent tubes,^669^ chemical sensing,^670^ anticounterfeiting,^671^ display devices,^672^ optical communication,^673^ energy conversion,^674^ optical storage,^675^ photovoltaics,^676^ photonics,^677^ biological imaging,^678^ and molecular thermometry.^679^ In many of these areas, for instance, in the context of light-emitting diodes (LEDs), organic photovoltaics, and fluorescent probes for diagnostics, the progress is really impressive making the exploration of luminescence of new materials a multidisciplinary field desired in various, sometimes very different branches of science and technology.^670−679^

Two kinds of commonly exploited building blocks used to construct luminescent materials are organic chromophores and metal ions, the latter often embedded in organometallic or coordination complexes.^663,677,680^ Many advantages can be assigned to both groups; however, a few drawbacks are usually ascribed to high-performance luminescent organic chromophores, which include the complexity of synthetic procedures generating high costs, relatively low Stokes shifts, broad emission bands, and relatively short emission lifetimes. Some of these features can be overcome by judicious synthetic approaches; however, as an attractive alternative, luminescent materials based on metal ions and their complexes were broadly recognized.^672,677,678,681^ Among them, metal complexes were found promising prerequisites, especially when considering their ability to tune the structure and properties using a molecular building blocks approach, as well as their great sensitivity to external stimuli, which is an important point towards applications, e.g., in sensors of chemical and physical stimuli.^682^ Thanks to these characteristics, luminescent materials based on metal complexes, ranging from discrete molecules to coordination polymers (CPs), including metal–organic frameworks (MOFs), are intensively studied as functional solid luminophores offering multiple optical functionalities, such as white-light emission,^683,684^ multicolored tunable visible emission,^685^ long-lived near-infrared phosphorescence,^686,687^ and the nonlinear optical property of up-conversion luminescence (UCL).^178,688^ They are efficiently realized by the photo- and electroluminescent materials, the most attractive light-emitting phenomena for the application aspect.^670−679^ Moreover, luminescent materials based on metal complexes are excellent platforms for the construction of multifunctional molecular systems, for which light emission property is combined with porosity, ferroelectricity, ionic conductivity, catalytic activity, and many others.^689−694^ This section will discuss the advances in combining luminescent properties with magnetic effects in molecular materials, mentioning, more generally, all the aspects of molecular magnetism where the luminescent signal from a metal complex became an important issue. The following parts of this section will include the design of luminescent magnetically ordered phases (4.1), luminescent spin transition materials (4.2), light-emitting molecular nanomagnets (4.3), as well as more specialized hot research topics, including luminescent thermometers based on single-molecule magnets (4.4), valuable magneto-optical correlations in luminescent molecular nanomagnets (4.5), magnetic field control over emission (4.6), and the exploration of a luminescent signal in molecular qubit systems (4.7).

### 4.1 Design of Luminescent Molecule-Based Magnets

In the last two decades, the idea of the implementation of luminescent functionality into magnetic nanoparticles has been of great interest. The combination of light emission and magnetic properties in one nanometric composite material gives the perspective for simultaneous biolabeling, optical or magnetic imaging, and cell sorting/separation, enormously important from a diagnostic and therapeutic point of view.^695^ Such luminescent magnetic nanocomposites are built of fluorescent quantum dots bonded with particles of magnetic materials, and their emission and magnetism originate from two independent components.^696^ As an alternative, luminescent molecule-based magnets, constructed of metal complexes providing both magnetic coupling and effective luminescence, enable the detection of a variety of luminescence effects in magnetic systems, and the coupling between these properties within one single-phase material. Most of the d-block metal ions, commonly used for molecule-based magnets, are not emissive due to the nonradiative relaxation through interactions with low-lying excited states.^697,698^ This causes difficulties in the design and synthesis of emissive magnets, and because of the lack of systematic work, it is difficult to assess the approach affecting the enhancement of both properties in one material, or their coupling. Despite this, they are realized mainly by three synthetic strategies, described by examples in the following paragraphs.

**Figure 32:**
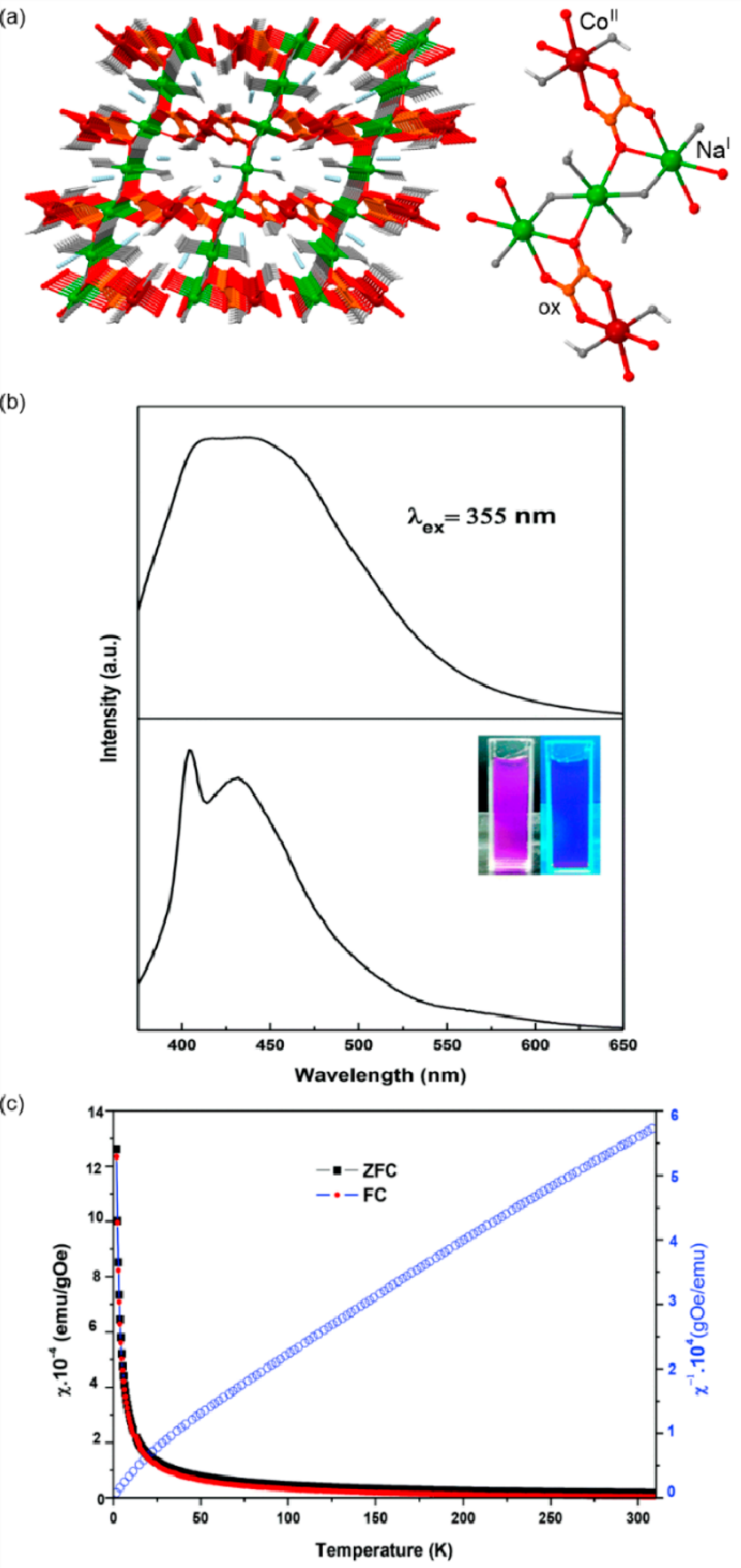
The representative structural views on {[Na^I^(H_2_O)_2_]_2_[Co^II^(ox)_2_(H_2_O)_2_]}·2H_2_O (ox = oxalato) coordination framework (a),^703^ its room-temperature photoluminescence spectra for the UV excitation in the aqueous solution (top, b) and in the solid state (bottom, b), and the set of *dc* magnetic characteristics, including the *χ*_M_(*T*) and *χ*_M_*T*(*T*) curves under the applied field of 1 kOe (c). Parts (b) and (c) were reproduced from ref (703) with permission from the Royal Society of Chemistry.

The first strategy employs molecular luminophores only coexisting in a supramolecular network with a magnetic system.^234,699^ Such an approach was successfully used for the synthesis of two-dimensional anionic oxamato-bridged Co^II^–Cu^II^ ferrimagnetic layers (*T*_c_ = 19 K) with emissive [Ru^II^(2,2′-bpy)_3_]^2+^ (2,2′-bpy = 2,2′-bipyridine) cations inserted between them.^234^ At room temperature, the luminescence of the Ru(II) chromophore is largely quenched by the bimetallic polymer, however, as the temperature is lowered, the emission intensity increases. It reaches a maximum around critical temperature and decreases as the temperature is further lowered. The synergy between magnetic and optical properties in this compound is due to the large internal field created in the magnetically ordered state, which perturbates the luminescent states of the counter-ionic fluorophore. A similar approach was used by J. Ferrando-Soria et al. in the preparation of layered Mn(II)–Cu(II) anionic MOF, [Mn^II^_2_Cu^II^_3_(mpba)_3_(H_2_O)_3_]^2–^ (mpba = *N*,*N*′-1,3-phenylenebis(oxamate) anion), with a methylviologen cationic dye.^699^ By combining its emitting properties with the ferromagnetic (*T*_c_ = 19 K) properties of the open bimetallic framework with shape-selective sorption behavior, this material allows the identification of small molecules and observation of the host-guest interactions. The second approach concerns the use of a diamagnetic luminescent species in the construction of a magnetic system.^700−706^ It was shown in a small group of coordination polymers, built of carboxylate-bridged Co(II)- or Ni(II)-based networks showing magnetic coupling and the emission property originating from a bridging organic ligand. A special family of materials in this group are metal oxalato complexes with alkali cations, {A^I^_2_[M^II^(C_2_O_4_)_2_(H_2_O)_2_]}·solvent (A = Na, K; M = Co, Ni), based on oxalato-bridged anionic chains (Figure 32).^703−706^ They exhibit antiferromagnetic interactions between metal ions, and the UV-to-vis luminescence attributed to ligand-to-metal charge transfer states.

**Figure 33:**
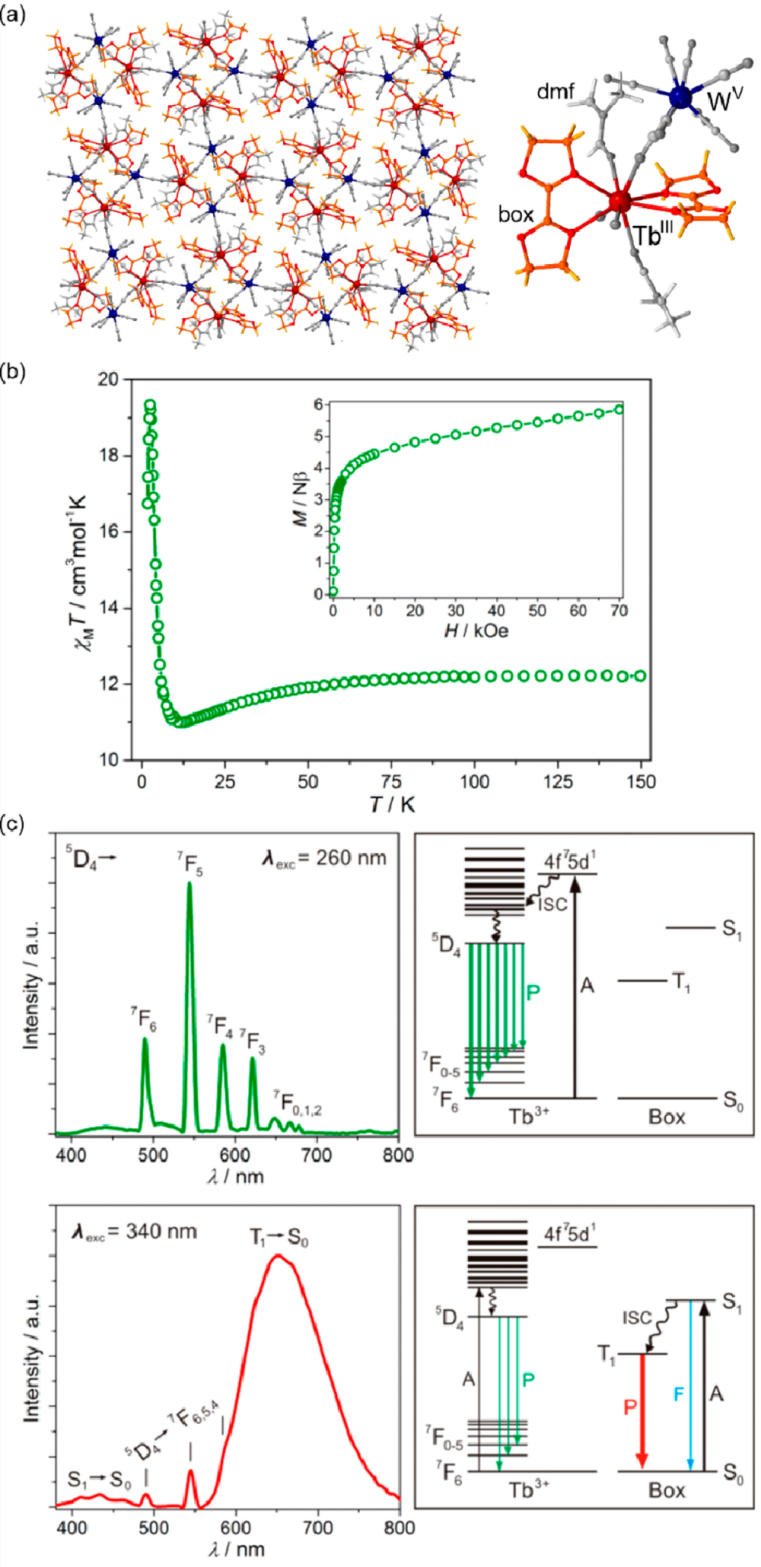
The representative structural views on {[Tb^III^(box)_2_(dmf)_2_][W^V^(CN)_8_]}·H_2_O (box = 2,2′-bis(2-oxazoline)) layered coordination network (a),^716^ its temperature dependence of the *χ*_M_*T* product under 2 kOe (b), shown together with the field dependence of magnetization at 1.8 K (the inset), and the low-temperature emission spectra under the indicated excitation light conditions, presented together with the related schematic energy level diagrams representing the observed optical phenomena (c). Parts (b) and (c) were adapted with permission from ref (716). Copyright 2014 American Chemical Society.

The third strategy employs metal centers which are both paramagnetic and luminescent. This approach was presented for d–f bimetallic coordination polymers, containing emissive and paramagnetic lanthanide(3+) ions bonded to longer organic molecular bridges or shorter cyanido linkages.^707−716^ The first group includes several examples of heterometallic coordination systems, consisting of Cu(II) centers surrounded by a macrocyclic oxamido ligand and combined with lanthanide(3+) ions (Pr, Nd, Sm, Yb) into one- and two-dimensional bimetallic coordination polymers, further connected also with 5-nitroisophthalate ligands into a three-dimensional coordination network.^717,718^ Within these materials, metal ions interact together antiferromagnetically while the Cu^II^–(macrocyclic oxamido) metalloligand serves as a sensitizer for the near-infrared luminescence of 4f metal ions. A series of magneto-luminescent coordination frameworks based on two paramagnetic molecular building blocks, i.e., 4f metal complexes and polycyanidometallate ions, [Cr^III^(CN)_6_]^3–^ and [M^V^(CN)_8_]^3–^ (M = Mo, W) have been also reported.^709−716^ The visible emission, originating from f–f electronic transition of Eu^3+^ and Tb^3+^ ions, was found in the series of {[Ln^III^(H_2_O)_5_][M^V^(CN)_8_]} (Ln = Eu, Tb, or mixed Sm/Tb, Eu/Gd, Eu/Tb, and Sm/Tb metal compositions; M = Mo, W) layered cyanido-bridged networks, exhibiting also intermetallic magnetic coupling leading to long-range magnetic ordering at low temperatures below 3 K.^709−711^ Later, several systems with additional organic ligands coordinated to lanthanide ions were developed, such as NIR-luminescent bimetallic Nd^III^(1,10-phenanthroline)–Mo^V^/W^V^ chains bearing ferromagnetic coupling,^712^ or chiral helices involving Nd^III^–W^V^ and Gd^III^–W^V^ metal pairs and 2,6-bis[4-isopropyl-2-oxazolin-2-yl]pyridine ligands that reveal the conjunction of ferromagnetic coupling with Nd^III^-based NIR emission for the first metals pair and antiferromagnetic coupling with red ligand-based phosphorescence for the second case.^713^ In the other example from this group, a layered Nd^III^(pyrimidine *N*-oxide)–Cr^III^ framework was reported to exhibit the combination of a long-range ferromagnetic ordering below 2.8 K and 4f-metal-based NIR emission sensitized by [Cr^III^(CN)_6_]^3–^ ions.^714^ The broad magneto-luminescent bifunctionality was found for the family of layered {[Ln^III^(box)_*n*_(dmf)_*m*_][M^V^(CN)_8_]}·*x*(solvent) (Ln = Ce–Yb; M = Mo, W; box = 2,2′-bis(2-oxazoline) ligand) networks.^715,716^ Among them, the Tb^III^–W^V^ analog combines ferromagnetism with visible luminescence, the color of which can be switched between Tb^III^-centered green and ligand-based red emission by excitation light (Figure 33).^716^ Considering all members from this family of compounds, the combination of the Ln^III^ centers with box ligands and [M^V^(CN)_8_]^3–^ ions resulted in magnetic interactions and spin ordering coexisting with various emission properties coming from the interaction between 4f metal ions and ligand excited states. Even the relatively rare Ho^III^- and Pr^III^-based emission, especially among molecular materials, could be observed together with intralayer magnetic coupling for this class of materials.^715^

In the field of photoluminescent materials, increased attention is devoted to the emissive materials based on d-block metal ions, such as Mn(II)-based complexes. From the viewpoint of magnetic properties, their high spin ground state (*S* = 5/2) is suitable for the construction of cryocoolers based on the magnetocaloric effect (MCE), but their tunable red emission of the d–d origin is parity forbidden and only appears for complexes with a partially reduced symmetry. In these contexts, the idea of constructing a luminescent MCE material was tested by W. Huang et al.^717^ For this purpose, the Mn^2+^ ions were combined with Gd(III) centers into bimetallic heptanuclear clusters, [Gd^III^_5_Mn^II^_2_(L^OMe^)_2_(OH)_4_(Ac)_6_(MeOH)_10_Cl_2_]Cl_3_·2MeOH (H_2_L^OMe^ = 1,2-bis(2-hydroxy-3-methoxybenzylidene)hydrazine; Ac = acetate). The presence of Mn(II) centers results in red phosphorescence but the emission of the material can be also induced by the application of mechanical stress. Moreover, both the temperature- and pressure-induced changes in the photoluminescence spectra were studied for this system. Upon lowering the temperature, luminescence quantum yield firstly decreases, reaches a plateau at 250 K, and then increases five times down to liquid nitrogen temperature. On the other hand, the increase of pressure at room temperature results in a subtle red shift of the emission, which is accompanied by the weakening of the emission intensity down to its disappearance under 15 MPa. Aiming at the photoluminescent MCE material, the *dc* magnetic properties were studied for this cluster-based system. According to the experimental magnetization data at 2 K, the critical value of the entropy change upon increasing the field to 70 kG, −Δ*S*_m_, reaches 35 J kg^–1^ K^–1^, which was attributed to the large metal-to-ligand mass ratio.^717^ The conjunction of temperature-variable luminescence and magneto-caloric effect is promising from the point of magnetic coolers with the optical readout of the obtained temperature, therefore, the family of luminescent MCE materials should be extended soon, prompted by such initial results in this topic.

### 4.2 Design of Luminescent Spin Transition Materials

Spin transition materials, including those revealing a spin crossover (SCO) effect between the high spin (HS) and the low spin (LS) state of a metal complex, e.g., Fe^II^ (see section 2.1), which exhibit luminescent properties would present an additional, i.e., emission-based, spin-state “read out” possibility. Tracking the HS/LS population during the SCO transition by measuring the relative intensity of the luminescence signal, or conversely, modulation of luminescence by controlling the spin state population are attractive features that can facilitate the integration of spin crossover materials in real devices.^288,718^ The design of luminescent materials exhibiting spin transitions is therefore extremely promising when taking into account their potential applications, e.g., in magneto-optical switches,^267^ nanothermometers,^719^ or a new generation of multifunctional sensors.^101,324,718^ Until now, two main strategies for the construction of such materials have been adopted. The first one is to build a bifunctional composite material containing luminescent and SCO-active components.^720−728^ The second one is to bind a luminophore to a metal center, undergoing the spin transition effect, within a single-phase molecule-based material.^729−738^ Moreover, considering the way by which the luminophore is integrated into the SCO material, the first class can be classified into three sub-groups: (i) a composite material made of luminescent material and SCO compound belonging to separate layers,^720,721^ (ii) functionalized (SCO-active-core)–(luminescent-shell) nanoparticles,^722−726^ and (iii) SCO complexes and their nanomaterials mixed with fluorescent species.^721,727,728^

**Figure 34:**
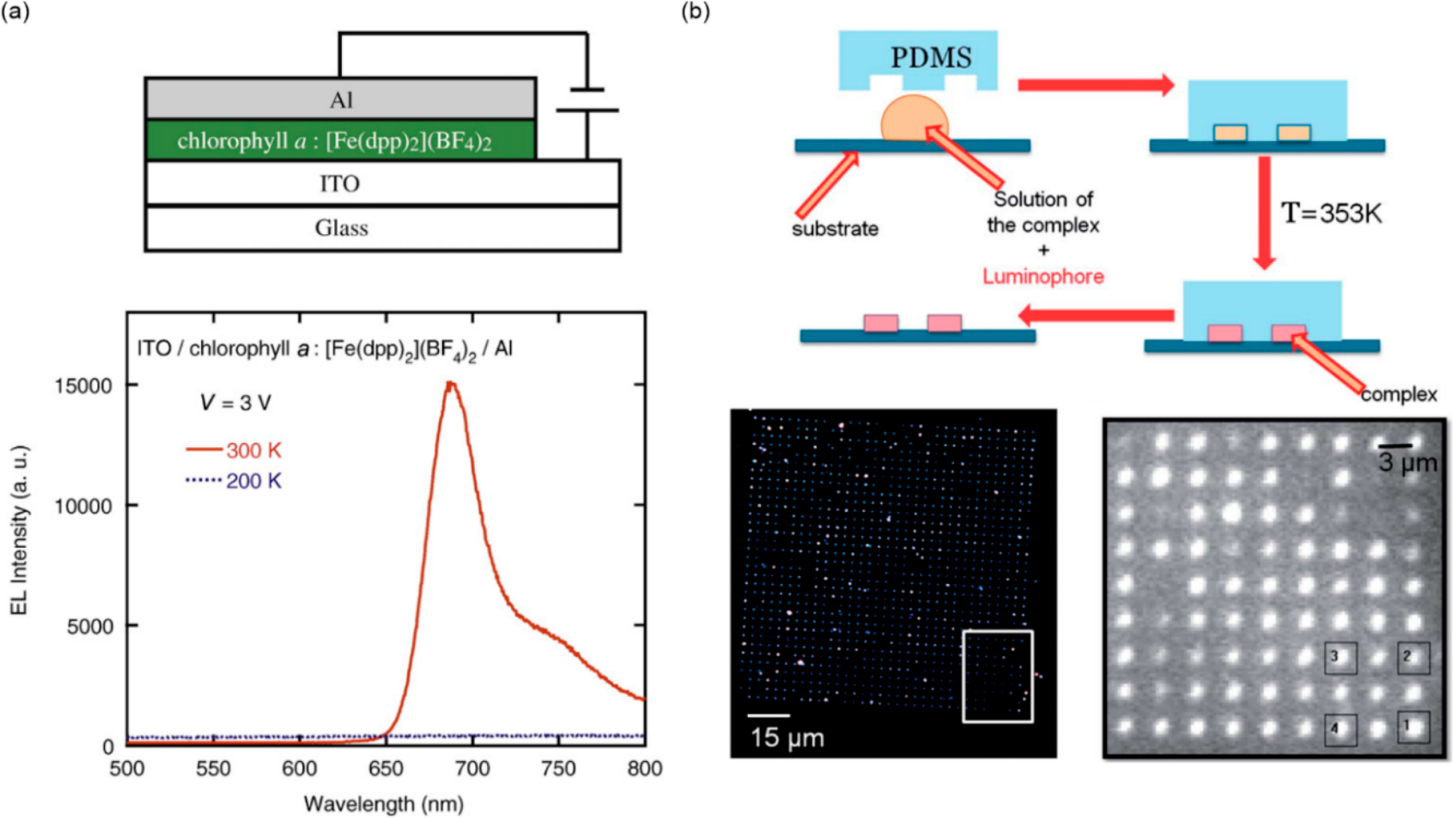
Examples of optical luminescence-based devices with incorporated Fe(II) SCO materials: the device built of the ITO/chlorophyll *a*/[Fe^II^(dpp)_2_](BF_4_)_2_/Al (dpp = 2,6-di-(pyrazol-1-yl)pyridine; where the Fe(II) is a mononuclear complex with two *N*,*N*,*N*-tridentate organic ligands): sequence of the thin films (a, top) and their temperature-variable electro-luminescence at 300 and 200 K under the voltage of 3 V (a, bottom), the scheme of the preparation for the arrays of nanodots based on SCO-active [Fe^II^(hptrz)_3_](OTs)_2_ coordination polymer (the analogous one to presented in Figure 1a; hptrz = 4-heptyl-1,2,4-triazole; OTs = tosylate anion) and doped with the acridine orange (b, top), shown with the dark field image of an obtained nanodot and fluorescence image of the dots in the highlighted area (b, bottom). Part (a) was reproduced with permission from ref (720). Copyright 2008 Elsevier Publishing. Part (b) was reproduced from ref (721) with permission from the Royal Society of Chemistry.

The approach exploring functional composite systems made of luminescent and SCO-active layers was shown by M. Matsuda et al. who proposed a system consisting of a spin-crossover complex of [Fe^II^(dpp)_2_](BF_4_)_2_ (dpp = 2,6-di(pyrazol-1-yl)pyridine) and chlorophyll *a*.^720^ They prepared the ITO/chlorophyll *a*/SCO complex/Al device and investigated its electroluminescent (EL) properties (Figure 34a). The comparison with a reference device, not containing the Fe^II^-based complex, revealed that its introduction into the electroluminescent device results in a small threshold voltage and large external quantum efficiency. More importantly, the luminescent spectra in the low-temperature region, where the Fe(II) metal centers are in the LS state, showed very weak emission only from the ITO electrode and not from the chlorophyll. It suggests that injected electrons pass through the active layer of SCO complexes which dramatically change upon the thermal spin transition leading to almost full quenching of the EL property. This on/off switching is reversible by heating back to the initial state.

The research on nanolayered SCO-fluorophore hybrids was expanded by the work of C. M. Quintero et al. They reported the materials composed of spin-coated thin films of an SCO compound, [Fe^II^(hptrz)_3_](OTs)_2_ (hptrz = 4-heptyl-1,2,4-triazole; OTs = tosylate) doped with luminescent acridine orange.^721^ This molecular luminophore was used in two ways, as the addition to spin-coated thin films as well as to SCO nanoobjects patterned using the soft lithography method (Figure 34b). Owing to a radiationless energy transfer process between the luminophore and the low-spin Fe(II) centers of the complex, it was achievable to follow the spin state changes through luminescence measurements even in thin films of thickness below 100 nm. It was also demonstrated that the layers of luminophore-doped spin transition materials can be patterned using a soft lithography approach and the optical monitoring of the SCO behavior can be realized in a large number of widely spaced individual nanoobjects simultaneously.

**Figure 35:**
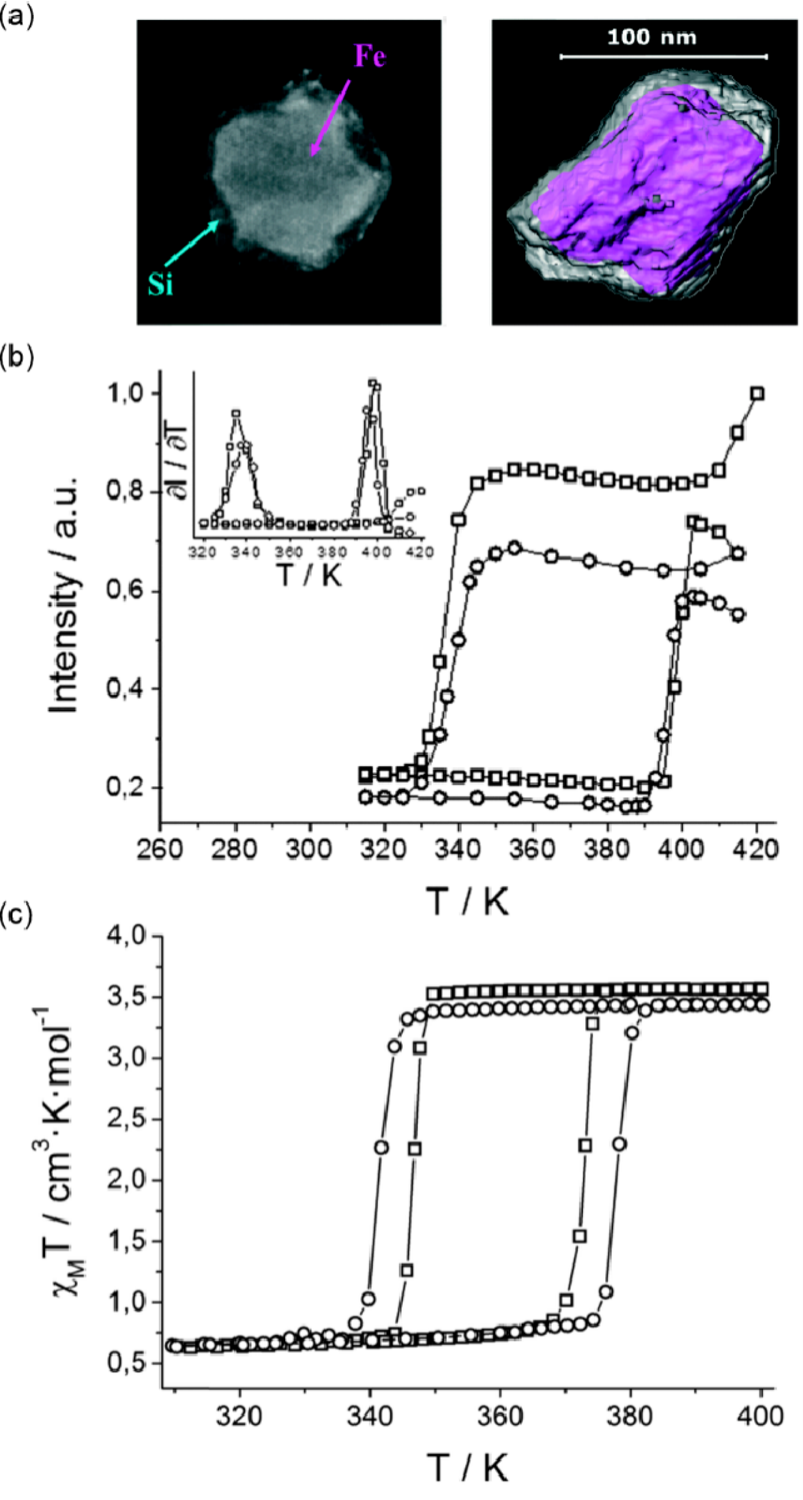
A two-dimensional HAADF-STEM (high-angle annular dark-field imaging on scanning transmission electron microscopy) image showing the core-shell structure of the SiO_2_–[Fe^II^(HTrz)_2_(Trz)](BF_4_) (HTrz = 1H-1,2,4,-triazole; where the Fe(II) part is the structural analog of the compound presented in Figure 1a) SCO nanoparticle and a three-dimensional surface rendered tomographic reconstruction (a), the thermal variation of the emission intensity of this core-shell structure decorated with dansyl (i.e., 5-(dimethylamino)naphthalene-1-sulfonyl) groups under the 495 nm excitation light (b), and the related thermal variation of the *χ*_M_*T* product under the 1 kOe field. Reproduced from ref (725) with permission from the Royal Society of Chemistry.

Some alternative strategies were realized by the functionalization of spin-crossover nanoparticles.^722−726^ Many research groups have concentrated their efforts on the design of bifunctional SCO/luminescence nanoparticles which relied on using a silica matrix for the SCO component. The high porosity of silica allows for the incorporation of SCO complexes and preparation of SiO_2_–SCO systems, in which surfaces can be functionalized by grafting active species, such as organic fluorophores, to afford bifunctional SCO/luminescence nanomaterials (Figure 35).^722−726^ Moreover, silica does not interfere with the magnetic field and is optically silent, so the SCO-core and fluorophore-shell particles keep their original optical and magnetic properties. As a result, the thermal hysteresis loop originating from the spin transition effect can be nicely reproduced in the thermal variation of the emission intensity of a decorating luminescent group, e.g., 5-(dimethylamino)naphthalene-1-sulfonyl) fluorophore.^725^

Luminescent spin transition materials are sometimes accessible through the mixing of SCO complexes with fluorescent molecules, not within a single-phase molecular material but rather in the aggregate or nanomaterials.^727,728^ This approach was used by M. Hiroyuki et al. who described the spin transition phenomena of lipophilic Fe(II)–1,2,4-triazole complexes with the long alkyl chains and their effect on the fluorescence of aromatic chromophores, 9,10-dimethoxyanthracene-2-sulfonate and 1-pyrenesulfonate used as counter-ions in the self-assembled aggregates.^727^ These organic ions were combined with tris(4-(3-dodecyloxypropyl)-1,2,4-triazole)iron(II) complex and their fluorescence characteristics were found to be regulated by thermal spin-state switching. The example of incomplete counterion replacement was shown by L. Salmon et al. who showed the properties of a two-component material system comprised of Fe(II)-triazole-type SCO nanoparticles of [Fe^II^(NH_2_Trz)_3_](X)_2_ (NH_2_Trz = 4-amino-1,2,4-triazole; X = nitrate or bis(2-ethylhexyl) sulfosuccinate anion) compound doped with rhodamine-110 (9-(2-carboxyphenyl)-3,6-diamino-3H-xanthylium) chloride fluorophore.^728^ The advantage of nanoparticles used is that they can be easily modified which enables good control of thermometric properties while the optical properties are virtually unchanged. On the other hand, by doping these complexes with luminescent anions (signal transducer) displaying weak thermal quenching and a good spectral overlap with the absorption of the SCO complexes (in the given spin state), it was shown that the luminescence can conveniently probe the spin state of the system, providing the way for temperature sensing.

**Figure 36:**
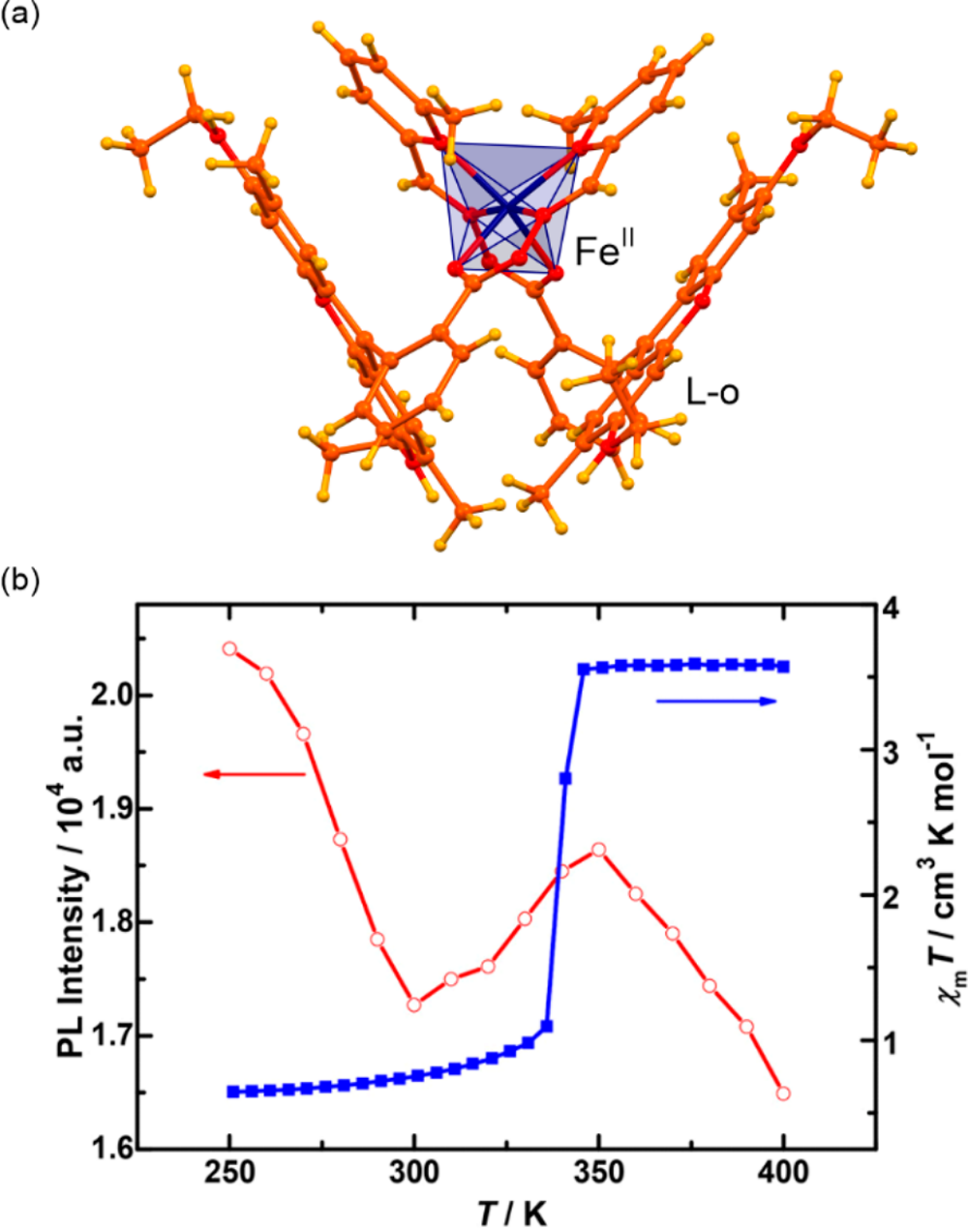
The structure of [Fe^II^(L-o)_2_]^2+^ cationic metal complex bearing the ring-opened ligand (L-o) of pyridinecarbaldehyde rhodamine 6G hydrazone (a),^729^ and the temperature dependences of the *χ*_M_*T* product and the fluorescence intensity at the emission (PL, photoluminescence) maximum under the 560 nm excitation light, gathered for the heating mode (b). Part (b) was adapted with permission from ref (729). Copyright 2018 American Chemical Society.

Aiming at the bifunctionality at the molecular level, i.e., within a single-phase molecular material, the concept is to construct luminescent spin transition materials through the use of a luminophore integrated as an intrinsic part of the SCO-active coordination compound.^729−738^ Being in line with the molecular building block approach, this method can be considered as a direct attachment (through, e.g., coordination bonds or weaker non-covalent interactions but within the single crystal lattice) of a luminophore to the SCO-active metal complex. In this context, a synergetic coupling between luminescence and spin-state switching is demonstrated both in supramolecular SCO systems as well as the related coordination polymers. J. Yuan et al. prepared a fluorescent spin-crossover Fe(II) anionic complex, [Fe^II^(L-o)_2_]^2+^, based on the emissive ring-opened form of a pyridinecarbaldehyde rhodamine 6G hydrazone ligand (L-o, Figure 36).^729^ This was the first example of a bifunctional SCO-emissive hydrazone complex that exhibits a multi-step SCO phenomenon and solvent effects on its temperature course. Moreover, the desolvated complex displays the synergy between SCO and fluorescence as temperature variable luminescence studies revealed a discontinuity in the emission intensity in the 200–400 K temperature range corresponding to the spin transition effect as detected in magnetic measurements. C. Lochenie et al. showed the SCO effect in one-dimensional coordination polymer, [Fe^II^(L_Schiff-phenazine_)(4,4′-bpy)] with a Schiff base-like ligand bearing a phenazine fluorophore (L_Schiff-phenazine_) and bridging 4,4′-bipyridine (4,4′-bpy) ligand.^734^ Investigation of the emission properties of this material with the change of temperature indicates that the SCO phenomenon can be traced by monitoring the luminescence since its color changes from greenish to yellow color upon the LS-to-HS transition. The combination of SCO and luminescence in a synergistic way was also explored for three-dimensional Hofmann-type coordination polymers, {[Fe^II^(L_bpb_/L_bpa_)][M^I^(CN)_2_]_2_} (M = Ag, Au).^736^ In the series with bridging pillared bis(4-pyridyl)butadiyne ligand (L_bpb_), {[Fe^II^(L_bpb_)][M^I^(CN)_2_]_2_}·pyrene, luminescence is an extrinsic property that stems from pyrene guest molecules. In contrast, the series with bridging bis(4-pyridyl)anthracene ligand (L_bpa_), {[Fe^II^(L_bpa_)][M^I^(CN)_2_]_2_}, represents an example of luminescence originating from the intrinsic property of a coordination skeleton.

**Figure 37:**
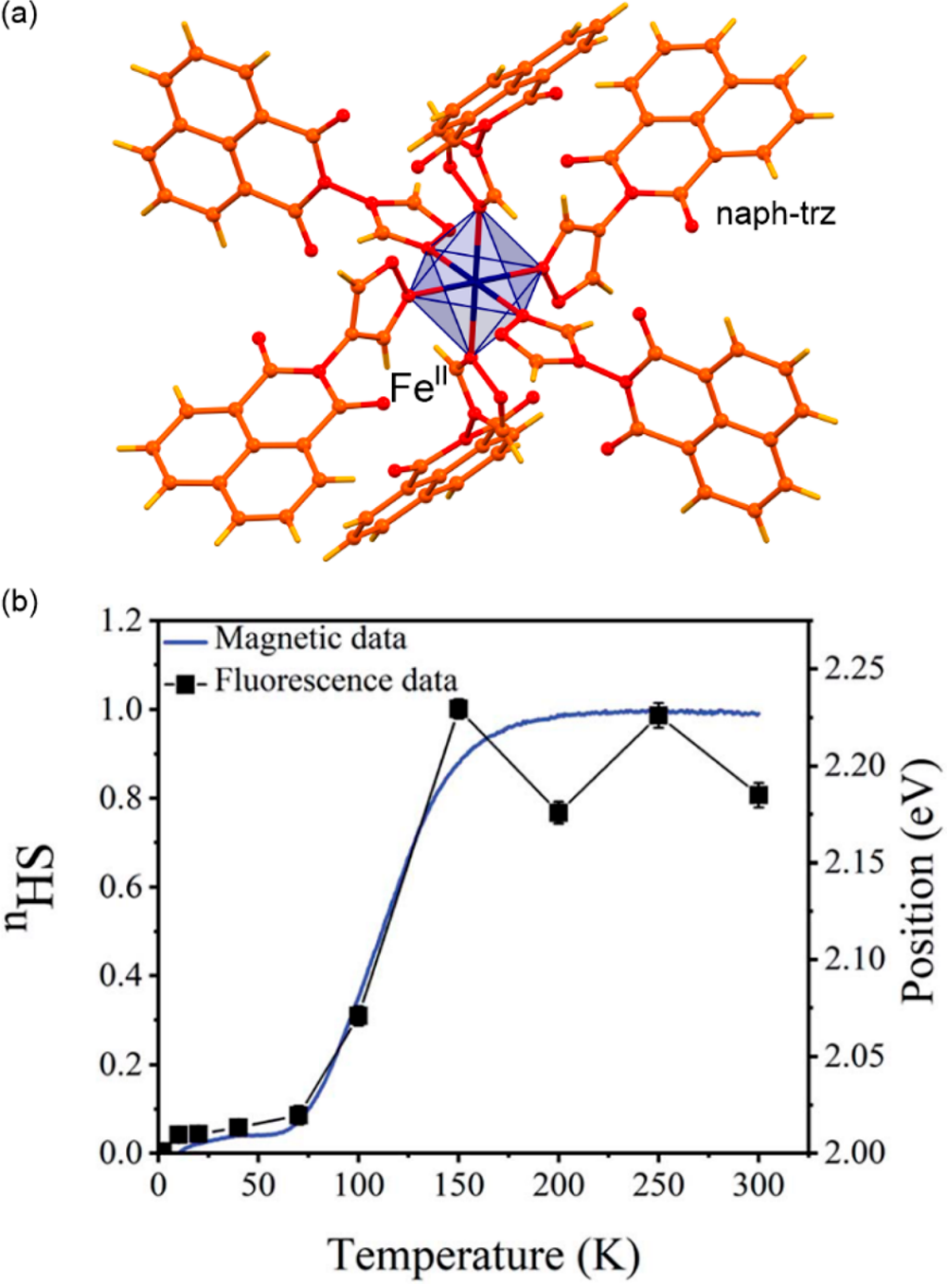
The structure of [Fe^II^(naph-trz)_6_]^2+^ (naph-trz = *N*-(1,2,4-triazol-4-yl)-1,8-naphthalimide) cationic metal complex (a)^737^ and its temperature dependences of the emission peak position (fluorescence data) correlated with the Fe(II) high-spin fraction derived from the magnetic characteristics (magnetic data) (b). Part (b) was reproduced from ref (737) with permission from the Royal Society of Chemistry.

In general, all these synthetic strategies successfully resulted in a more or less marked modulation of the luminescence triggered by the spin state change. Regarding the SCO-luminescence coupling mechanism, the spectral overlap between the luminophore (the sensitizer) and the SCO center (the acceptor) results in an emission-re-absorption process in which the latter acts as a quencher of the luminescence. A strong correlation between SCO and luminescence was recently shown for mononuclear Fe(II) complexes, [Fe^II^(naph-trz)_6_]^2+^, whose coordination sphere is saturated, for the first time, by six phosphorescent ligands, *N*-(1,2,4-triazol-4-yl)-1,8-naphthalimide (naph-trz) (Figure 37).^737^ In this material, the thermal dependence of fluorescence energy perfectly corresponds to the thermal changes in the high-spin fraction derived from the magnetic measurements. Moreover, due to the interaction between both properties, the emerging response is more than the sum of the individual responses of the ligand and the metal. These results are expected to be highly important for future studies on SCO nanoparticles for which the magnetic signal is too low to be studied for single objects, while the emission spectra can be still detected.^737,738^

Very recently, an interesting example of a luminescent SCO Fe(II) molecule-based system was reported, which utilizes the variation of emission from the attached Eu^3+^ ions.^739^ The obtained complexes, [Eu^III^Fe^II^(L2)_3_]^5+^ (L2 = segmental bidentate-tridentate ligand), upon irradiation with the UV light, reveals typical Eu(III) emission of the f–f electronic transitions’ origin in the MeCN solution. While the SCO effect for the Fe(II) center in these molecular cations appears close to the room temperature, the emission intensity was followed in the 233–333 K range. As a result, instead of a continuous increase of the intensity while lowering the temperature, within the ca. 270–310 K range, the integrated intensity drops upon cooling, which is related to the appearance of the energy transfer process between the Eu(III) and LS Fe(II) centers.

In this context, taking into account a large number of SCO complexes, especially those based on the Fe(II) centers, which reveals the LIESST effect in the cryogenic region, one noticed that the related photochromism could be followed by the changes in luminescence if only photoswitching and emission abilities will be simultaneously ensured. Several such systems have been reported up to now; however, no correlation between the emission and LIESST phenomenon was documented in most of them. For instance, such a case was reported for the [Fe^II^(naph-trz)_6_]^2+^ moiety described earlier in this section,^737^ or the different molecular species of [Fe^II^(L_ndptmi_)_2_(NCE)] (E = S, Se; L_ndptmi_ = (naphtha-1-yl)-*N*-(3,5-di(pyridine-2-yl)-4H-1,2,4-triazol-4-yl)methanimine) and for the [Fe^II^(pyr-pybox)_2_]^2+^ (pyr-pybox = 2,2′-(4-(pyren-1-yl)pyridine-2,6-diyl)bis(4,4-dimethyl-4,5-dihydrooxazole)) cation in MeCN solution.^740,741^ Nevertheless, for a molecular analog of the former system, [Fe^II^(L_pdptmi_)_2_(NCS)_2_] (L_pdptmi_ = (pyrene-1-yl)-*N*-(3,5-di(pyridin-2-yl)-4H-1,2,4-triazol-4-yl)methanimine), as well as for the {[Fe^II^(bpben)][Au^I^(CN)_2_]_2_} (bpben = 1,4-bis(4-pyridyl)benzene) Hofmann-type network an influence of low-temperature irradiation upon luminescence was observed.^732,742^ A different example of a photoactive and luminescent SCO complex involves the use of N_4_dte-functionalized chelating ligands (L-N_4_dte = 1,2-bis(5-(2-(pyrazol-3-yl)-pyridin-5-yl)-2-methylthiophen-3-yl)-cyclopentene). The resulting [Fe^II^_2_(L-N_4_dte)_3_]^4+^ molecular cation reveals the conjunction of SCO and LIESST effects, accompanied by the reversible photocyclization in solution at room temperature, modifying the SCO characteristics and ligand-centered emission.^267^ All systems mentioned in this paragraph will be described broadly in section 7.1, related to photoswitchable luminescent molecule-based magnetic materials.

### 4.3 Design of Luminescent Molecular Nanomagnets

Although many different molecular nanomagnets have been reported, the combination of light-emitting functionalities with single-molecule magnetism is still challenging.^100,743,744^ Such magneto-luminescent molecules have the prospects for assisting and enriching the data storage and quantum computing applications typically ascribable to various types of magnetic molecules based on d- and f-block metal complexes. One of the most widely used strategies for the synthesis of luminescent molecular nanomagnets, especially single-molecule magnets (SMMs), is based on exploring the specific physical properties of trivalent lanthanide (Ln^3+^) ions, therefore the luminescent SMMs reported so far are almost all Ln-SMMs, including those based on Dy^3+^, Tb^3+^, Yb^3+^, Er^3+^ ions, as well as a few examples of SMMs containing rarely exploited Ce^3+^, Nd^3+^, and Ho^3+^ ions.^100,743,745^ Emissive Ln-SMMs combine significant magnetic anisotropy of a single ion origin, resulting from the combined contributions from the spin-orbit coupling and crystal-field effects,^744,746,747^ with characteristic luminescent properties of lanthanide ions, usually originating from their metal-centered f–f electronic transitions.^18,185^ The examples of Ln^III^-based coordination systems considered for emission-related applications where the luminescence originates from the organic ligands or attached additional transition metal complexes are relatively rare;^748,749^ however, the coordinated molecules play a crucial role in the sensitization of lanthanide emission which is of great importance since the f–f electronic transitions of 4f metal ions are strictly forbidden and their efficient emission needs proper energy transfer from the neighboring molecular components.^185,750−753^ On the other hand, organic or inorganic ligands, as well as hybrid metalloligands, can affect the magnetic anisotropy of lanthanide ions by modifying their coordination environment which strongly determines the effective anisotropic energy barrier for the SMM property.^74−78,744,746,747^ So, the essence of the construction of luminescent Ln-SMM consists of the use of coordination chemistry tools for the proper modification of lanthanide magnetic properties, as well as the selection of appropriate chromophores for sensitization of their luminescence. The strategy to achieve high-performance SMM property follows generally the rules related to the oblate- or prolate-type of electron density distribution for the most preferred high-value *m*_J_ level of a respective lanthanide ion.^55,744,746,747,754^ This issue was broadly addressed in the last decade in the field of molecular magnetism and several approaches were presented up to date.^74−78,747^ To achieve luminescent Ln-SMMs, the effort cannot be only devoted to the control of magnetic anisotropy of lanthanide complexes but also to generate the desired luminescent functionalities. The numerous electronic levels in lanthanide ions, resulting from the strong spin-orbit coupling, are responsible for their characteristic emission lines, mainly in the visible and near-infrared range.^185^ Unfortunately, as mentioned above, due to the forbidden nature of the transitions within the f orbitals, lanthanide ions are characterized by low molar absorption coefficients. The optical absorption efficiency can be improved by introducing organic, inorganic, or hybrid ligands to the 4f metal ions coordination sphere, which exhibit a high molar absorption coefficient. In most cases, they are capable of absorbing UV light and efficiently filling the excited states of lanthanides through an effective energy transfer.^750−753^ Moreover, they isolate lanthanide ions from the environment, reducing nonradiative relaxation processes.^755^ In this context, many works have been published describing the construction of, usually organic, molecular antennas serving as sensitizers for the optimization of lanthanide luminescent properties.^750−753,756^ The basic principle of sensitization efficiency depends on the energy difference between the ligand triplet (donor) state and the Ln^3+^ emitting (acceptor) state. Usually, when the energy levels of the triplet states of ligands are 2500–4500 cm^–1^ higher than the emitting 4f state, sensitization is the most effective.^757^

As a consequence of these features, in some mononuclear Ln^III^-based complexes presenting the SMM property, organic ligands provide only the appropriate geometry for the magnetic effect but they do not interfere with the observation of lanthanide luminescence. Such examples are sandwich-type lanthanide complexes with macrocyclic ligands, such as Na^I^[Dy^III^(DOTA)(H_2_O)]·solvent (DOTA = anion of 1,4,7,10-tetraazacyclododecane-1,4,7,10-tetraacetic-acid),^237,758^ and [Dy^III^(15C5)(H_2_O)_4_]^−^ or [Dy^III^(12C4)(H_2_O)_5_]^−^, employing various crown ethers (15C5 and 12C4),^759^ in which the fine structure of f–f electronic transitions within luminescence spectra can be observed, despite that the organic ligand does not support directly the lanthanide emission. It is worth noting, that in the area of luminescent materials, the case that the triplet state of an applied ligand does not match the lanthanide emission level is sometimes desired as the dual emission, both from lanthanide ion and ligand, can be then utilized, e.g., for white-light emission or sensing with stimuli-responsive systems.^757,760,761^ Such a situation was presented for various lanthanide molecular materials, including those exhibiting SMM properties.^137,762−764^

**Figure 38:**
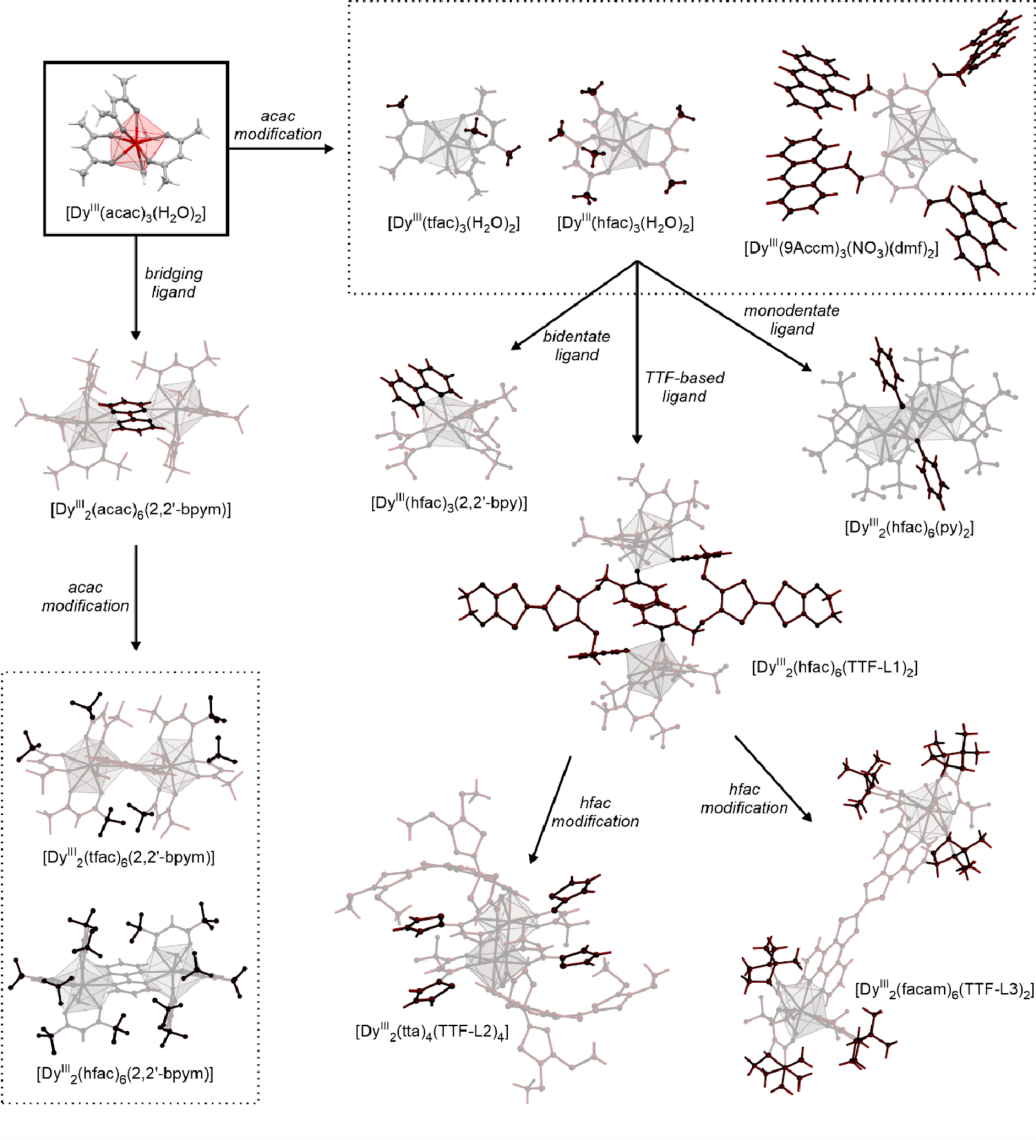
Schematic representation of the routes for structural modifications of luminescent [Dy^III^(acac)_3_(H_2_O)_2_] (acac = acetylacetonate) complexes, realized in two ways, by modifying the acac ligand or coordinating additional antenna ligands with the electron donating ability (tfac = 1,1,1-trifluoroacetylacetonate; hfac = hexafluoroacetylacetonate; 9Accm = 1,7-di-9-anthracene-1,6-heptadiene-3,5-dione; tta = 2-thenoyltrifluoroacetonate; facam = 3- trifluoroacetyl-(+)-camphorate anion; 2,2′-bpym = 2,2′-bipyrimidine; 2,2′-bpy = 2,2′-bipyridine; py = pyridine; TTF-L1, TTF-L2, TTF-L3 = three types of tetrathiafulvalene-based ligands).^766−772^ The key modification parts were indicated by the darker color.

A representative set of results in the quest for luminescent Ln-SMMs is shown in the works on diketonate lanthanide complexes. In 2010, S. Gao and co-workers presented a series of neutral mononuclear [Ln^III^(acac)_3_(H_2_O)_2_] (Ln = Dy, Ho, Er; acac = acetylacetonate anion) complexes with almost ideal *D*_4d_ symmetry exhibiting the SMM behavior with a high energy barrier.^765^ Further efforts concerned the introduction and modification of luminescent properties of these complexes, which was realized in two ways: (i) by modifying the acac ligand or (ii) by replacing coordinated water molecules with appropriate auxiliary antenna ligands (Figure 38).^766−772^ In this regard, the acac molecules can be modified with strong electron-withdrawing trifluoromethyl groups which was shown to improve the Ln^III^-centered luminescence. The resulting complexes of the [Ln^III^(hfac)_3_(H_2_O)_2_] (hfac = hexafluoroacetylacetonate) composition were used to prepare {[Dy^III^(hfac)_3_(pyNO)]_2_} (pyNO = pyridine *N*-oxide) derivatives showing remarkable SMM behavior and metal-based luminescence with a quantum yield of 0.1%.^766^ Despite the luminescence sensitization by the organic ligand, this small value was postulated to be due to multiphonon deactivation by N–O vibrations. Aqua ligands were also replaced with a chelating 2,2′-bipyridine (2,2′-bpy) to prepare [Dy^III^(hfac)_3_(2,2′-bpy)] species.^767^ Under UV excitation, these complexes exhibit Dy^III^-centered f–f emission more efficiently sensitized by 2,2′-bpy ligand than for the pyNO case.

An attractive continuation of this research was realized by reporting the extensive library of organic binders that can modify the acac-side for this type of complexes. The selected ligand of 1,7-di-9-anthracene-1,6-heptadiene-3,5-dione (9Accm), belonging to the broad family of curcuminoid systems, was applied to prepare the [Dy^III^(9Accm)_3_(NO_3_)(dmf)_2_] and [Dy^III^(9Accm)_3_(py)] (py = pyridine) compounds which reveal ligand-based visible emission and dual luminescence (visible ligand-based emission and NIR emission of lanthanides), respectively.^768^ Later, F. Pointillart and co-workers demonstrated the next generation of advanced materials from this family of compounds which relies on the combination of other Ln^III^-diketonate complexes with tetrathiafulvalene-based ligands that are able to efficiently sensitize the 4f metal ions emission, especially in the NIR range.^769−772^

Apart from some other examples of homometallic luminescent coordination polymers showing the SMM property,^773−777^ the use of transition-metal-based chromophores as sensitizers for lanthanide luminescence was explored as an alternative route toward emissive molecular nanomagnets. They reveal a few promising features, including suitable energies of triplet (donor) states for the sensitization of vis-to-NIR 4f-metal-centered emission, relatively long excited-state lifetimes, and high efficiencies of intersystem crossing.^753^ Simultaneously, transition metal complexes can play the analogous role as organic ligands in constraining the lanthanide geometry toward SMM properties.^100,778^ In this group, compartmental ligands like Schiff-bases are widely used for the construction of luminescent SMM systems, due to the two different coordination sites showing affinity with transition metal ions and Ln^3+^ ions.^779^ In this regard, many examples of Zn^II^–Ln^III^ heterometallic molecules utilizing such compartmental ligands were presented. It was found that Zn(II)-containing chromophore can absorb energy and transfer energy towards lanthanide ions in the outer site, resulting in an enhancement of the luminescence while the proper positions of negatively charged O-donor coordination sites ensure the SMM effect.^780−784^ Many luminescent molecular nanomagnets involve also other transition metal complexes.^785−799^ For instance, an Ir(III)–Dy(III) phosphonate, [Dy^III^Ir^III^_6_(ppy)_12_(bpp)_2_(bppH)_4_]^−^, containing [Ir^III^(ppy)_2_(bpp)] (ppy = 2-phenylpyridine, bpp = 2-pyridylphosphonate) complexes, shows dual functionality, i.e., photoluminescence and field-induced slow relaxation of magnetization.^785^ Its solid-state emission spectrum exhibits a broad band centered at ca. 531 nm, attributed to the LMCT (ligand-to-metal charge transfer) transition of the Ir(III)-based moieties which indicated that the triplet states of Ir^III^-containing chromophores lie below the emitting state of the Dy^3+^ ion, leading to an ineffective sensitization process. Thus, the emission is separately related to the Ir(III) center while the SMM property corresponds to the Dy(III) center. This disadvantage could be overcome using cyanido transition metal complexes as the supporting metalloligands for lanthanide complexes.

**Figure 39:**
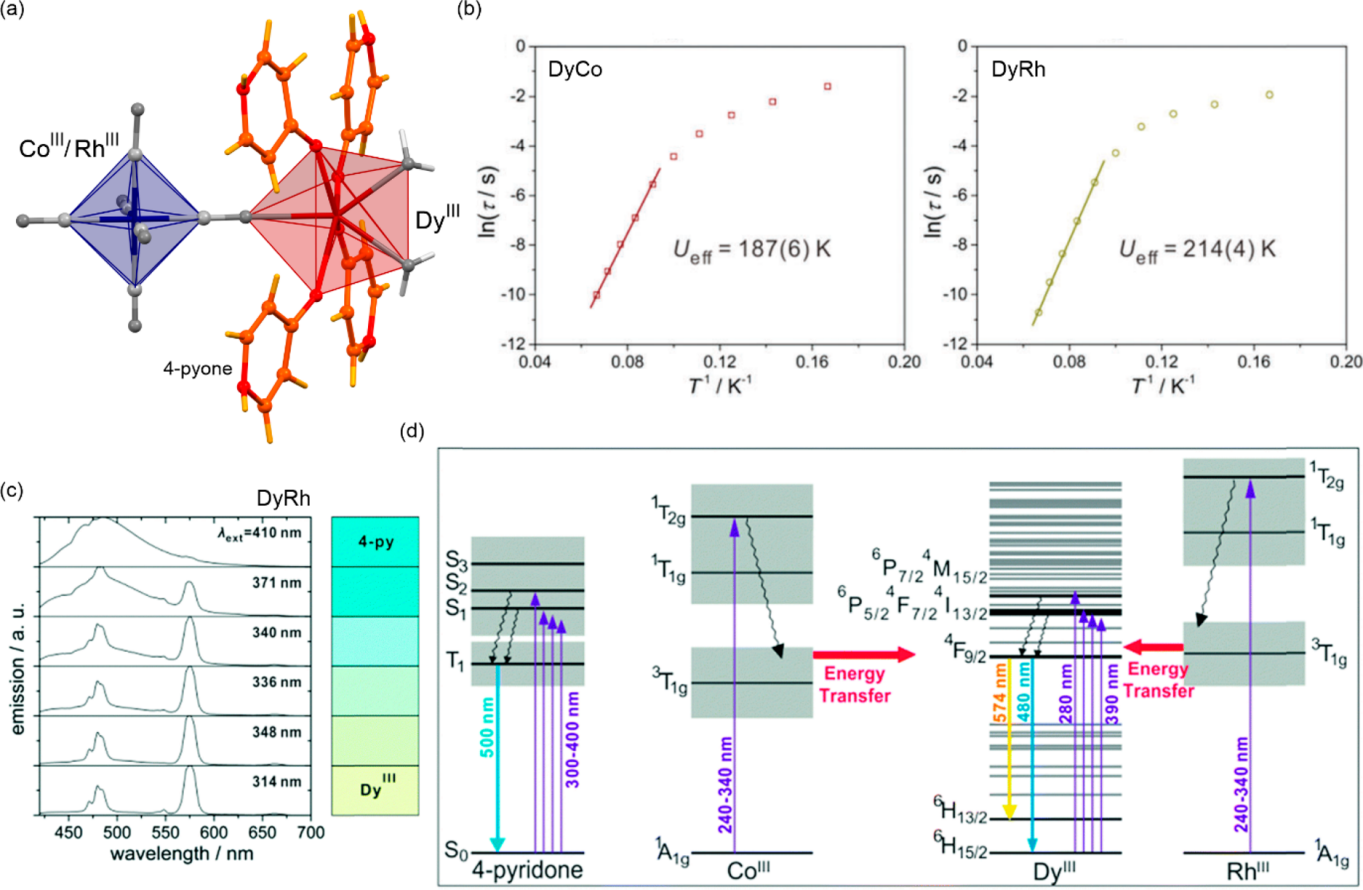
The structure of {[Dy^III^(4-pyone)_4_(H_2_O)_2_][M^III^(CN)_6_]}·*n*H_2_O (4-pyone = 4-pyridone; M = Co, Rh) dinuclear molecules (a),^793^ their temperature dependences of the zero-*dc*-field magnetic relaxation times with the indicated effective thermal energy barriers representing the strength of magnetic anisotropy in both compounds (b), room-temperature solid-state emission spectra of the {Dy^III^Rh^III^} analog obtained under the indicated excitation wavelengths, shown together with the resulting emission colors (c), and the schematic energy level diagram showing the mechanisms of electronic interplay between incorporated molecular building blocks (d). Parts (b), (c), and (d) were reproduced from ref (793) with permission from the Royal Society of Chemistry.

Polycyanidometallates, thanks to the coordination through a short cyanido molecular bridge, can efficiently transfer energy to lanthanide ions if only the donor electronic states of proper energy are accessible.^788−799^ In this context, many systems involving complexes with a different number of cyanido ligands have been analyzed, including [M^I^(CN)_2_]^−^ (M = Au, Ag),^788,789^ [Fe^II^(CN)_2_(2,2′-bpy)_2_] (2,2′-bpy = 2,2′-bipyridine),^790^ [Cd^II^(CN)_4_]^2–^,^789^ [Pt^IV^Br_2_(CN)_4_]^2–^,^791^ [Cu^I^_2_(CN)_5_]^3–^,^792^ [M^III^(CN)_6_]^3–^ (M = Co, Rh),^239,789,793-787^ [M^IV/V^(CN)_8_]^4–/3–^ (M = Mo, W, Re),^798,799^ and others. Cyanido transition metal complexes were found efficient in inducing both strong lanthanide magnetic anisotropy as well as tunable emission properties. From the optical properties point of view, the luminescence of lanthanide ions, observed in the visible or NIR range, can be effectively enhanced by polycyanidometallate ions using their charge transfer or d–d electronic states that serve as donor states within the antenna effect.^239,789,793,794^ Moreover, the magnetic anisotropy of lanthanide ions can be controlled using cyanido metal complexes applied as the support providing a rigid framework whereas additional organic ligands induce proper magnetic axiality.^792,800^ Alternatively, cyanido ligands, especially used as a sole type of ligand in the coordination sphere, can be also used as the direct source of lanthanide magnetic anisotropy.^134,794^ The approach of using polycyanidometallates for the design of luminescent Ln-SMMs can be exemplified by dinuclear {[Dy^III^(4-pyridone)_4_(H_2_O)_2_][M^III^(CN)_6_]}·solvent (M = Co, Rh) molecules (Figure 39).^793^ They exhibit a dual magneto-luminescent nature that combines color-tunable photoluminescence and zero-*dc*-field slow magnetic relaxation. At room temperature, the emission of {Dy^III^Co^III^} molecule covers the colors from yellow to greenish blue while the emission of Rh^III^-analog is slightly blue-shifted ranging from yellow to light blue, including white light emission for the excitation by the 336 nm light. This prominent tunability of emission color is ascribed to the mixing of two emissive components (from the 4-pyridone ligand and lanthanide ion) in various ratios by a set of selected excitation wavelengths. In this context, a hexacyanidometallate ion plays the role of a sensitizer of lanthanide emission which affects the ligand-to-lanthanide emission equilibrium. As a result, white light emission can be achieved by the Co-to-Rh substitution which happens concomitantly with the improvement of SMM properties illustrated by the increase of effective anisotropic energy barrier.^793^

### 4.4 Luminescent Thermometers Based on Single-Molecule Magnets

The conventional strategies to measure temperature, one of the most critical parameters influencing every physical or chemical process, cannot be conveniently used at the nanoscale because of size limitations regarding the sensor itself. As a consequence, new, very different nanothermometry has emerged aiming at the development of thermometers with micrometric or sub-micrometric spatial resolution.^801−803^ Similar issues appear for tracing the temperature in the non-invasive contactless way which is often crucial for various, e.g., biological, applications, and cannot be easily realized by classical methods of thermometry. In this regard, the optical thermometry utilizing thermally activated luminescence of lanthanide ions in diverse materials was investigated.^761,802,804,805^ Taking into account that lanthanide ions are also great prerequisites for the construction of single-molecule magnets (SMMs),^79,743,744^ a new pathway toward Ln-SMM-based luminescent thermometers appeared. It opens the avenue toward contactless temperature sensing for electronic devices, medical diagnostics, or chemical reactors, and in combination with the SMM effect, gives the possibility to construct bifunctional magnetic luminescent thermometers, considered for future electromagnetic SMM-based devices self-monitoring its temperature.^694,806−808^

The principles for the construction of luminescent thermometers based on molecular nanomagnets require the simultaneous design of magnetic anisotropy and thermally modulated emission. The application of such systems in real devices assumes thermal overlapping of these two properties, allowing the possibility of monitoring the temperature at low temperatures within the SMM behavior.^134,808^ However, the thermal probing capabilities of such systems can be extended to higher temperatures, as thermally activated mechanisms of non-radiative relaxation can affect the luminescent properties in various temperature ranges.^795,809,810^ An additional advantage and perspective is the modulation of the luminescent properties of such nanothermometers under the influence of an external magnetic field (see section 4.6).^808^ The set of energy states of lanthanide ions is only weakly modified within the crystalline environment, becoming its luminescent fingerprint (used for optical probing).^179^ Despite this, the 4f emission bands depend on the temperature in different ways. Firstly, as the temperature increases, their emission peaks get wider due to homogeneous broadening which is related to lattice vibrations.^811^ Moreover, the overall intensity of the spectrum decreases as nonradiative, i.e., assisted by lattice phonons, relaxation routes become more likely.^812^ For the same reason, the lifetimes of radiative transitions become shorter. An additional feature influencing the thermometric behavior of 4f metal complexes is the efficiency of the temperature-dependent energy transfer from ligands or attached metal complexes.^813−815^ All these features contribute to the great interest in Ln^III^-based optical thermometers, including those that are based on lanthanide SMMs. Such systems have already been described,^100,699,805,808^ so we here outline the design principles for optical thermometers based on SMMs using selected examples.

**Figure 40:**
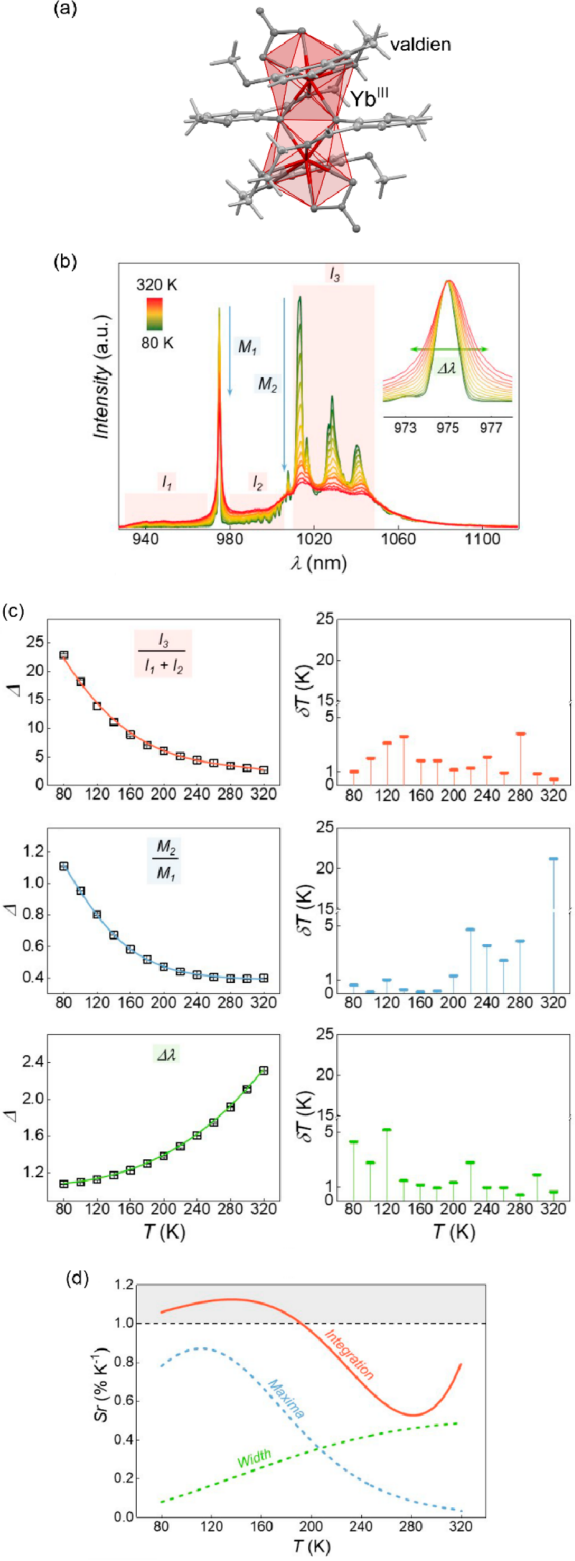
The structure of [Yb^III^_2_(valdien)_2_(NO_3_)_2_] (valdien = the deprotonated form of *N*1,*N*3-bis(3-methoxysalicylidene)diethylenetriamine) dinuclear molecule serving as a field-induced SMM (a)^817^ and its optical properties, including the NIR emission spectra related to the Yb(III)-centered ^2^F_5/2_→^2^F_7/2_ transition, recorded under the 375 nm excitation in the 80–320 K temperatures range (b), the thermometric parameters (*Δ*) for indicated thermometric approaches (the ratio between integrated regions, the ratio between selected detailed emission components, and the width of the selected emission peak), shown together with the respective fits of the *Δ*(*T*) dependences and the temperature uncertainties of the optical thermometric effect (c), and the relative thermal sensitivities of the considered thermometric approaches (d). Parts (b), (c), and (d) were reproduced from ref (817) with permission from the Royal Society of Chemistry.

Most SMM-based optical thermometers are based on Dy^3+^ and Yb^3+^ ions, while the other lanthanides were explored to a much lesser extent.^239,772,795,808,816−821^ The former can be characterized by very strong magnetic anisotropy but their yellow luminescence is rather weak.^56,751^ In contrast, the temperature-modulated NIR luminescence of Yb^3+^ ions is often well-explored but slow magnetic relaxation almost exclusively appears in the presence of an external magnetic field due to strong quantum tunneling of magnetization.^822,823^ These remarks underline how challenging is to take control of these two properties, the SMM behavior and optical thermometry, simultaneously. From the emission perspective, the use of organic ligands or metal complexes serving as sensitizers for the optimization of luminescent properties of lanthanide complexes, e.g., increasing the emission quantum yield, can be valuable in optical thermometry.^813−815^ In this context, the temperature can lead to a higher probability of the back-energy transfer from the 4f-metal center to the ligand, which affects the spectrum greatly and can be employed for thermometry.^805,824^ Various approaches for self-calibrating luminescent thermometers are exploited, all based on variations of the emission profile associated with f–f electronic transitions of the lanthanide ion. This includes, e.g., the ratio between the integrated intensity of selected bands or their sums, a straightforward comparison of the intensity of selected bands, the inhomogeneous broadening of the emission profile, or the emission lifetime.^805^ All these possibilities were found for NIR-emissive [Yb^III^_2_(valdien)_2_(NO_3_)_2_] (valdien = the deprotonated form of *N*1,*N*3-bis(3-methoxysalicylidene)diethylenetriamine) dinuclear molecule serving also as a field-induced Single-Molecule Magnet (Figure 40).^817^

**Figure 41:**
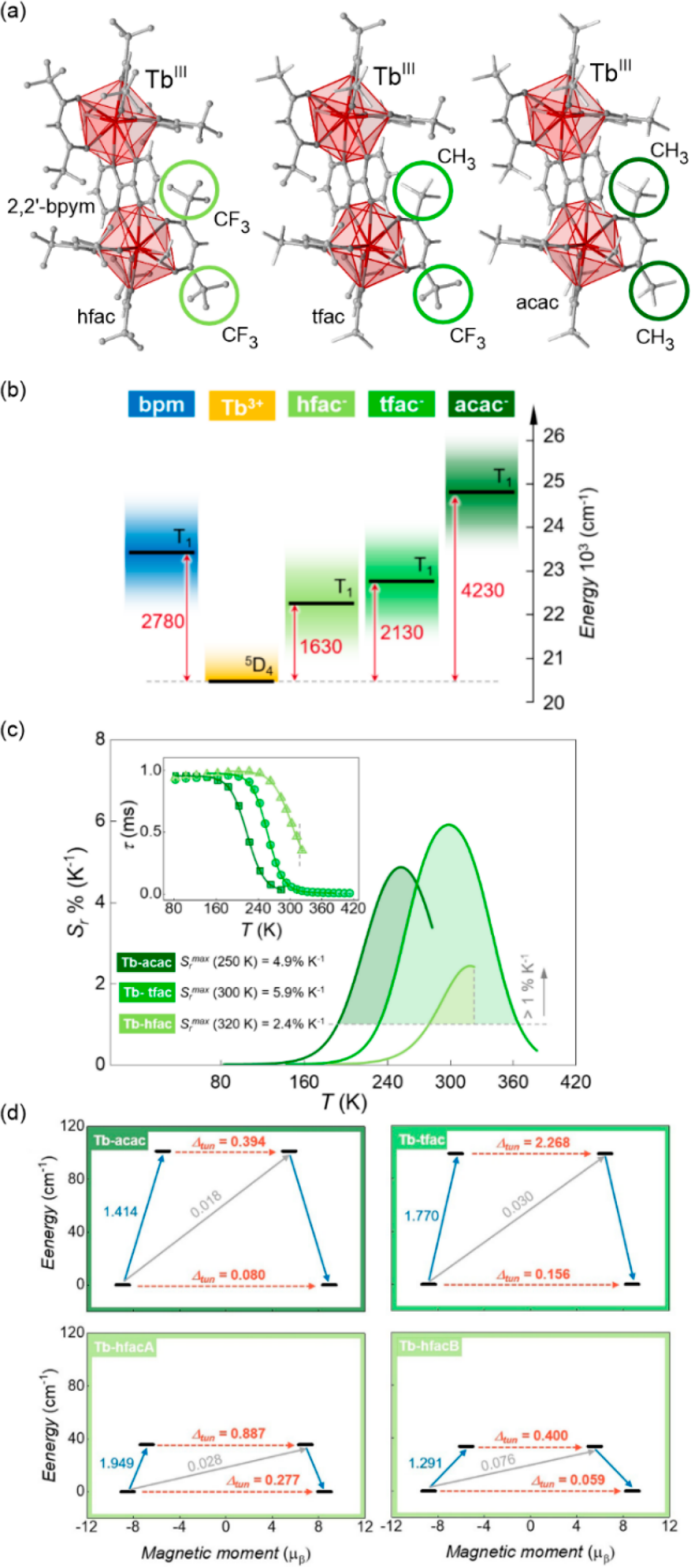
The representative views on the series of [Tb^III^_2_(2,2′-bpym)(L_dk_)_6_] (2,2′-bpym = 2,2′-bipyrimidine; L_dk_ stands for three different diketonate ligands, including hfac = hexafluoroacetylacetone, tfac = 1,1,1-trifluoroacetylacetonate, and acac = acetylacetonate) dinuclear molecules (a),^772^ the relative energy positions of the triplet states of the incorporated ligands in relation to the ^5^D_4_ emitting level of Tb(III) centers (b), the comparison of the relative thermal sensitivity curves, *S*_r_(*T*), obtained from the fitting procedure of the temperature dependences of emission lifetime values shown in the inset (c), and the *ab-initio*-calculated energy splitting of two lowest-lying pairs of the *m*_J_ levels for the indicated complexes, shown together with the transition probabilities and tunneling gaps representing the magnetic relaxation processes observed for these molecules (d). Parts (b), (c), and (d) were adapted with permission from ref (772). Copyright 2019 John Wiley & Sons.

Fine-tuning of the ligand scaffold that results in better luminescence thermometric parameters was shown by M. Murugesu and co-workers. They report the family of dinuclear Tb^III^ complexes, [Tb^III^_2_(bpym)(L_dk_)_6_] (bpym = 2,2′-bipyrimidine), with acac (acetylacetonate), tfac (1,1,1-trifluoroacetylacetonate) and hfac (hexafluoroacetylacetone) diketonate ligands (L_dk_) for which the impact of modified ligands on the coordination geometry of the metal center and the sensitization of its photoluminescence was studied (Figure 41).^772^ Apart from the influence of the ligand’s modification on the magnetic properties, the energy difference between Tb^III^ emitting ^5^D_4_ state and the closest triplet state (either of bpym or diketonate ligands) influences the brightness of the complex emission and governs its behavior as a luminescent thermometer. In the family of related compounds, optical properties of the [Dy^III^_2_(bpym)(tfac)_6_] were exploited for the construction of quite a remarkable luminescent thermometer.^808^ In this case, the thermometry is enabled by two mechanisms, each predominant in different temperature ranges. Above room temperature, an increased probability of back-energy transfer from Dy^III^ centers to ligands allows the use of two selected emission bands to probe the temperature (double-band thermometer, engaging ligand phosphorescence and lanthanide emission). On the contrary, at low temperatures, the thermally induced electron population redistribution between sublevels of the Dy^III^^4^F_9/2_ multiplet was used, which works down to the cryogenic temperatures regime (single-band thermometry). This enables probing temperature at the scale of a single molecule over a broad temperature range spanning from approximately 5 to 398 K.^808^

**Figure 42:**
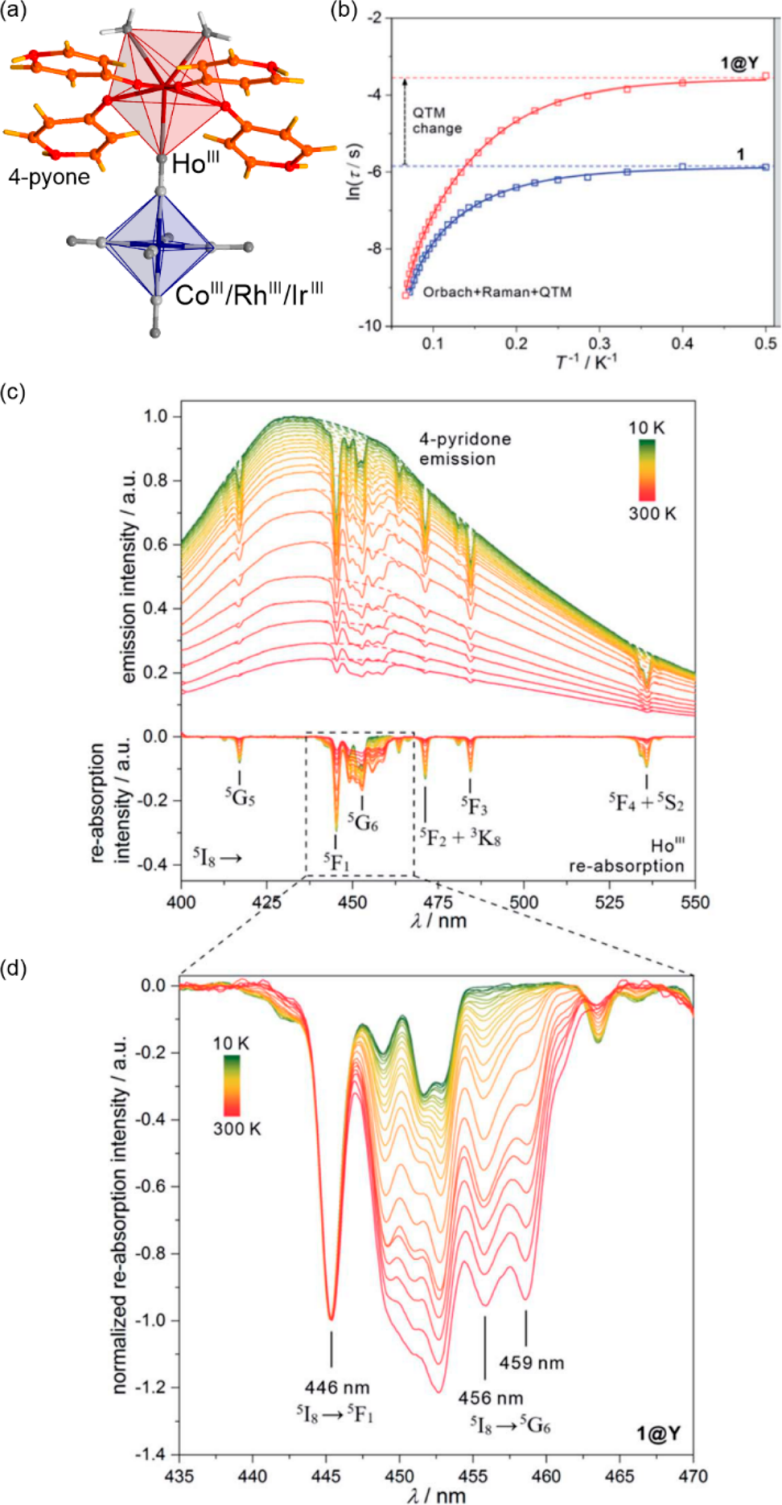
The structure of {[Ho^III^(4-pyone)_4_(H_2_O)_2_][M^III^(CN)_6_]}·*n*H_2_O (4-pyone = 4-pirydone; M = Co, Rh, Ir) dinuclear molecules (a),^795^ the temperature dependence of zero-*dc*-field magnetic relaxation times for Ho–Co compound (**1**) and its Y(III)-diluted analog (**1@Y**), shown with indicated dominant magnetic relaxation processes (b), the set of temperature-dependent emission spectra of **1@Y** measured under the 370 nm light irradiation, shown with the extracted luminescence re-absorption spectra obtained after the subtraction of the ligand emission component (c), and the enlargement of the related re-absorption spectra in the wavelength range of 435–470 nm, representing the efficient optical thermometry effect (d). Parts (b), (c), and (d) were reproduced from ref (795) with permission from the Royal Society of Chemistry.

Considering other lanthanide ions in the context of SMM-based optical thermometers, Ho^III^ complexes appear to be an unusual alternative. They can reveal zero-*dc*-field slow magnetic relaxation in the coordination environment similar to Dy^III^ SMMs but exhibit usually rather weak luminescence.^715,795^ However, it was found that an efficient luminescent re-absorption effect, appearing due to the numerous absorption peaks of Ho^3+^ ions in the visible range, can be applied for achieving a high-performance SMM-based optical thermometer.^795^ In the series of dinuclear molecules, {[Ho^III^(4-pyridone)_4_(H_2_O)_2_][M^III^(CN)_6_]}·solvent (M = Co, Rh, Ir), the selected 4-pyridone ligands exhibit pronounced blue emission and ensure substantial magnetic anisotropy of Ho^III^ centers, resulting in the SMM property, improved after magnetic dilution with Y^3+^ ions (Figure 42). Moreover, strongly temperature-dependent Ho^III^ absorption peaks may be easily detected within the emission pattern of the attached luminophore which was used as a source of ratiometric luminescent thermometry with an impressive relative thermal sensitivity. Moreover, both the crucial re-absorption effect as well as the lanthanide magnetic anisotropy could be further modulated by the attached [M^III^(CN)_6_]^3–^ ions.^795^

Among other recent approaches toward next-generation SMM-based luminescent thermometers, NIR emissive Nd^III^ centers showing field-induced SMM behavior were utilized.^818,825^ More importantly, strongly magnetically anisotropic and, simultaneously, green-emissive Tb^III^ complexes were also employed which provides a wide temperature overlap between the optical thermometry and SMM features.^134^ Last but not least, the first attempts at combining the emission signal with magnetic characteristics in formulating advanced thermometric parameters for high-performance SMM-based opto-magnetic thermometers were also reported.^826^

Among luminescent thermometers based on SMMs, a single unique example of ytterbium(III)-based molecular anion was found to exhibit the conjunction of field-induced slow magnetic relaxation and NIR emission utilized to construct the optical thermometer based on the ratio between hot and cold emission components, which is additionally accompanied by the proton conductivity originating from the presence of cationic charge carriers crystallizing with anionic luminescent SMMs.^239^ Due to its multifunctional character, this unusual case of SMM-based luminescent thermometer will be described in section 7.4.

### 4.5 Magneto-Optical Correlations in Luminescent Molecular Nanomagnets

For future applications of SMMs in high-density information storage and spintronics, it is essential to understand the mechanisms staying behind their magnetic hysteresis effect which is related to the energies and features of sublevels of the ground electronic multiplets.^59,72,746,827,828^ In this context, direct-current (*dc*) magnetic measurements, including temperature dependence of magnetic susceptibility and field dependences of magnetization, can be used to get insight into the energy splitting of the ground multiplet; however, it becomes a difficult task when the molecular symmetry of a metal complex is low.^794,829^ The theoretical approaches, such as *ab initio* calculations, are the solution as they can provide the energy splitting of the ground multiplet but the experimental support is crucial to validate the performed calculations.^743,816,829,830^ Moreover, some important features could not be fully taken into account, such as deviation from the ideal geometry which is often not easily reflected in the structural model, e.g., due to the significant structural disorder, non-negligible magnetic interactions that cannot be treated easily using the *ab initio* approach; in addition, the effect of the magnetic field on the energy scheme of the ground electronic multiplet should be examined experimentally to fully understand the scope of opto-magnetic interactions in an investigated SMM system (see section 4.6.).^743,830,831^

Considering the usage of optical effects in studying the crucial energy splitting of the ground multiplet in SMMs, the set of investigated molecular nanomagnets mainly includes those based on trivalent lanthanide ions. The energy positions of the *m*_J_ levels within the ground multiplet can be extracted spectroscopically which provides the energy gaps that can be further compared with the best-fit parameters obtained in the analysis of slow magnetic relaxation processes, e.g., an energy barrier related to the Orbach relaxation.^744,745,830,811^ In this context, far-infrared (FIR) absorption spectra as well as emission characteristics were employed to gain information on the lowest-lying energy levels of the Ln SMMs (refs (237, 239, 759, 769, 770, 773, 774, 780, 789−792, 794, 798, 816, 817, 819, 821, and 831−837)). When compared with SMMs constructed of high-nuclearity clusters based on various metal ions whose mechanisms of slow magnetic relaxation processes are often difficult to be explained using optical methods even with the support of theoretical calculations,^61,827,838,839^ lanthanide molecular nanomagnets, due to their relative simplicity, are excellent objects for such magneto-optical correlations.^237,840^ Below, we describe a few examples presenting the general idea of the application of high-resolution optical spectra, mainly emission ones, in the elucidation of magnetic phenomena of lanthanide-based SMMs.^770,773,780,789,816,834−837^

**Figure 43:**
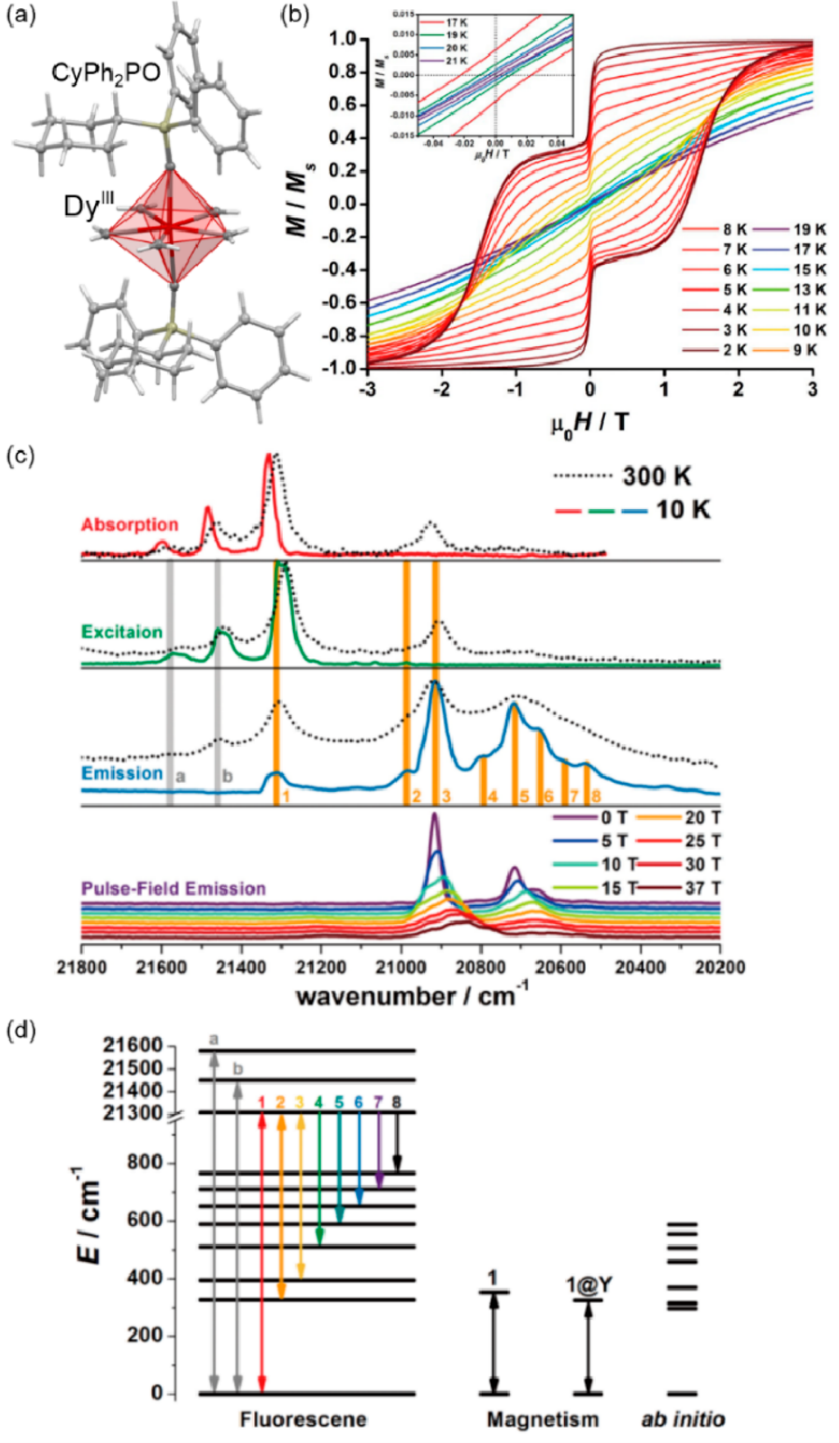
The structure of [Dy^III^(CyPh_2_PO)_2_(H_2_O)_5_]^−^ (CyPh_2_PO = cyclohexyl(diphenyl)phosphine oxide) anionic metal complex (a),^837^ its temperature-variable magnetization versus magnetic field curves (b), the set of absorption, excitation (*λ*_em_ = 574 nm), and emission (*λ*_exc_ = 361 nm) spectra, measured at 300 and 10 K (the vertical lines indicate the postulated energies of the emission components related to the ^4^F_9/2_→^6^H_15/2_ electronic transition), shown together with the pulsed-field emission spectrum at 5 K (c), and the schematic energy levels diagram determined by the fluorescence spectra, compared with the anisotropic energy barriers for the Dy(III)-based SMMs, found experimentally using the magnetic data of the Dy(III) complex (**1**) and its Y(III)-diluted analog (**1@Y**) as well as determined theoretically (*ab initio*) (d). Parts (b), (c), and (d) were adapted with permission from ref (837). Copyright 2017 John Wiley & Sons.

The correlations between the emission spectra and magnetic properties mainly concern luminescent SMMs based on Dy^3+^ and Yb^3+^ ions. In the first group, J. Long et al. showed magneto-luminescent correlations in dinuclear {[Zn^II^(NO_3_)(L_bmsda_)Dy^III^(NO_3_)_2_(H_2_O)]} complex with compartmental *N*,*N*-bis(3-methoxysalicylidene)-1,2-diaminoethane ligand (L_bmsda_), which exhibits field-induced SMM properties and sensitized luminescence of incorporated Dy^3+^ ions.^780^ At the low temperature (10 K), the emissive transition to the ground state, ^4^F_9/2_ → ^6^H_15/2_, ranges from 20500 to 21300 cm^–1^ and can be well modeled assuming 12 components of a multi-Gaussian function fit. More than eight electronic transitions expected for the ^6^H_15/2_ multiplet from the bottom of the emissive one indicate the presence of four additional "hot bands" originating from the first excited state above the lowest lying *m*_J_ level of the ^4^F_9/2_ level (approximately 55 cm^–1^ higher). The analysis in the high-energy area allows authors to determine the energy difference between the two lowest ±*m*_J_ states of the ground ^6^H_15/2_ multiplet (48 cm^–1^), which is quite consistent with the barrier obtained from the *ac* magnetic data (35 cm^–1^). Moreover, the compatibility of both designated barriers is even better for the diluted complex in the yttrium matrix (45 cm^–1^ fitted for *ac* data), due to the weakening of the dipole interactions and the reduction of the QTM effect. The similarity of the emission spectra confirms also that the Dy(III) chemical environment remains identical in the diluted matrix, and does not affect the Orbach energy barrier.^780^ A similar comparison of the thermal energy barrier obtained from the magnetic data and emission spectra was performed for many other Dy^3+^ complexes.^834−837^ For instance, comprehensive magneto-optical correlations were performed for mononuclear [Dy^III^(CyPh_2_PO)_2_(H_2_O)_5_]^−^ (CyPh_2_PO = cyclohexyl(diphenyl)phosphine oxide) complex serving as a high-performance SMM (Figure 43).^837^ In emission spectra at 10 K, eight Kramers doublets coming from the crystal-field splitting of the ^6^H_15/2_ multiplet can be successively determined from the fine structure of the ^4^F_9/2_→^6^H_15/2_ transition. The energy gap matches well with the spin-reversal barrier determined by the magnetic measurements for both the original and Y^III^-diluted samples and shows semi-quantitative agreement with the *ab initio* calculations. The excitation and absorption spectra provide also supporting evidence on the details of the energy splitting of the ground multiplet. Moreover, the influence of the magnetic field on the ground-state energetic scheme was also discussed using the emission spectra.^837^

A rare example of the magneto-optical correlations in an Er^III^-based luminescent SMM was shown for a layered phosphonate [Er^III^(notpH_4_)(H_2_O)]ClO_4_·solvent (notpH_6_ = 1,4,7-triazacyclononane-1,4,7-triyl-tris(methylenephosphonic acid)) coordination network.^773^ This compound shows field-tunable dual magnetic relaxation processes, both thermally activated, which co-exist with the near-infrared 4f-metal-centered photoluminescence. The room-temperature well-resolved spectrum reveals characteristic ^4^I_13/2_→^4^I_15/2_ Er^III^ emission which was fitted with a 10-component multi-Gaussian function. Apart from eight bands resulting from the splitting of the ground ^4^I_15/2_ multiplet, the other bands result from hot transitions, and the assignment of the peak positions provides insight into the energy level diagram. The energy gap between the ground and the first excited sublevel of the ground ^4^I_15/2_ multiplet is around 31 cm^–1^, which is close to the energy barrier of 24.2 cm^–1^ obtained by *ac* magnetic measurements.

**Figure 44:**
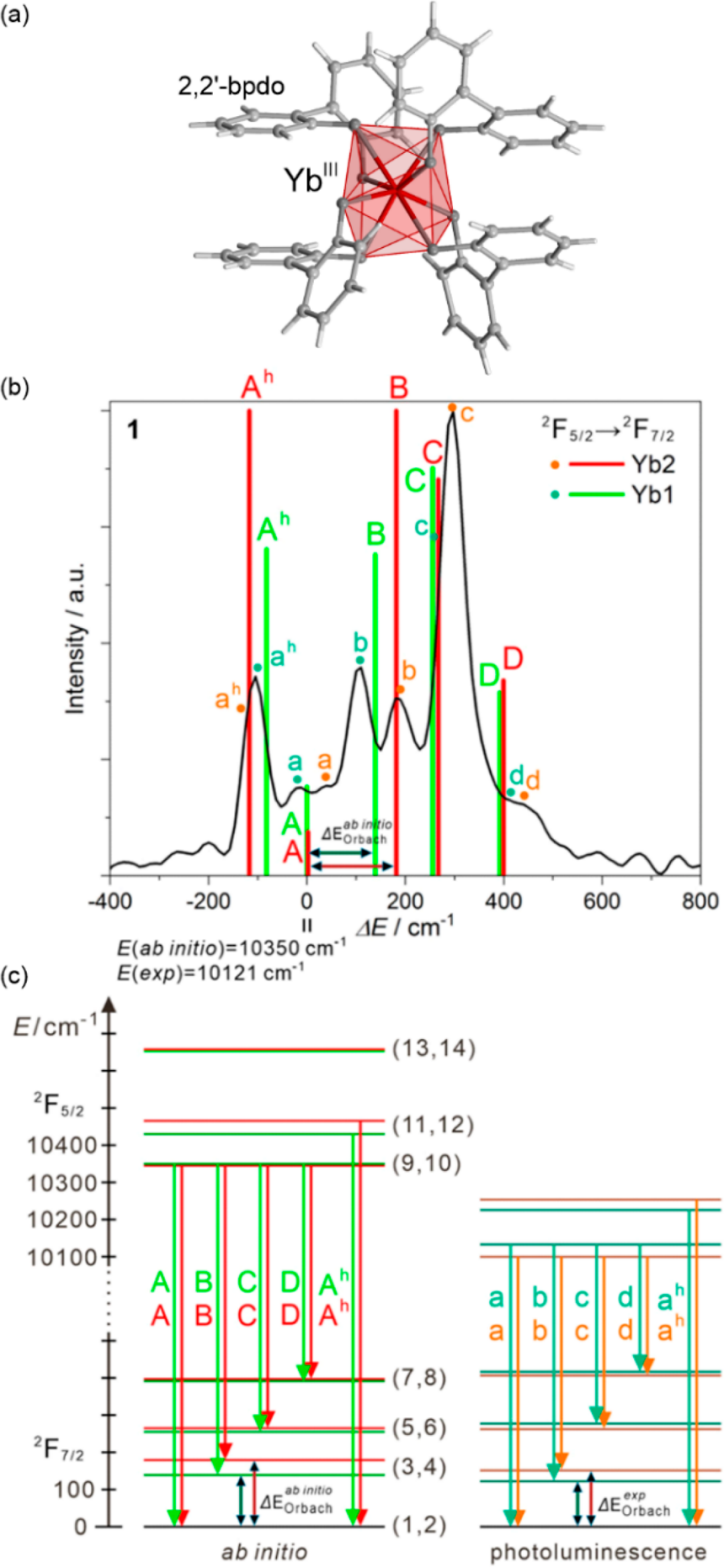
The structure of [Yb^III^(2,2′-bpdo)_4_][Ag^I^(CN)_2_]_3_·(solvent) (2,2′-bpdo = 2,2′-bipyridine *N*,*N*′-dioxide) supramolecular framework (only the Yb(III) molecular cation revealing a field-induced SMM behavior was shown) (a),^789^ its high-resolution emission spectrum at 80 K for the 325 nm excitation, shown with the cumulative oscillator strengths (colored bars) for two crystallographically independent Yb1 and Yb2 sites, obtained from the *ab initio* calculations (b), and the comparison of the energy level diagrams obtained from the *ab initio* approach and the experimental emission spectra (photoluminescence) (c). Parts (b) and (c) were adapted with permission from ref (789). Copyright 2021 American Chemical Society.

Many studies on magneto-optical correlations were demonstrated for Yb(III) complexes showing pronounced emission in the NIR range.^770,789,816^ K. S. Pedersen et al. used the high-resolution emission spectra related to characteristic ^2^F_5/2_→^2^F_7/2_ electronic transition to define the energy barrier between the ground and first excited electronic doublet in mononuclear [Yb^III^(trensal)] (H_3_trensal is 2,2′,2″-tris(salicylideneimino)triethylamine) complex.^816^ They found out that the first excited electronic doublet was placed nearly 500 cm^–1^ above the ground one. This result is inconsistent with the thermal energy barrier (38 cm^–1^) obtained by fitting magnetic relaxation times of a thermally activated Orbach process. This difference confirms that QTM, Raman, and direct processes constitute the main relaxation pathways in this system and the relaxation dynamics cannot be described by the Orbach relaxation process. A more complex methodology based on the *ab initio* calculations confronted with experimental magnetic and luminescent data for investigation of NIR-emissive molecular nanomagnets was shown for a series of [Yb^III^(2,2′-bpdo)_4_]^3+^ (2,2′-bpdo = 2,2′-bipyridine *N*,*N*′-dioxide) complexes embedded in the crystal lattices with diverse cyanido/thiocyanidometallates (Figure 44).^789^ The theoretical calculations were used to determine the character of Yb^III^ electronic ground states, as well as magnetic easy-axis with tunable axiality within the series. Moreover, they gave insight into the energy scheme both for the ground multiplet as well as for the emissive level, which leads to the successful reproduction of the emission spectra (Figure 44bc). These results made it possible to find the 0–0 emission line and detect the hot bands. This was extremely helpful for reliable magneto-luminescent correlations including the determination of energy gaps between two lowest-lying Kramers doublets. The significant differences between the barriers determined by the two experimental methods (*ac* magnetism and photoluminescence) indicate that the SMM behavior in the whole series is dominated by the phonon-assisted Raman relaxation. This example shows the great potential of using the *ab initio* calculations for the elucidation of magneto-luminescent properties of Yb^III^ complexes.^789^

### 4.6 Magnetic Field Control over Luminescence in Molecular Materials

The modulation of luminescence in functional solids through external physical stimuli, like mechanical strain or force, pressure, and electric or magnetic field, has gained great interest in materials science.^841−843^ In recent years, much attention has been paid to magnetic control over emission, in particular, luminescence modulation by a pulsed magnetic field which holds the promise of developing new magneto-optical technologies for magnetic field detection, aircraft guidance, and high-accuracy communication.^844,845^ Successful magneto-optical interaction is expected in materials with optimized magnetic and optical properties where the magnetic nature of the system makes it more responsive to an external magnetic field, especially in the context of their optical responsivity. This was studied in some hybrid materials,^846−849^ and simultaneously the studies on biological effects influenced by an external magnetic field were presented.^850,851^

An unexpectedly small scientific effort was devoted to research on magnetic field impact on luminescence in materials based on transition metal ions and their complexes.^852,853^ On the other hand, promising candidates for the significant interaction between a magnetic field and light emission were found among lanthanide ions, including their coordination compounds.^842−845^ Magnetic field splits electronic energy levels and shifts their energies by the Zeeman effect, directly influencing the energy positons of emission components which is particularly visible for lanthanide luminescence characterized by sharp peaks. Magnetic field affects also energy transfer routes which generate the influence over the emission intensity and other spectroscopic parameters.^842−847,854^ This opens the pathway for optical sensing of magnetic field and, on the other hand, magnetic switching of luminescence. In this context, functional solids with lanthanide dopants such as Eu^3+^, Yb^3+^, or Er^3+^ ions, have attracted considerable attention.^843,854^ Many approaches were developed to engineer and control the energy transfer processes in these materials which enables the modulation of the emission color, oscillation strength, and photoconversion efficiency by, e.g., changing the host matrix. In such systems, it was shown that optical phenomena can be tuned by a magnetic field, including, e.g., their emission intensity and lifetime.^855−858^ Moreover, examples of related optical sensors able to remotely probe the magnetic field strength were reported.^844,845,859,860^

**Figure 45:**
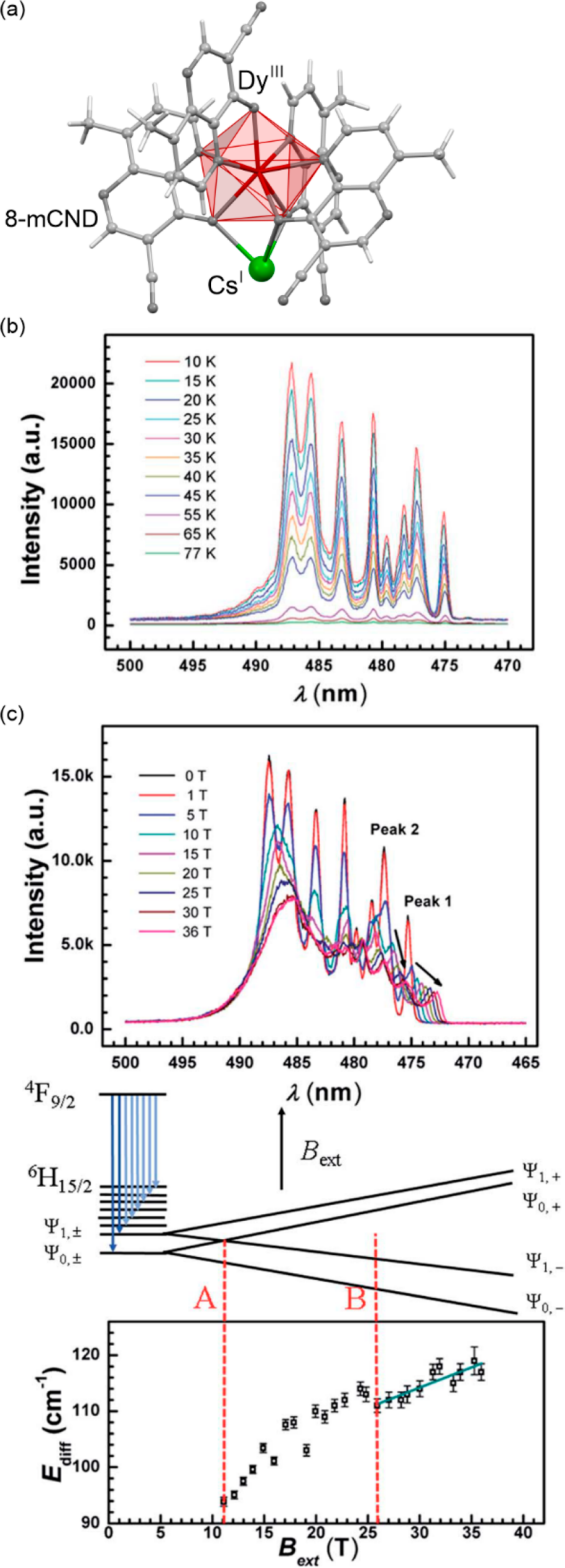
The structure of [Cs^I^Dy^III^(8-mCND)_4_(MeOH)(Me_2_CO)]·2(Me_2_CO) (8-mCND = the anion of hydroxy-8-methyl-1,5-naphthyridine-3-carbonitrile ligand) molecule (a),^831^ its UV-light-induced emission spectra measured in the 10–77 K range, representing the ^4^F_9/2_→^6^H_13/2_ electronic transition of Dy(III) centers (b), and the analogous emission spectra gathered at 5 K under a pulsed magnetic field up to 36 T, recorded in the 465–500 nm range, and shown together with a diagram of the energy splitting related to the Zeeman effect (c). Parts (b) and (c) were reproduced from ref (831) with permission from the Royal Society of Chemistry.

The development of luminescent lanthanide SMMs (see sections 4.3–4.5), which directly combine magnetic and optical properties at the molecular level, opened the interest in examining the expected magnetic field control over their photoluminescent features. As the Zeeman effect is postulated to be much smaller than the crystal field and spin-orbit coupling phenomena in lanthanide SMMs, the strong magnetic field, usually a pulsed high field, has to be employed to detect the significant opto-magnetic interaction. The related pioneering studies have been recently presented.^808,819,831,837^ For the first time, photoluminescence spectra under a magnetic field for lanthanide SMMs were investigated by Y. Bi et al.^831^ They prepared a series of molecular materials incorporating [A^I^Dy^III^(8-mCND)_4_(MeOH)(Me_2_CO)]·2(Me_2_CO) (8-mCND = the anion of hydroxy-8-methyl-1,5-naphthyridine-3-carbonitrile ligand) with various alkali metal ions (A) (Figure 45). This series behaves as single-molecule magnets under a zero *dc* field with an effective anisotropic energy barrier (*U*_eff_) of ca. 95 cm^–1^ and exhibits visible photoluminescence, both properties originating from the Dy^3+^ ion. Under a weak external magnetic field below 0.6 T, the Dy^III^ emission peaks are superimposed to the zero magnetic field ones (with estimated Zeeman splitting energy less than 2.8 cm^–1^) indicating a rather small influence of weak magnetic field on the emission and the related energy gaps between *m*_J_ levels which are crucial in the SMM characteristics. Under a strong magnetic field up to 36 T, a blue shift of the emission peaks is observed, demonstrating the increased role of the Zeeman effect. Interestingly, emission peaks of longer wavelengths become strongly mixed and change into an unstructured band, whereas two emission peaks of shortest wavelengths remain distinguishable from the others. The authors analyzed the Zeeman effect which could be directly derived from the luminescence spectra by subtracting the position of these two well-resolved peaks that excludes the effect of the ground state.^831^

The magnetic-field-induced splitting of the spectral lines arising from the Zeeman effect on the Kramers doublets into two components was also shown by R. A. S. Ferreira et al. for the luminescent SMM, [Dy^III^(acac)_3_(H_2_O)_2_]·H_2_O (acac = acetylacetonate).^819^ Using pulsed magnetic fields, the Dy(III) emission spectrum was measured as a function of the external magnetic field at a constant temperature, and the number and energy of the emission components depend strictly on the magnetic field. Moreover, this complex could be used as a luminescent magnetic field sensor, and it behaves as a luminescent molecular thermometer operating between 10 and 290 K (at *H* = 0 T) and between 4 and 180 K (for *H* up to 45 T). In contrast to the examples in which the effect of the magnetic field influence on luminescence was observed, this system represents the first optical sensor allowing the dual and synchronous measurement of the temperature and magnetic field using a single photoluminescence spectrum. Moreover, these examples show that luminescence spectroscopy under a strong magnetic field can be considered a novel complementary method to study the lowest magnetic sublevels and give insightful information on SMMs, often difficult to obtain from magnetic or EPR measurements.^808,819,831,837^

### 4.7 Optically Addressable Molecular Qubits

A quantum bit, named qubit, is the fundamental unit for the promising realization of a wide range of quantum technologies including single-photon generation, quantum metrology, and quantum information protocols.^861−865^ In all of these applications, a generalizable readout strategy is needed. Optically addressable solid-state spin systems provide an attractive framework for the readout of molecular systems, for which photon collection efficiency is a key figure of merit.^866^ The well-studied optically addressable quantum qubit candidates are the defect-based qubits in wide bandgap semiconductor materials, like a nitrogen-vacancy center in diamond,^864,867,868^ transition metal dopants (chromium or vanadium) in silicon carbide,^869^ or rare-earth ions in garnet host crystals,^870^ which have demonstrated an optical initialization and readout mechanism using resonant excitation. However, defect-based qubits are restricted to their host materials, limiting their tunability, scalability, and integration into hybrid architectures.^871,872^ The solution seems to be accessible using coordination chemistry, in which nuclear spins can be controllably placed around a molecular qubit,^873^ arrays of spins can be created in 1-, 2- and 3-dimensional coordination architectures,^874^ and molecular spins can be conveniently integrated into electronic and photonic devices.^875,876^ These capabilities provide remarkable control over the intrinsic and extrinsic environment of molecular qubits. Creating such an interface in a molecular platform would generate a class of qubits that can be engineered with atomic precision, with potentially transformative applications for quantum technologies.

**Figure 46:**
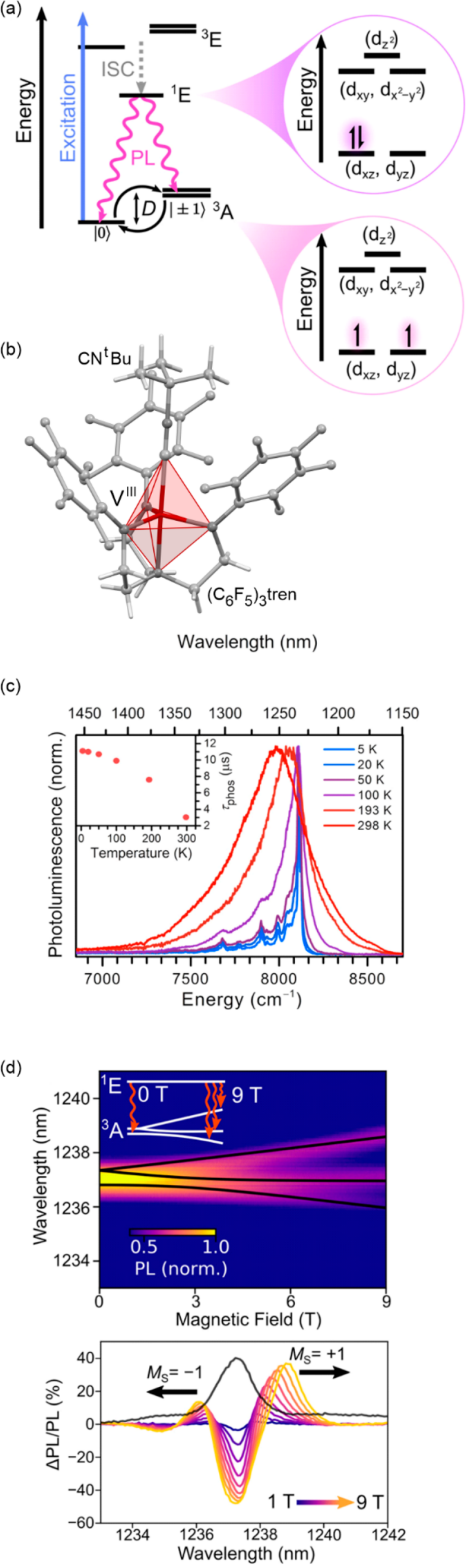
Excited-state manifold for the *S* = 1 metal complex in an ideal *C*_3_ symmetry with qualitative orbital splitting diagram and electronic configurations for the ^1^E and ^3^A states (a), the structure of [V^III^((C_6_F_5_)_3_tren)(CN^t^Bu)] ((C_6_F_5_)_3_tren = 2,2′,2′′-tris[(pentafluorophenyl)amino)triethylamine; CN^t^Bu = tert-butyl isocyanide) complex being the representative of the *S* = 1 system (b),^877^ its variable-temperature luminescence spectra and emission lifetimes (c), and the variable-magnetic-field emission spectra, shown with the simulated Zeeman splitting and differential emission (in relation to the case without external field) (d). Parts (a), (c), and (d) were adapted with permission from ref (877). Copyright 2020 American Chemical Society.

The spin-selective optical initialization and readout in molecular qubit candidates based on metal complexes can be realized by the application of luminescence under a magnetic field (see also the related section 4.6. above). To realize optical read-out, an *S* = 1 ground state featuring weak zero-field splitting is a convenient platform to enable microwave-based manipulation, coupled with a strong-field ligand to ensure the lowest-lying excited state of a singlet type that radiatively decays to the ground state. Several metal ions and their oxidation states support an *S* = 1 ground state,^866^ among which the V^3+^-containing coordination complexes reveal two crucial attributes. Vanadium features an intrinsic nuclear spin of 7/2 that may serve as a long-lived quantum memory.^877^ Moreover, V^III^-based molecular systems can exhibit photoluminescence close to the telecommunication band, i.e., in the range of 1260–1625 nm. Within the broad scope of V^3+^-containing compounds that yield the desired triplet ground state, M. S. Fataftah et al. synthesized a trigonal bipyramidal compound, [V^III^((C_6_F_5_)_3_tren)(CN^t^Bu)] ((C_6_F_5_)_3_tren = 2,2′,2′′-tris[(pentafluorophenyl)amino)triethylamine; CN^t^Bu = tert-butyl isocyanide) (Figure 46).^877^ The combination of a trivalent oxidation state of vanadium and the *C*_3v_ symmetry results in the ^3^A ground state, where the frontier orbitals include two degenerate, singly occupied molecular orbitals with sufficiently small zero-field splitting to achieve EPR-based addressability. The employed ligands engender a strong ligand field environment around the metal ion, such that the lowest-lying excited state is the ^1^E term, and the resulting ^1^E→^3^A electronic transition is radiative. Moreover, the ground-state spin sublevels could be resolved in the emission spectra. At 3 K, this compound reveals an emission around 1237 nm, comparable to a zero-field splitting parameter, *D*, determined from the EPR measurements. Under an applied magnetic field, Zeeman splitting of the *M*_S_ sublevels was observed where emission is resolved into each ground-state spin sublevel in the photoluminescence spectrum at the high field. Its resolution illustrates that, under an applied magnetic field, this system would be promising for spin-selective optical excitation, thus its analogs have the potential as optically addressable molecular qubit candidates.^877^

**Figure 47:**
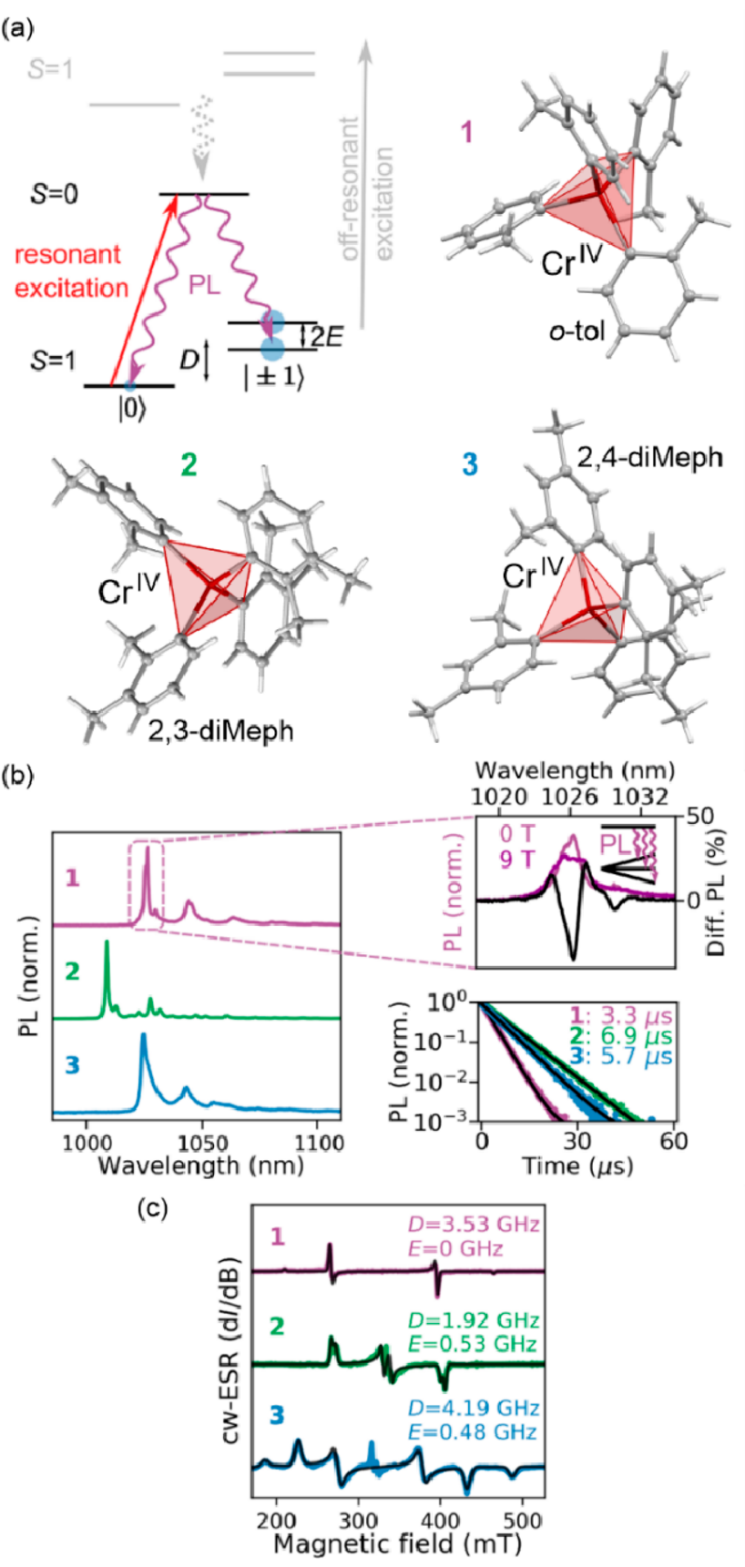
The energy-level diagram of high-symmetry Cr(IV) complexes depicting their possible photoluminescence, shown together with the [Cr^IV^(L-aryl)_4_] complexes of this type, including the systems with three different aryl ligands, L-aryl (o-tol = *o*-tolyl, 2,3-diMeph = 2,3-dimethylphenyl, and 2,4-diMeph = 2,4-dimethylphenyl) (a),^878^ low-temperature luminescence spectra of these complexes, presented together with their Zeeman splitting under the 0 and 9 T external magnetic fields (b), and the related X-band continuous-wave electron spin resonance spectra collected at 77 K (c). Reproduced with permission from ref (878). Copyright 2020 American Association for the Advancement of Science.

Another example of coordination-complex-based molecular qubits with an optically addressable ground-state spin was shown by S. L. Bayliss et al. in a series of organometallic chromium(IV) molecules (Figure 47).^878^ They selected a Cr^4+^ ion coordinated by strong-field aryl ligands in the [Cr^IV^(L-aryl)_4_] complexes which differ by the placement of a single methyl group within coordinated ligands (L-aryl = *o*-tolyl, 2,3-dimethylphenyl, 2,4-dimethylphenyl). They also focused on the isostructural Sn(IV)-diluted analogs reducing interactions between Cr(IV) centers. The d^2^ electronic configuration of the Cr^4+^ ion placed in a pseudo-tetrahedral environment produces a triplet (*S* = 1) ground state with small zero-field splitting. This configuration leads to narrow optical transitions between the *S* = 1 ground state and the *S* = 0 excited state. This, when combined with the ground-state zero-field splitting, enables optical spin readout and initialization through spin-selective resonant excitation. Under off-resonant excitation (785 nm), the ground-state population is promoted to the first *S* = 1 excited state, then undergoes fast intersystem crossing to the *S* = 0 state, and decays to the *S* = 1 ground state, emitting near-infrared light for all reported compounds. However, even minor ligand modifications in these systems result in a unique ground-state spin structure with slightly different splitting values determined from electron spin resonance measurements. The energy level structure was confirmed by measuring the emission under a high magnetic field using off-resonant excitation. Owing to the *S* = 0 excited state, the Zeeman splitting for the ground state manifests directly as a shift in optical emission energies. This effect is clearly shown by taking the difference in photoluminescence spectra at 9 and 0 T; optical emission into the spin sublevels shifts to lower and higher energies, giving characteristic peaks in the differential spectrum (Figure 47).^878^

The catalog of qubit materials was also expanded toward Ni^2+^-based coordination compounds because Ni(II) centers in octahedral symmetry meet all the criteria for optical readout of spin. M. K. Wojnar et al. presented two Ni(II) metal complexes, [Ni^II^(phen)_3_](BF_4_)_2_ and [Ni^II^(pyr_3_)_2_](BF_4_)_2_ (phen = 1,10-phenanthroline; pyr_3_ = tris-2-pyridylmethane), exhibiting weak zero-field splitting that enables EPR addressability.^879^ Ni(II) centers are typically considered ″EPR-silent″; however, a high-symmetry octahedral coordination environment supports the small zero-field splitting values required for spin manipulation. The strong field ligands enable emission in the 938–944 nm range, satisfying the emissive readout criterion for an optically addressable molecular qubit.

## 5 Chirality- and Non-Centrosymmetricity-Related Optical Effects in Molecule-Based Magnetic Materials

Chirality is a frontier research theme in chemistry and biology. Purposefully tailored chiral molecules can exhibit exceptional functionalities, such as chiral sensing, chiral catalysis, and chiral pharmaceutics.^880−882^ In physics, chirality has also been pivotal in uncovering various novel phenomena.^883,884^ For instance, the selective interaction between chiral molecules and electron spin gave rise to an effect called chirality-induced spin selectivity (CISS), opening new avenues in spintronics and molecular quantum devices.^885,886^ Owing to the optical activity of chiral material systems depicted by the circular dichroism (CD) effect,^887,888^ coupling of chirality with magnetism is achievable and leads to magneto-optical interplay, called magneto-chiral dichroism (MChD), which, e.g, enables the optical readout of magnetization states.^889,890^ The other chiroptical properties, such as optical rotatory dispersion (ORD) or circularly polarized luminescence (CPL), are also intensively investigated due to their application potential in advanced sensors, lighting display technologies, and information processing.^891−893^

On the other hand, non-centrosymmetricity in crystalline solid-state materials, which is closely related to chirality, can offer nonlinear optical (NLO) processes, such as second and higher-order harmonic generation (SHG and others), two-photon absorption, or the optical Kerr effect, which are applicable in photonic devices, such as laser systems or sensors.^200−202,207,894−896^ Some of them were combined with magnetic properties, for instance, the SHG activity with magnetic ordering provides the route to magnetization-induced SHG (MSHG).^209^ When special cases of non-centrosymmetric space groups are achieved, that is the polar ones, the next group of physical effects, including piezoelectricity, pyroelectricity, and ferroelectricity, is also accessible.^897−899^ Their application potential is broad, e.g., in memory devices, sensors, or actuators,^898,900,901^ and their strong correlation with magnetism towards multiferroicity was explored.^902,903^

Taking into account the extensive set of chirality- and non-centrosymmetricity-related physical functionalities, especially those optical ones, it was found highly desirable to search for novel materials that can form chiral structures as well as crystallize in non-centrosymmetric space groups.^121,897,904−908^ This quest was primarily focused on inorganic solids, such as metal oxides;^904,905^ however, molecular materials based on metal complexes and/or organic molecules offer broader possibilities for the rational design of chirality and symmetry breaking as they are constructed using a molecular building blocks approach which explores an almost limitless scope of molecular precursors, including chiral ones.^121,897,906−908^ In this regard, molecule-based magnetic materials share this great structural tunability, moreover, providing the promise for the fruitful interplay between magnetism and chirality/non-centrosymmetricity-related optical phenomena as indicated by the above-mentioned MChD or MSHG coupling effects.^209,889,890^

This section will discuss, firstly, design principles and advances in the implementation of chirality and symmetry breaking towards structural non-centrosymmetricity of various groups of molecule-based magnetic materials, including magnetically ordered systems (section 5.1), spin transition materials (section 5.2), and molecular nanomagnets (section 5.3). Subsequent sections are devoted to the discussion of two main, broadly recognized types of optical phenomena embedded into molecular magnetic systems, including circular dichroism (CD) effects (natural and magnetic ones) and magneto-chiral dichroism (MChD) phenomenon (section 5.4), as well as second harmonic generation (SHG) (section 5.5). More advanced combinations of chirality and non-centrosymmetricity, in particular polarity, with photoswitching effects or luminescence in molecule-based magnetic systems will be discussed separately in section 7.

### 5.1 Design of Chiral and Non-Centrosymmetric Molecule-Based Magnets

**Figure 48:**
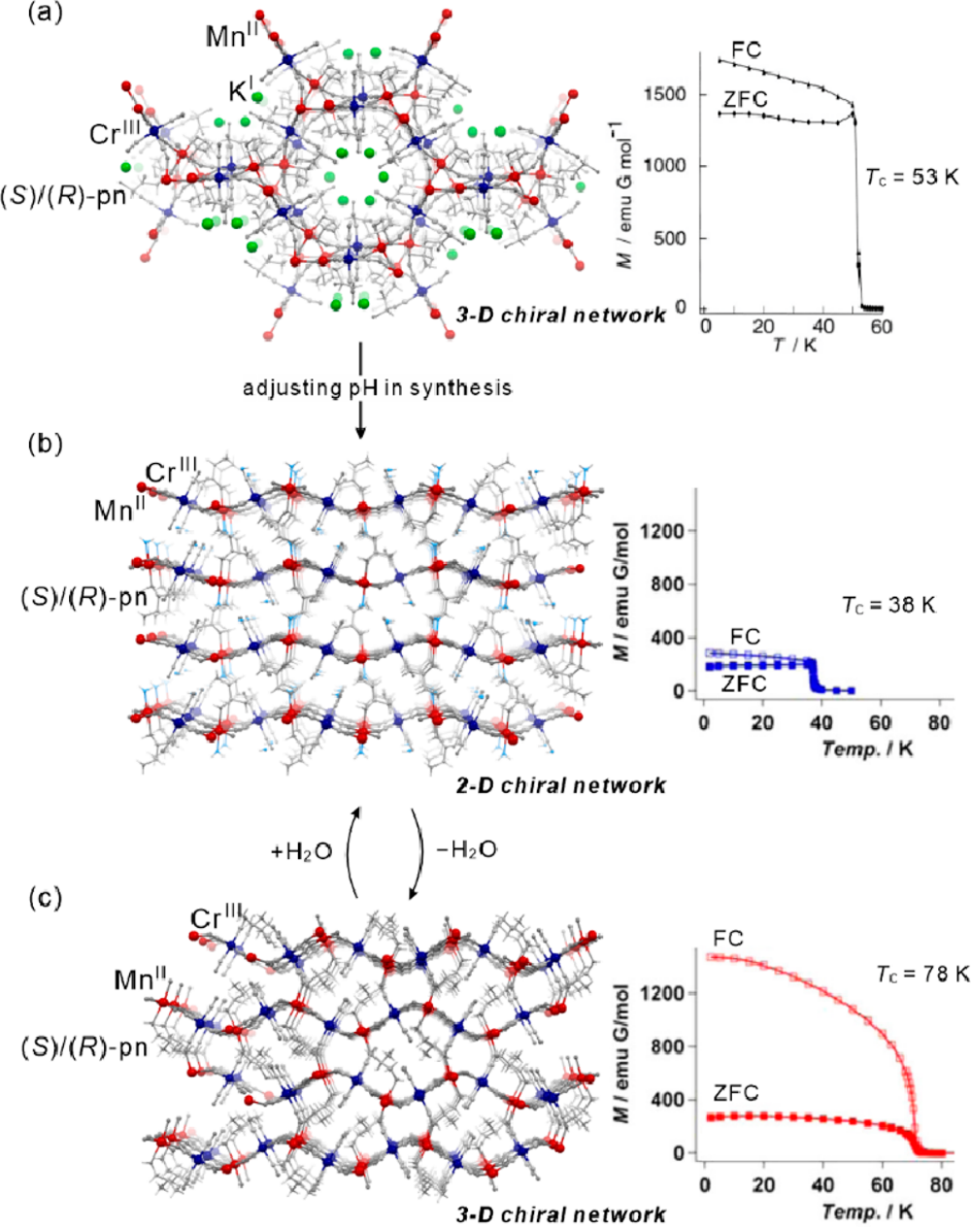
Representative structural views (left panel) and field-cooled (FC) with zero-field-cooled (ZFC) magnetic curves (right panel) of three chiral Mn^II^–[Cr^III^(CN)_6_]^3–^ molecule-based ferrimagnets with (*S*)- or (*R*)-1,2-diaminopropane (pn) ligands, including 3-D K^I^_0.4_((*S*)-pnH)_0.6_{[Mn^II^((*S*)-pn)][Cr^III^(CN)_6_]} network obtained from a slightly basic solution (a),^910^ layered {[Mn^II^((*R*)-pnH)(H_2_O)][Cr^III^(CN)_6_]}·H_2_O framework, obtained by adjusting the solution to relatively acidic pH (b),^235^ and the dehydration-induced 3-D {[Mn^II^((*R*)-pnH)][Cr^III^(CN)_6_]} framework (c).^911^ Part (a) was adapted with permission from ref (910). Copyright 2001 John Wiley & Sons. Parts (b) and (c) were adapted with permission from ref (911). Copyright 2016 American Chemical Society.

Achieving long-range magnetic ordering below the critical temperature (*T*_c_) in molecule-based materials requires efficient exchange interaction between magnetic centers, usually ensured by molecular bridges linking these spin centers within a two- or three-dimensional coordination framework.^31−38,909^ Cyanido-bridged heterometallic d–d coordination assemblies, e.g., Prussian blue analogs (PBAs), are one of the representatives of the realization of molecule-based magnets showing relatively high critical temperatures of long-range magnetic ordering.^29,36,37^ Moreover, within this group of materials, the implementation of enantiomorphic or (at least) bulky organic ligands can rationally introduce chirality and/or non-centrosymmetricity which provides an effective approach toward chiral and non-centrosymmetric molecule-based ferro- and ferrimagnets.^121,909^ Early efforts in these regards were made by K. Inoue et al. who reported in 2001 a three-dimensional Mn^II^–[Cr^III^(CN)_6_]^3–^ network incorporating chiral (*S*)-1,2-diaminopropane ligands ((*S*)-pn), which exhibits ferrimagnetism with *T*_c_ = 53 K (Figure 48a).^910^ Initially, the (*S*)-pn ligand coordinated to Mn(II) centers was deprotonated due to the basic synthesis condition, i.e., pH of 7–8. However, if the pH of the reaction solution was adjusted to ca. 6–7, a fully protonated (*S*)-pnH ligand was formed which resulted in a two-dimensional chiral Mn^II^–[Cr^III^(CN)_6_]^3–^ coordination polymer, showing ferrimagnetism with a decreased *T*_c_ of 38 K (Figure 48b).^235^ More recently, the *R*-enantiomer of this chiral layered framework was reported to exhibit a dehydration-driven structural transformation into a 3-D coordination network. The resulting crystalline phase reveals the *T*_c_ increased to 73 K. The dehydration effect can be reversibly switched back while maintaining the non-centrosymmetric *P*2_1_2_1_2_1_ space group (Figure 48c).^911^ Similar approach utilizing cyanido-bridged assemblies was also done by E. Coronado et. al. who synthesized enantiomorphic layered Ni^II^–[Fe^III^(CN)_6_]^3–^ ferromagnets with *T*_c_ = 14 K by utilizing a chiral *trans*-(1*S*, 2*S* or 1*R*, 2*R*)-cyclohexane-1,2-diamine ligand.^912^ The slightly decreased *T*_c_ of 11 K was found for the analog obtained using the *cis*-isomer.^913^

**Figure 49:**
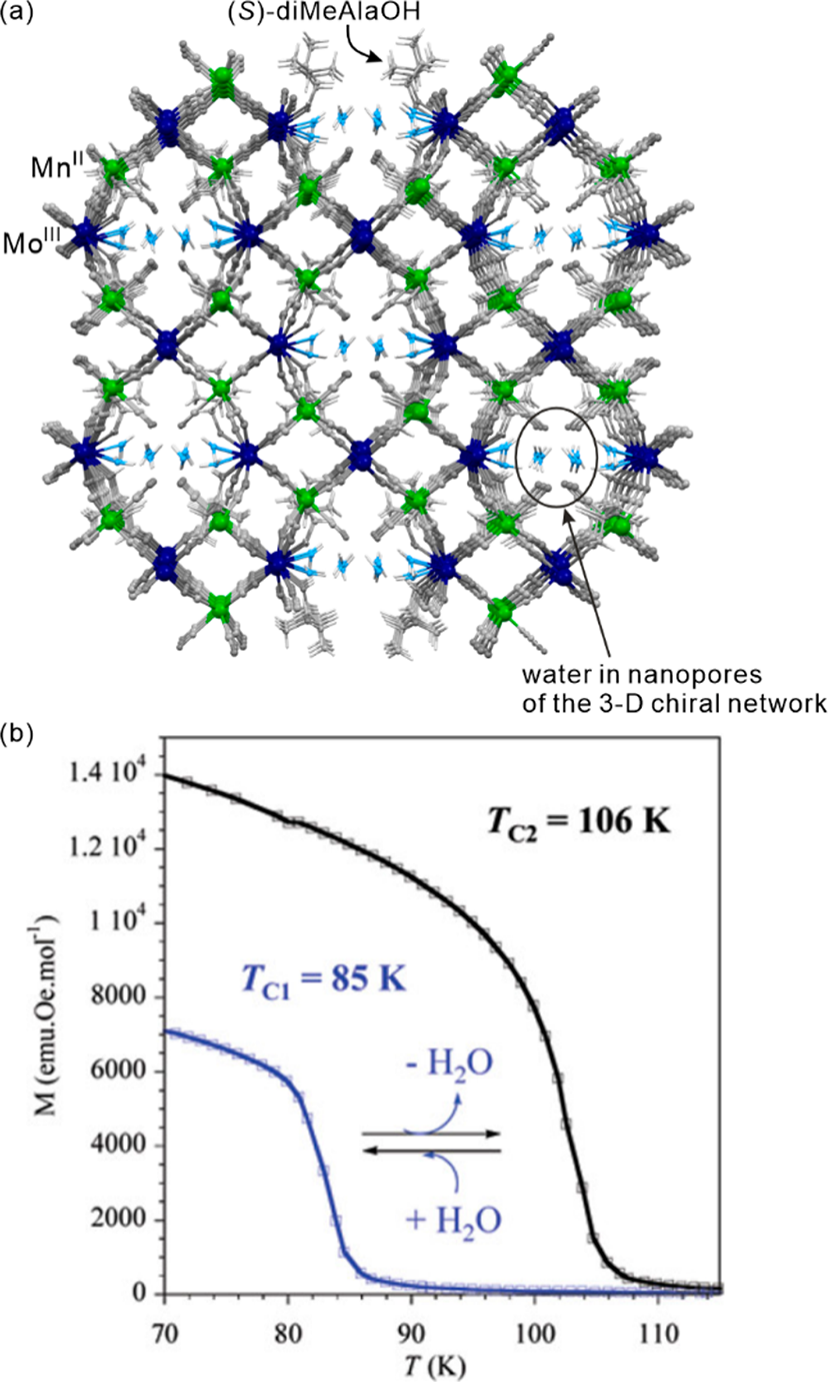
Structure of 3-D chiral {[Mn^II^((*S*)-diMeAlaOH)(H_2_O)]_2_Mn^II^[Mo^III^(CN)_7_]_2_}·2H_2_O ((*S*)-diMeAlaOH = (*S*)-*N*,*N*-dimethylalaninol) coordination network with indicated water molecules occupying the structural nanopores (a)^919^ and the temperature dependences of molar magnetization for the as-synthesized water-containing framework and their dehydrated phase, shown with the indicated values of the critical temperatures of related magnetic phase transitions (b). Part (b) was adapted with permission from ref (919). Copyright 2007 American Chemical Society.

Apart from the hexacyanidometallate-based coordination networks, other paramagnetic cyanido metal complexes, such as heptacyanido- or octacyanidometallates were also employed for achieving enantiomorphic molecule-based ferri-/ferromagnets.^914−919^ A noteworthy example is based on a 3-D nanoporous Mn^II^–[Mo^III^(CN)_7_]^4–^ network in which the chirality is induced by (*S*)- or (*R*)-*N*,*N*-dimethylalaninol ligands leading to the crystallization in a non-centrosymmetric *C*2 space group (Figure 49).^919^ Thanks to the robust nanoporous structure, crystallization water molecules can be thermally and reversibly removed which is accompanied by the switching of the *T*_c_ value between 85 K and 106 K.

**Figure 50:**
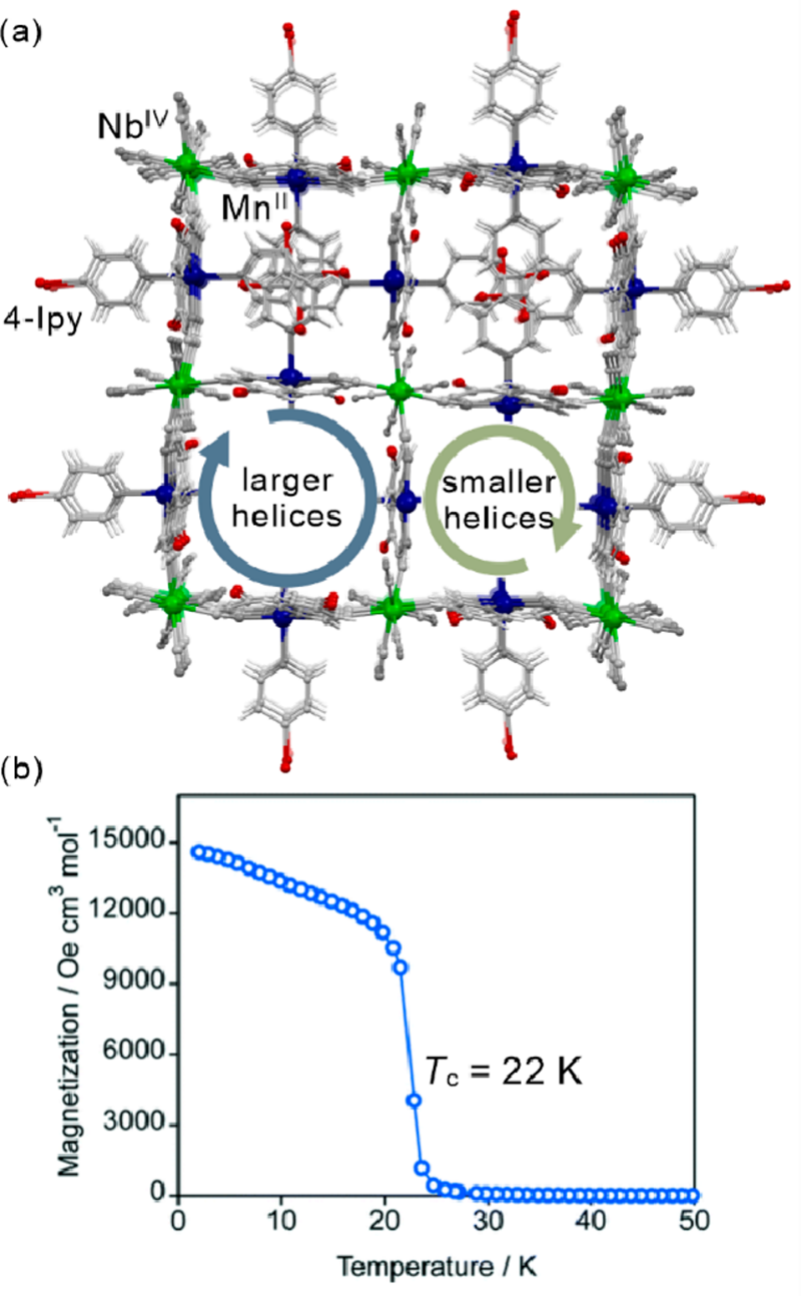
The structure of 3-D chiral {[Mn^II^(4-Ipy)_4_]_2_[Nb^IV^(CN)_8_]} (4-Ipy = 4-iodopyridine) coordination network with the indicated two types of helical structural channels (a)^921^ and its temperature dependence of molar magnetization with the indicated value of the critical temperature of the magnetic phase transition (b). Part (b) was reproduced from ref (921) with permission from the Royal Society of Chemistry.

Besides the usage of a chiral ligand, spontaneous resolution of enantiopure molecule-based magnets can be frequently generated in cyanido-bridged coordination systems due to the steric effects of an applied organic ligand which is often supported by relatively irregular coordination topologies of polycyanidometallate-based frameworks.^143,920,921^ For instance, by incorporating 4-halogen-substituted pyridine achiral ligands, 4-Xpy (X = Br and I) into octacyanidoniobate(IV)-containing bimetallic frameworks, T. Ohno et al. were able to form an enantiopure Mn(II)–Nb(IV) ferrimagnet (*T*_c_ = 28 K) bearing the 4-Brpy derivative,^920^ and the other Mn(II)–Nb(IV) ferrimagnet with *T*_c_ = 22 K that incorporates larger 4-Ipy derivative (Figure 50).^921^ Their chirality stems from the size inequality between left-handed and right-handed helices appearing within cyanido-bridged coordination skeleton, ascribable to non-innocent supramolecular interactions involving halogen substituents on the pyridine rings. The appearance of chirality was found to be correlated both with the size of the halogen atom as well as with the ionic radius of the examined 3d metal ion.^920,921^

Among non-cyanido coordination networks, chiral molecule-based magnets were constructed using oxalato-based bimetallic assemblies,^121,922^ molecular perovskite-type frameworks,^923^ and some other systems based on organic molecular bridges.^924^ These results opened a perspective to study various chirality-related optical phenomena, including magneto-chiral dichroism and second harmonic generation, in the magnetically ordered phases (see sections 5.4 and 5.5).

### 5.2 Design of Chiral and Non-Centrosymmetric Spin Transition Materials

**Figure 51:**
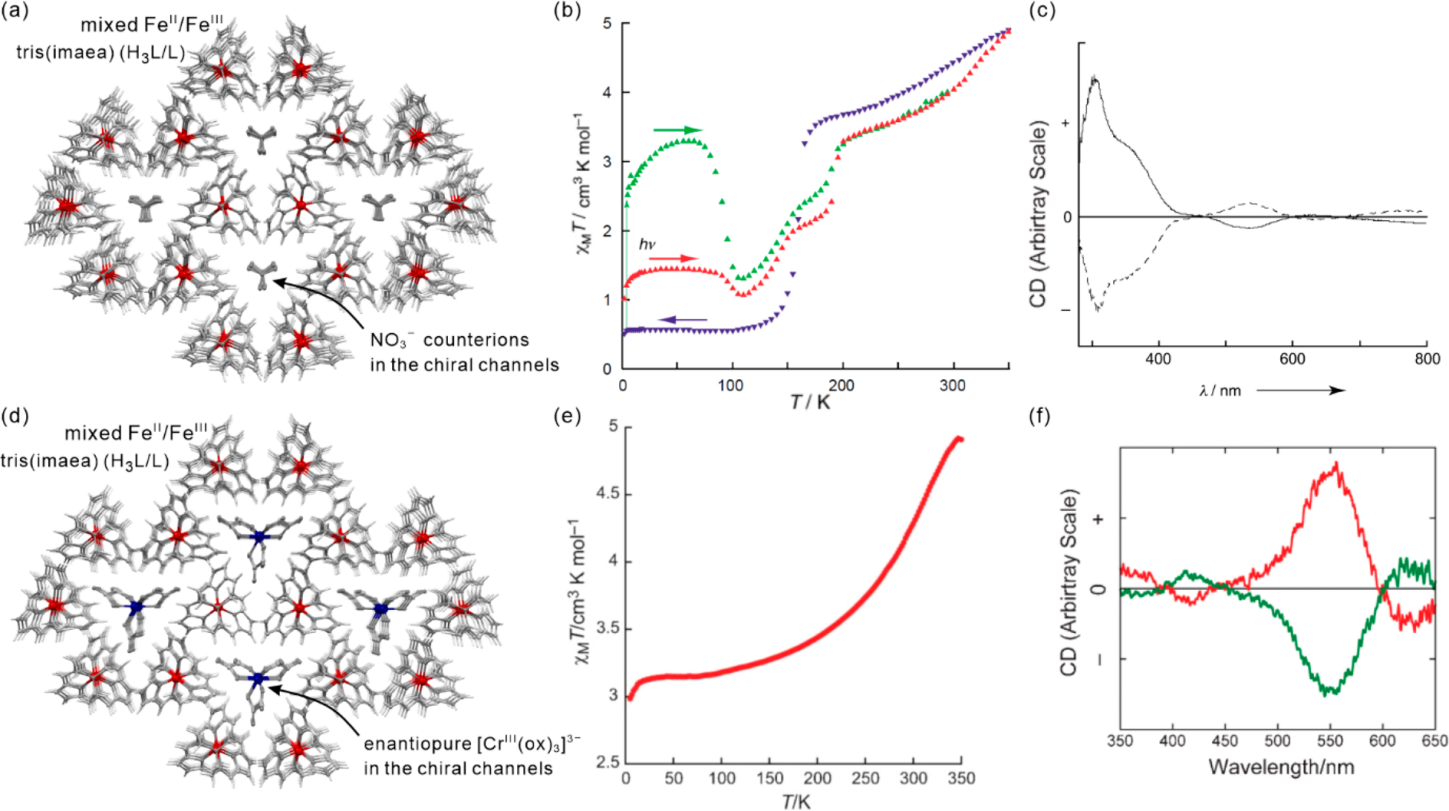
The structure of chiral [Fe^II^(H_3_tris(imaea))][Fe^III^(tris(imaea))](NO_3_)_2_ (tris(imaea) = tris{[2-{(imidazole-4-yl)methylidene}amino]ethyl}amine in the H_3_L or L^3–^ form) supramolecular framework (a),^470^ its temperature- and light-induced spin transition characteristics followed by changes in the *χ*_M_*T* product (b), and the CD spectra in KBr pellets of the selected crystals of this compound (c), the structure of analogous chiral [Fe^III^(H_3_tris(imaea))][Fe^III^(tris(imaea))][Cr^III^(ox)_3_]·3H_2_O (ox = oxalato) supramolecular network (d),^925^ its temperature dependence of the *χ*_M_*T* product (e), and the CD spectra of this compound and its enantiomeric form (f). Parts (b) and (c) were adapted with permission from ref (470). Copyright 2003 John Wiley & Sons. Parts (e) and (f) were reproduced from ref (925) with permission from the Royal Society of Chemistry.

Since most spin crossover (SCO) compounds are based on six-coordinated transition metal complexes of a distorted octahedral geometry (see section 2.1) that often naturally form two enantiopure forms of *Δ* (clockwise) or *Λ* (anticlockwise) homochiral configuration, the related chiral spin transition materials can be obtained by a spontaneous resolution process enabling the separate crystallization of these chiral forms. Such results were presented by Y. Sunatsuki et al. in 2003 who reported a mixed-valence supramolecular sheet material built of alternately connected tripod-like [Fe^II^(H_3_tris(imaea))]^2+^ and [Fe^III^(tris(imaea))]^0^ molecules where tris(imaea) is a hexadentate tris{[2-{(imidazole-4-yl)methylidene}amino]ethyl}amine ligand (Figure 51a–c).^470^ The observed spontaneous resolution is induced by the hydrogen bonding interaction favorably formed between molecules possessing the same, *Δ* or *Λ*, absolute configuration. Many different spin states of this material were accessed by temperature change (e.g., multi-step SCO) and light stimulation (e.g., LIESST effect at low *T*). Nevertheless, the chirality-related functionality was not discussed for this kind of SCO system until 2013 when the analogous 2-D supramolecular assembly containing only Fe(III) centers was reported.^925^ In this material, the chiral recognition of [Cr^III^(oxalato)_3_]^3–^ guest molecular anions can be realized in line with the homochirality of supramolecular sheets (Figure 51d–f).

**Figure 52:**
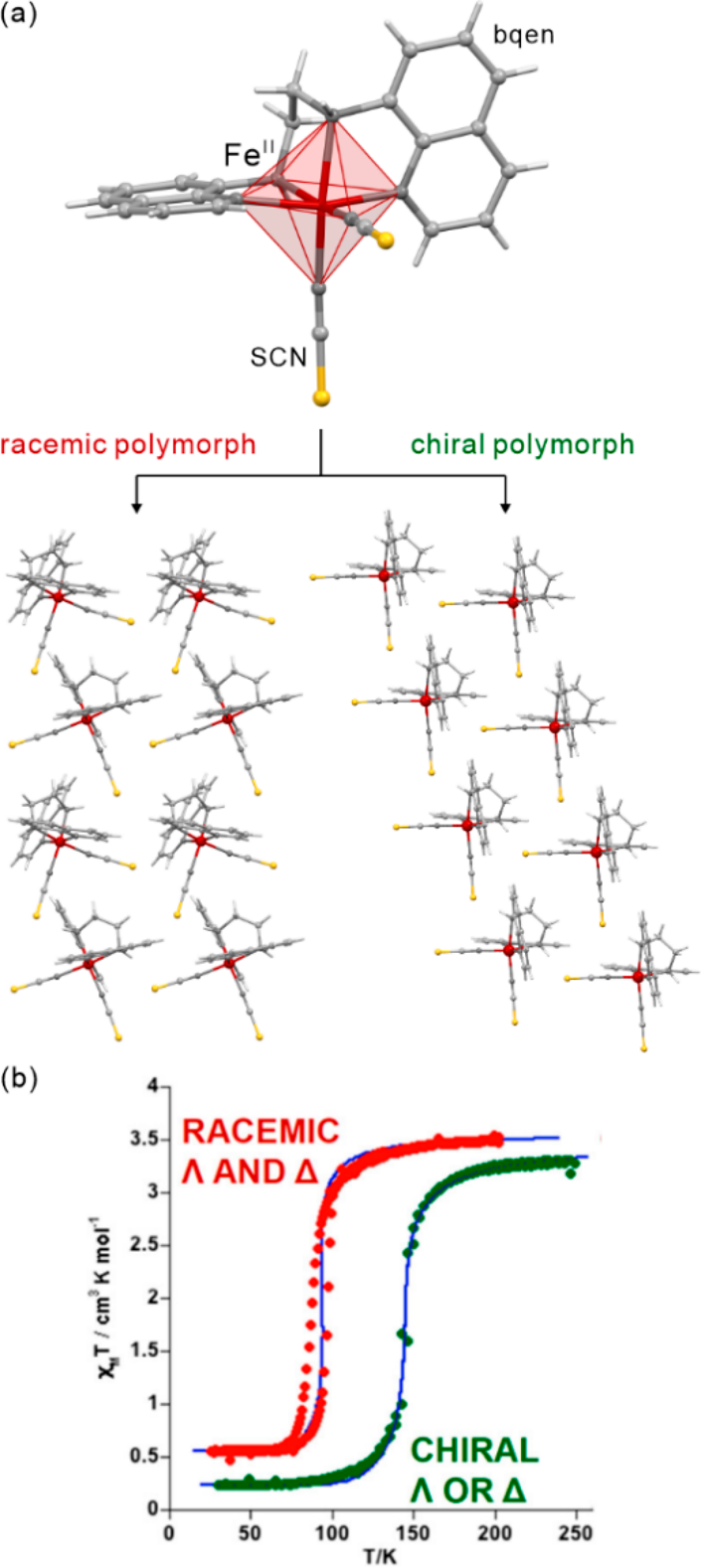
The structure of [Fe^II^(bqen)(NCS)_2_] (bqen = *N*,*N*′-bis(8-quinolyl)ethane-1,2-diamine) complex and the two types of its crystal packing, including the racemic polymorph and the chiral one (a),^928^ and their temperature dependences of the *χ*_M_*T* product, depicting the impact of the chirality on the thermal spin crossover effect (b). Part (b) was adapted with permission from ref (928). Copyright 2017 American Chemical Society.

Syntheses relying on spontaneous resolution are often accompanied by racemic byproducts. When the chiral form and the racemized phase share the same chemical component but differ in crystal packing, the effect of polymorphism becomes viable, which is an important issue in the field of SCO systems.^926,927^ C. Bartual-Murgui, J. A. Real and co-workers reported the polymorphic effect between the homochiral (either *Δ* or *Λ*) and racemized (coexistence of both *Δ* and *Λ*) phases of [Fe^II^(bqen)(NCX)_2_] complexes, where bqen is a tetradentate *N*,*N*′-bis(8-quinolyl)ethane-1,2-diamine ligand and X stands for S or Se (Figure 52).^928^ The different crystal packings between the two polymorphs lead to modified angular distortion parameters on Fe(II) complexes. Thus, in both S- and Se-containing derivatives the homochiral phase exhibits an increased spin transition temperature compared with the racemic phase.^928^

**Figure 53:**
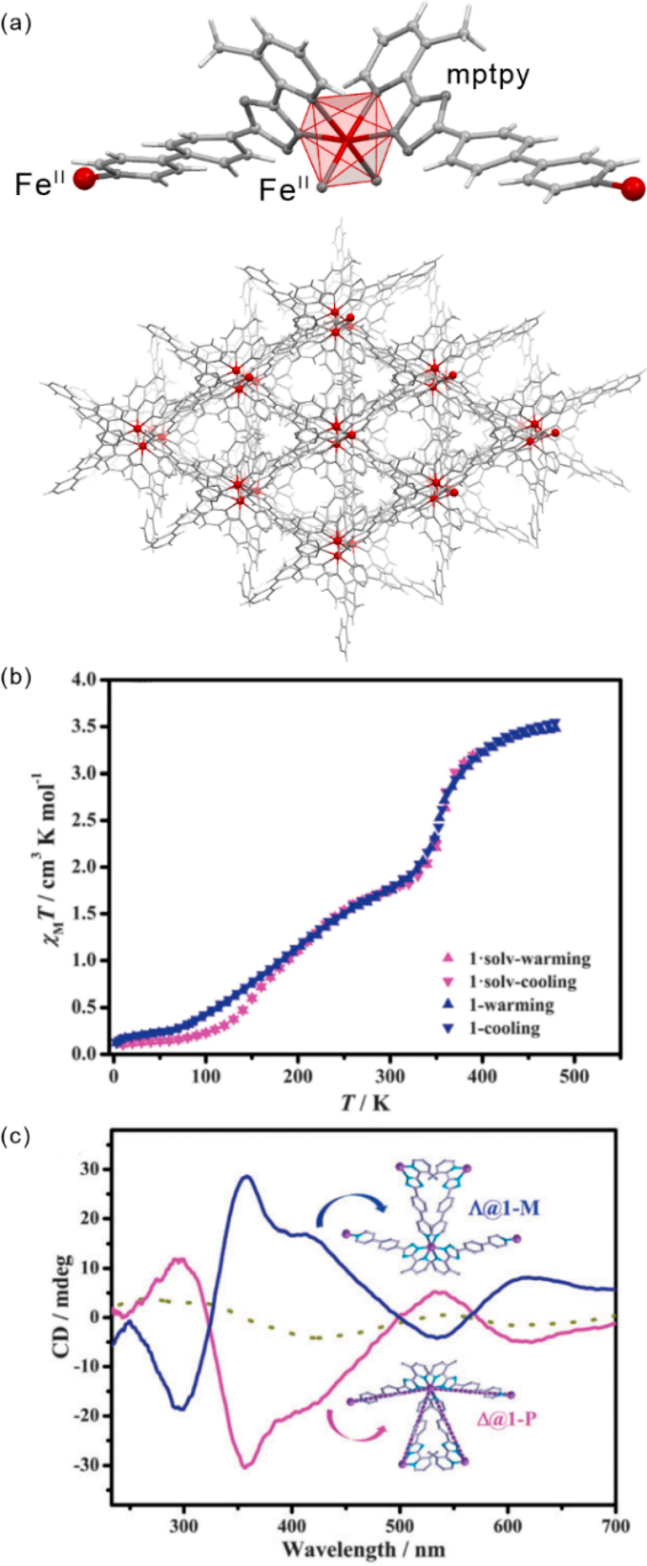
The structural views on 3-D chiral [Fe^II^(mptpy)_2_]·EtOH·0.2dmf (mptpy = 3-methyl-2-(5-(4-(pyridin-4-yl)phenyl)-4H-1,2,4-triazol-3-yl)pyridine) coordination framework showing the SCO effect (a),^929^ the temperature dependences of the *χ*_M_*T* product for its solvated and desolvated phases (b), and the CD spectra of the bulk sample (dark yellow) and selected enantiopure single crystals (navy and magenta) (c). Parts (b) and (c) were reproduced from ref (929) with permission from the Royal Society of Chemistry.

In addition to discrete complex molecules, spontaneous resolution can also be realized in extended polymeric systems. In 2014, M.-L. Tong and co-workers reported a three-dimensional MOF depicted by the formula of [Fe^II^(mptpy)_2_]·EtOH·0.2dmf, where mptpy is a deprotonated form of 3-methyl-2-(5-(4-(pyridin-4-yl)phenyl)-4H-1,2,4-triazol-3-yl)pyridine. This material exhibits a two-step gradual SCO, slightly modifiable by the desolvation process (Figure 53).^929^ The spontaneous resolution of this framework is ensured by the helical structure generated along the *c* crystallographic axis, and it was guaranteed by the asymmetric shape of the mptpy ligand which has a long monodentate tail part attached to the bidentate main body. To more straightforwardly control the homochirality or non-centrosymmetricity of SCO materials in large preparation scales, the application of chiral ligands is a more effective approach. A plethora of mononuclear SCO compounds based on chiral ligands containing an asymmetric carbon atom were reported.^930−938^ For instance, employing a tridentate pybox-type (pyridine-2,6-bis(oxazoline)) ligand, M. A. Halcrow and co-workers reported a series of [Fe^II^(pybox)_2_](ClO_4_)_2_ compounds with homo- and heterochiral crystalline phases,^930^ as well as their heteroleptic analogs showing chirality-modified SCO effects.^931^ A similar study was provided by T. Liu, Y.-Y. Zhu, and co-workers who combined various counterions with [Fe^II^(pybox)_2_]^2+^ complexes.^932^ Chiral Schiff bases are also efficient in the formation of chiral SCO materials.^933−937^ For instance, with bidentate (*S*)-1-phenyl-*N*-(1-butyl-imidazole-2-yl-methylene)ethylamine (L_pbimea_), two chiral polymorphs of [Fe^II^(L_pbimea_)_3_]^2+^ can be afforded.^937^ The phase crystallizing in the *P*2_1_3 space group exhibits a gradual SCO near room temperature while the other one packed in the *R*3 space group is trapped in a high spin state but exhibits a ferroelectric property.

**Figure 54:**
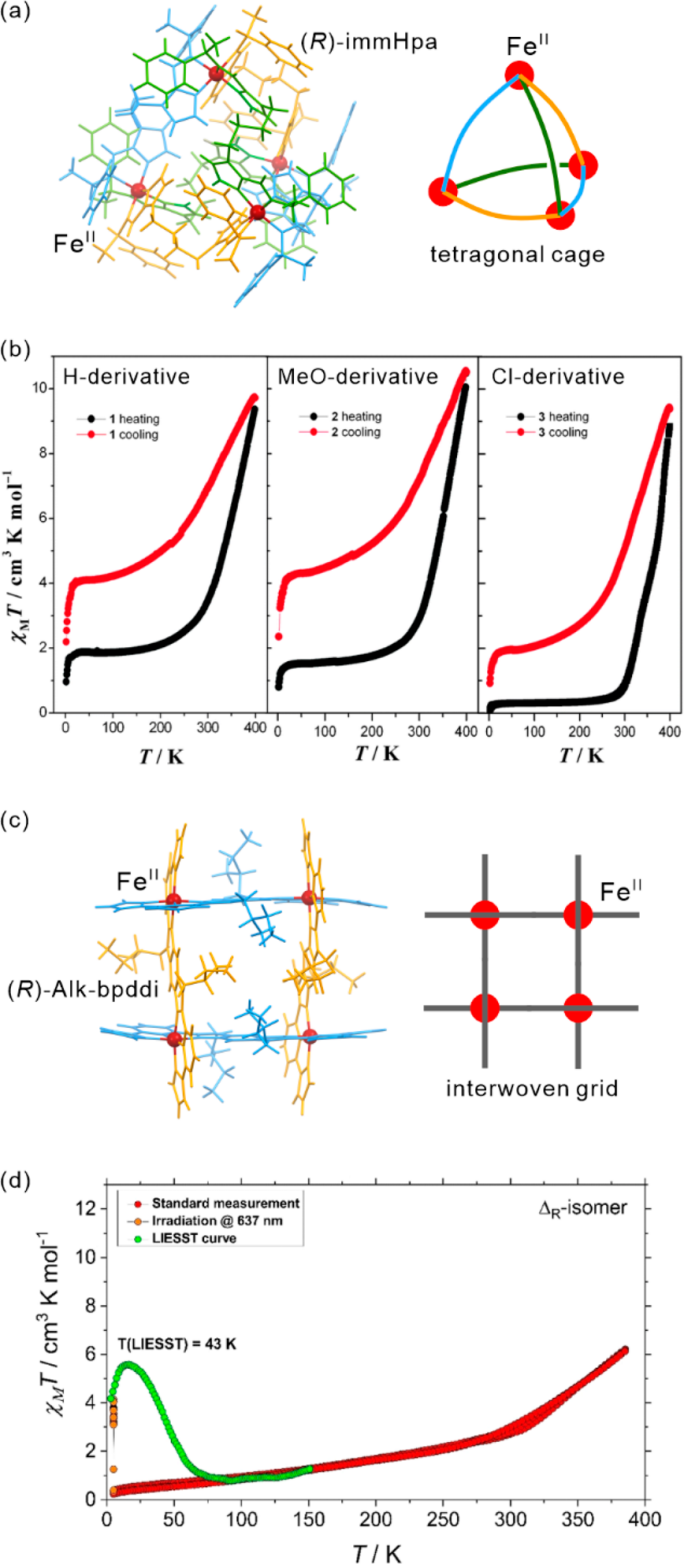
The structure of chiral [Fe^II^_4_((*R*)-immHpa)_6_]^8+^ ((*R*)-immHpa = (*R*)-enantiomer of the H-derivative of 1,4-di((imidazol-2-ylmethylene)-1-phenylethanamine)butane) molecular cationic cage, shown together with the ball-stick model of the related tetrahedral cage (a),^939^ the courses of the SCO effect for three different derivatives of this molecular cage, illustrated by the temperature dependences of the *χ*_M_*T* product (b), the structure of chiral [Fe^II^_4_((*R*)-Alk-bpddi)_4_]^8+^ ((*R*)-Alk-bpddi = 2,6-bis(6-(pyrazol-1-yl)pyridin-2-yl)-1,5-dihydrobenzo[1,2-d:4,5-d′]di-imidazole ligand decorated with the chiral alkyl substituent of the (*R*)-form) molecular cation, shown together with the ball-stick model of the [2x2] square grid topology (c),^945^ and its temperature dependence of the *χ*_M_*T* product representing the thermal and photoinduced (LIESST) SCO effect (d). Part (b) was reproduced from ref (939) with permission from the Royal Society of Chemistry. Part (d) was adapted with permission from ref (945). Copyright 2021 John Wiley & Sons.

Besides numerous mononuclear SCO complexes, chiral ligands can also help in forming enantiopure polynuclear spin transition clusters, e.g., if they have more than one chelating pocket and serve as molecular bridges. When the chelating pocket of a bridging ligand is bidentate, e.g., 1,4-di((imidazol-2-ylmethylene)-1-phenylethanamine)butane (immpa) derivatives, it can react with Fe^2+^ ions with the stoichiometry of immpa:Fe = 6:4, producing a tetrahedral SCO cage (Figure 54ab).^939^ The homochirality of this tetrahedral cage (*ΔΔΔΔ*- or *ΛΛΛΛ*- conformation) is selectively driven by the absolute (*SS*)- or (*RR*)- configuration of the bridging ligand. Alternatively, when bridging ligands have two tridentate chelating pockets within the *trans*-configuration, they can react, for instance, with Fe(II) centers with the stoichiometry of 1:1, forming tetranuclear SCO clusters with [2×2] grid-shape topology,^940-941,942,943^ e.g., an [Fe^II^_4_(bpddi)_4_]^8+^ grid with bpddi standing for a 2,6-bis(6-(pyrazol-1-yl)pyridin-2-yl)-1,5-dihydrobenzo[1,2-d:4,5-d′]di-imidazole ligand and its derivatives.^944^ Without chirality embedded in the ligand, such grid SCO molecule can possess overall chirality due to the helical structure of four bridging ligands; however, it was not optically resoluted in the bulk sample. By introducing asymmetric carbon atoms to this parent bridging ligand, M. Ruben and co-workers reported that the *Δ*- or *Λ*-isomer of SCO-active molecular square grids can be selectively enriched by the related chirality-introducing (*RR*)- or (*SS*)-ligands (Figure 54c,d).^945^

The counterions accompanying SCO-active complexes can also serve as chirality-inducing agents. A successful example of this strategy was demonstrated by G. Morgan and co-workers who adopted a BINOL-derived spiroborate, (*S*,*S*)- or (*R*,*R*)-bis[1,1′-binaphthyl-2,2′-diolato]boron, serving as a chiral counterion to resolve the Mn(III) SCO complexes toward homochiral *Δ* or *Λ* configurations, respectively.^946^

Among chiral and polar spin transition materials, some of them also reveal photomagnetic behavior, either originating from the LIESST effect in the case of Fe(II) SCO complexes, photo-induced electron transfer, or even related to the photoinduced dissociation of cyanido ligand as observed in [M^IV^(CN)_8_]^4–^-based (M = Mo, W) coordination systems. The latter scenario was reported both for chiral 1,2-diaminocyclohexane-based Cu^2+^–[M^IV^(CN)_8_]^4–^ networks,^947^ as well as for a porous {[Mn^II^(bmeep)]_2_[W^IV^(CN)_8_]}·10H_2_O (bmeep = 2,6-bis[1-(2-(*N*-methylamino)ethylimino)ethyl]pyridine) photomagnet.^948^ On the other hand, the formation of chiral Fe^2+^–[Nb^IV^(CN)_8_]^4–^ LIESST-active coordination framework allowed to photoswitch second harmonic light as well as critical temperature of long-range magnetic ordering.^143^ Moreover, monometallic chiral SCO complexes were employed to study their excited electronic states by the ultrafast CD and absorption spectroscopies.^949^ All these examples will be presented more in section 7 related to optical multifunctionality in molecule-based magnetic materials.

### 5.3 Design of Chiral and Non-Centrosymmetric Molecular Nanomagnets

**Figure 55:**
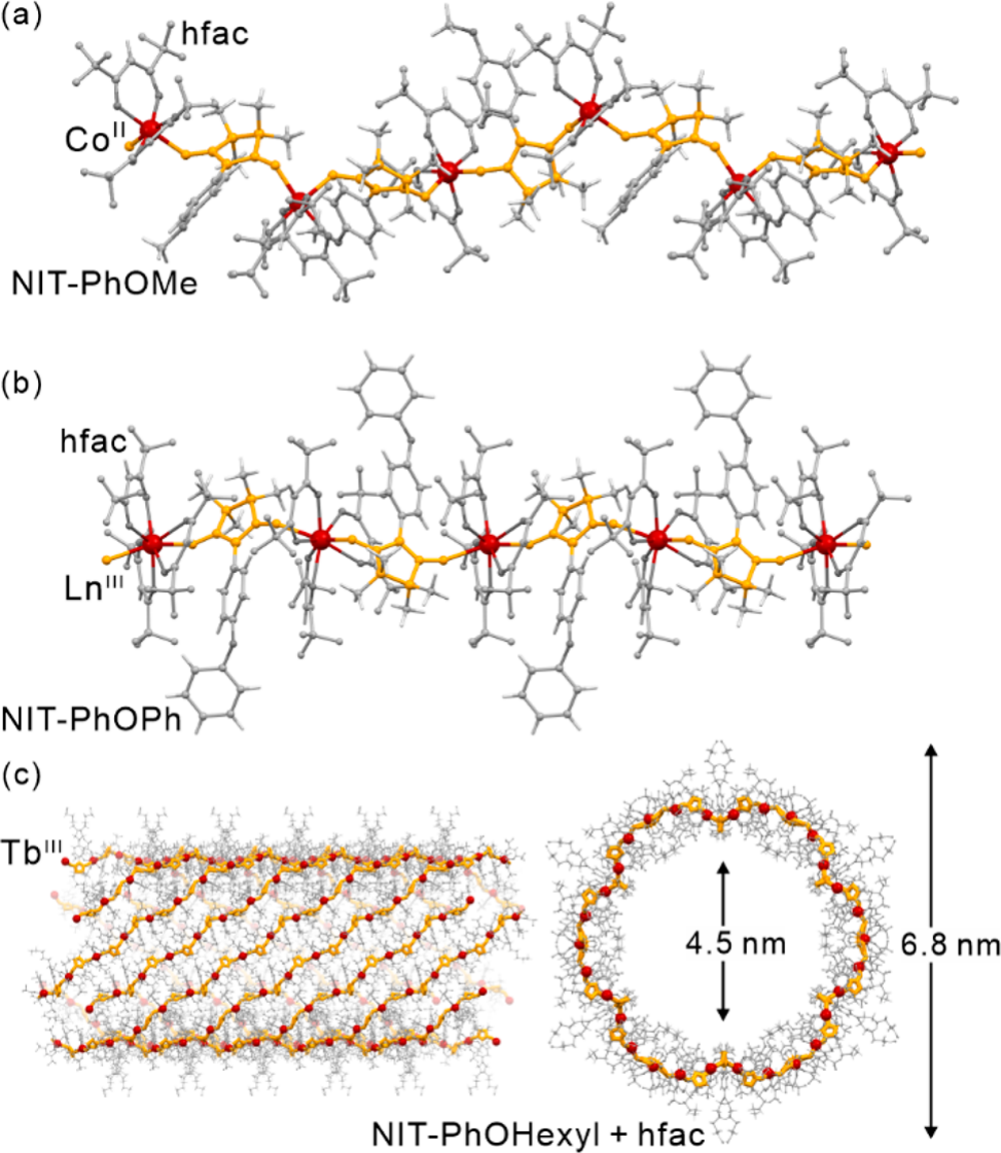
Examples of chiral single-chain magnets formed using the indicated metal centers and the nitronyl nitroxide (NIT)-type radicals: [Co^II^(hfac)_2_(NIT-PhOMe)]}(hfac = hexafluoroacetylacetonate, NIT-PhOMe = 2,4′-methoxo-4,4,5,5-tetramethylimidazoline-1-xoyl-3-oxide) helical chains (a),^66^ [Ln^III^(hfac)_3_(NIT-PhOPh)] (Ln = Dy, Tb, Ho, Er; NIT-PhOPh = 2,4′-benzoxo-4,4,5,5-tetramethylimidazoline-1-oxyl-3-oxide) helical chains (b),^950^ [Tb^III^(hfac)_3_(NIT-PhOHexyl)] (NIT-PhOHexyl = 2-(4′-hexoxyphenyl)-4,4,5,5-tetramethylimidazoline-1-oxyl-3-oxide) nanometer-sized tubular chains in two representative views (c).^955^

The context of chirality introduced to molecular nanomagnets appeared simultaneously when the pioneering works on single-chain magnets (SCMs) were presented. Antiferromagnetic interactions between 2-(4-methoxyphenyl)-4,4,5,5-tetramethylimidazoline-1-oxyl-3-oxide (NIT-PhOMe) organic radicals and [Co^II^(hfac)_2_] (hfac = 1,1,1,5,5,5-hexafluoroacetylacetonate) complexes resulted in the formation of ferrimagnetic spin chains revealing the first observation of the SCM behavior (Figure 55a).^66^ Despite the absence of intrinsically chiral molecular components, this first SCM spontaneously crystallizes as the mixture of two phases appearing in the enantiomorphic pair of *P*3_1_ and *P*3_2_ space groups due to the differently handed helical structures. As a result, the selected crystals consisting of enantiopure helical SCMs were successfully investigated in the context of a magneto-chiral dichroism (MChD) effect (see section 5.4.) as well as second harmonic generation (section 5.5).^950,951^

Such nitronyl nitroxide (NIT) radical derivatives were also found efficient in forming non-centrosymmetric polar SCMs incorporating lanthanide centers (Ln^III^), e.g., NIT-PhOPh can be assembled with [Ln^III^(hfac)_3_] units to form SHG-active SCMs as the mixture of enantiopure crystals of the *P*2_1_2_1_2_1_ space group (Figure 55b).^950−954^ By substituting the phenoxyl group with long aliphatic chains, the helical NIT–Ln^III^ SCM forming a chiral supramolecular nanotube structure was reported (Figure 55c).^955^ It could be further harnessed with metallogel characteristics helpful for making 2-D molecular nanodevices by surface deposition.^956^ In addition to such spontaneous resolution processes, an NIT radical derivative decorated by enantiopure (1*R*)-(−)-myrtenal-based substituent was utilized to prepare a chiral Ln^III^–radical SCM showing optical activity visualized by circular dichroism (CD) spectra.^957^ Besides the usage of NIT-type radicals, chiral single-chain magnets were realized in 1-D coordination polymers revealing efficient exchange coupling between paramagnetic spin centers based on metal ions bridged by short negatively charged ligands, including oxalato,^958^ oxamato,^959,960^ cyanido,^961−963^ and acetato.^964,965^ In this regard, the optical resolution of the related SCMs was mostly induced by enantiomeric organic ligands, such as homochiral BINOL ([1,1′-binaphthalene]-2,2′-diol),^959,960^ salen (*N*,*N*′-bis(salicylidene)ethylenediamine),^961,965^ and pybox (pyridine-2,6-bis(oxazoline)) derivatives.^962^

**Figure 56:**
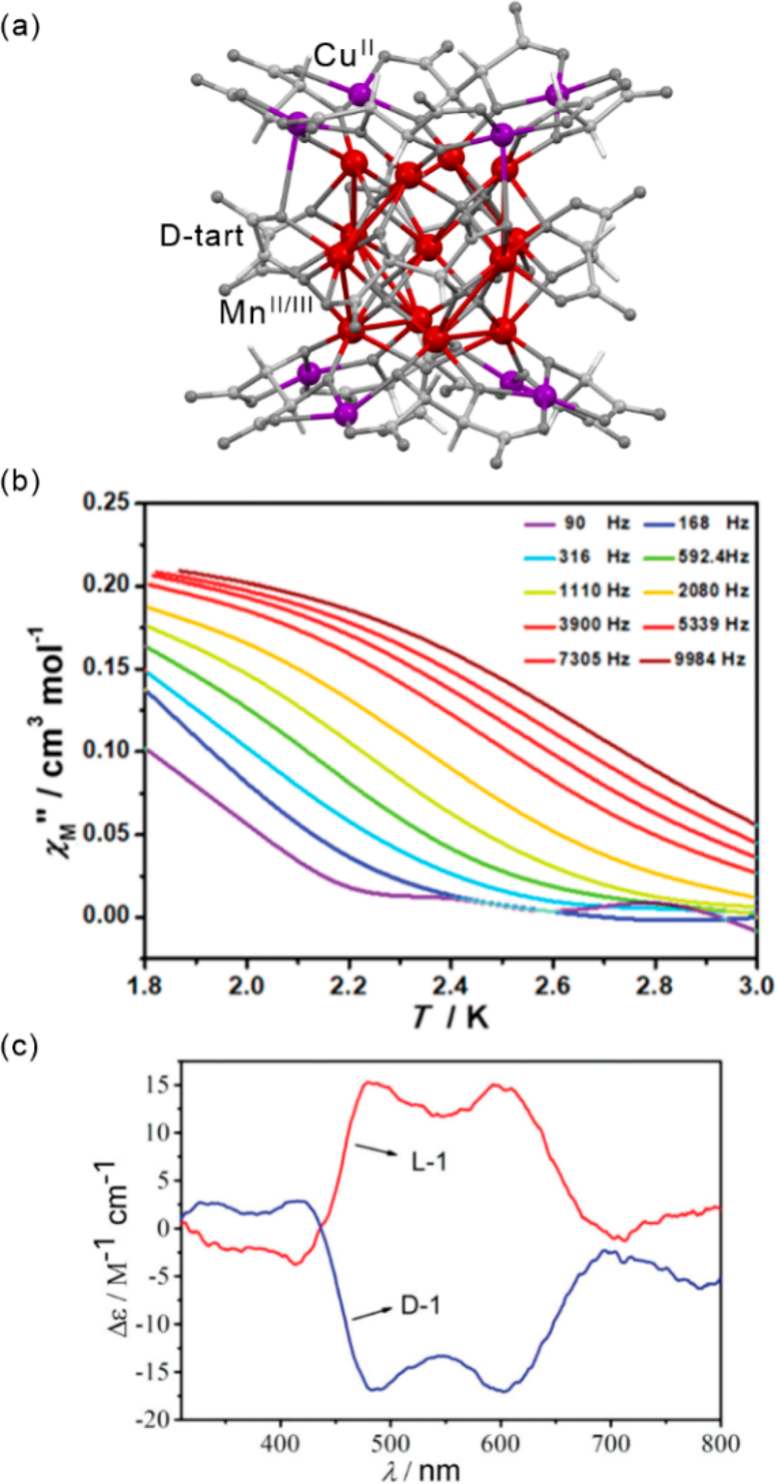
The structure of chiral [Mn^II^Mn^III^_12_Cu^II^_8_(D-tart)_12_(μ_3_-O)(O_2_CH)_0.5_(O_2_CMe)_0.6_(H_2_O)_0.9_]^10–^ (D-tart = D-tartrate) anionic coordination cluster (a),^972^ its temperature dependences of the out-of-phase magnetic susceptibility at various indicated frequencies for the zero *dc* field, representing the SMM behavior for this compound (b), and the solid-state CD spectra for two enantiomers (with D-tart and L-tart) (c). Parts (b) and (c) were reproduced from ref (972) with permission from the Royal Society of Chemistry.

Implementation of chirality into single-molecule magnets (SMMs) was also realized in multimetallic coordination clusters molecules^966−972^ as well as mononuclear complexes.^771,777,973−982^ In most cases, a straightforward strategy relying on the usage of chiral ligands was explored. For instance, in the cluster-type SMMs, by replacing acetate ligand with (*R*)/(*S*)-chloropropionate, classical {Mn^III/IV^_12_} nanomagnets turn into a pair of chiral ones showing the opposite Cotton effects in the CD spectra.^966^ Similarly, chiral (L)/(D)-tartrate ligands assisted in the formation of an enantiomeric pair of larger-sized {Mn^II^Mn^III^_12_Cu^II^_12_} clusters exhibiting slow relaxation of the magnetization co-existing with natural optical activity illustrated by the CD spectra (Figure 56).^972^

**Figure 57:**
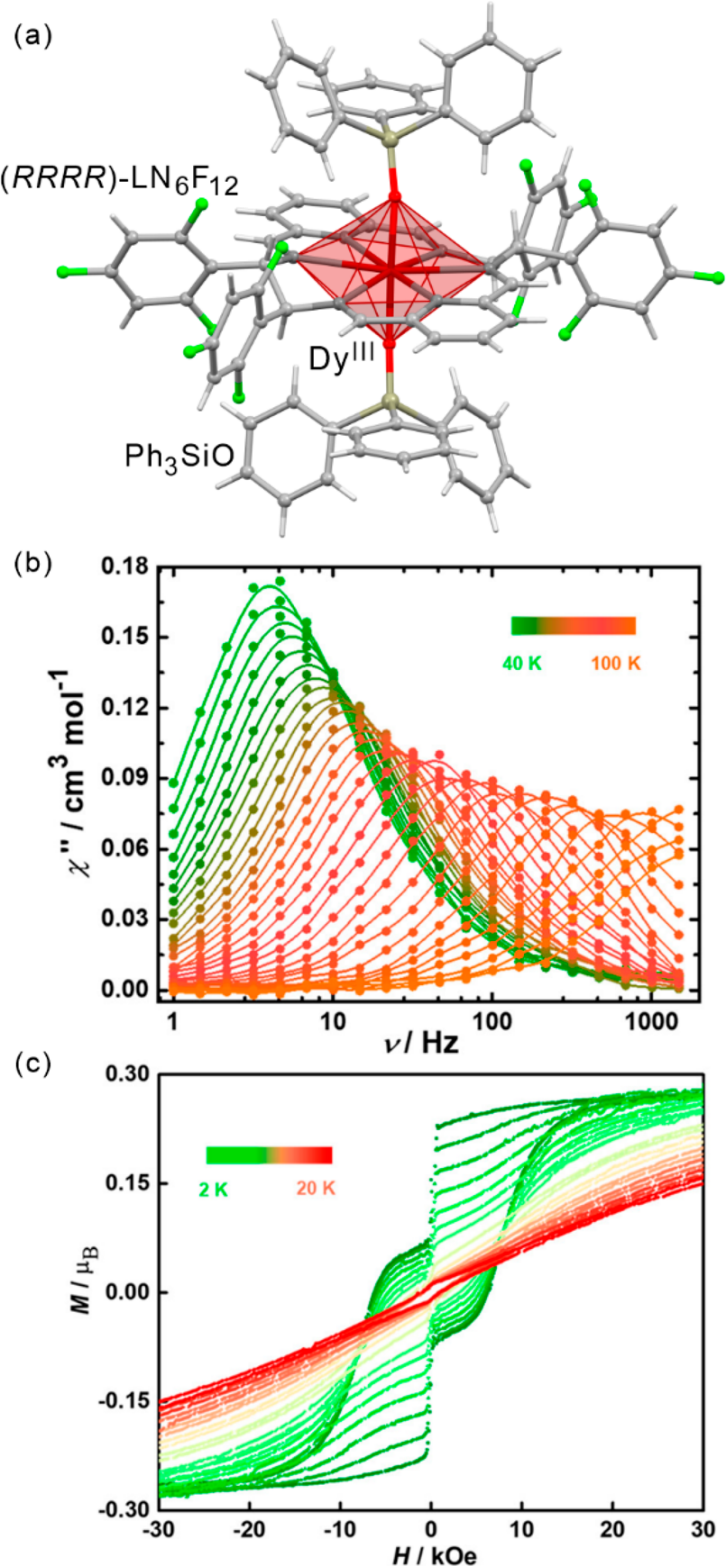
The structure of chiral [Dy^III^((*RRRR*)-LN_6_F_12_)(Ph_3_SiO)_2_](BPh_4_) ((*RRRR*)-LN_6_F_12_ = an enantiopure Schiff base ligand obtained from the condensation of two (1*R*, 2*R*)-1,2-bis(2,4,6-trifluorophenyl)ethane-1,2-diamine and two 2,6-diformylpyridine molecules; Ph_3_SiO = triphenylsilanolate) complex (a),^982^ its representative *ac* magnetic characteristics for the indicated temperature range, illustrating the SMM behavior (b), and the related temperature-variable magnetic hysteresis loops appearing below the blocking temperature (c). Parts (b) and (c) were adapted with permission from ref (982). Copyright 2021 American Chemical Society.

In the case of mononuclear molecular nanomagnets, the prototype SMM of [Tb^III^(Pc)_2_]^−^ (Pc^–^ = bis(phthalocyaninate) anion) can also become chiral when the enantiopure substituent is introduced to the Pc ligand.^973,974^ Many other chiral lanthanide(III)-centered SMMs were prepared taking advantage of chiral ligands, such as derivatives of BINOL ([1,1′-binaphthalene]-2,2′-diol), diverse ligands of the Schiff base-type, and others.^771,777,975−981^ Among them, Z. Zhu et al. reported a chiral high-performance Dy^III^ SMM which utilizes an enantiopure Schiff base employing (1*R*, 2*R*)- or (1*S*, 2*S*)-1,2-bis(2,4,6-trifluorophenyl)ethane-1,2-diamine as an equatorial ligand together with triphenylsilanolate anions as axial ligands (Figure 57).^982^ The resulting enantiomorphic SMMs exhibit a remarkable anisotropic thermal energy barrier exceeding 1800 K with a high blocking temperature of 20 K. For this compound, the chiral substituent sterically offers a rigid molecular structure leading to perfect axial linearity of O-donor negatively charged silanolate ligands which accounts for the considerably improved SMM characteristics compared with the achiral prototype. Furthermore, the air-stability and capability of post-synthetic treatment of this SMM also offered a friendly route for surface deposition of these high-performance chiral nanomagnets.^982^

**Figure 58:**
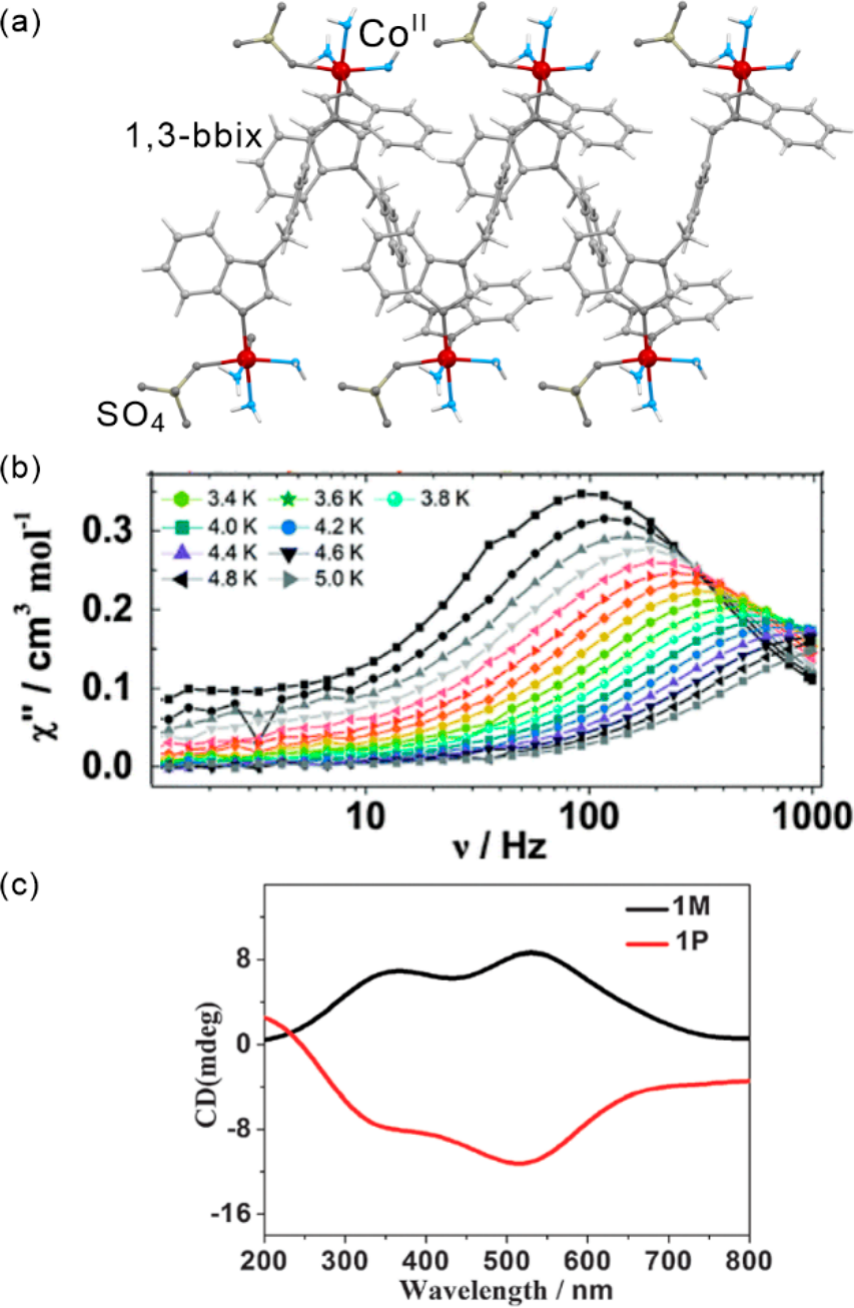
The structure of chiral (*M*)-[Co^II^(SO_4_)(1,3-bbix)(H_2_O)_3_] (1,3-bbix = 1,3-bis((1*H*-benzo[*d*]imidazol-1-yl)methyl)benzene) coordination chain obtained using the chiral molecular agent in the synthesis (a),^986^ the related out-of-phase magnetic susceptibility versus frequency curves at the indicated temperatures, representing the SMM behavior (b), and the CD spectra for two obtained enantiomorphic compounds, (*M*) and (*P*) (c). Parts (b) and (c) were reproduced from ref (986) with permission from the Royal Society of Chemistry.

Although using chiral ligands has been the primary design strategy, a plethora of spontaneously resoluted chiral SMMs was also reported. For the cluster-type ones, chiral {Mn^III^_9_} SMMs can be obtained by assembling Mn^III^ centers with salicylaldoxime, acetate, and methanol achiral ligands,^983^ while among mononuclear SMMs, the reaction of Dy^III^Cl_3_ with pyridine *N*-oxide (pyNO) could yield spontaneously resoluted chiral [Dy^III^(pyNO)_6_(H_2_O)_2_]Cl_3_ molecular nanomagnets.^984^ Unique examples of spontaneous resolution and/or symmetry breaking are attributed to the mononuclear SMMs embedded in spontaneously formed chiral coordination polymers. For instance, in the lanthanide-octacyanidometallate coordination systems, the coordination of 4f metal centers to tetramethylurea ligands led to the crystallization in a non-centrosymmetric *I*2 space group while preserving noticeable 4f-metal-centered magnetic anisotropy.^985^ The direction of spontaneous resolution can even be controlled by the addition of an inducing agent in the synthesis. In this regard, a metal–organic coordination chain built of Co^II^ centers and achiral 1,3-bis((1*H*-benzo[*d*]imidazol-1-yl)methyl)benzene (1,3-bbix) ligands can be selectively resoluted into *M* or *P* enantiomers by adding (*S*)- or (*R*)-3-phenyl-2-((phosphonomethyl)amino)propanoic acid to the synthesis (Figure 58).^986^ The resulting chiral (*M*)- and (*P*)-[Co^II^(SO_4_)(1,3-bbix)(H_2_O)_3_] chains contain magnetically isolated Co(II) complexes that exhibit SMM behavior visualized by slow magnetic relaxation features below 5 K; simultaneously, the enantiopure character of two obtained crystalline phases was confirmed by the CD spectra.^986^

### 5.4 Circular and Magneto-Chiral Dichroism in Molecule-Based Magnetic Materials

Molecule-based magnetic materials crystallizing in non-centrosymmetric space groups that lack mirror symmetry can exhibit natural optical activity (NOA) of differential absorption between left and right circularly polarized light, also known as circular dichroism (CD). The NOA of chiral materials can be characterized by natural CD (NCD) spectroscopy, and the optical resolution between two enantiomers can be evidenced by opposite Cotton effects in the respective CD spectra.^888,987,988^ Electronic CD (ECD) spectroscopy working in the UV–vis region is commonly utilized for characterizing chiral materials, including chiral molecule-based magnetic materials, showing pronounced electronic absorption transitions. It is a particularly useful optical method to probe if the chirality is transferred from peripheral chiral groups to the main chromophores.^918,975,989^ On the other hand, vibrational CD (VCD) spectroscopy working in the NIR regime is a complemental tool for investigating chirality, e.g., residing in organic ligands.^899,990,991^

Circular dichroism can be alternatively induced by magnetization, and the related effect is called magnetic circular dichroism (MCD).^992,993^ Owing to its dependence on the magnetization of the medium, MCD provides a useful spectroscopic tool to study the magnetic anisotropy and other magnetic features of molecule-based materials, including molecular nanomagnets.^72,235,918,994−998^ Particularly, with the development of synchrotron technology, X-ray magnetic circular dichroism (XMCD) spectroscopy can be realized by adopting polarized irradiation in X-ray absorption spectroscopy (XAS). The advantage of element-selectivity and high sensitivity in XMCD was used, e.g., to support the characterization of various molecule-based magnetic materials and electron-transfer systems,^2,572,999−1001^ as well as single-ion magnetic anisotropy and relaxation dynamics of finite layers of organized molecular magnets deposited on surfaces.^656,1002−1005^ Notably, such magnetic optical activity (MOA) of materials originates from the breaking of time-reversal symmetry by the external magnetic field, thus, the MCD effect occurs regardless of chirality. Nevertheless, when chirality appears, the conjunction of NOA and MOA provides a prerequisite to their advanced magneto-optical interplay. The related effect is named magneto-chiral dichroism (MChD) and depicts the differential emission or absorption of unpolarized light between two enantiomers under the external magnetic field.^121,889,890,1006^ The MChD effect is dependent on the product of light wavevector (***k*)** and magnetic field (***B***); thus, for a magnetized chiral medium, the relative magnetization directions (parallel or antiparallel to the ***k***) can be differentiated by its opposite MChD effects on a single unpolarized light source. Therefore, MChD opens an undamaging approach for optical read-out of the magnetization state using unpolarized light. Driven by such intriguing magneto-optical coupling, considerable scientific attention has been recently devoted to the realization of pronounced MChD at easily accessible temperatures for both chiral molecular ferro-/ferrimagnets and chiral molecular nanomagnets.^141,142,922,950,974,1007−1009^

**Figure 59:**
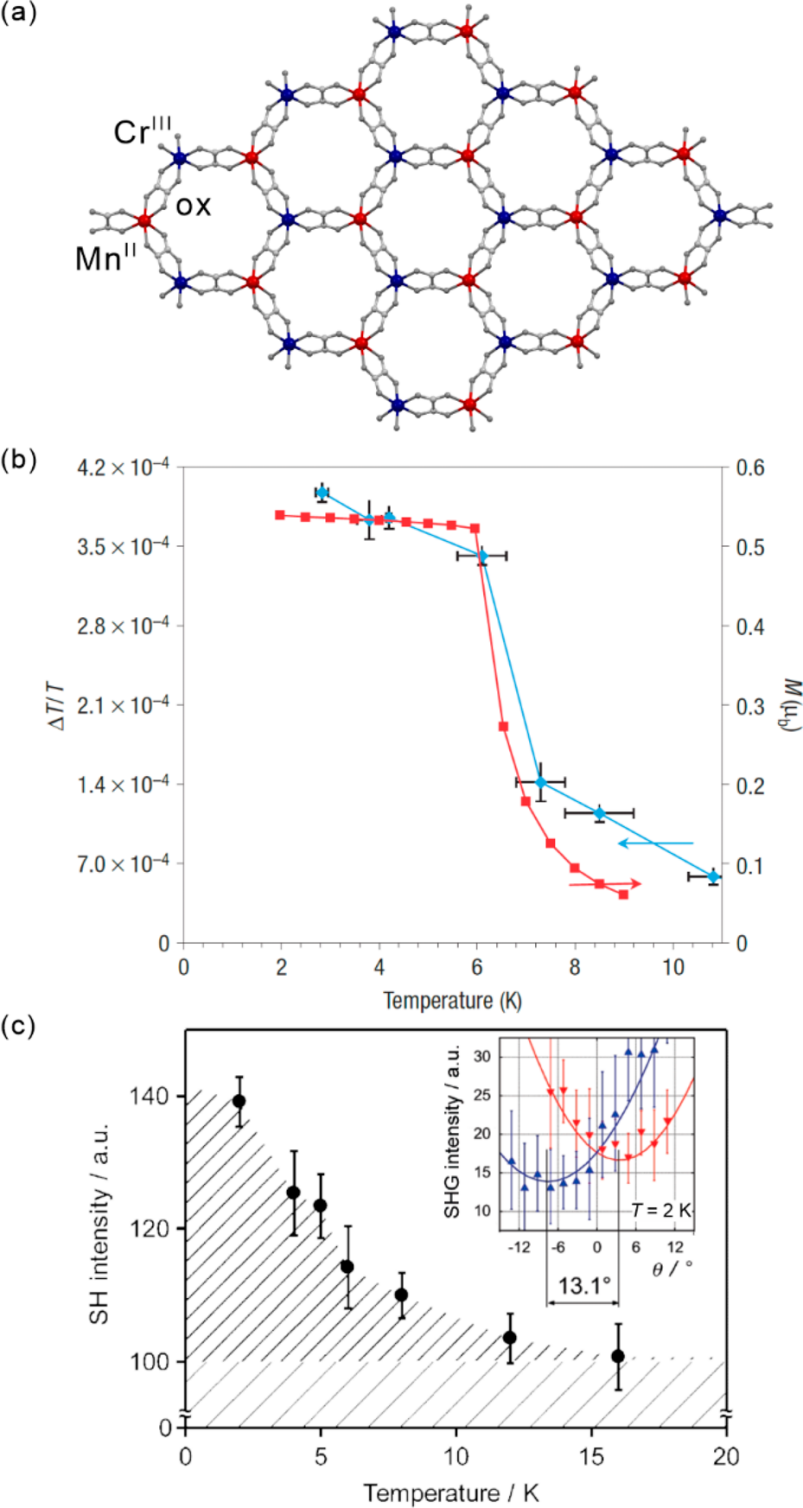
The structure of chiral (N(CH_3_)(*n*-C_3_H_7_)((*S*)-s-C_4_H_9_)){Mn^II^[Cr^III^(ox)_3_]} (ox = oxalato) layered coordination framework (a) (chiral organic cations are omitted for clarity),^922^ the comparison of the temperature dependences of the magneto-chiral dichroism (MChD) signal measured at 615 nm (Δ*T*/*T*) and molar magnetization, showing the correlation between MChD and ferromagnetism (b), the thermal variation of the second harmonic (SH, detected at 532 nm for the incident radiation of 1064 nm) signal under the 300 Oe magnetic field, demonstrating the appearance of the magnetization-induced SHG (MSHG) effect, shown together with the polarization analysis of the SHG signals for the ferromagnetic state (*T* = 2 K) changing upon the shift of the external magnetic field from +1700 Oe (blue points) to −1700 Oe (red points), which indicates the angular switching of the SHG polarization plane upon the magnetization reversal (c). For details regarding the SHG part see the text of section 5.5. Part (b) was reproduced with permission from ref (922) under terms of the CC-BY license. Copyright 2008 Springer Nature. Part (c) was adapted with permission from ref (133). Copyright 2009 American Chemical Society.

For magnetically ordered molecule-based materials, the first observation of MChD was realized in 2008 using the enantiomeric pair of oxalato (ox)-bridged layered coordination frameworks incorporating chiral counter-ions, (N(CH_3_)(*n*-C_3_H_7_)((*S*)-s-C_4_H_9_)){Mn^II^[Cr^III^(ox)_3_]}, which showed a significant increase of the Cr^III^-centered MChD signal below *T*_c_ = 7 K of a ferromagnetic ordering (Figure 59).^133,922^ Recently, a similar phenomenon was observed at a much higher ordering temperature of 38 K by M. Atzori et al.,^141^ who revisited a previously investigated chiral ferrimagnetic framework, {[Mn^II^(*S*/*R*-pnH)(H_2_O)][Cr^III^(CN)_6_]} (pn = 1,2-diaminopropane),^235^ thus, markedly enhancing the working temperature of potential MChD-based magneto-optical devices. Furthermore, this work underlined that chirality lying even in the secondary coordination sphere could boost the MChD effect of achiral magnetic metal centers.^141^

**Figure 60:**
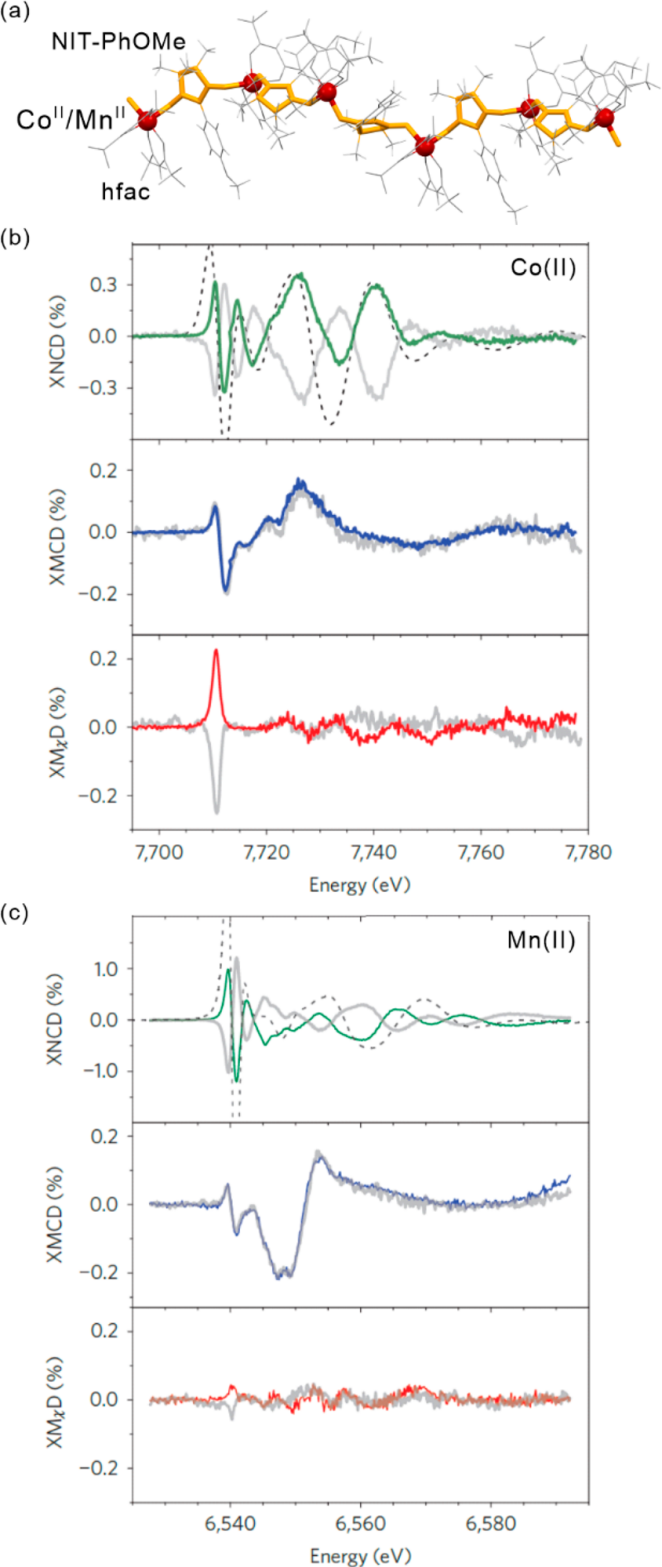
The structure of chiral [M^II^(hfac)_2_(NIT-PhOMe)] (M = Co, Mn; hfac = hexafluoroacetylacetonate, NIT-PhOMe = 2,4′-methoxo-4,4,5,5-tetramethylimidazoline-1-xoyl-3-oxide) helical chains (a),^950^ the normalized spectra of K-edge X-ray natural circular dichroism (XNCD), X-ray magnetic circular dichroism (XMCD), and X-ray magneto-chiral dichroism (MChD) collected at the temperature of 5 K and the magnetic field of 3 T for the Co(II)-based chains (b), and their Mn(II)-containing analogs (c). In (b) and (c), colored solid curves and grey solid curves represent the spectra of two obtained enantiomers, respectively, and dashed curves represent the calculated XNCD spectra for the *P*3_1_ enantiomer. Parts (b) and (c) were reproduced with permission from ref (950) under terms of the CC-BY license. Copyright 2015 Springer Nature.

Among lower dimensional coordination systems serving as molecular nanomagnets, R. Sessoli et al. investigated the MChD effect in a helical single-chain magnet, [Co^II^(hfac)_2_(NIT-PhOMe)] (hfac = hexafluoroacetylacetonate, NIT-PhOMe = 2,4′-methoxo-4,4,5,5-tetramethylimidazoline-1-xoyl-3-oxide), as well as its Mn^II^-substituted analog, using K-edge X-ray absorption spectroscopy (Figure 60).^950^ Thanks to the large magnetic anisotropy of Co^II^ centers, this chiral SCM revealed a pronounced MChD signal on the Co-related pre-edge, while for the Mn^II^ analog such signal was ca. 10 times weaker due to the absence of orbital contribution to the magnetic moment of the Mn^II^ centers. In addition, this work questioned the classical MChD model by showing that the strength of MChD in hard X-ray energy regime does not follow the simple product of NOA and MOA, and more advanced models are demanded for comprehensive understanding of MChD effect in the wide electromagnetic wave spectrum.^950^ For single-molecule magnets, observation of MChD has also been realized in the enantiomeric pair of dinuclear Dy^III^-based triple-decker SMMs and the pair of mononuclear Tb^III^-based double-decker SMMs dissolved in chloroform solvent.^974,1008^ The observed MChD signals in the UV–vis range were attributed to the porphyrin and phthalocyanine ligands containing chiral moieties.

**Figure 61:**
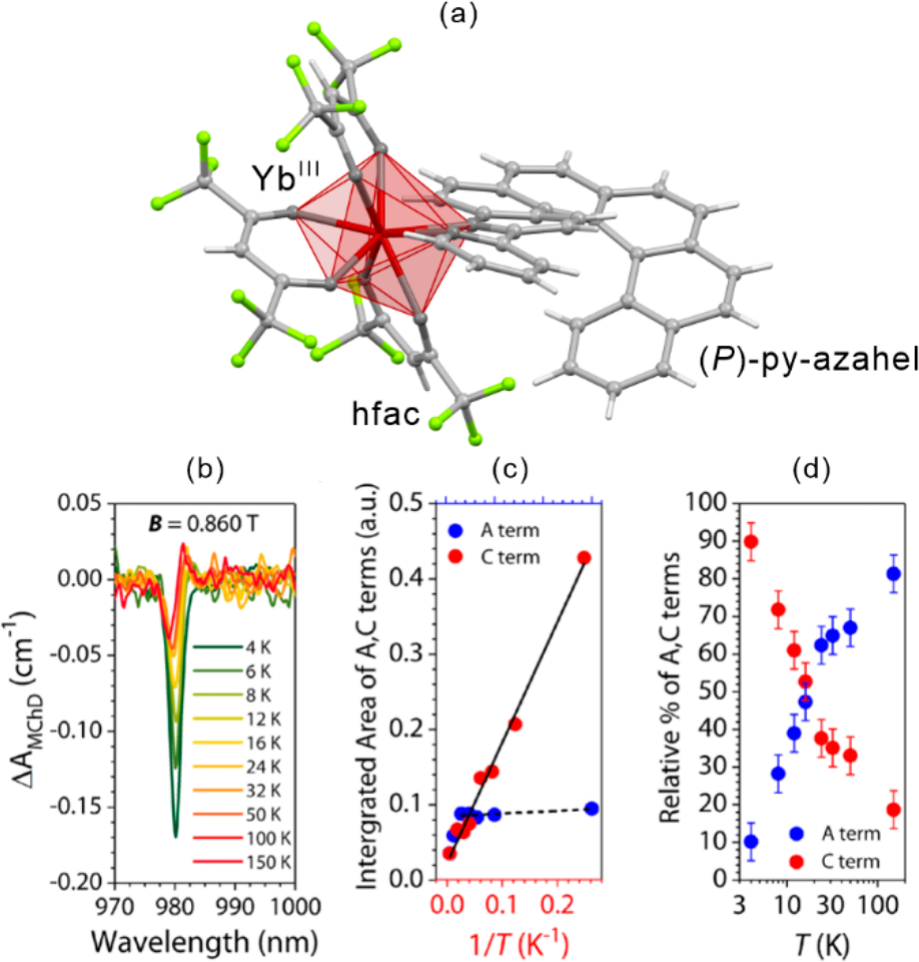
The structure of chiral [Yb^III^(hfac)_3_((*P*)-py-azahel)] (hfac = hexafluoroacetylacetonate, py-azahel = 3-(2-pyridyl)-4-aza[6]-helicene of the (*P*) or (*M*) form) metal complex (a)^1009^ and the MChD spectra of a (*P*)-enantiomer at variable temperatures from the 4–150 K range related to the Yb^III^-centered ^2^F_7/2_→^2^F_5/2_ electronic transition (b); two mechanisms contributing to the MChD, the A term related to the lifting of the degeneracy of energy levels due to the magnetic field and the C term related to changes of population of the ground state levels, are disentangled with their respective integrated areas as a function of temperature (c) and the relative percentage of their contributions to the MChD signals at variable temperatures (d). Parts (b), (c), and (d) were adapted with permission from ref (1009). Copyright 2021 American Chemical Society.

**Figure 62:**
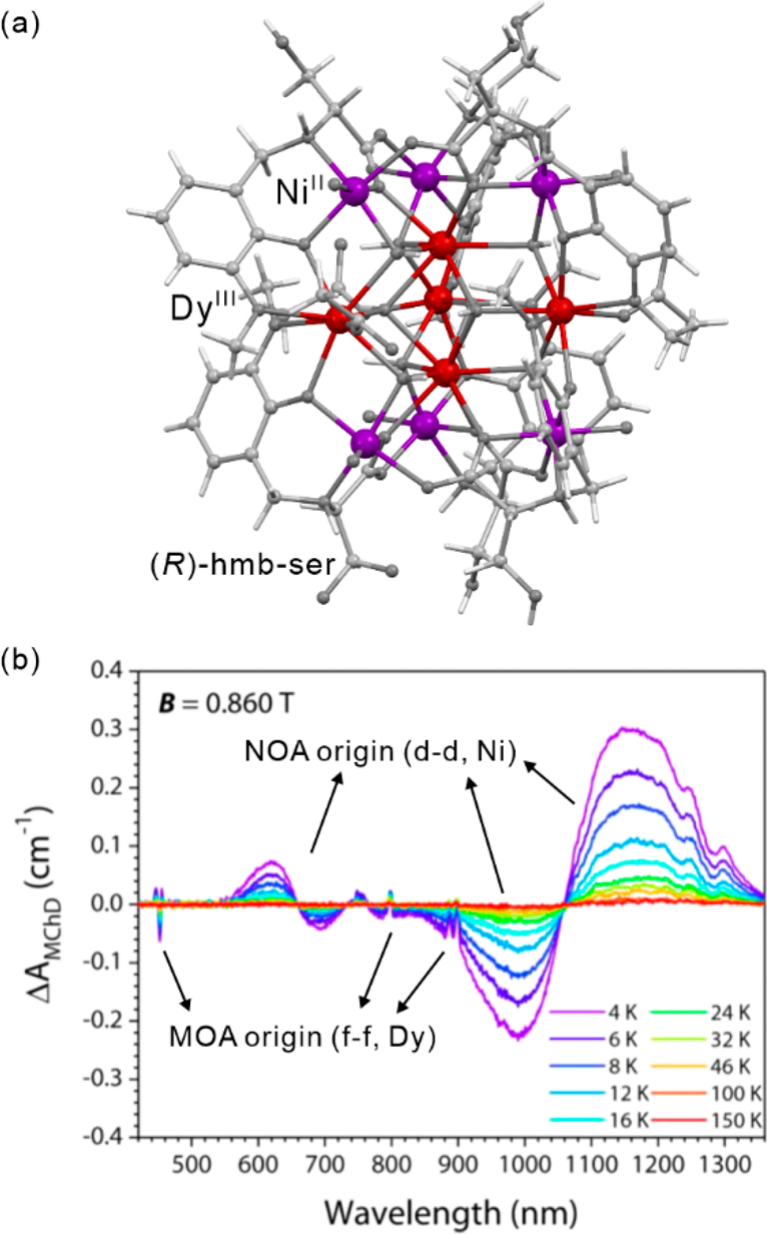
The structure of chiral [Dy^III^_5_Ni^II^_6_((*R*)-hmb-ser)_6_(ac)_3_(μ_3_-OH)_9_(H_2_O)_6_](ClO_4_)_3_·15H_2_O (hmb-ser = (2-hydroxy-3-methoxybenzyl)serine of the (*R*) or (*S*) form; ac = acetate) coordination cluster (a)^142^ and its temperature-variable MChD spectra collected under a magnetic field of 0.86 T within the 4–150 K temperature range (b). The dominant magnetic optical activity (MOA) origin of the MChD signal related to the Dy(III) centers, as well as the analogous dominant natural optical activity (NOA) origin of the MChD signal related to the Ni(II) centers, were indicated in (b). Part (b) was adapted with permission from ref (142). Copyright 2022 American Chemical Society.

In pursuit of MChD signal directly originating from metal-based chromophores, M. Atzori et al. studied paramagnetic helicene-based mononuclear Yb^III^ complexes (Figure 61) while X. Wang et al. reported the analogous physical investigation of polynuclear 3d–4f {Dy^III^_5_Ni^II^_6_} clusters (Figure 62) which are enantiopure in their solid-state crystalline forms.^142,1009^ The former work showcased the first MChD example based on lanthanide-centered absorption properties related to their f–f electronic transitions as well as the first disentanglement of two different contribution mechanisms of magneto-chiral dichroism activity.^1009^ The latter work underlined the metal-selective contribution of NOA and MOA effects toward the overall co-existing 3d- and 4f-centered MChD signals (Figure 62).^142^ Furthermore, these pronounced signals can be observed up to the high-temperature limit of 150 K. Although the studied compounds were not proven to serve as SMMs, these pioneering works offered an insightful understanding of MChD mechanisms and laid the foundation for the design of high-temperature SMM-based devices utilizing the MChD signal. Given that recent chiral molecular nanomagnets could reach an anisotropic energy barrier over 1800 K while achieving good air stability, the exploration of the MChD signal is expected to bring the fruitful conjunction of molecular nanomagnetism with chirality-induced advanced magneto-optical interplay.^982,1006^

In this context, in recent years, increased scientific attention has been devoted to the introduction of both chirality and photoluminescence for Ln(III)-based single-molecule magnets, aiming at the generation of circularly polarized luminescence (CPL). For instance, such attempts were made using Yb^3+^ ions combined with 3-trifluoroacetyl-(+)-camphorato (facam) and bis(1,10-phenantro[5,6b])tetrathiafulvalene ligands or for the series of the Dy(III)-based complexes with BINOL-functionalized (BINOL = ([1,1′-binaphthalene]-2,2′-diol)) bisphosphate ligands.^777,1010^ Such examples, combining both structural chirality/polarity, luminescence, and magnetic properties, were gathered and discussed in section 7.3. Moreover, optical thermometry based on the magnetic circular dichroism effect was recently presented for lanthanide(III) complexes.^1011^ This uncovers outstanding potential in the development of advanced SMM-based magneto-optical systems further exploring MCD, MChD, as well as CPL effects in molecule-based magnetic materials.

### 5.5 Second-Harmonic Generation in Molecule-Based Magnetic Materials

**Figure 63:**
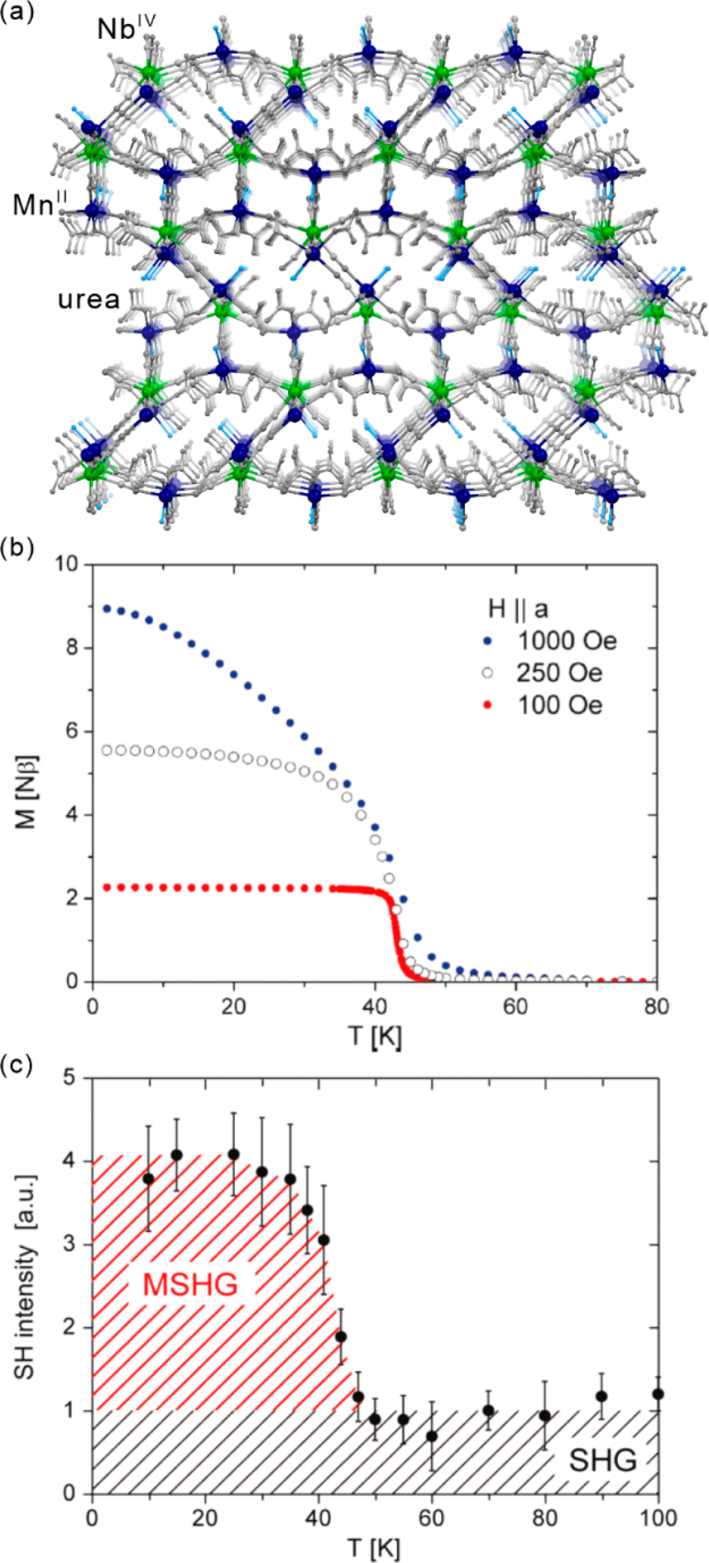
The representative view on chiral α-{[Mn^II^(urea)_2_(H_2_O)]_2_[Nb^IV^(CN)_8_]} coordination framework (a),^1023^ its temperature dependences of molar magnetization for the single crystal at the indicated magnetic field conditions (b), and the related temperature dependence of the SHG signal with the indicated area corresponding to the magnetization-induced SHG (MSHG effect) (c). Parts (b) and (c) were adapted with permission from ref (1023). Copyright 2011 American Chemical Society.

Second harmonic generation (SHG) is a nonlinear optical (NLO) process of light-matter interaction showing frequency doubling of the incident light propagating through the solid, and it occurs in crystals belonging to most non-centrosymmetric space groups, except for those in the 432 point group.^19,202^ The SHG effect is one of the broadest investigated NLO processes due to its vital applications in the laser industry, ultra-short pulse measurements, advanced optical microscopy, and physical characterization of new materials, e.g., ferroelectrics or chiral phases, as it is used as a tool to detect non-centrosymmetricity.^19,202,1012−1016^ The SHG activity was found in diverse molecular magnetic materials that were constructed from proper molecular building blocks ensuring their chiral, polar, or at least non-centrosymmetric crystal structures. These materials could be, then, considered as bifunctional solid systems linking magnetic phenomena with NLO properties.^100,122,1017−1021^ More importantly, in molecule-based magnets demonstrating long-range magnetic ordering, the interplay between SHG and magnetism creates a coupling effect, called magnetization-induced SHG (MSHG), in which the SHG intensity is enhanced significantly due to the large spontaneous magnetization below the *T*_c_ (refs (133, 143, 209, and 1022−1024)). It was primarily demonstrated using a well-known class of molecule-based magnets based on Prussian blue analogs.^209^ C. Train et al. also observed such MSHG phenomenon exploring oxalato-bridged heterometallic coordination frameworks. They reported a layered molecule-based ferromagnet of (N(CH_3_)(*n*-C_3_H_7_)((*S*)-s-C_4_H_9_)){Mn^II^[Cr^III^(ox)_3_]} (ox = oxalato) which was initially investigated for its MChD effect (see Figure 59 and the related section 5.4).^133^ It was shown that its second harmonic (SH) intensity increased by 40% in the cooling course through the *T*_c_ (Figure 59c). In addition, magnetization at 2 K along the crystallographic *b* axis led to a lowered symmetry of the magnetic space group with non-zero nonlinear magnetic susceptibility tensor, and the MSHG polarization of this compound exhibited an angular switching of 13.1° upon magnetization reversal, demonstrating an alternative way for optical read-out of magnetization state.^133^ The MSHG effect was also observed in a chiral cyanido-bridged α-{[Mn^II^(urea)_2_(H_2_O)]_2_[Nb^IV^(CN)_8_]} network exhibiting an impressive 4-times stronger SH intensity below its ferrimagnetic ordering temperature of 43 K when compared with a paramagnetic phase (Figure 63).^1023^

**Figure 64:**
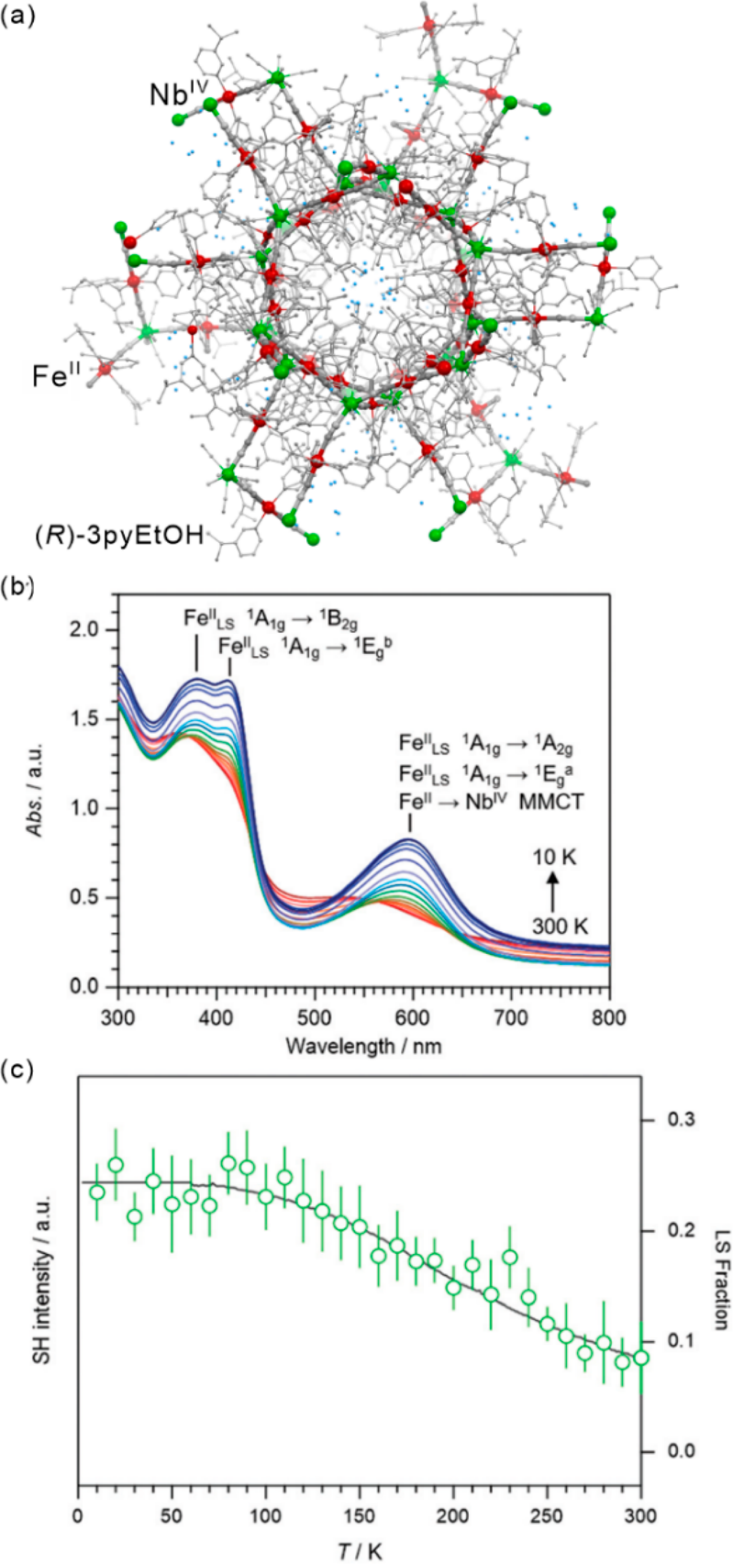
The structure of chiral {[Fe^II^_2_((*R*)-3pyEtOH)_8_][Nb^IV^(CN)_8_]}·6H_2_O (3pyEtOH = 1-(3-pyridyl)ethanol in the (*R*) or (*S*) form) coordination framework (a),^482^ its temperature-variable UV–vis absorption spectra with the indicated electronic transitions appearing for the low-spin (low-temperature) phase (b), and the temperature dependence of the SH intensity upon 2.2 mW incident laser irradiation compared with the Fe^II^ low-spin (LS) fraction (c). Parts (b) and (c) were reproduced from ref (482) with permission from the Royal Society of Chemistry.

Besides molecule-based systems showing long-range magnetic ordering, spin transition materials were also demonstrated to exhibit switching of SHG signal as a function of its magnetism related to the change of a spin state. In the enantiomeric pair of spontaneously resoluted {[Fe^II^_2_(4-bromopyridine)_8_][Nb^IV^(CN)_8_]} networks showing highly cooperative SCO behavior, the temperature-dependent SH intensity is synchronized with the *χ*_M_*T*–*T* plot, additionally reproducing the thermal hysteresis of the Fe^II^-centered thermal spin transition.^143^ In another pair of three-dimensional cyanido-bridged {[Fe^II^_2_(*R*-/*S*-1-(3-pyridyl)ethanol)_8_][Nb^IV^(CN)_8_]} networks, the temperature dependence of SH intensity stays in line with the gradual appearance of Fe^II^ low-spin states related to thermally-induced SCO (Figure 64).^482^ The changes in the SHG effect occur together with the changes in the optical absorption. Such synchronous switching of SHG, optical absorption, and magnetic properties, was further employed in the above-mentioned 4-bromopyridine-containing Fe^II^–Nb^IV^ assembly for a more advanced coupling effect relying on the photoswitching of SH light characteristics, e.g., its polarization plane, which was achieved by linking chirality and photomagnetism. This multifunctionality is discussed in section 7.2.^143^

Within the subfield of molecular nanomagnetism, the expected synergy between SHG and molecular magnetic bistability has not yet been reported and remains a future challenge. Nevertheless, the incorporation of SHG activity within these systems can lead to advanced multifunctional magnetic materials which was achieved in a series of SCMs and SMMs crystallizing in non-centrosymmetric space groups (refs (798, 951, 953, 958, 981, 1018, 1019, and 1021)). In particular, lanthanide(III) complexes provided a good molecular platform to investigate the magnetic and structural factors that contribute to the SHG behavior which was presented by preparing a series of 4f-element-containing analogs.^1025,1026^ In the family of isostructural SCMs, which are based on [Ln^III^(hfac)_3_] complexes (Ln = Eu, Tb–Yb) and NIT-PhOPh (2,4′-benzoxo-4,4,5,5-tetramethylimidazoline-1-oxyl-3-oxide) organic radicals (Figure 55), the non-centrosymmetric *P*2_1_2_1_2_1_ was detected. This gives the SHG signal which was found to be mainly tuned by intermolecular π-stacking interactions while magnetic contributions from lanthanide ions are negligible.^953^

Another route toward the generation of the SHG activity for a lanthanide(III)-containing system was lately reported. It employs the nitroprusside complex, [Fe^II^(CN)_5_(NO)]^2–^, spontaneously breaking the symmetry of the crystal lattice and resulting in a non-centrosymmetric *Pna*2_1_ space group.^531^ However, as the presence of the pentacyanidonitrosylferrate(II) moiety generated the photoswitchability of the SH signal, its case will be described in section 7.2 devoted to photoswitchable polar molecule-based magnetic materials.

The presence of the polar crystal structure not only induces the SHG activity but can also result in the generation of ferroelectricity. For magnetic materials such an effect is highly desired, e.g., for the case of magnetically ordered phases, where a conjunction of magnetic and electrical ordering effects may result in multiferroicity and related magneto-electric effects.^1027^ On the other hand, it was lately presented even for a mononuclear Yb(III)-based field-induced SMM showing ferroelectricity, that at room temperature a multi-state switchable system can be obtained by manipulating both electric and magnetic fields.^1028^ The examples from both groups, showing also d- or f-block metal-centered emission, respectively, will be discussed broadly in section 7.4, as examples of the most advanced multifunctionality achievable for molecule-based magnetic materials.

## 6 Switching of Optical Phenomena in Molecule-Based Magnetic Materials by Non-Light External Stimuli

In the previous sections of this Review, several molecular materials exhibiting the sensitivity of magnetic and/or optical features upon external stimuli in the form of temperature change (sections 2 and 4.4) or light irradiation (section 3) were discussed. In a more general context, the nature of molecule-based materials, relying on their great relative sensitivity toward various structural and environmental factors, makes them promising in terms of efficient switching or modulation of physical properties, in particular optical effects, by other non-light external stimuli including chemical factors such as humidity or gas vapors,^1029−1031^ electric field,^1032,1033^ and pressure or applied mechanical force.^1034−1036^ As such, a vast number of coordination systems, including MOF materials,^196,1037,1038^ as well as discrete molecules,^1039−1041^ were shown to reveal externally switchable electrical or optical properties, proving this concept. This leads to the broad branch of research on stimuli-responsive materials with multiple applications, including advanced sensors, actuators, optoelectronic devices, information storage and processing systems, and medical tools.^391,1042−1044^ The mechanism of the sensitivity toward external stimuli may take advantage of various physical effects or chemical processes; thus, should be well recognized for every material’s property separately. For instance, the inclusion of guest molecules may affect the electronic structure by non-covalent supramolecular interactions, modify the phonon mode scheme, or even induce redox activity of the initial system, and in all these cases the optical properties such as absorption and emission properties can be changed.^1045−1047^ Similar factors may also affect the magnetic properties, thus for stimuli-responsive magnetic materials often one can observe the simultaneous change of magnetic and optical features, the latter especially related to the optical absorption variation.^1048,1049^ In the following sections, we will briefly describe three groups of dynamic molecule-based magnetic materials that can be classified as multifunctional due to their external-stimulus-responsivity influencing both magnetic and optical characteristics. Molecule-based magnetic materials depicted in these sections, however, belong to a few different classes, as they vary from simple paramagnetic systems, through magnetically ordered phases, as well as spin-crossover and electron-transfer materials, up to molecular nanomagnets, with some of them often being additionally sensitive to previously discussed external stimuli of temperature change and light irradiation. In the following sections, we will focus on the switching of optical phenomena in diverse molecular magnetic materials by chemical stimuli (6.1), electric field (6.2), and pressure or mechanical force (6.3).

### 6.1 Chemical Stimuli for Switching of Optical Phenomena in Molecule-Based Magnetic Materials

**Figure 65:**
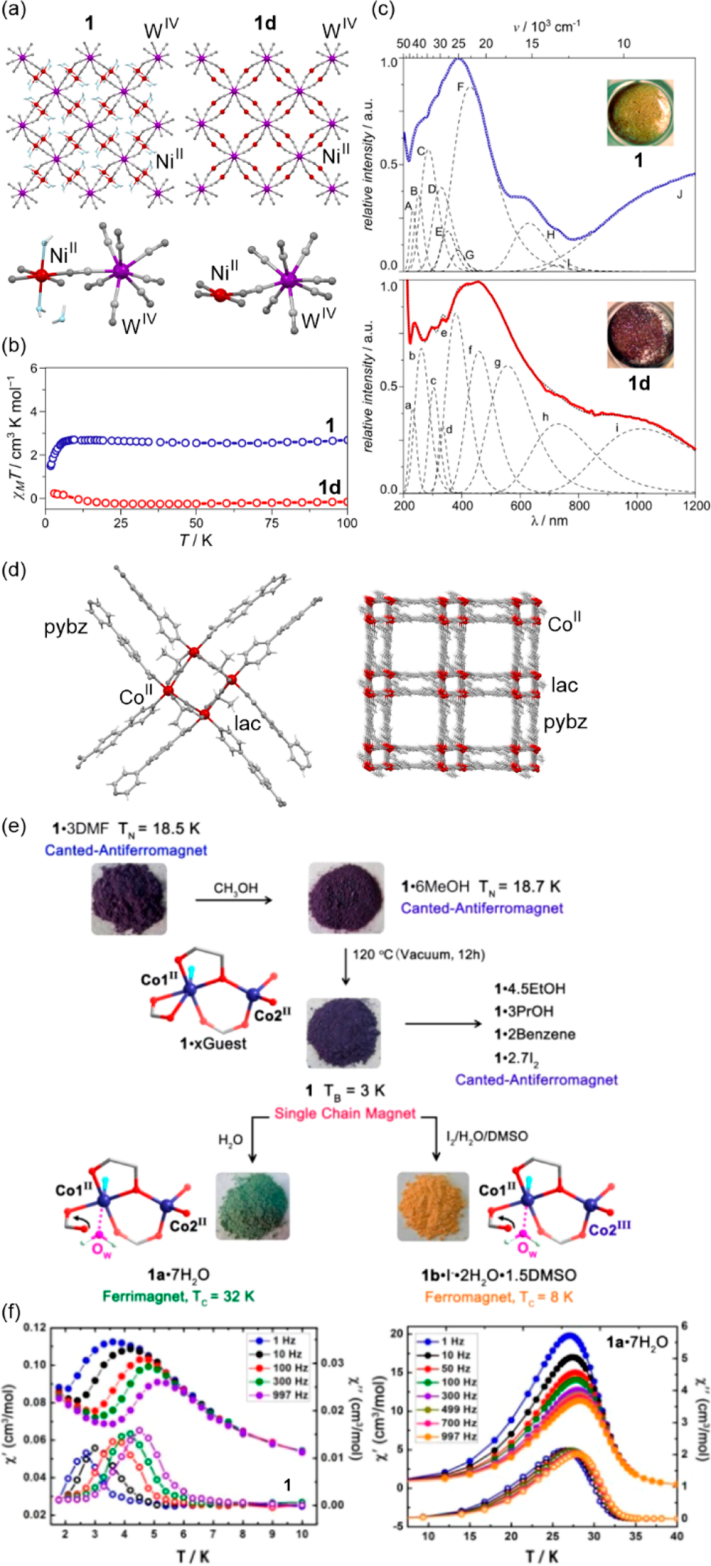
The representative structural views on {[Ni^II^(H_2_O)_2_]_2_[W^IV^(CN)_8_]}·4H_2_O coordination network and its dehydrated {Ni^II^_2_[W^IV^(CN)_8_]} form (a),^1063^ the related temperature dependences of the *χ*_M_*T* product (b), and their UV–vis-NIR absorption spectra shown with the photos of the powder samples (c), the structure of [Co^II^_3_(lac)_2_(pybz)_2_]·3dmf (lac = D- and L-lactate; pybz = 4-pyridyl benzoate) coordination network (d),^1064^ the scheme of its post-synthetic modifications, shown together with the resulting magnetic ground states, color of the samples, and changes in coordination and valence of embedded Co ions (e), and the temperature dependences of the *ac* magnetic susceptibilities for two indicated phases (f). Parts (b) and (c) were adapted with permission from ref (1063). Copyright 2017 American Chemical Society. Parts (e) and (f) were adapted with permission from ref (1064). Copyright 2014 American Chemical Society.

As already mentioned, the influence of chemical factors on the physical properties of molecule-based materials can be large when compared with usually much less sensitive classical inorganic solids. For several decades, such a feature has been explored in metal–organic frameworks (MOFs) in various aspects, involving changes in optical, electrical, catalytic, magnetic properties, and others.^1050−1055^ In general, as coordination compounds are often synthesized in the solution, they tend to incorporate solvent molecules of crystallization, which can be removed to activate the crystalline material toward the sorption of various solvent vapors and gases. This can happen for the related coordination frameworks showing various magnetic properties. In this regard, the critical point, especially for molecule-based magnetic materials exploring often subtle structural changes largely affecting the magnetic effects, is the determination of the crystal structure variation upon the influence of the chemical stimulus. Therefore, in the best-case scenario, the removal of the solvent of crystallization, as well as every structural change involving the further inclusion of guest molecules, should occur through a single-crystal-to-single-crystal (SC-SC) transformation,^1056−1059^ or at least without the loss of crystallinity for the powder sample enabling the studies by powder X-ray diffraction (P-XRD) techniques.^108,1060^ Moreover, the reversibility of every change is another important factor, especially considering such materials as potential sensors. Therefore, from both points of view, materials with a highly-dimensional rigid framework or systems with a significant degree of structural flexibility leading toward the reversible breathing effect are desired.^1061,1062^ For instance, such an extremely rigid coordination skeleton is found for a purely inorganic three-dimensional {[Ni^II^(H_2_O)_2_]_2_[W^IV^(CN)_8_]}·4H_2_O framework where each Ni^II^ center is coordinated by four bridging cyanido ligands and two additional *trans*-positioned aqua ligands, forming a geometry close to an octahedron (Figure 65a).^1063^ For the [W^IV^(CN)_8_]^4–^ moieties all cyanido ligands are involved in the formation of intermetallic linkages. Upon the thermal treatment, both weakly bounded water molecules of crystallization, as well as aqua ligands, are removed in an SC-SC manner, leaving the Ni^II^ centers square planar. Such a change in the coordination number and geometry changes the Ni^II^ spin state from *S* = 1 to *S* = 0. As the octacyanidometallate complex involved in the formation of the network is diamagnetic itself, the course of the molar magnetic susceptibility indicates the transformation from the paramagnetic towards the diamagnetic phase, accordingly (Figure 65b). The change of the spin state for the Ni(II) complex is also accompanied by the change in the UV–vis-NIR absorption properties.^1063^ Weakly colored Ni^II^ octahedral complexes together with yellow [W^IV^(CN)_8_]^4–^ moieties result in yellow powder samples of this system. After the dehydration, the appearance of strong visible light absorption originating from the d–d electronic transitions of the square-planar Ni^II^ complexes leads toward a deep red sample color (Figure 65c).^1063^ Interestingly, the dehydration of such a system is also accompanied by a change in thermal expansion properties from positive thermal expansion (PTE) to negative thermal expansion (NTE) which is related to the dominant role of low-energy transverse vibrations of cyanido bridges in the solvent-free framework.^370,1063^

Another example of an exceptionally dynamic coordination polymer, [Co^II^_3_(lac)_2_(pybz)_2_] (lac = D- and L-lactate; pybz = 4-pyridylbenzoate),^1064^ is composed of metal-lactate chains further linked by pybz ligands into the 3-D MOF (Figure 65d). The rigidity of this coordination skeleton allows for the post-synthetic elimination of dmf molecules of crystallization in a single-crystal-to-single-crystal manner. Then the desolvated phase incorporates various gases and organic solvents without losing crystallinity. All of these phases, as well as the dmf-solvated one, behave as canted antiferromagnets due to interchain magnetic exchange interactions. Moreover, the desolvated system acts as a single-chain magnet (SCM) with an anisotropic thermal energy barrier of 41(8) K, suggesting good magnetic isolation of anisotropic spin chains within the supramolecular framework for such a phase. All of the depicted phases are characterized by a purple sample color originating from a constrained octahedral geometry of Co^II^ sites coordinated by bidentate lactate ligands. Different structural features, accompanied by changes in the magnetic behavior and optical absorption properties are found when the desolvated phase is treated with water. Then the sample partially loses crystallinity, but the Raman spectroscopy suggested the transformation of the Co^II^ complex into the regular octahedron with an additional aqua ligand and lactate molecule coordinated using only one oxygen atom of the carboxylate group. A similar structural change is observed when the sample is treated with the I_2_/dmso/H_2_O mixture used to oxidize the Co^II^ tetrahedral site to Co^III^. In both latter cases, the intense absorption in the visible region is reduced, thus the water-solvated system is pale green, while the partially oxidized network reveals an orange color. In terms of magnetism, the change of the sign of the interchain exchange results in ferrimagnetic behavior for the former system with the *T*_c_ of 32 K, while the latter, due to the presence of Co^III^ sites, acts as a ferromagnet but with a reduced *T*_c_ = 8 K (Figure 65e,f).^1064^

The efficient and reversible switching of both optical and magnetic properties governed by chemical stimuli was well presented within an extensively studied class of spin-crossover materials.^1065^ In such materials, the application of chemical factors can affect not only the temperature dependence of the molar HS-to-LS (high-spin-to-low-spin) ratio but also even change the spin state at room temperature. The former case, for instance, was shown for discrete complexes of [Fe^II^(tpa)(NCS)_2_] (tpa = tris(2-pyridylmethyl)amine) upon exposition to methanol vapors.^1066^ The related solvent-induced SC-SC process changes the *χ*_M_*T*(*T*) magnetic characteristics from a *quasi*-single-step cooperative SCO transition occurring in the 150–200 K region, toward a multi-step broad transformation between 200 and 350 K. Moreover, for both variously solvated phases, the impact of pressure upon spin transition was presented.^1066^ Room-temperature switching of the spin state by solvent exchange was presented for another discrete system built of [Fe^II^(L_pHtp_)_2_] (L_pHtp_H = 2-(pyrazol-1-yl)-6-(1*H*-tetrazol-5-yl)pyridine).^1067^ Here, an as-synthesized system, after the removal of crystallization solvent by the thermal treatment, reversibly sorbs methanol and ethanol which is accompanied by the spin state switching. This effect can be detected by the change of the crystal’s color from yellow (HS state) when exposed to alcohol vapors to red (LS state) which was found for the air-stable phase.^1067^

**Figure 66:**
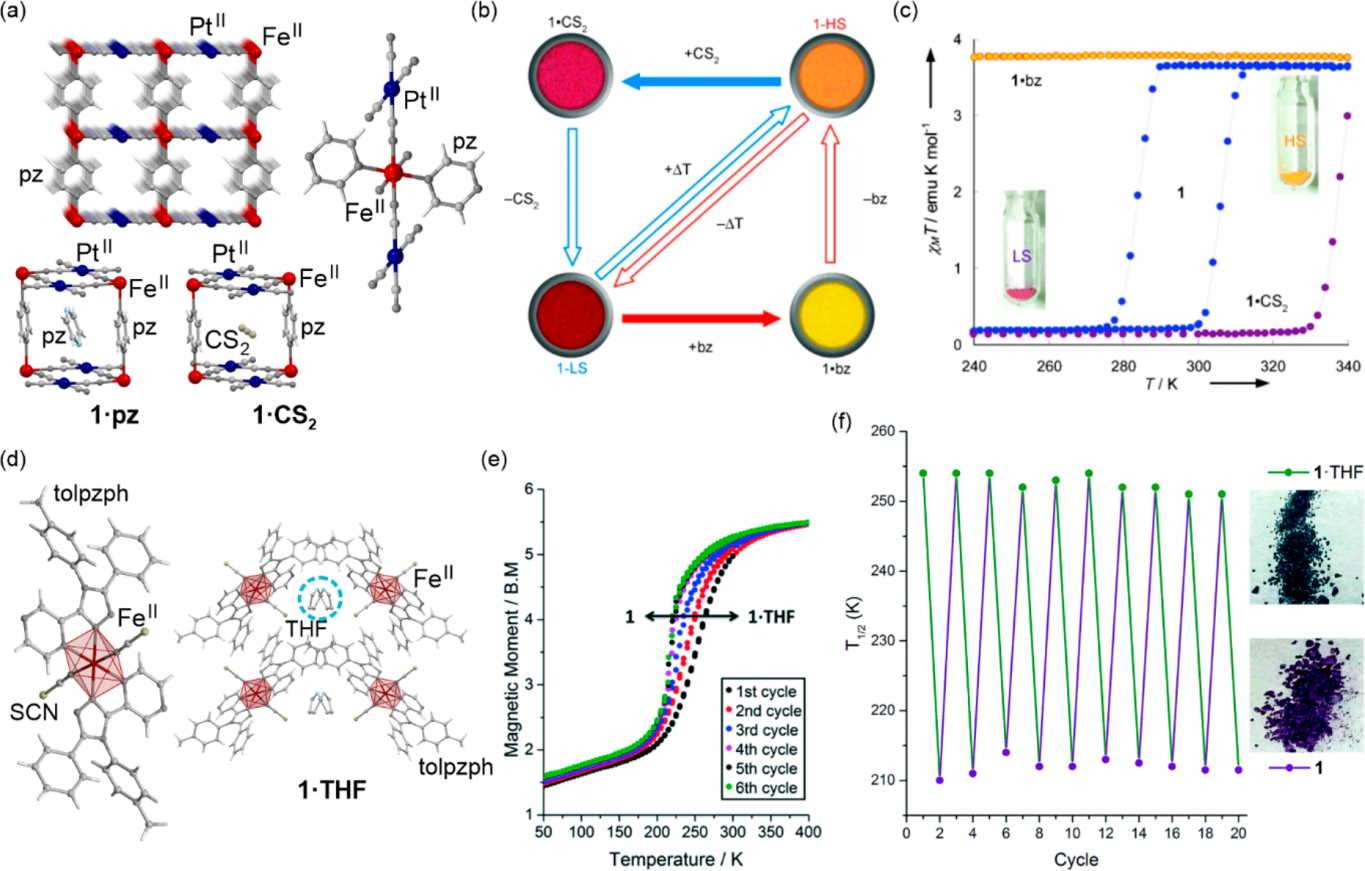
The representative views on {[Fe^II^(pz)][Pt^II^(CN)_4_]}·2H_2_O (pz = pyrazine) Hofmann-type coordination framework, shown together with the insight into the pores occupied by the guest molecules for the pyrazine- and CS_2_-containing phases (a),^316^ schematic presentation of the chemically- and thermally-driven processes, presented together with the respective color changes (HS and LS represents the labels for the high- and low-spin states of Fe(II) sites, respectively) (b), and the related temperature dependences of the *χ*_M_*T* product for the as-synthesized sample and the phases filled with benzene (bz) and CS_2_ molecules (c), the structural views on [Fe^II^(tolpzph)_2_(NCS)_2_]·THF (tolpzph = 4-*p*-tolyl-3-(phenyl)-5-(2-pyrazinyl)-1,2,4-triazole) coordination system, including the positions of the THF molecules of crystallization (d),^1072^ the related temperature dependences of the magnetic moment measured on cooling from 300 to 50 K, and further in the 50–400 K cycles (e), and the curve representing the changes in the *T*_1/2_ values (temperature of the 50% yield of the thermal Fe(II) SCO completeness) upon 10 consecutive cycles of interconversions between the thermally desolvated and the THF-containing phases, shown together with the photos of the respective powder samples (f). Parts (b) and (c) were adapted with permission from ref (316). Copyright 2009 John Wiley & Sons. Parts (e) and (f) were reproduced from ref (1072) with permission from the Royal Society of Chemistry.

Apart from the discrete Fe^II^-based systems, the related Hofmann-type coordination networks were broadly employed to study the impact of chemical stimuli on the SCO behavior.^316,1068−1071^ Most of the materials from this family of compounds crystallize with additional solvent molecules occupying the interstitial space and its post-synthetic removal was found to affect the temperature-variable magnetic characteristics. For instance, {[Fe^II^(proptz)_2_][M^II^(CN)_4_]}·2H_2_O (proptz = (*E*)-3-phenyl-*N-*(4*H*-1,2,4-triazol-4-yl)prop-2-yn-1-imine; M^II^ = Pd^II^, Pt^II^) layered frameworks,^1068^ showing the hysteretic SCO in the 100–150 K range, when thermally desolvated, exhibit the increased cooperativity accompanied by the shift of transition temperature near room temperature. Therefore, at the same time, the optical absorption properties are affected, and while the hydrated phase near room temperature is yellow, the presence of the SCO thermal hysteresis after dehydration in this temperature region may lead toward the observation of strongly absorbing LS state. Such sensitivity of Hofmann-type networks toward guest molecules was employed to modulate the spin state and optical absorption properties by the inclusion of benzene and CS_2_ molecules within the pores of the dehydrated {[Fe^II^(pz)][Pt^II^(CN)_4_]} (pz = pyrazine) framework (Figure 66a-c).^316^ Near room temperature, the dehydrated phase shows a hysteretic behavior in magnetic properties related to the SCO phenomenon; however, when exposed to benzene or CS_2_ vapors, magnetic characteristics are completely modified. For a benzene-treated system, the Fe(II) sites remain in the HS state in the whole temperature range, while the inclusion of CS_2_ molecules stabilizes the LS state, leading to shifted SCO behavior toward higher temperatures. Moreover, different solvents including water, some alcohols, acetone, pyrazine, toluene, pyridine, furan, THF, and more, tend to stabilize the HS state at room temperature, while the CS_2_ impact upon SCO seems to be unique and related to the specific interaction between S-atoms with pyrazine and Pt^II^ centers.^316^ Owing to the thermal memory effect of the desolvated phase near room temperature, by following the absorption properties, it was found that the sample, after the desorption of the guests, can reach a different spin state than before their sorption. Such behavior is apparent for benzene and CS_2_ vapors. Therefore, the orange HS sample of a desolvated network transforms to the red LS state upon sorption of CS_2_ and retains deep red color after its desorption. Then the sorption of benzene reverses the SCO resulting in a yellow powder sample, that upon desorption closes the spectacular full cycle of structural and spin-state transformations.^316^

A similar solvent memory effect is reported for a mononuclear Fe^II^ complex, [Fe^II^(trz-tet)_2_(H_2_O)_4_]·4H_2_O (trz-tetH = 5-(4*H*-1,2,4-triazol-yl)-2*H*-tetrazole) which acts as a methanol and ethanol sensor.^101^ Upon the immersion of pelleted sample in both alcohols, the white pellet turns pink and the spin state change is retained even after vacuum treatment. Then to recover the original spin state, the exposition towards water vapors is necessary. Such behavior, however, suggests a different mechanism of spin-state switching, which is related to the number of aqua ligands within the first coordination sphere of the Fe(II) moiety. Moreover, this complex reveals also the sensitivity of optical absorption properties towards ammonia, HCl, HBr, and hydrazine, but it is only for ammonia that this effect can be attributed to the partial SCO.^101^ Another molecular SCO system showing high sensitivity of light absorption to solvent vapors is built of THF-solvated [Fe^II^(tolpzph)_2_(NCS)_2_] (tolpzph = 4-*p*-tolyl-3-(phenyl)-5-(2-pyrazinyl)-1,2,4-triazole) complexes (Figure 66d–f).^1072^ The THF molecules found in the supramolecular framework can be thermally removed, while the desolvated phase readily recovers to the pristine state when exposed to THF vapors. The temperature dependences of the *χ*_M_*T* in several cycles of heating and cooling reveal a moderate modification of the SCO behavior upon loss of THF molecules. Additionally, despite that the observed SCO transition is incomplete at room temperature, one may observe SCO-related absorption changes at room temperature between the THF-solvated and the desolvated system originating from the different fractions of HS and LS sites. Reversible changes regarding the solvent content were monitored within several cycles of sorption and THF thermal removal by following the value of *T*_1/2_, proving the reversibility and integrity of chemical switching.^1072^

An extraordinary example of a molecule-based magnetic material switchable by the solvent content is related to {[Ni^III^(cyclam)][Fe^II^(CN)_6_]}^−^ (cyclam = 1,4,8,11-tetraazacyclotetradecane) chains accompanied, in the crystal lattice, by additional H_3_O^+^ cations and five water molecules per formula unit.^1073^ The as-synthetized phase for this system behaves as a simple paramagnet in the whole temperature range owing to the LS state of Fe^II^ complexes. Upon dehydration in a vacuum at a slightly elevated temperature, the sample undergoes a transition related to the electron transfer between Ni^III^ and Fe^II^ centers, resulting in the Ni^II^–Fe^III^ pair, where both metal centers bear non-zero spin. The latter results in the increased value of the *χ*_M_*T* product and long-range magnetic ordering below 5 K. Further rehydration of the system leads to partial recovery of the initial state, thus, the reversible electron transfer effect appears in the solid state. However, the studies on single crystals show a clear change in the crystal’s color from yellow for the dehydrated sample to blue after the rehydration.

**Figure 67:**
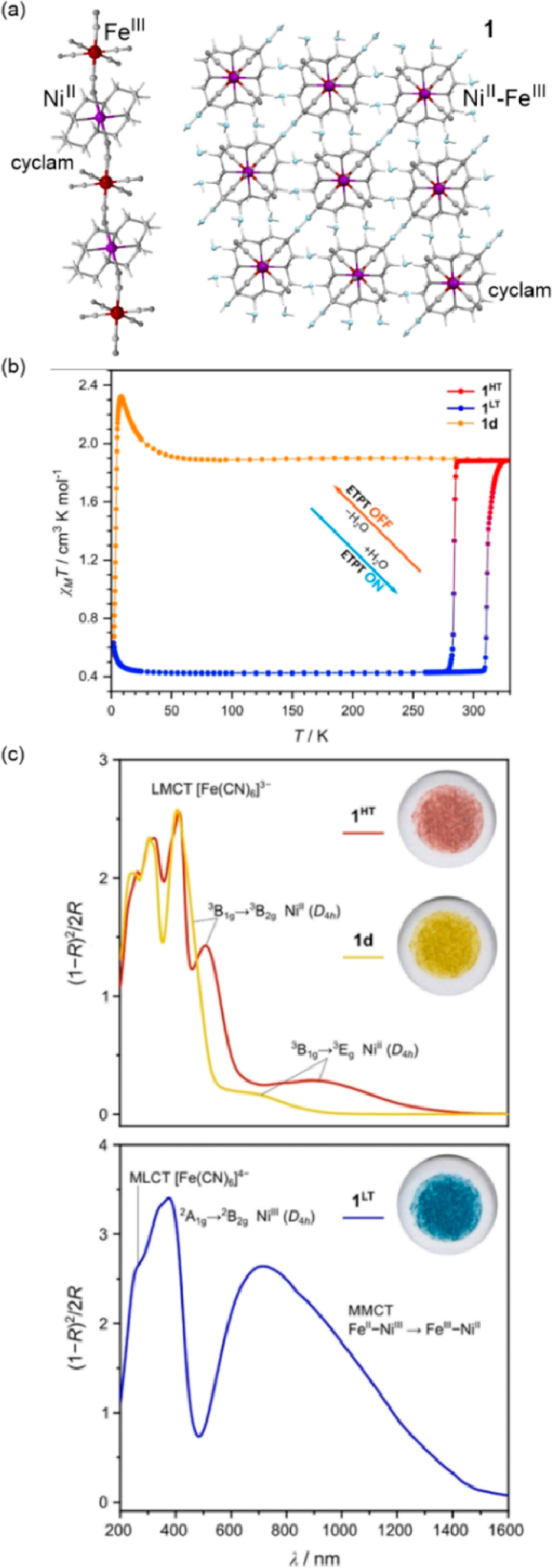
The structure of (NH_4_){[Ni^II^(cyclam)][Fe^III^(CN)_6_]}·5H_2_O (cyclam = 1,4,8,11-tetraazacyclotetradecane) coordination chains and their arrangement in the supramolecular network (a),^366^ the temperature dependences of the *χ*_M_*T* product for the as-synthesized phase (HT and LT represent the labels for its high- and low-temperature phases, respectively, differing in the oxidation states of the metal sites) and the dehydrated phase (**1d**) (ETPT = electron transfer phase transition) (b), and the UV–vis-NIR absorption spectra of the related phases, shown with the assignment of the strongest absorption bands and the photos of the respective powder samples (c). Parts (b) and (c) were adapted with permission from ref (366). Copyright 2020 John Wiley & Sons.

Further studies regarding the analogous chains incorporating the ammonium cations revealed that such a system can be more spectacularly switched by various external stimuli including temperature, pressure, humidity, and light (Figure 67).^366^ While the as-synthetized hydrated phase, (NH_4_){[Ni^II^(cyclam)][Fe^III^(CN)_6_]}·5H_2_O, reveals a distinct thermally-induced electron transfer accompanied by the hysteresis between 284 and 312 K, after its dehydration, the electron transfer pathway is quenched; thus the post-synthetically obtained material shows antiferromagnetic ordering below 4.9 K. Considering room-temperature water sorption properties, a large hysteresis loop within sorption-desorption isotherm is observed. The electron transfer process and the dehydration significantly modify the optical absorption, i.e., the low-temperature Ni^III^–Fe^II^ phase shows an intense broad MMCT band in the vis-NIR region resulting in the deep blue color of the sample. The high-temperature phase, on the contrary, reveals a rather weak absorption in the red part of the spectrum, and strong blue and yellow light absorption resulting in the red color of the sample. The dehydration process slightly shifts the absorption bands related to the crystal field transitions of the *D*_4h_ Ni^II^ complexes, changing the sample color towards yellow. To induce the electron transfer process for the dehydrated sample, external pressure was applied, however, such pressure-induced transition even at 1.16 GPa does not occur completely down to low temperatures. On the other hand, for the original system, the impact of even small external pressure upon the shape and range of the hysteresis loop was observed. The room-temperature photoswitching was also tested for this material but it was attributed rather to the local heating effect. Nevertheless, this system demonstrated an impressive interplay between external stimuli, including chemical ones, and an electron transfer process affecting both optical and magnetic characteristics.^366^

In recent years, several studies have been devoted to the switching of optical and magnetic properties of single-molecule magnets by solvent exchange.^100,1074−1077^ The presence of single-crystal-to-single-crystal transformation for a [Co^II^(L_Brph-terpy_)_2_](DPAS)_2_·dmf·2H_2_O (L_Brph-terpy_ = 4′-(4-bromophenyl)-2,2′:6′,2′′-terpyridine; DPAS^–^ = 4-(phenylamino)benzenesulfonate) molecular system, related to the removal of crystallization water molecules, enables the switching of the magnetic behavior between the spin crossover effect for the hydrated system and SMM behavior for its dehydrated phase.^1078^

**Figure 68:**
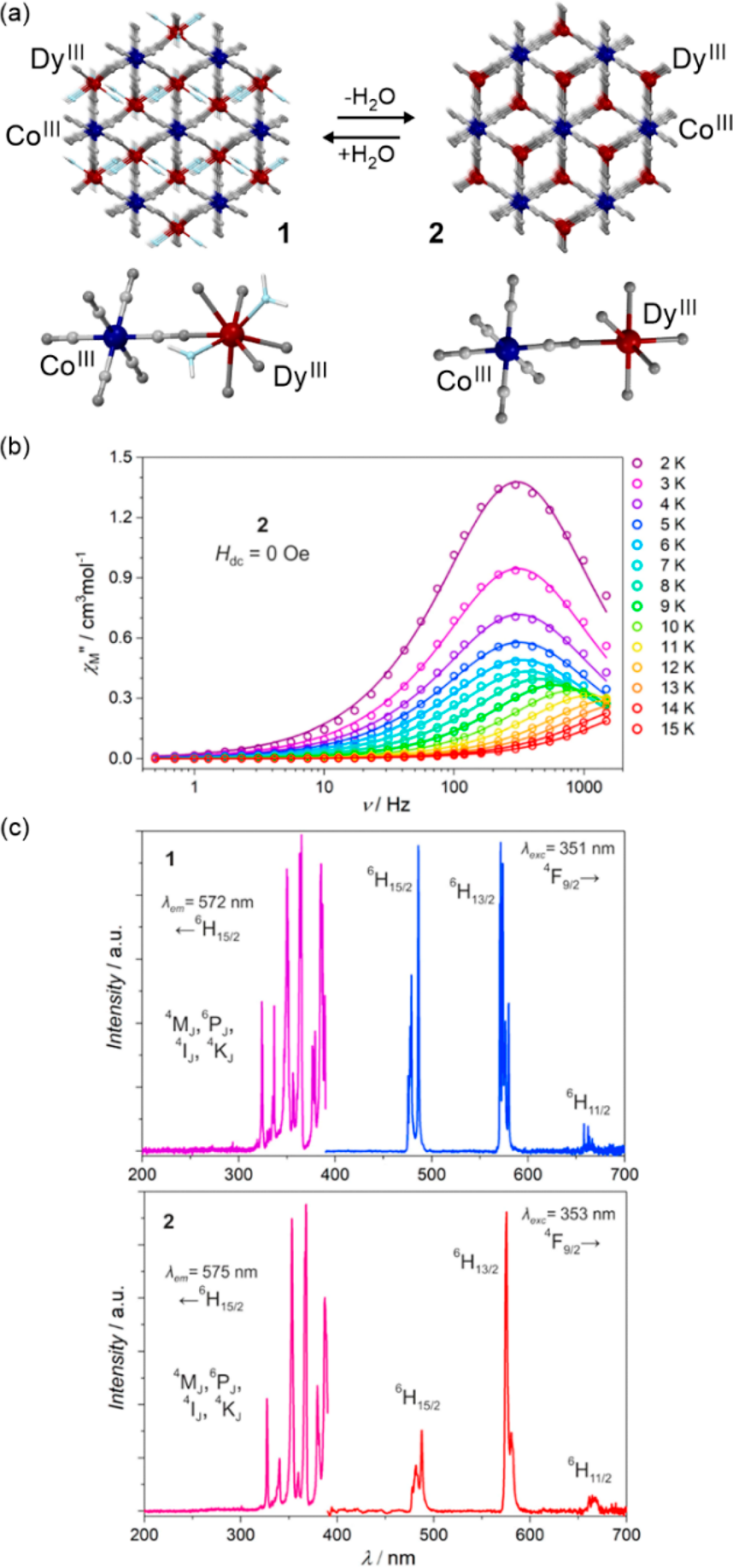
The structural views on {[Dy^III^(H_2_O)_2_][Co^III^(CN)_6_]}·2.2H_2_O coordination network in its as-synthesized hydrated form (**1**) and the dehydrated phase of {Dy^III^[Co^III^(CN)_6_]} (**2**) (a),^794^ the representative *ac* magnetic characteristics demonstrating the SMM behavior occurring for the phase **2** (b), and the low-temperature (*T* = 3.5 K) photoluminescence (excitation – left sides, emission – right sides) spectra for both investigated phases, shown together with the assignment of the main emission components to the selected f–f electronic transitions (c). Parts (b) and (c) were adapted with permission from ref (794). Copyright 2019 American Chemical Society.

Some lanthanide(III)-based molecular magnets were also shown to exhibit solvent-dependent slow magnetic relaxation properties;^100,1074,1075,1077^ however, the related phenomena are slightly different. To separate the impact of structural changes upon the single-ion anisotropy and modification of the phonon modes involved in the Raman relaxation pathway, the *ab initio* theoretical analysis is required, based on proper structural studies. Additionally, for the Ln^III^-based SMMs one may expect to observe the changes both within the SMM effect and the emission spectrum instead of optical absorption properties. Considering the latter, the chemical switching behavior of a dinuclear Dy^III^–Co^III^ complex was reported.^1079^ It exhibits changes in field-induced relaxation dynamics and the pattern of the emission spectra, both induced by the humidity change resulting in the SC-SC transformation related to the removal of the part of water molecules of crystallization and the reorganization of the Dy^III^ first coordination sphere. Further, this strategy was continued by exploring a 3-D cyanido-bridged d–f framework, {[Dy^III^(H_2_O)_2_][Co^III^(CN)_6_]}·2.2H_2_O, which can be reversibly dehydrated at high temperatures to form a {Dy^III^[Co^III^(CN)_6_]} system with trigonal prismatic Dy^III^ complexes (Figure 68).^794^ While the initially obtained octacoordinated Dy^III^ complexes with six N-bonded cyanido ligands and two aqua ligands forming the distorted square antiprism geometry show no slow magnetic relaxation even under the applied magnetic field, the dehydrated system reveals distinct zero-*dc*-field SMM behavior characterized by the energy barrier of 51.8(8) K. Moreover, the emission signal, originating from the f–f electronic transitions of Dy^III^ complexes, changes upon dehydration not only in the splitting of the maxima related to the *m*_J_ level structures but also in the intensity ratio between main emissive components.^794^

Very recently, the dehydration-induced trigonal prismatic geometry was obtained for Tb^3+^ ions embedded in the analogous framework. It results in the SMM property with a large energy barrier of the Orbach relaxation pathway.^134^ For both studied systems of {[Tb^III^(H_2_O)_2_][Co^III^(CN)_6_]}·2.7H_2_O and its magnetically-yttrium(III)-diluted analog, namely {[Tb^III^_0.026_Y^III^_0.974_(H_2_O)_2_][Co^III^(CN)_6_]}·2.7H_2_O, the removal of both crystallization and coordination water molecules improve the magnetic characteristics of the SMM behavior, as well as modify the observed luminescence thermometry effect which is based on the relative intensity between emissive bands related to hot and cold transitions within the Tb^III^ electronic multiplets. As a result, the SMM behavior for the magnetically diluted and dehydrated system overlaps in the wide temperature region with the optical thermometry working range, providing a unique type of multifunctional stimuli-responsive magneto-luminescent molecule-based material.^134^

### 6.2 Electric Field for Switching of Optical Phenomena in Molecule-Based Magnetic Materials

**Figure 69:**
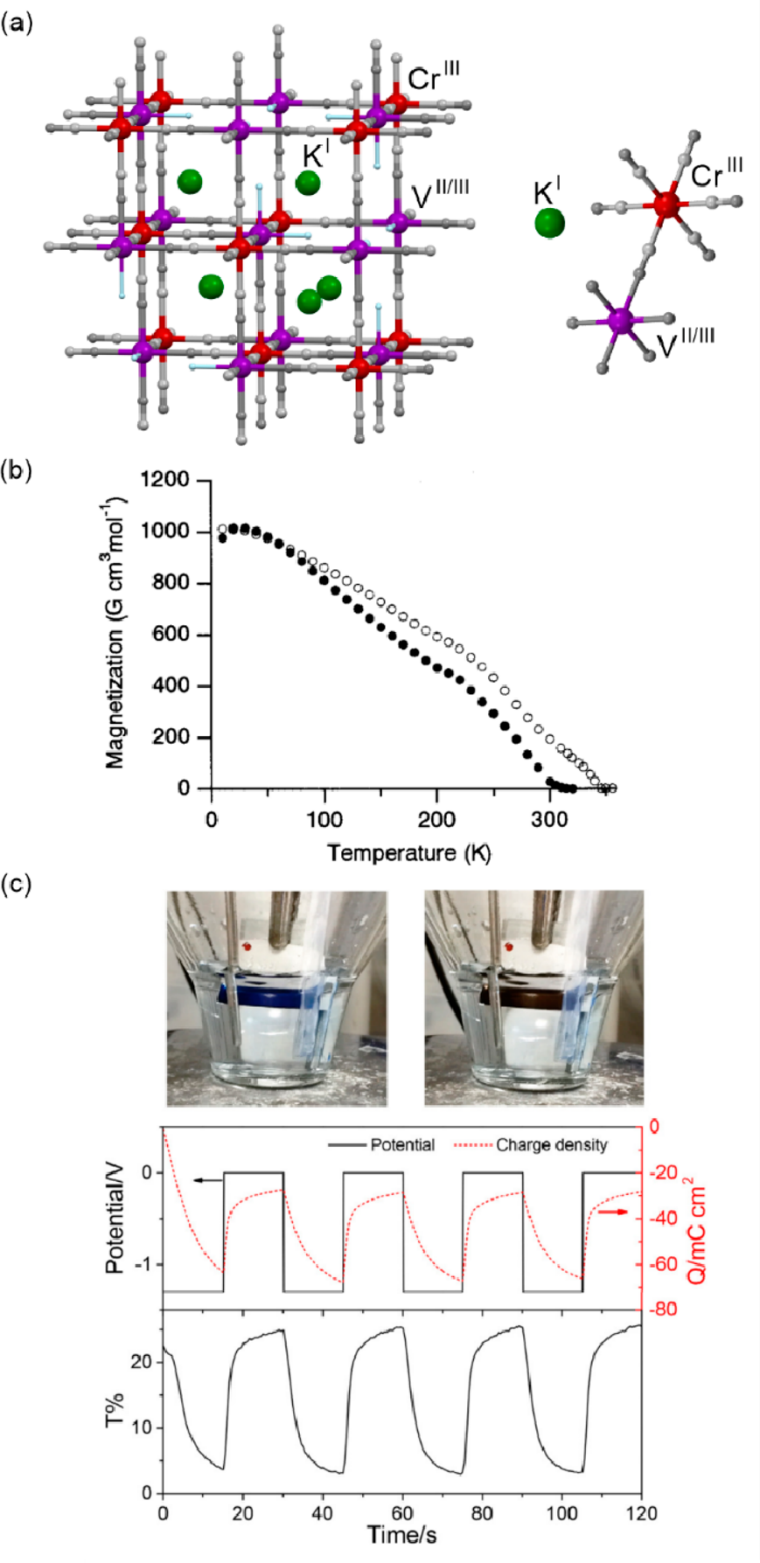
The structural model for the family of K^I^_x_{V^II/III^_y_[Cr^III^(CN)_6_]_z_}·*n*H_2_O coordination frameworks (a),^98,506^ including electrochemically synthesized K^I^_0.31_{V^II^_0.49_V^III^_0.51_[Cr^III^(CN)_6_]_0.94_}·6.5H_2_O and K^I^_0.61_{V^II^_0.97_V^III^_0.03_][Cr^III^(CN)_6_]_0.88_}·7.2H_2_O·0.4EtOH phases, for which the temperature dependences of the magnetization are presented (b) (filled black and empty circles, respectively), and the representative views on the electrochromic switching of this type of molecule-based magnets (c), shown by the photos of the related films in the electrochemical cell (in aqueous solution of KCl) at 0 V (left) and −1.3 V (right), the time-sequence of the applied potential and corresponding charge density (central part), and the related changes in the transmittance at 465 nm (bottom). Part (b) was adapted with permission from ref (994). Copyright 2000 American Chemical Society. Part (c) was reproduced with permission from ref (1083). Copyright 2017 Elsevier Publishing.

Another way to externally switch the optical properties of a material, which is a convenient way in the context of its potential use in novel electronic devices, relies on the application of an electric field or current to the thin film samples or single crystals. When absorption properties are strongly affected by an electric field, the resulting electrochromic material may be employed for the construction of smart windows or mirrors, sensors, and light modulators.^156,162,1080,1081^ Additionally, it was postulated that if a material combines electrochromic behavior with a long-range magnetic ordering, then modulation of light polarization through the Faraday effect may be achieved.^1082^ Therefore, some preliminary studies were performed on opto-magnetic molecule-based materials, especially exploring Prussian blue analogs (PBAs). For instance, the weakly crystalline phases of mixed-valence V^II/III^–Cr^III^ PBAs, which were synthesized as thin films revealing above-room-temperature critical temperatures of spin ordering, were employed for the observation of electric-field-induced switching of optical absorption (Figure 69).^994,1083^ The two reported systems of the exact complex formulas of K^I^_0.31_{V^II^_0.49_V^III^_0.51_[Cr^III^(CN)_6_]_0.94_}·6.5H_2_O and K^I^_0.61_{V^II^_0.97_V^III^_0.03_[Cr^III^(CN)_6_]_0.88_}·7.2H_2_O·0.4EtOH were obtained using the variable-potential electrochemical deposition, showing then *T*_c_ values of 310 and 345 K, respectively.^994^ Later, the related thin films of these types of materials were prepared and studied from the viewpoint of electric field sensing.^1083^ Upon application of voltage of ca. −0.5 V, the decrease in the transmittance was observed at 540 nm, which was associated with the increase in the absorption of the MMCT (metal-to-metal charge transfer) band between V^II^ and Cr^III^ centers. Then down to −0.85 V, a continuous decrease of transmittance in this region is observed. Further decrease of potential to −1.22 V modifies the transmittance at 465 nm. An additional absorption band, which appears in this region, was attributed to the MLCT transition within [Cr^II^(CN)_6_]^4–^ moieties to empty π* orbitals of the cyanido ligands. Moreover, the simultaneous decrease of absorption at 800 nm, which is attributed to the forbidden metal-centered transition of [Cr^III^(CN)_6_]^3–^ units, was detected. As a result, upon cycling the voltage between 0 and −1.3 V, the color of the sample is switched between blue and black.^1083^ Quantitively, these changes can be followed by the transmittance at 465 nm, and for the selected film, the transmittance at this wavelength varies between 25% for 0 V down to ca. 3% at −1.3 V. As a standard characteristic parameter of the electrochromic performance, the coloration efficiency, *η*, was determined for both switching modes. From the dependence of optical density as a function of applied charge density to the film, linear dependencies were fitted, giving *η* = −25.2(2) cm^2^ C^–1^ and *η* = −32(1) cm^2^ C^–1^ for blue to black and black to blue changes, respectively.^1083^ Apart from the high-*T*_c_ V^II/III^–Cr^III^ PBAs, the thin film samples of classical Fe^III^–Fe^II^ PBA were analyzed from the point of electrochromic modulation.^1084^ Such electrodeposited films for a specific speed of stirring the precursor solution were shown to reveal improved durability within several cyclic voltammetry cycles. These materials under different applied potential values reveal four colors of the film including quasi-transparent, blue, green, and yellow. Moreover, due to such complicated changes in the absorption spectrum, by employing the specific electric potential, one can selectively switch between the states transmitting in three distinct modes of operation: transmitting vis and NIR light, only visible light, or being non-transparent in both regions.^1084^

**Figure 70:**
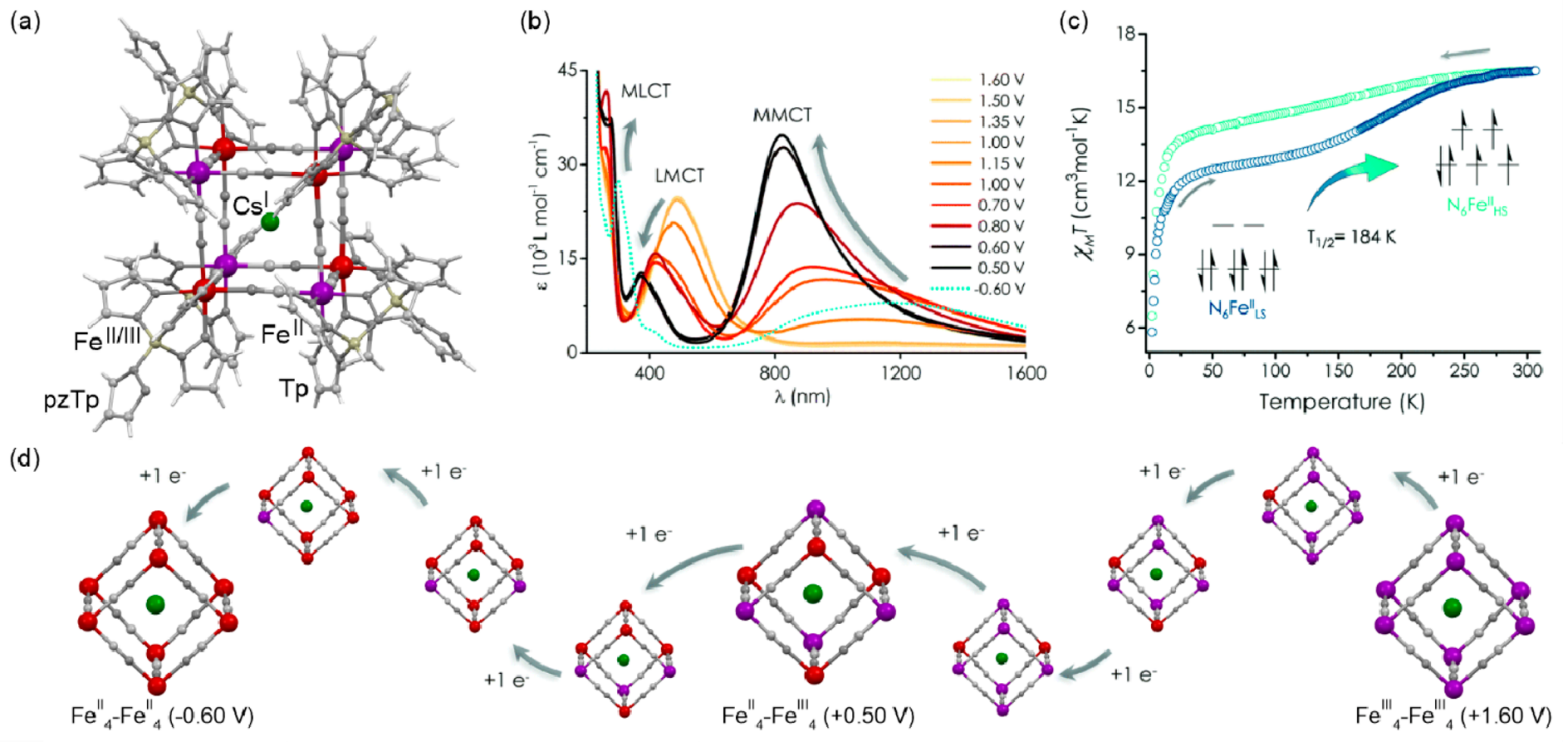
The structure of Cs^I^{[Fe^II^(Tp)(CN)_3_]_4_[Fe^II^_1_Fe^III^_3_(pzTp)_4_]} (Tp = hydrotris(pyrazol-1-yl)borate; pzTp = tetrakis(pyrazolyl)borate) cluster (a),^387^ the changes in the UV–vis-NIR absorption spectra at modulated applied potential with the indicated origin of main absorption bands (b), the temperature dependences of the *χ*_M_*T* product for freshly prepared crystals of the clusters exhibiting the SCO effect (blue curve) and the subsequent curve measured after heating to 310 K that indicate the irreversibility of the transition (cyan curve) (c), and the scheme of the 9 redox states of the cluster accessible through cyclic voltammetry (d). Parts (b), (c), and (d) were reproduced from ref (387) with permission from the Royal Society of Chemistry.

Molecular analogs of cesium-containing PBAs were also employed to study the impact of the electric field on the optical absorption properties. To construct such Cs^I^{M^II/III^_4_Fe^II/III^_4_} cages, R. Lescouëzec and co-workers employed [Fe^III^(Tp)(CN)_3_]^−^ (Tp = hydrotris(pyrazol-1-yl)borate) preorganized entities and combined them with Mn^2+^ or Fe^2+^ ions in the presence of the pzTp (pzTp = tetrakis(pyrazol-1-yl)borate) ligand (Figure 70).^387,1085^ As the result, molecular systems of the general formula of Cs{[M(pzTp)]_4_[Fe(Tp)(CN)_3_]_4_}·*n*(solv) (M = Mn, Fe), were obtained. Their structure resembles the core of PBAs with eight cyanido-bridged metal atoms forming a cage encapsulating the alkali metal ion. For the Fe-based analog, during the synthesis, the electron transfer occurs between iron centers, thus the [Fe^II^(Tp)(CN)_3_]^2–^ complexes are detected in the final system.^387^ Moreover, for the N-bonded centers, three of them are in the +III formal oxidation state, while the last remains as a Fe^2+^ cation. The latter at room temperature remains in the HS state, and within the magnetic measurements, SCO behavior can be observed in the first cycle of heating. However, heated back to 300 K, the SCO behavior becomes irreversible, probably due to the thermal desolvation of the sample. While dissolved in different organic solvents, the integrity of the Cs{Fe_4_Fe_4_} cages is preserved, and the determined oxidation states of the Fe centers are retained. Upon cycling the electric potential, the impressive set of redox states of these cages can be established. Such a reversible multi-redox activity was found to change the color of the solution, due to the change within the intensity of MLCT, LMCT, and MMCT bands (Figure 70).^387^ Moreover, when the part of Fe centers is exchanged with Mn, the cluster grows as a mixed-valence system for both d-block metal ions.^1085^ The resulting cage comprises three Mn^II^ and one Mn^III^ complexes, linked by two [Fe^II^(Tp)(CN)_3_]^2–^ and two [Fe^III^(Tp)(CN)_3_]^−^ units. Direct-current magnetic studies for the solid-state sample suggest, that upon heating above room temperature, the onset of an electron transfer process appears between the LS Fe^II^ and HS Mn^III^ centers towards Fe^III,LS^–Mn^II,HS^ pairs. In contrast to the previously described system, here only the [Fe(Tp)(CN)_3_]^−/2–^ units reveal redox activity in the solution. As a result, while increasing the electric potential, the enhancement of the LMCT band is observed, while a drop in the applied voltage enhances the MLCT-related absorption.^1085^

Among the other molecular systems suitable for the electrochromic studies, the Fe^II^- and Ru^II^-based potentially magnetic chains with ligands possessing the 2,2′:6′,2″-terpyridine chelating groups, and a triphenylamine core, were shown to dramatically switch the absorption properties as thin films upon application of relatively small electric potential.^1086^ This is due to their ability of dual oxidation of both the metal center and the organic core, thus for the Fe^II^-based material the color changes from red to green at 1.0 V. Similar changes are observed for the Ru^II^ derivative, however, their transparency is overall higher. Although the magnetic properties of the obtained materials were not investigated, the potential of triarylamine core combined with redox-active d-block metal complexes, which can provide various magnetic characteristics, in the construction of electrochromic materials was proven.^1086,1087^

### 6.3 Pressure and Mechanical Force for Switching of Optical Phenomena in Molecule-Based Magnetic Materials

The pressure and mechanical force were widely recognized as efficient external stimuli for switching light absorption, emission, and other optical effects.^1088−1091^ Simultaneously, the pressure-induced structural changes in molecular materials were found to be a tool for switching a broad range of magnetic phenomena from spin transitions, through magnetic ordering to SMM characteristics.^110,268,1092,1093^ Concerning the potential switching of both magnetic and optical effects in molecule-based materials, the spin transition systems were reported to be the most promising. As most iron(II) complexes showing the spin-crossover behavior reveal the impact of pressure upon the temperature and cooperativity of the related magnetic transition which usually leads to optical absorption modulation (see section 2), such magnetic molecular systems appear to be suitable for the construction of dual output pressure switches or sensors employing also optical absorption features.^268,434,1092^ It is well known and already presented in section 2 that the low-spin Fe^II^ octahedral complexes are usually strongly colored due to their spin-allowed inter-configurational electronic transitions between the fully occupied *t*_2g_ end empty *e*_g_ states. They disappear for the high-spin state for which the sample’s color is usually pale yellow or green. Such a dramatic color change may serve as an additional feature to study the impact of pressure, along with the concomitant changes in magnetic characteristics. As an example, both pressure and temperature-induced changes upon absorption properties were studied for [Fe^II^(hyetrz)_3_]I_2_·0.5EtOH (hyetrz = 4-(2′-hydroxyethyl)-1,2,4-triazole) molecular system.^1094^ Under ambient conditions, its powder sample is almost white which corresponds to the HS state of the embedded Fe(II) centers. The temperature-variable refractivity spectra were used to determine the HS fraction in the range of thermally-induced SCO that occurs for this compound. The observed hysteretic behavior reflects the first-order character of the spin transition. On the other hand, the application of contact pressure at room temperature induces a series of changes in the optical absorption spectra, especially corresponding to the ^1^A_1g_→^1^T_1g_ and ^5^T_2g_→^5^E_g_ electronic transitions, which results in the sample turning purple under tens to hundreds of MPa. The related pressure dependence was analyzed, both following peak intensities and positions, and it was found that under 100 MPa, the pressure-induced SCO is quasi-complete at room temperature. Moreover, for the cooling cycle of the thermal hysteresis, it was possible to use the refractivity spectra to follow the thermal variation of the HS fraction under 0, 50, and 100 MPa of the external pressure.

**Figure 71:**
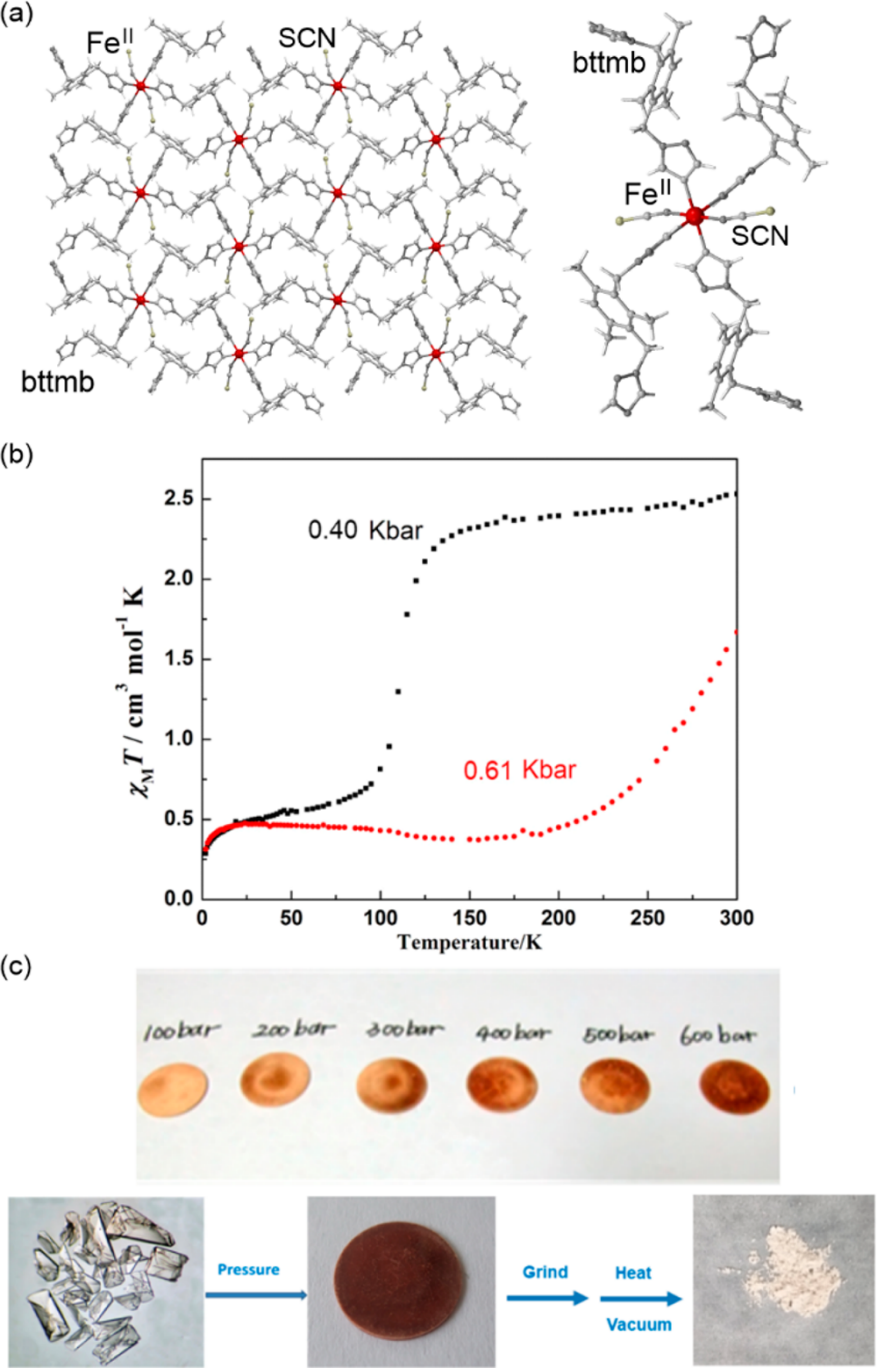
The representative structural views on [Fe^II^(bttmb)_2_(SCN)_2_] (bttmb = 1,3-bis(1,2,4-triazol-1-ylmethyl)-2,4,6-trimethylbenzene) layered coordination network (a),^1095^ its temperature dependences of the *χ*_M_*T* product under the indicated pressures (b), and the color changes of this compound (mixed with KBr) under various indicated pressures, shown together with the color changes from transparent colorless crystals to purple under the pressure, followed by the color recovery achieved by grinding and heating under vacuum (c). Parts (b) and (c) were adapted with permission from ref (1095). Copyright 2018 American Chemical Society.

An enormous impact of the external pressure was presented for layered [Fe^II^(bttmb)_2_(SCN)_2_] (bttmb = 1,3-bis(1,2,4-triazol-1-ylmethyl)-2,4,6-trimethylbenzene) material (Figure 71).^1095^ Under the ambient pressure, it shows no SCO phenomenon within the 2–300 K range. However, upon applying *p* = 0.4 kbar, an abrupt drop of the *χ*_M_*T* appears on cooling at ca. 100 K, which is related to the incomplete HS to LS conversion. At the increased pressure of 0.61 kbar, the SCO transition loses in cooperativity and the increase of the HS fraction upon heating becomes gradual. Nevertheless, a dramatic increase in the transition temperature is observed, as the SCO phenomenon is still incomplete at 300 K at 0.61 kbar. From the viewpoint of optical properties, the sample pressed with KBr into the pellet revealed a memory effect related to the pressure-induced SCO. With the increasing pressure used for the formation of the pellets, the intensity of the light absorption increased. To reverse the color change, it was found necessary to grind the pellet and then heat the sample under vacuum. The integrity of the complex under these conditions was confirmed by the XPS measurement.^1095^

Considering that the external pressure may modify the absorption properties through the occurrence of the SCO transition, the analysis of the structural data obtained under pressure may give additional information about the effect itself. Such set of measurements was performed, for instance, for a 3-D {[Fe^II^(pym)(H_2_O)][Ag^I^(CN)_2_]_2_}·H_2_O (pym = pyrimidine) Hofmann-type framework.^318,1096^ This system undergoes the SCO effect with a hysteretic temperature dependence for cooling and heating cycles, showing the pressure impact upon the temperature range and width of thermal hysteresis. Additionally, the detailed studies of the absorption spectra, measured under variable pressure at 300 K, enable plotting the pressure dependence of the HS fraction, also showing a hysteretic behavior in the ca. 0–1 GPa range. Visibly, the pressure-induced SCO behavior manifests as the change of the crystal color from yellow at the ambient pressure, towards red after its application. The X-ray diffraction data for a single crystal under 0 and 0.5 GPa were collected at 323 K to study both the HS and LS states being thermodynamically stable at these conditions.^318^ The SCO behavior appears only for one of the two symmetry-independent centers, which is coordinated by the two N-atoms of pyridine rings and four N-atoms of cyanido ligands. The overall cell volume for the HS spin state is only ca. 2.4% larger than for the LS state, as the LS-to-HS transition is associated mainly with an elongation within the *a* direction and a contraction along the *c* axis. These findings under the same external conditions are obtained due to the unique pressure-based SCO hysteresis, established using the pressure-variable optical measurement.^318,1096^

An interesting example of the external modification of the spin-state was lately reported for the Fe(III)-based material obtained by the oxidation of layered [Fe^II^(bib)_2_(SCN)_2_] (bib = 4,4′-bis(1-imidazolyl)bibenzene) MOF upon the exposition to the air atmosphere.^1097^ For the bulk sample of the oxidized material, all the centers remain in their HS (*S* = 5/2) state, as shown by magnetic measurements, supported by Mössbauer spectroscopy. However, upon formation of the nanolayer by mechanical exfoliation method, ca. 57% of the metal centers undergo the spin transition toward the LS (*S* = 1/2) state. Such change between bulk sample and a nanolayer strongly modifies the photocatalytic activity of this MOF material toward the selective reduction of CO_2_. Electron transfer systems were also studied from the viewpoint of optical and magnetic properties switching by applying external pressure. Such works were shown for 2-D Cs^I^{[Co^II^(3-CNpy)_2_][W^V^(CN)_8_]}·H_2_O (3-CNpy = 3-cyanopyridine) coordination network showing the electron transfer-coupled spin transition (ETCST) between Co^II,HS^–W^V^ and Co^III,LS^–W^IV^ pairs upon cooling.^1098^ At room temperature, the ECTST phenomenon can be induced by the application of ca. 0.5 GPa hydrostatic pressure, as proven by magnetic measurements. On the other hand, this transition was found to occur with a significant change of the sample color from red to green at 0.44 GPa, as well as the presence of pressure-oriented bistability can be noted. The reported behavior is an effect of the large pressure-induced shift of the transition temperature, reaching 250 K/GPa slope for the upper critical temperature, which was attributed to the large change in volume within the ETCST.^1098^

The uncommon example of the electron transfer process for RE^3+^–[Fe^III^(CN)_6_]^3–^ (RE = rare earth metals) systems was also investigated under external pressure.^1099^ For {[Y^III^(dmf)_4_(H_2_O)_3_][Fe^III^(CN)_6_]}·H_2_O molecular material, pale yellow crystals reversibly change to red when the pressure reaches ca. 4.5 GPa. As this process is only a little RE^3+^-dependent, the Nd^3+^ analog also undergoes such a color change above ca. 3 GPa, while after the exchange of the polycyanidometallate anion to [Co^III^(CN)_6_]^3–^, even the pressure of 10 GPa does not influence the color of the sample. The variable-pressure XANES data suggest the effect being a pressure-induced ligand-to-metal electron transfer process at the Fe(III) site resulting in the reduction of the [Fe^III^(CN)_6_]^3–^ to the [Fe^II^(CN)_6_]^2–^ moiety.^1099^

**Figure 72:**
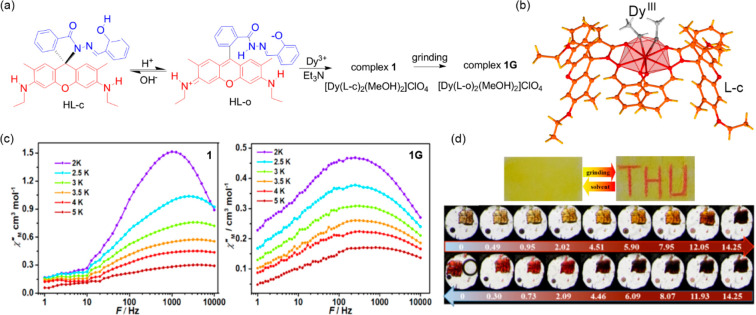
Schematic presentation of the pH-driven equilibrium between the ring-closed (HL-c) and ring-opened (HL-o) forms of the rhodamine 6G salicylaldehyde hydrazone ligand and its subsequent reaction with Dy(III) towards [Dy^III^(L-c)_2_(MeOH)_2_](ClO_4_) molecule (**1**) and its further grinded form of **1G** (a), the structural view for the complex **1** (b),^1100^ the frequency dependences of the out-of-phase *ac* magnetic susceptibility for **1** and **1G** at the indicated temperatures (c), and the visualization of the reversible color change occurring upon grinding and solvent fuming, shown together with the micrographs of the compound **1** under indicated sequence of modulated external pressure (d). Parts (a), (c) and (d) were adapted with permission from ref (1100). Copyright 2021 American Chemical Society.

An unprecedented case of the modification of the crystal structure upon the application of mechanical stress was found for a [Dy^III^(L-c)_2_(MeOH)_2_]ClO_4_·Et_2_O·H_2_O·0.5MeOH molecule-based system employing the rhodamine 6G salicylaldehyde hydrazone (HL-c) which, depending on the conditions, adopt either closed (c) or the open (o) form (Figure 72).^1100^ The L-c ligand itself is not mechanochromic; however, when the complex with lanthanide ion is formed, even under gentle grinding, the sample’s color changes from yellow to red, which is attributed to the ring opening. The same effect can be induced by the application of hydrostatic pressure, and, to reverse it, the presence of a methanol atmosphere is necessary. The as-synthesized complex of Dy^III^ itself is coordinated by the two O-atoms of MeOH molecules and by the two tridentate ligands. When the pressure-induced ring opening occurs, the powder X-ray diffraction pattern reveals an amorphous character but is recovered upon MeOH addition. With the application of hydrostatic pressure in the 0 to 11 GPa range, the color of the sample changes from yellow to orange, and turns brown above ca. 12 GPa up to ca. 14 GPa. Then when the pressure is removed to eliminate the impact of intermolecular interactions, a clear red color is observed being the result of the ring opening. As the ligand itself is fluorescent, the changes within the emission spectra can also be monitored under pressure. Interestingly, for the as-synthetized material, the maximum of the emission appears at 485 nm, and by increasing the pressure its value decreases giving rise to a new emission band at ca. 580 nm, attributable to the emission of the ring-opened form. This complex of Dy^III^ also reveals field-induced SMM behavior. However, the effect of QTM and the large distribution of the relaxation times are both visible. Although there is no significant change in the energy barrier between the as-synthetized and pressure-treated form of the complex, clearly the open-form of the ligand results in slower relaxation dynamics, presumably due to reduced QTM.^1100^

## 7 Optical Multifunctionality in Molecule-Based Magnetic Materials

The scientific attention towards inducing several physical properties in a single-phase material increases every year within the materials chemistry community.^744,909,1101−1104^ The multi-functional systems can be, in general, obtained by the preparation of composites where the separate components reveal different physical phenomena. This usually limits the interaction between them down to the shared surface area.^1102,1105−1107^ Alternatively, the multifunctionality is accessible within the synthesis of single-phase systems showing at least two distinct features incorporated at the molecular level.^100,121,144,694,909,1101^ The latter can be achieved by different means but the most rational pathway is given by the bottom-up approach where each property originates from a specific applied molecular component.^1108−1111^ This route opens the further pathway for convenient processing of the multifunctional material into the nanoscale fulfilling the current technological demand for miniaturization. Miniaturization itself is indeed one of the reasons driving the studies regarding multifunctional materials,^1112^ especially when two physical features simply coexist in a single phase (refs (690, 713, and 1113−1115)). Nevertheless, scientists in the field opt to search for some level of coupling between them. This can result in the influence of one property upon the other, or even lead to the appearance of an additional feature, i.e., a physical cross-effect stemming from the multifunctional character of the system.^121,143,892,1028,1104,1116,1117^ Optical properties especially well interact with other phenomena such as magnetism, as was presented in the previous parts of this review, resulting in bifunctional, opto-magnetic materials (see, e.g., sections 4. and 5.). However, the design of materials with a higher degree of multifunctionality is a tempting, but at the same time, difficult task for synthetic chemists. Within the molecular approach, this usually requires either employing at least three different molecular components within a single-phase system^1118^ or including preorganized bifunctional molecular precursors,^1119^ while another successful pathway involves the conjunction of molecular design and composites formation.^1120^ In this context, special attention should be given to optical multifunctionality. For this reason, we should distinguish three highly-desired optical phenomena, which can be further combined or coupled with non-optical physical features: (a) photochromism, (b) luminescence, and (c) nonlinear optical or chiroptical properties. Photochromism (a) originating from the photoinduced changes in a material, when combined with other optical functionalities (b or c), can result in the photoswitching of the second property, the read-out signal, thus, such materials are considered good candidates for optical storage devices.^161,1121^ The conjunction of photoluminescence with nonlinear optical properties (b plus c) gives the possibility to utilize both phenomena in multipathway energy conversion materials or induce two-photon-processes-related photo-luminescence.^1122−1124^ Last but not least, luminescent chiral systems (b plus c) reveal circularly polarized luminescence (CPL).^892,1116^ This effect makes chiral luminophores promising for the construction of CPL diodes for future display devices, e.g., 3-D displays, and photonic technologies.^1125−1127^ The above division was selected within this section to guide us through the limited but continuously growing number of multifunctional molecular magnetic materials exploring various optical features. Therefore, in section 7.1, photoswitchable magneto-luminescent molecular systems were collected and discussed, while section 7.2 contains the overview of chiral and polar magnetic materials with photochromic molecular units. Then in section 7.3, we will present multifunctional magnetic luminophores where the chiral or polar character of the framework was generated. In the final part (section 7.4), unique materials examples that exhibit the combination of a few different optical and non-optical physical effects in molecule-based magnetic materials were depicted.

### 7.1 Photoswitchable Luminescent Molecule-Based Magnetic Materials

As presented in the previous sections (e.g., 2 and 3), for some of the spin-crossover (SCO) materials, the effect of light-induced excited spin-state trapping (LIESST) can be generated at cryogenic temperatures. Additionally, in recent years a considerable number of coordination systems, combining both SCO and visible luminescence, was constructed aiming at spin transition-induced emission modulation (see section 4.2). As an important step forward, one may consider combining both luminescence and LIESST activity within a Fe(II)-based SCO material to generate a photoresponsive luminescent molecular system where low-temperature light irradiation would induce changes in the emission characteristics due to modification of the Fe(II) electronic structure. However, for most systems combining the LIESST effect centered at Fe(II) centers with photoluminescence, only the correlation between temperature-driven spin transition and emission intensity was presented. This is, e.g., the case of [Fe^II^(L_ndptmi_)_2_(NCS)] and [Fe^II^(L_ndptmi_)_2_(NCSe)] (L_ndptmi_ = (naphth-1-yl)-*N*-(3,5-di(pyridin-2-yl)-4H-1,2,4-triazol-4-yl)methanimine) complexes,^740^ as well as the system containing pyrene-decorated pybox-type ligands, [Fe^II^(pyr-pybox)_2_](ClO_4_)_2_ (pyr-pybox = 2,2′-(4-(pyren-1-yl)pyridine-2,6-diyl)bis(4,4-dimethyl-4,5-dihydrooxazole)),^741^ and [Fe^II^(napht-trz)_6_](tcnsme)_2_·4CH_3_CN (napht-trz = *N*-(1,2,4-triazol-4-yl)1,8-naphthalimide; tcnsme^–^ = 1,1,3,3-tetracyano-2-thiomethylpropenide anion) assembly.^737^ Yet, {[Fe^II^(bpben)][Au^I^(CN)_2_]_2_} (bpben = 1,4-bis(4-pyridyl)benzene) Hofmann-type coordination network with encapsulated pyrene guest molecules, shows, upon both thermal phase transition and under 520 nm light irradiation at 10 K, changes in the emission spectra related to the luminescence of guest molecules themselves and exciplexes originating from their π–π interactions with the bpben linkers.^742^ Similar results were reported for mononuclear [Fe^II^(L_pdptmi_)_2_(NCS)_2_] (L_pdptmi_ = (pyrene-1-yl)-*N*-(3,5-di(pyridin-2-yl)-4H-1,2,4-triazol-4-yl)methanimine) complexes, where light irradiation at 10 K induces a noticeable increase of the emission intensity due to LS to HS transformation.^732^ Upon dissolution in methanol, the SCO effect is preserved which is also accompanied by the appearance of visible luminescence originating from the L-N_4_dte ligands. UV light irradiation at room temperature for the solution containing [Fe^II^_2_(L-N_4_dte)_3_]^4+^ helicates results in significant changes in optical absorption, especially related to the appearance of an additional band centered at ca. 550 nm. Such a change originates from the dte ring closure, thus upon following visible light irradiation reversible process of ring opening occurs, which is not observed in the solid state. Ligand-based photoinduced changes result in modified SCO characteristics and ligand emission. UV irradiation quenches the emission and shifts the emission maxima towards higher energies while visible light recovers the original signal centered at ca. 440 nm.^267^

**Figure 73:**
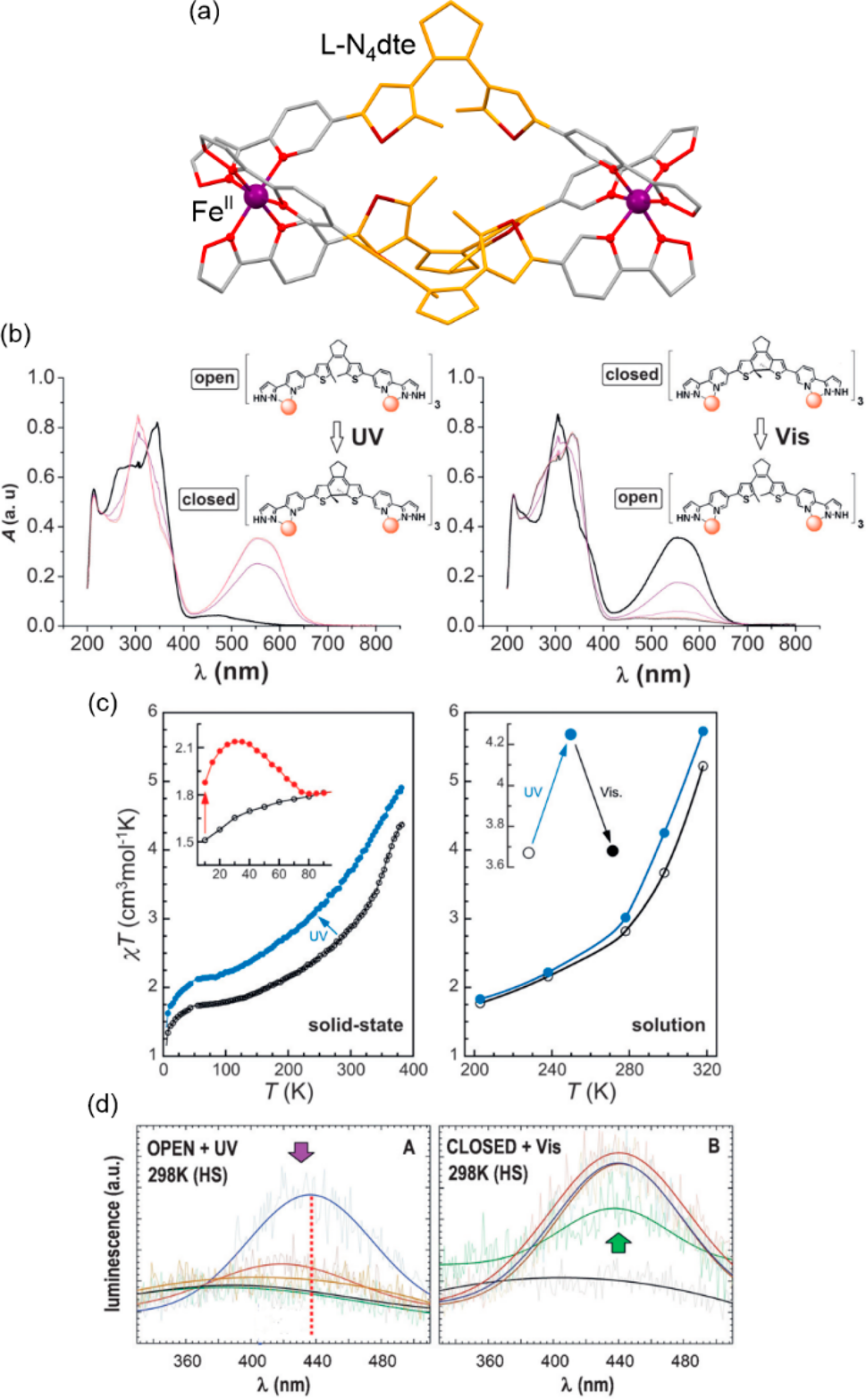
The structure of [Fe^II^_2_(L-N_4_dte)_3_](ClO_4_)_4_·(solvent) (L-N_4_dte = 1,2-bis(5-(2-(pyrazol-3-yl)-pyridin-5-yl)-2-methylthiophen-3-yl)cyclopentene) dinuclear molecule (a),^267^ electronic absorption spectra of the [Fe^II^_2_(L-N_4_dte)_3_]^4+^ ions in methanol and their evolution under UV light irradiation (left part), and the evolution of the absorption spectrum for the UV light irradiated solution under the subsequent visible light irradiation (right part) (b), the temperature dependences of the *χ*_M_*T* product in the solid state for the methanolic solvate of the compound (left part) and the analogous curves for the compound dissolved in the methanol solution (right part), for both cases before and after UV light irradiation, shown together with the partial LIESST effect in the solid state at 10 K (left inset) and the photoreversibility in the solution at 298 K (right inset) (c), and room-temperature fluorescence spectra of these molecular cations in the methanolic solution gathered using the 290 nm light excitation upon the exposition to the UV irradiation (left part) and after the subsequent exposition to visible light (right part) (d). Parts (b), (c), and (d) were adapted with permission from ref (267). Copyright 2017 John Wiley & Sons.

A different method of light control over both SCO property and photoluminescence was reported by M. Estrader, G. Aromi, and co-workers.^267^ By assembling two Fe(II) centers with three bis(pyrazolylpyridyl) ligands incorporating dithienylethene units (L-N_4_dte), [Fe^II^_2_(L-N_4_dte)_3_]^4+^ helicates are formed (Figure 73). They reveal SCO behavior accompanied by the LIESST effect at cryogenic temperatures related to the conversion of 7% Fe(II) LS sites in the solid state.

While for the SCO systems, the photoinduced (LIESST) effect has a rather predictable influence on both magnetic and luminescent properties, a solid-state photoreaction of organic photochromes was found to reveal a non-trivial influence upon the relaxation processes of lanthanide(III) single-molecule magnets. In these cases, the change of luminescence is also observed but it is usually based on the modification of the electronic structure related to the employed photochrome itself, thus no direct coupling with the magnetic behavior of Ln(III) center is present. This scenario, by employing Dy(III) SMMs, was realized by G.-M. Wang and co-workers who inserted 2,4,6-tri(4-pyridyl)-1,3,5-triazine (TPT) molecules into the crystal lattice formed by [Dy^III^_3_(H–HEDP)_3_(H_2_–HEDP)_3_] (HEDP = hydroxyethylidenediphosphonate) chains.^661^ For the pristine system before irradiation, no SMM behavior is reported. However, under UV light, first, the crystals change their color from colorless to blue which is represented by the modification of electronic absorption spectra, as well as the blue emission of TPT ligand weakens over the time of irradiation. Such behavior is attributed to the solid-state formation of TPT^•^ and O^•^ radicals within the PET mechanism from HEDP ligands. From the point of magnetic properties, at first, only a small contribution from ferromagnetic interactions between Dy(III) centers is observed below 5 K in the *dc* magnetic characteristics. Apart from the increase of the *χ*_M_*T* signal due to radical formation, more pronounced magnetic exchange is found after irradiation. Moreover, the presence of improved magnetic contact seems to quench the QTM effect, thus the photoirradiated phase reveals zero-*dc*-field slow relaxation of the magnetization effect with an effective energy barrier of Orbach relaxation of ca. 108 K, but without an appearance of the *M*(*H*) hysteresis loop.^661^

**Figure 74:**
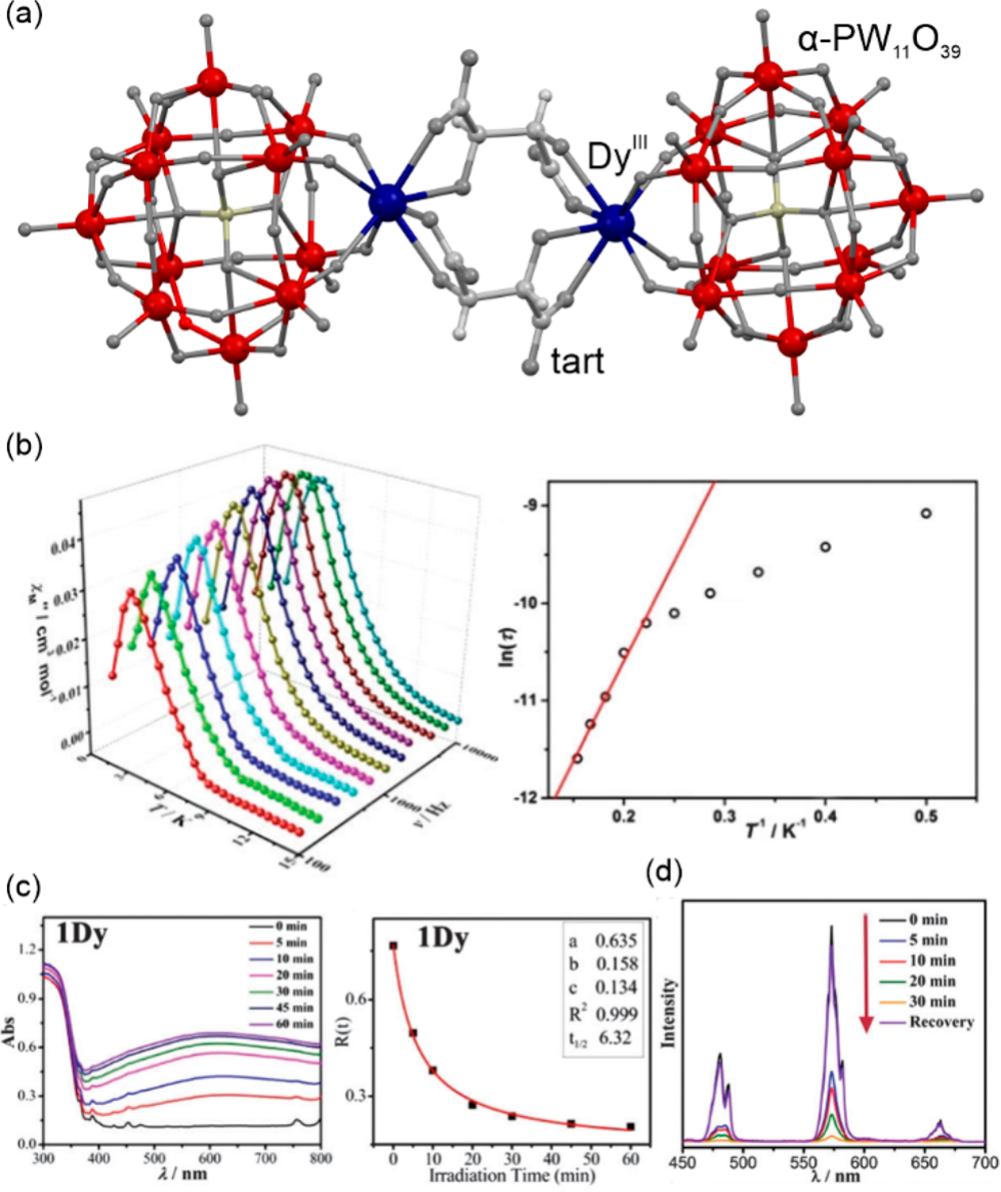
The structure of (N(CH_3_)_4_)_6_K_3_H_7_{[Dy^III^(tart)(α-PW_11_O_39_)]_2_}·27H_2_O (tart = tartrate) coordination system (a),^662^ the temperature dependences of the imaginary part of the *ac* magnetic susceptibility under the 4 kOe *dc* field (left part), shown together with the temperature dependence of the resulting relaxation time (right part) (b), time evolution of the solid-state diffuse reflectance absorption spectra under the Xe lamp irradiation, shown together with the related decay curve for the reflectivity at 605 nm (c), and time evolution of the emission spectra upon the 367 nm excitation, also under the Xe lamp irradiation, present with its recovery on exposure to air for 5 days (d). Parts (b), (c), and (d) were reproduced from ref (662) with permission from the Royal Society of Chemistry.

A completely different approach, that is utilizing the photoactivity of phosphotungstates was presented by J. Niu, J. Wang, and co-workers (Figure 74).^662^ Combining Dy^3+^ ions with tartaric acid and α-[PW_11_O_39_]^n–^ moieties enables the formation of {[Dy^III^(C_4_H_2_O_6_)(α-PW_11_O_39_)]_2_}^16–^ molecular anions accompanied by tetramethylammonium, potassium, and protonium cations, as well as the large number of water molecules of crystallization within the crystal lattice. The resulting system with negligible absorption in the visible range changes its color under the Xe lamp irradiation. The appearance of a broad band in the visible range was attributed to an intervalence transition between photogenerated W(V) centers and remaining W(VI) sites. Changes within the absorption spectrum disappear over time when the powder sample is kept in the dark under ambient conditions, suggesting reversible photochromism. Under 367 nm excitation, the as-synthetized system reveals Dy(III)-centered emission due to its f–f electronic transitions. Light exposure significantly decreases the overall room-temperature emission intensity but it recovers to the initial value after ca. 5 days in the dark. Alternate-current magnetic studies present distinct field-induced SMM behavior with a small energy barrier of 20 K for the initial system. However, at 2 K, the presence of a butterfly-shaped hysteresis loop was noted. Despite this, no magnetic data regarding the photogenerated metastable state were reported.^662^

**Figure 75:**
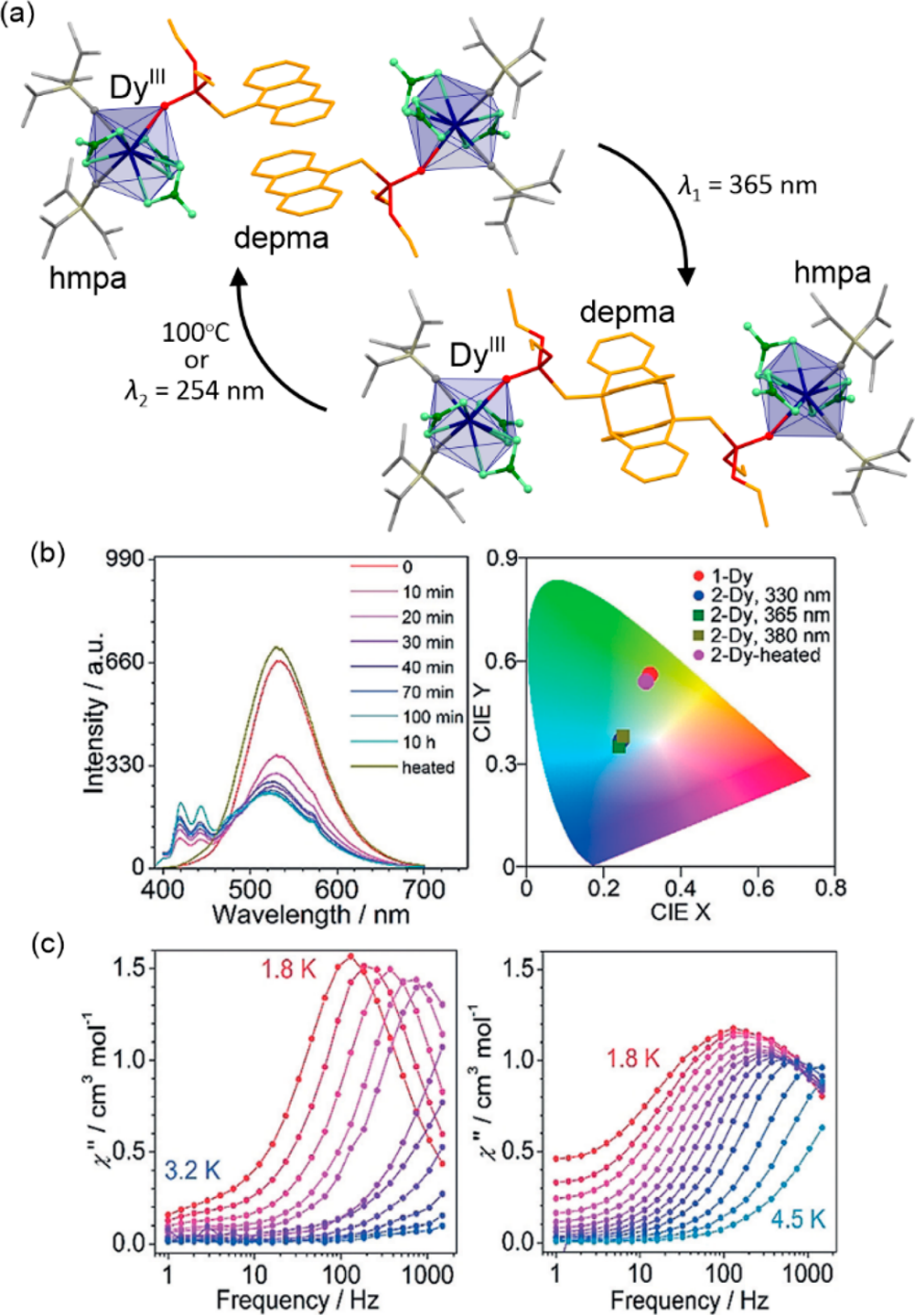
The structure of [Dy^III^(depma)(NO_3_)_3_(hmpa)_2_] (depma = 9-diethylphosphonomethylanthracene; hmpa = hexamethylphosphoramide) metal complex and the photogenerated dinuclear species from two such complexes (a),^1128^ time evolution of the emission spectra under the 365 nm light excitation and after thermal recovery at 100°C, shown with the CIE 1931 chromaticity diagram illustrating emission color change due to photodimerization (b), and the temperature-variable frequency dependencies of the *ac* magnetic susceptibility before (left side) and after irradiation (right side) under the 500 Oe *dc* field (c). Parts (b) and (c) were adapted with permission from ref (1128). Copyright 2018 John Wiley & Sons.

In recent years, to achieve efficient photoswitching of both magnetic and optical properties, a rich variety of anthracene-functionalized O-donor ligands was explored by L.-M. Zheng and coworkers. For instance, [Dy^III^(depma)(NO_3_)_3_(hmpa)_2_] mononuclear complex (hmpa stands for hexamethylphosphoramide and depma is 9-diethylphosphonomethylanthracene) combines both ligand-centered excimer-type yellow-green emission and field-induced slow magnetic relaxation (Figure 75).^1128^ Upon 365 nm light irradiation at room temperature, two neighboring molecules unveil [4+4]-photocycloaddition in the solid state in a single-crystal to single-crystal manner. Then the binuclear species show slightly modified SMM characteristics under the applied magnetic field with the energy barrier for the Orbach relaxation changing from 20.4 K to 43.2 K. On the other hand, the overall room-temperature emission upon 365 nm light irradiation of the pristine system abruptly decreases, and the dimerized product reveals a blue-white emission color due to the superposition of reduced ligand-centered luminescence and appearance of Dy(III) f–f electronic transitions. Owing to the light reversibility of the [4+4]-cycloaddition, the 254 nm irradiation recovers the initial state, but the same effect can be quickly obtained by heating the photoirradiated sample to ca. 100°C. The reverse transition appears both for single crystals, as well as within magnetic and photoluminescence studies. Similar systems comprising of Dy(III) nitrate, depma or dmpma (9-dimethylphosphonomethylanthracene) chromophores, and supporting diphosphonates also reveal field-induced SMM behavior and visible luminescence; however, the efficiency of light-induced switching was found to decrease for diphosphonate derivatives with three isopropyl groups, showing the impact of steric hindrance upon the kinetics of cycloaddition and its completeness both for single crystals and powder samples.^1129^ Combining Dy(III), chloride anions, and depma ligands results in two structural polymorphs (called α and β) with different energy barriers of the spin reversal under the applied field of 32.3 K and 66.6 K, respectively.^1130^ Both of them also reveal photoinduced cycloaddition and their emission properties originating from the excimer-type luminescence vary both before and after UV light irradiation.

Within the same family of compounds, the introduction of 4-OHpy ligands (4-OHpy = 4-hydroxypyridine) and SCN^–^ anions finally induces strong magnetic anisotropy of Dy(III) centers accompanied by the depma units, resulting in the appearance of zero*-dc*-field SMM behavior and a magnetic hysteresis loop of the molecular origin at cryogenic temperatures. The resulting [Dy^III^(SCN)_2_(NO_3_)(depma)_2_(4-OHpy)_2_] system with an emissive peak maximum centered around 540 nm, within minutes, undergoes cycloaddition under the 365 nm light irradiation.^658^ This effect reduces the signal of ligands’ emission while simultaneously the energy barrier of the Orbach relaxation decreases from 288 to 138 K. The latter is accompanied by a shrinkage of the *M*(*H*) butterfly-shaped hysteresis loop at 2 K while the estimated magnetic blocking temperature (*T*_B_) drops from 3.8 K to 2.6 K. Thermal treatment leads to recovery of the initial state with both optical and magnetic properties as of the pristine material. The related system built of Dy(III), 4-OHpy, depma, and SCN^–^ anions additionally undergoes an order-disorder phase transition related to the 4-OHpy *trans*-positioned ligands, thus an additional property of the temperature-variable dielectric constant transition appears.^657^ Therefore, this molecular system will be mentioned in section 7.4. related to achieving even more complex multifunctionality in molecule-based magnetic materials.

A family of different molecular systems built of binuclear Dy(III)-based complexes bridged by 2,6-dimethoxyphenol derivatives with dmpma or depma ligands was also presented.^659,660^ All of its members reveal photoswitchable luminescence which is accompanied by the increasing anisotropic energy barrier appearing after thermally reversible photoinduced transformation. Despite the presence of zero-*dc*-field SMM behavior, all these molecular systems lack the typical *M*(*H*) hysteresis loop of a molecular origin down to cryogenic temperatures, both before and after the photocycloaddition process.

### 7.2 Photoswitchable Chiral and Polar Molecule-Based Magnetic Materials

**Figure 76:**
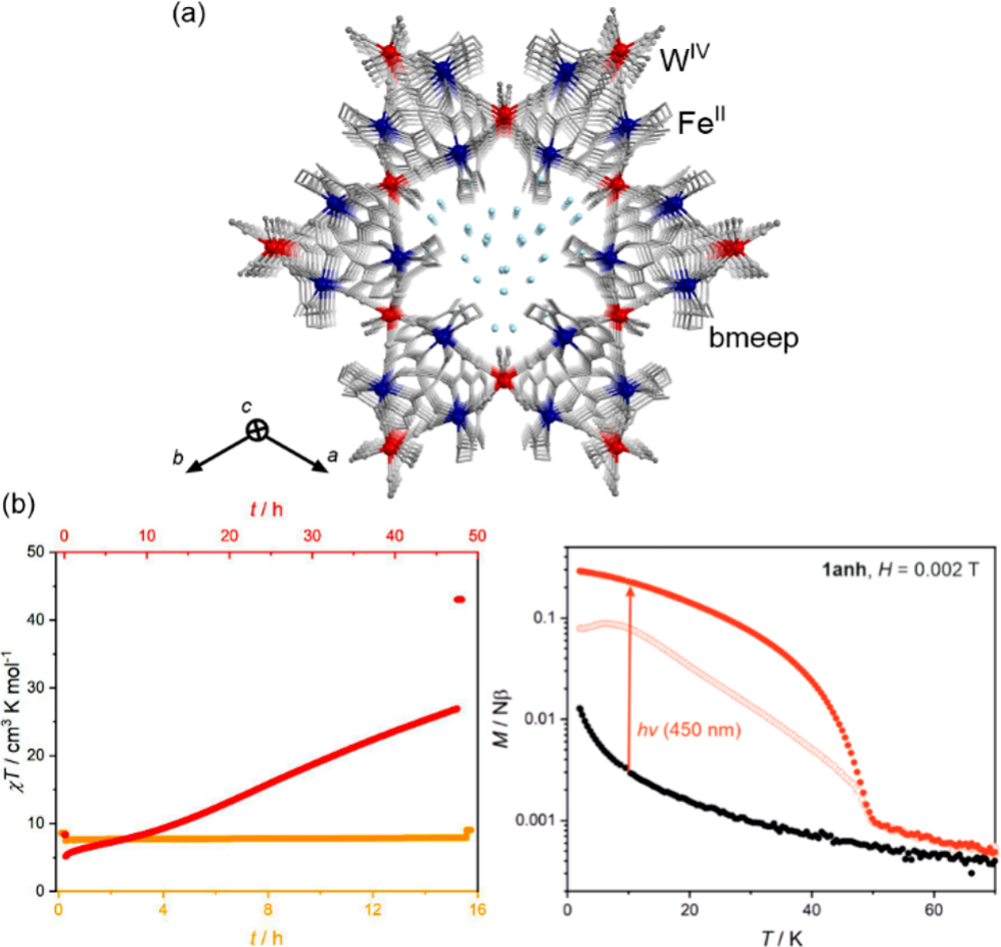
The representative structural view on chiral {[Mn^II^(bmeep)]_2_[W^IV^(CN)_8_]}·10H_2_O (bmeep = 2,6-bis[1-(2-(*N*-methylamino)ethylimino)ethyl]pyridine) coordination network (a),^948^ the time evolution curves of the *χ*_M_*T* product for the hydrated (orange) and fully dehydrated (red) phases under the 450 nm light irradiation at 10 K, shown alongside the field-cooled and zero-field-cooled magnetization curves for the anhydrous phase of this compound before and after irradiation with the 450 nm light (b). Part (b) was reproduced from ref (948) with permission from the Royal Society of Chemistry.

Similar to photoswitchable luminescent molecule-based magnetic materials (see above), the number of chiral or polar photoswitchable magnetic systems is very limited. Despite that chirality or polarity for molecular magnetic systems can be generated either by the spontaneous resolution using achiral precursors or rationally implemented chiral or polar molecular units, respectively, even the chiral magnetic molecular systems lacking photochromism in solid-state or solution form a relatively small class of materials as discussed in section 5. However, among various groups of photoswitchable molecule-based magnetic materials, some examples of the chiral and polar ones were reported. For instance, by utilizing [Mo^IV^(CN)_8_]^4–^ complexes, known for their intrinsic photomagnetism and potential electron transfer phenomenon with accompanying Cu(II) complexes in the solid state, {[Cu^II^((*R*,*R*)/(*S*,*S*)-chxn)_2_]_2_[Mo^IV^(CN)_8_]}·H_2_O (chxn = 1,2-diaminocyclohexane) chiral assemblies were obtained.^947^ Their enantiopurity was confirmed by circular dichroism (CD) spectra while the reversible photomagnetism was registered after their irradiation with blue light at 10 K. In the photoexcited state, antiferromagnetic exchange appears between Cu(II) centers and photogenerated *S* = 1 Mo(IV) complexes of substantial magnetic anisotropy. Interestingly, the use of ligand’s racemate as a precursor results in the coordination system of a layered topology, also showing photomagnetism with the modified coupling constants values. Nevertheless, the impact of light irradiation neither on the expected second-harmonic (SH) light generation nor on CD spectra was recorded for these chiral chain systems.^947^ The similar nature of the photoinduced response was presented for a porous {[Mn^II^(bmeep)]_2_[W^IV^(CN)_8_]}·10H_2_O (bmeep = 2,6-bis[1-(2-(*N*-methylamino)ethylimino)ethyl]pyridine) network which reveals chirality as the result of spontaneous resolution into a racemic mixture of enantiopure crystals belonging to the *P*3_1_21 and *P*3_2_21 space groups (Figure 76).^948^ Heating this system to ca. 60 °C removes the solvent of crystallization in a single-crystal-to-single-crystal manner and activates photomagnetic behavior at 10 K using a 450 nm light source. The latter leads to the appearance of long-range magnetic ordering with *T*_c_ of 48 K but, even after two hours annealing at 300 K, the pristine state cannot be fully recovered.^948^

Among cyanido-bridged coordination systems, an archetypical multifunctional Rb^I^{Mn^II/III^[Fe^II/III^(CN)_6_]} Prussian blue analog reveals physical effects related to its polar crystal structure.^209,369^ As described in the previous section (3.2.1), this PBA undergoes thermally activated and light-induced electron transfer transitions. Moreover, as shown by T. Nuida et al.,^209^ owing to the related thermal structural transition between the *F*43m and *I*4m2 space groups, SH response can be registered for both high-temperature and low-temperature phases, with its temperature dependence following the magnetic thermal hysteresis loop. Additionally, the low-temperature phase, with mixed valence Fe and Mn sites, reveals the conjunction of long-range magnetic ordering and ferroelectricity.^369^ However, no coupling between the photo-induced electron transfer and SHG or ferroelectricity was reported.

**Figure 77:**
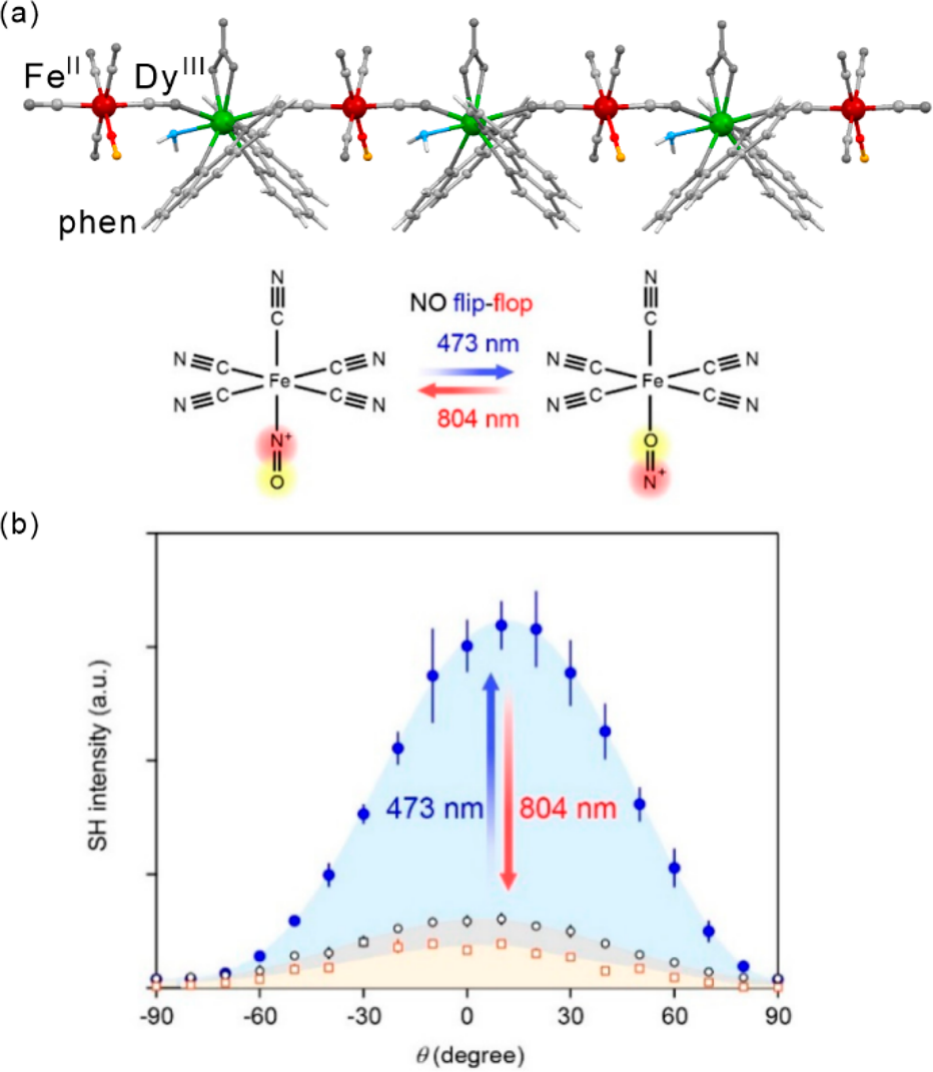
The structure of polar {[Dy^III^(phen)_2_(NO_3_)(H_2_O)][Fe^II^(CN)_5_(NO)]}·3H_2_O (phen = 1,10-phenanthroline) coordination chains, presented together with the scheme of the observed photoinduced flip-flop isomerization of the nitrosyl group of a nitroprusside fragment (a),^531^ the angular dependence of the SH intensity at *T* = 100 K, measured before photoirradiation (black circles), after the 473 nm irradiation (blue circles), and after the 804 nm irradiation (orange squares). Adapted with permission from ref (531). Copyright 2021 American Chemical Society.

Apart from the photoactivity of cyanido metal complexes resulting in the change of the spin state, different behavior can be observed for pentacyanidonitrosylferrate(II) complexes, which exhibit photoinduced flip-flop isomerization of the NO group upon visible light irradiation. Such a behavior was introduced to cyanido-bridged dysprosium(III)–nitroprusside coordination chains, {[Dy^III^(phen)_2_(NO_3_)(H_2_O)][Fe^II^(CN)_5_(NO)]}·3H_2_O (phen = 1,10-phenanthroline), which crystallize in a polar *Pna*2_1_ space group (Figure 77).^531^ The as-synthetized phase reveals a distinct SH signal upon NIR light irradiation, that further can be enhanced by introducing blue laser irradiation, activating the flip-flop transition of the nitrosyl group at 100 K. Then the reverse modification of the SH signal can be achieved by the red light. Such an effect was attributed to the pronounced variation of electric polarization of a crystal, further contributing to the change of SHG-related hyperpolarizability. Although this system additionally contains Dy^3+^ ions, neither luminescent properties nor the SMM behavior were reported, being the consequence of strongly absorbing in the visible region [Fe^II^(CN)_5_(NO)]^2–^ complexes and isotropic Dy(III) coordination environment. Moreover, the efficiency of the nitrosyl photoisomerization was estimated at the level of 5%, and despite it the SH signal increases upon blue laser irradiation ca. 5 times. Therefore, this way to switch the SHG seems as an attractive pathway for future studies.

**Figure 78:**
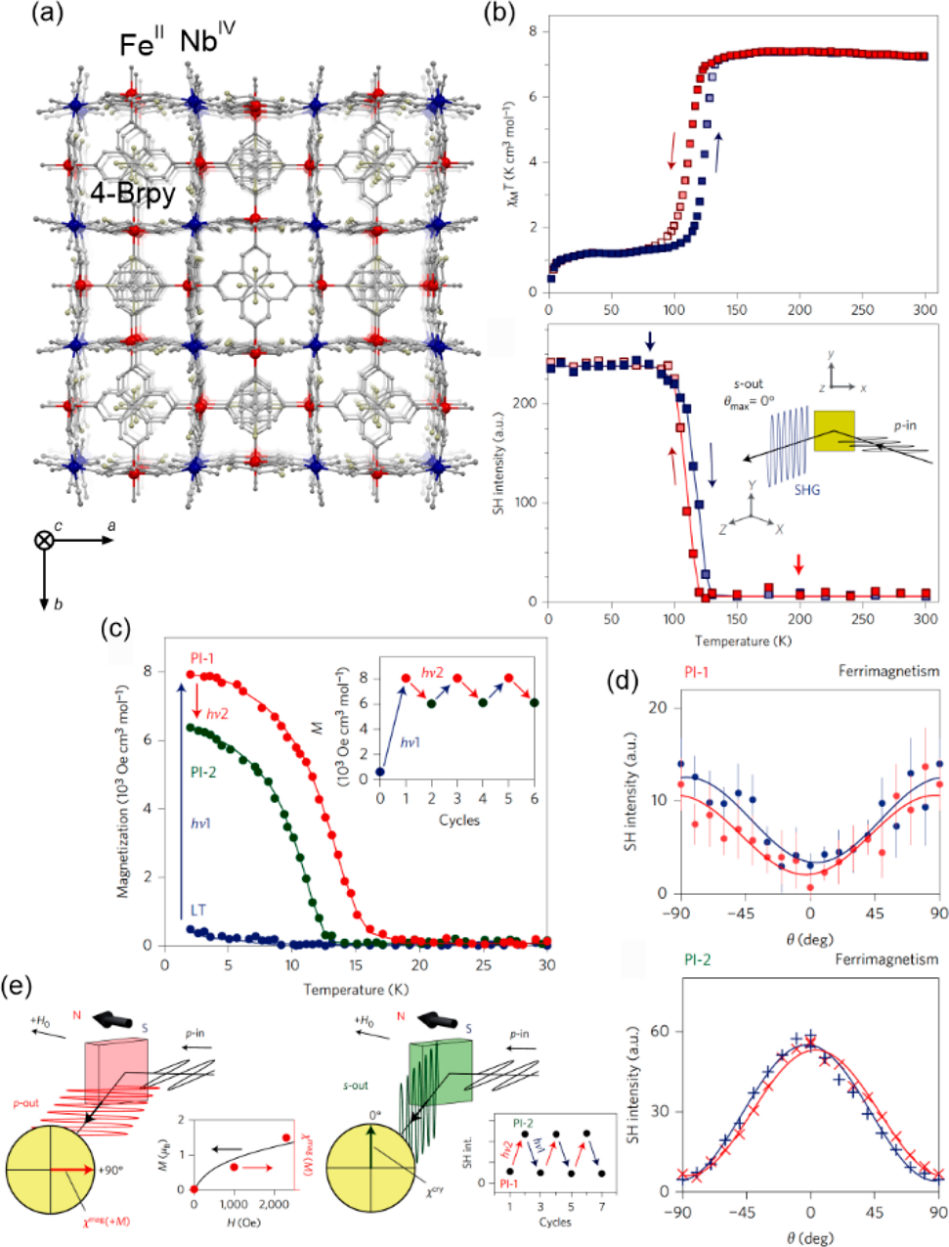
The structure of (+)-{[Fe^II^(4-Brpy)_4_]_2_[Nb^IV^(CN)_8_]}·2H_2_O (4-Brpy = 4-bromopyridine) coordination network (a),^143^ the temperature dependence of the *χ*_M_*T* product for the powder sample at 5 kOe, shown with the temperature dependence of the SH intensity for the single crystal (b), the magnetization versus temperature curves for in the dark, after irradiation by the 473 nm light, and further irradiation with the 785 nm light, together with several cycles of alternating irradiation at 5 K (c), the SH intensity versus *θ* (analyzer rotation angle) plots for the phases produced by the 473 nm (upper part) and 785 nm (lower part) light irradiation at 2 K (d), and the illustration of the SH polarization plane switching (e). In (e), the left small panel shows the magnetic field dependence of the SH intensity for the 473 nm light irradiated phase while the right small panel represents the SH intensity switching at *θ* = 0° by alternate irradiation with the 473 and 785 nm light sources. Parts (b), (c), (d), and (e) were reproduced with permission from ref (143) under terms of the CC-BY license. Copyright 2014 Springer Nature.

Another method of switching the magnetic state of cyanido-bridged frameworks (see section 3.) involved the use of the LIESST effect for the systems built with Fe(II) centers and octacyanidoniobate(IV) complexes. Therefore, assembling these molecular building blocks within a three-dimensional coordination framework together with supporting 4-bromopyridine ligands, leading to spontaneous resolution upon crystallization, resulted in the formation of SHG-active {[Fe^II^(4-Brpy)_4_]_2_[Nb^IV^(CN)_8_]}·2H_2_O (4-Brpy = 4-bromopyridine) photomagnetic system (Figure 78).^143^ Upon cooling, high-spin Fe(II) centers unveil a cooperative SCO phenomenon with a thermal hysteresis loop in the 110–130 K range, which is accompanied by the related modification of the SH light intensity owing to the transition between high-temperature *I*4_1_22 and low-temperature *F*222 space groups. Light irradiation at cryogenic temperatures using 473 nm wavelength induces the photogeneration of HS state at Fe(II) sites, which magnetically couple with *S* = 1/2 Nb^IV^ centers, leading to the long-range magnetic ordering. The photoinduced phase adopts the *I*4_1_22 space group as evidenced by the X-ray diffraction experiment. Therefore, such a structural change is sufficient to switch the maximum of the *θ* dependence of second harmonic light by ca. 90 degrees under the applied magnetic field, where *θ* stands for the analyzer rotation angle. Further irradiation with the 785 nm light activates the partial reverse-LIESST effect, lowering the magnetization and critical temperature of long-range magnetic ordering. Nevertheless, this is sufficient to recover the *F*222 space group symmetry, thus the maximal *θ* value returns to ca. 0 degrees. Both by following magnetization at 2 K, as well as the SH light intensity at 0° *θ* value, switching between two photogenerated phases is observed within several cycles of alternating irradiation. The observed spectacular effect of 90-degree switching of output light linear polarization is attributed to the alternating dominance of structural and magnetic contributions to the SH tensor.^143^

The presence of chiral metal centers can certainly affect the crystal packing, influencing simultaneously the cooperativity of a spin transition. As shown by T.-T. Ma et al., homochiral complexes of [Fe^II^(*RR*/*SS*-L_bpchda_)(NCSe)_2_] (L_bpchda_ = *N*^1^,*N*^2^-bis(pyridin-2-ylmethyl)cyclohexane-1,2-diamine) reveal the thermal SCO around ca. 150 K which is also accompanied by the LIESST effect at cryogenic temperatures appearing after 532 nm light irradiation.^927^ A reverse-LIESST effect operates under 880 nm light resulting in partial relaxation of the photoexcited state. Similar results are obtained for different polymorphs of these systems with only slightly lower cooperativity. Nevertheless, the impact of the LIESST effect on the SHG activity and the chiroptical effects was not reported. However, when employing the racemic mixture of *SS* and *RR* form of L_bpchda_, the resulting system remains at the high-spin state within the whole temperature range.

Some chiral Fe(II) complexes, which potentially can reveal photoswitching of magnetic and optical properties due to the spin transition effect, were also studied utilizing ultrafast pulse techniques.^946,1131,1132^ For instance, [Fe^II^(phen)_3_]^2+^ (phen = 1,10-phenanthroline) low-spin complexes reveal a short-lived high-spin state under optical pumping. This complex was combined with different chiral anions, such as [As^III^_2_(tartrate)_2_]^2–^, tris(catechol)phosphate(V), tris(catechol)arsenate(V), and 3,4,5,6-tetrachlorocatecholphosphate(V).^1131^ Then the chiral forms of [Fe^II^(phen)_3_]^2+^ complexes racemize upon dissolution; however, in the solid state enantiomorphic space groups can be induced. Therefore for some of the systems, strong SHG activity is recorded, and its intensity can be correlated with electronic absorption properties. The [Fe^II^(phen)_3_][*Δ*-As^III^_2_(tartrate)_2_] system under femtosecond laser source reveals ultrafast LS-to-HS switching which can be recorded using transient absorption spectra, as well as time-resolved SHG experiment.^1131^ A different system of [Fe^II^(4,4′-diMebpy)]^2+^ (4,4′-diMebpy = 4,4′-dimethyl-2,2′-bipyridine) was studied in solution in the presence of chiral tris(3,4,5,6-tetra-chlorobenzene-1,2-diolato-κ^2^O^1^,O^2^)phosphorus(V) anions.^949^ Using a combination of transient absorption measurements and ultrafast CD spectroscopy, the decay of the photoinduced HS state was found to be accompanied by ultrafast changes in the optical activity due to the coupling of excited state relaxation to a symmetry-breaking twisting mode of the complex.

### 7.3 Chiral and Polar Luminescent Molecule-Based Magnetic Materials

Among molecular magnetic materials aiming at the conjunction of both luminescence and chirality or polarity within the crystal structure, the most common synthetic pathway involves the use of visible or NIR-emissive lanthanide(3+) ions. Some of the related attention in recent years was devoted to heterometallic Ln^III^–[M^V^(CN)_8_]^3–^ coordination assemblies, where [M^V^(CN)_8_]^3–^ (M^V^ = Mo, W) ions of *S* = 1/2 ensure exchange interactions with f-block metal centers, at the same time enabling the observation of 4f-metal-centered luminescence due to optical transparency in the visible-to-NIR regions.^715^ Then the introduction of chiral organic ligands may, besides ensuring the chiral structure, additionally introduce sensitization of the emission, such as for {[Eu^III^(*RR*/*SS*-*i*Pr-pybox)(dmf)_4_]_3_[W^V^(CN)_8_]_3_}·dmf·8H_2_O (*i*Pr-pybox = 2,2′-(2,6-pyridinediyl)bis(4-isopropyl-2-oxazoline)) chains exhibiting natural optical activity (NOA) and thermally switchable emission.^1133^ Similar molecular systems, built with Gd^3+^ and Nd^3+^ ions, supported by *SS*/*RR*-*i*Pr-pybox or *SRSR*/*RSRS*-Ind-pybox ligands (Ind-pybox = 2,6-bis[8H-indeno[1,2-d]oxazolin-2-yl]pyridine) combine magnetic exchange interaction, NOA, and visible light ligand-centered or sensitized 4f-metal-centered NIR emission.^713^ While for these systems chirality is generated naturally by employing chiral ligands, for {[Ln^III^(dma)_5_][W^V^(CN)_8_]} (dma = *N*,*N*-dimethylacetamide) systems with various lanthanides(III) centers, spontaneous resolution results in a non-centrosymmetric *P*2_1_ space group.^1025^ As a consequence, depending on the f-block metal ion, tunable SHG activity was recorded and discussed using theoretical approaches.

Despite the presence of magnetic exchange interactions for the systems presented above, such d–f magnetic coupling gives only little functionality due to the lack of magnetic ordering. Therefore, a more promising pathway included the use of magnetically isolated Ln(III) complexes, which facilitates the observation of SMM behavior. As for now, several methods of breaking the structural symmetry in the related materials were presented, at the same time introducing improved emissive features and SMM behavior. The use of diamagnetic [M^IV^(CN)_8_]^4–^ (M = Mo, W) ions enabled the construction of {[Nd^III^_4_(H_2_O)_17_(pzdo)_5_][M^IV^(CN)_8_]_3_}·9H_2_O (pzdo = pyrazine-*N*,*N*′-dioxide) layered networks crystallizing in the non-centrosymmetric and polar *C*2 space group as the result of the spontaneous resolution process.^798^ Therefore, in addition to the Nd(III)-centered field-induced slow relaxation of magnetization and sensitized NIR emission, both materials reveal a noticeable, however, rather weak SH signal. A much better SHG response was presented for {[Yb^III^(2,2′-bdpo)_2_(H_2_O)][Cu^I^_2_(CN)_5_]}·5H_2_O (2,2′-bpdo = 2,2′-bipyridine *N*,*N*′-dioxide) framework and its Nd^III^-substituted analog, reaching up to ca. 138% of the KDP reference.^792,1134^ For the latter system, both slow relaxation of magnetization effect and sensitized NIR emission were previously presented, as well as zero-*dc*-field SMM behavior in the case of the Dy^III^-based analog.^792^ Another molecular nanomagnet built with Dy^3+^ ions and partially deprotonated *N*,*N*′,*N*′′,*N*′′′-tetra(3,5-dimethyl-2-hydroxybenzyl)-1,4,7,10-tetraazacyclododecane combines field-induced SMM behavior with sensitized visible light emission and SH light intensity reaching ca. 30% versus the KDP reference owing to the *P*ca2_1_ space group.^1018^ On the other hand, [Yb^III^(tppo)_3_(NCE)_3_] (E = S, Se; tppo = triphenylphosphine oxide) materials crystallizing in the *R*3 space group show the ca. 86% and 127% SH signals of KDP, respectively.^1135^ The latter materials were employed for the construction of luminescent thermometers based on the Yb(III) f–f emission and revealed a field-induced slow magnetic relaxation. Additionally, both systems show an ultralow-frequency Raman scattering (16 cm^–1^) due to collective vibrations of the tppo ligands and pseudohalides.

**Figure 79:**
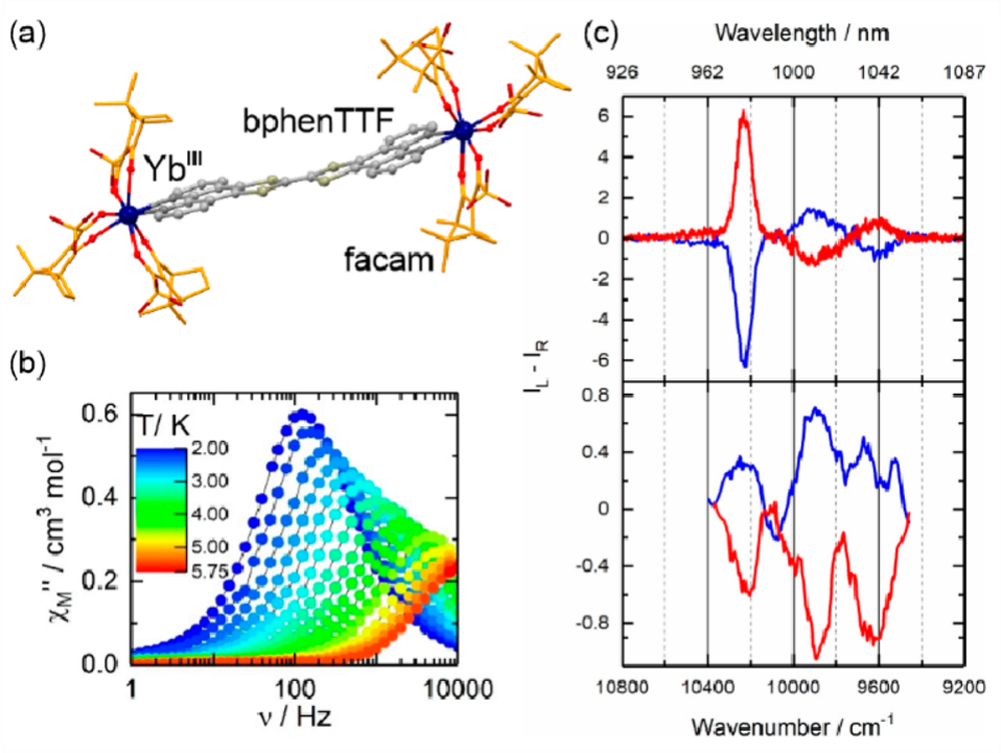
The structure of chiral {[Yb^III^(facam)_3_]_2_(bphenTTF)} (facam = 3-trifluoroacetyl-(+)-camphorate, bphenTTF = bis(1,10-phenantro[5,6b])tetrathiafulvalene) dinuclear molecule (a),^1010^ its temperature-variable frequency-dependencies of the imaginary part of the *ac* magnetic susceptibility under 1200 Oe (b), and room-temperature NIR CPL spectra for both enantiomers (related to the chirality of the facam ligand) in the dichloromethane solution upon the 300 nm excitation (upper part), shown together with the analogous spectra in solid state upon the 360 nm excitation (bottom part) (c). Parts (b) and (c) were adapted with permission from ref (1010). Copyright 2021 John Wiley & Sons.

Several attempts towards the generation of chirality aimed at achieving circularly polarized luminescence (CPL) for molecular nanomagnets were reported. For instance, the conjunction of Yb(III) centers with 3-trifluoro-acetyl-(+)-camphorato (facam) and bis(1,10-phenantro[5,6b])tetrathiafulvalene ligands resulted in binuclear lanthanide(III)-containing SMMs (Figure 79).^1010^ Both enantiomers crystallize in the chiral *C*2 space group, thus their NIR emission originating from emissive f–f electronic transition of Yb(III) centers was utilized to register NIR CPL spectra under UV light excitation. Although CPL spectra were registered both in solution and in the solid state, the former conditions give a much larger signal-to-noise ratio as higher |*g_lumc_*| values are observed. Upon transformation to the solid state, the maximal |*g_lum_*| value drops from 0.013 to 0.001 for the transition centered around 10235 cm^–1^. Both enantiomers are also redox-active due to the presence of tetrathiafulvalene triad as shown by cyclic voltammetry in dcm solution.^1010^

**Figure 80:**
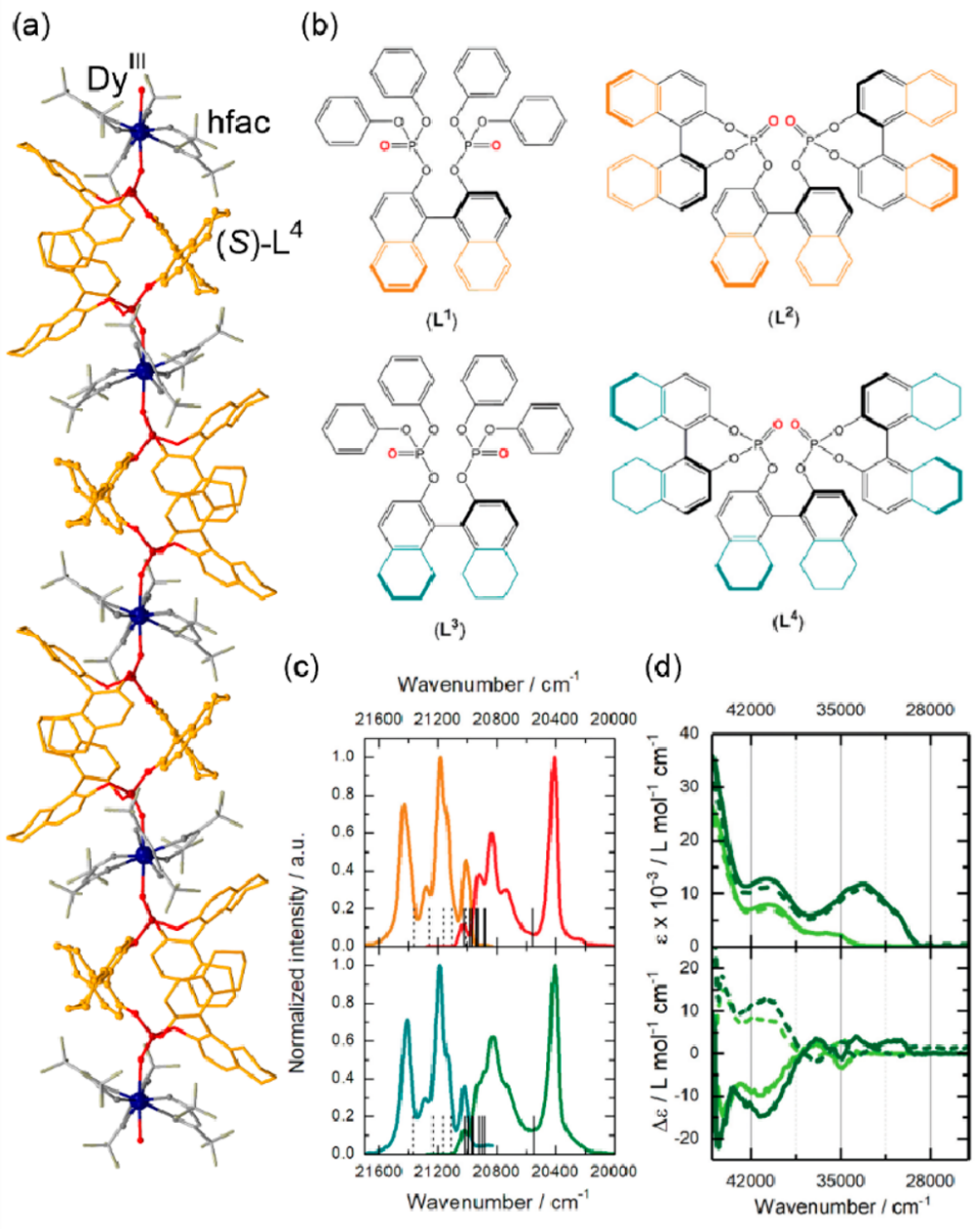
The structure of chiral [Dy^III^(hfac)_3_((*S*)-L^4^)] (hfac = 1,1,1,5,5,5-hexafluoroacetylacetonate; (*S*)-L^4^ = the (*S*) enantiomer of the binaphthyl-2,2′-diyl (BINOL) phosphate-type ligand, shown in the (b) part) coordination chain incorporating the Dy(III) complexes of the SMM character (a),^777^ the set of BINOL-derived ligands used for the construction of chiral luminescent Dy(III)-based chains (analogous to this presented in the (a) part) (b), representative excitation and emission spectra of two enantiomorphic Dy(III)-based chains incorporating (*S*)-L^3^ and (upper part) and (*S*)-L^4^ (bottom part) ligands (c), and the set of electronic absorption spectra (upper part) and electronic circular dichroism (ECD; bottom part) spectra for (*S*/*R*)-L^4^ (light green lines) and {[Dy^III^(hfac)_3_((*S*/*R*)-L^4^)] (dark green lines) (d). Parts (b), (c), and (d) were reproduced from ref (777) with permission from the Royal Society of Chemistry.

Another successful pathway toward chiral molecular nanomagnets involved the use of BINOL-functionalized bisphosphate ligands, which were combined with Dy(III) centers (Figure 80).^777^ Three different mono and tris(binaphthyl)-derived bisphosphate ligands were employed, which gave chain-like coordination polymers crystallizing in chiral space groups. All of them reveal SMM behavior, while its origin has to be attributed to the dominance of the Raman relaxation mechanism. ECD spectra proved that chirality originating from BINOL-functionalized ligands is maintained in dcm solutions of the respective complexes. Although two of the systems were shown to exhibit sensitized visible light emission at 10 K, which enabled magneto-optical correlations, the related CPL experiments were not performed.^777^

The introduction of chirality was also found possible for binuclear lanthanide(III) SMMs obtained with the support of (*S*/*R*)-(+)-2-(6-methoxy-2-naphthyl)propionic acid (*S*/*R*-HL_mnp_) and 1,10-phenanthroline (phen).^1136^ The [Ln^III^_2_(*S*/*R*-L_mnp_)_6_(phen)_2_]·3dmf·H_2_O analogs with Eu^III^, Gd^III^, Tb^III^, and Dy^III^ were obtained, but only for the Dy^III^ systems, slow relaxation of magnetization under applied magnetic field was reported. The Eu^III^ and Tb^III^-based materials reveal intense emission at room temperature in the solid state originating from the f–f electronic transitions, the Gd^III^ analog shows a ligand-centered emission, while in the case of Dy^III^, the energy transfer towards the f-block metal center is rather inefficient and the emission is weak. Even though for all the systems the solid-state ECD spectra confirm the chirality of the complexes, the CPL measurements were only reported for the Eu^III^-based analog, showing a small |*g_lum_*| value of ca. 1·10^–3^ for the ^5^D_0_→^7^F_2_ emissive transition in the solid state. For the measurements performed in dcm solution, it seems that such organic ligands tend to dissociate from the complex, as no emission signals were observed.^1136^ Better magnetic and optical characteristics were presented for another binuclear system but composed of Dy^III^ and Zn^II^ centers encapsulated by a Shiff base ligand (L^Me2^), while the chirality was introduced at the f-block metal center using enantiopure [3-(trifluoromethylhydroxymethylene)camphorate]^−^ (camph-L) anions.^135^ The resulting systems, [L^Me2^Zn^II^(Cl)Dy^III^((+/−)-camph-L)_2_(MeOH)] shows a field-induced SMM behavior with an energy barrier for the Orbach process of ca. 25 K. Two distinct emission bands in the visible region attributed to two f–f electronic transitions of Dy(III) were found. Angle-dependent single-crystal CPL experiments show a small variation in the distribution of the related dissymmetry factor, |*g_lum_*|, which reaches 0.04 and 0.18 at 5 K.

**Figure 81:**
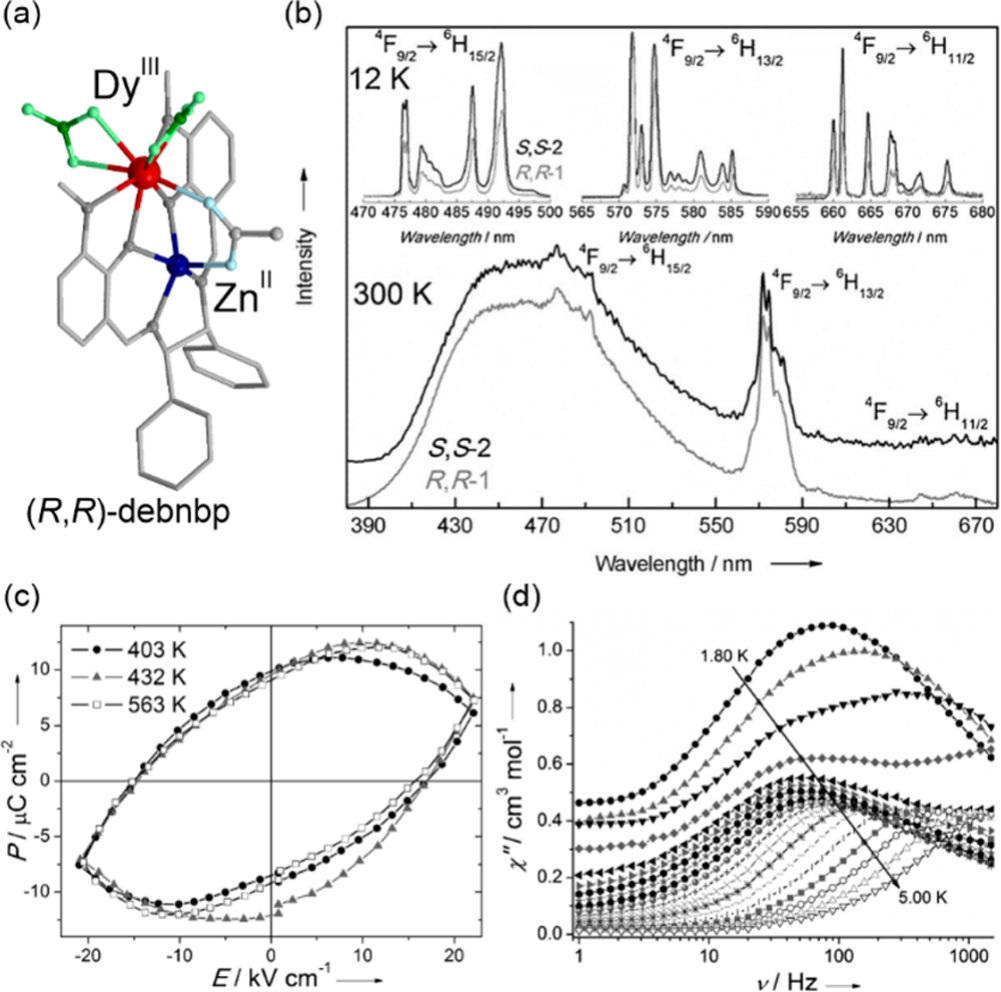
The structure of chiral polar [Dy^III^Zn^II^((*R*,*R*)-debnbp)(OAc)(NO_3_)_2_] ((*R*,*R*)-debnbp = the (*R*,*R*) enantiomer of the 2,2′[2,2-diphenyl-1,2-ethanediyl]bis[(*E*)-nitrilomethylidyne]bis(6-methoxy)phenol ligand; OAc = acetate) dinuclear molecule (a),^833^ emission spectra acquired at room temperature and at 12 K upon the 365 nm excitation for both enantiomers of the molecules (b), ferroelectric hysteresis loops measured at indicated temperatures on the selected single crystal of this compound (c), the temperature-variable frequency dependences of the imaginary part of the *ac* magnetic susceptibility at the 1500 Oe *dc* field (d). Parts (b), (c), and (d) were adapted with permission from ref (833). Copyright 2015 John Wiley & Sons.

A similar binuclear assembly built with Shiff base ligand, Zn^II^, and Dy^III^ centers was used for achieving the conjunction of SMM behavior, visible luminescence, and ferroelectricity.^833^ In this case, the enantiopure Shiff bases of *S*,*S*-H_2_debnpb or *R*,*R*-H_2_debnpb (2,2′[2,2-diphenyl-1,2-ethanediyl]bis[(*E*)-nitrilomethylidyne]bis(6-methoxy)phenol) were employed (Figure 81). The resulting chiral dinuclear Zn^II^–Dy^III^ complexes crystallize in the polar *P*2_1_ space group which is suitable to observe ferroelectricity. Thanks to the absence of solvent of crystallization, these materials unveil a ferroelectric hysteresis loop measured on a single crystal even at such temperature as 563 K. Moreover, the field-variable alternate-current magnetic studies indicate SMM behavior under 1.5 kOe external field with two energy barriers of ca. 20.5 and 51.7 K, which stays consistent with two symmetry-independent complexes within the crystal lattice. While at room temperature, the emission spectra show the presence of overlapped f–f electronic transitions of Dy^3+^ ions with the Shiff base ligand emission, at 12 K it was possible to collect high-resolution emission spectrum of the ^4^F_9/2_→^6^H_15/2_ origin and correlate its structure with the results of magnetic characterization.^833^

For a different assembly, where lanthanide(3+) ions were combined with a chiral (−)-4,5-pinenepyridyl-2-pyrazine ligand (pinpypz), 1-D [Ln^III^(hfac)_3_(pinpypz)]·H_2_O coordination systems were formed.^1137^ They crystallize in the *C*c space group and show room-temperature Ln(III)-centered emission for the cases of Sm^3+^, Eu^3+^, and Tb^3+^ ions. For the Dy^III^ analog, the presumable energy back-transfer process quenches its emission. Despite this, only for the latter compound, the field-induced SMM behavior is present under a 2 kOe external field with a small effective energy barrier of ca. 16 K. On the other hand, all reported systems show room-temperature ferroelectricity with the *P*(*E*) hysteresis loop collected on single crystals. Additionally, all these materials show the SHG effect with its efficiency being comparable with KDP and Ln(III)-dependent, reaching 120% of the KDP signal for the Sm^3+^-based analog.^1137^

**Figure 82:**
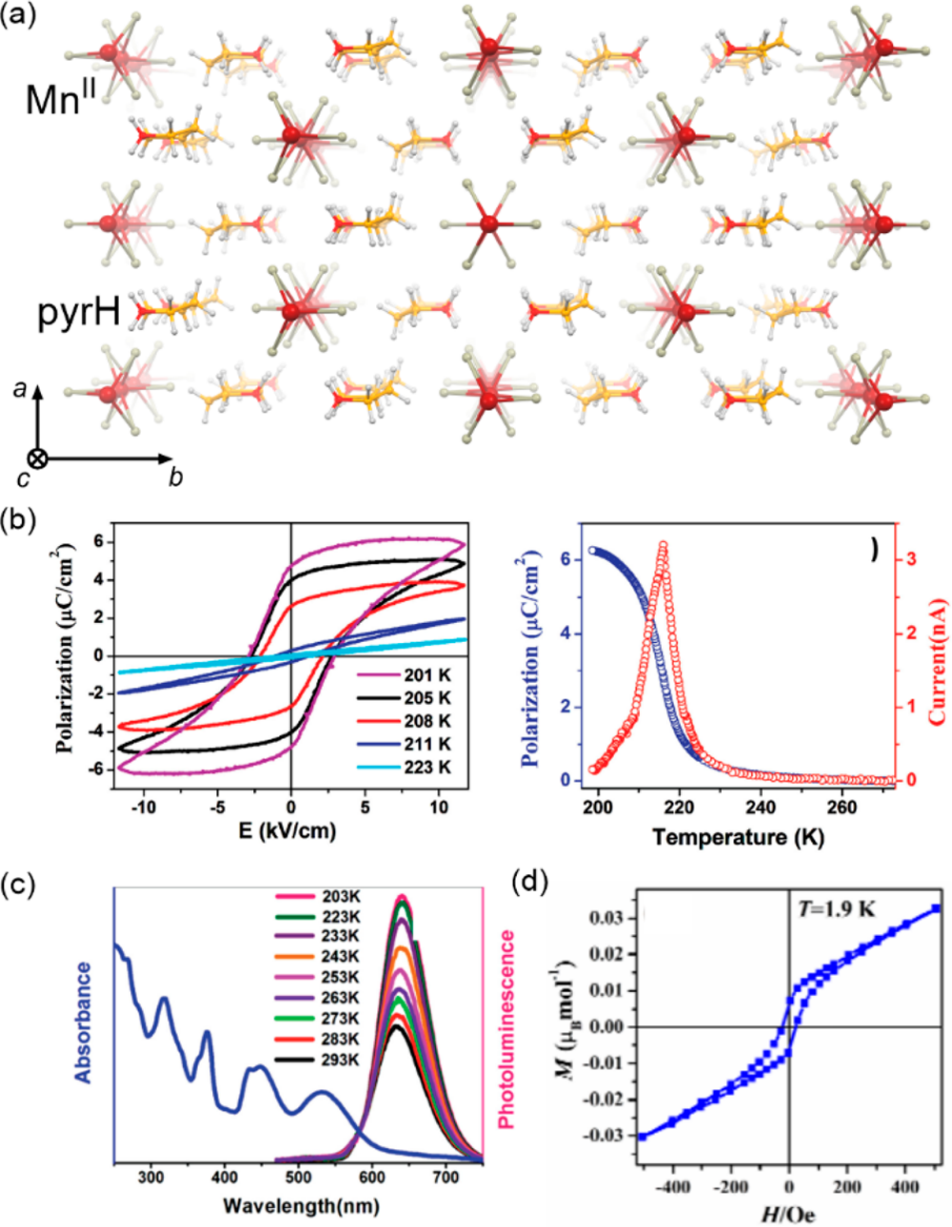
The structure of polar (pyrH)[Mn^II^Br_3_] (pyrH = pyrrolidinium) molecular hybrid below the critical temperature of ferroelectric ordering (a),^1027^ the ferroelectric hysteresis loops measured at various indicated temperatures along the *c* axis of the selected single crystal, shown together with the temperature dependence of spontaneous polarization and pyroelectric current measured also along the *c* axis (b), the UV–vis absorption spectrum and temperature-variable emission spectra (c), and the magnetization versus field hysteresis loop at 1.9 K (d). Parts (b), (c), and (d) were adapted with permission from ref (1027). Copyright 2015 John Wiley & Sons.

While, for polar or chiral lanthanide(III) complexes, luminescent characteristics were combined with the SMM behavior, a (pyrrolidinium)[Mn^II^Br_3_] molecular hybrid exhibits the conjunction of strong red luminescence with magnetic and ferroelectric ordering features (Figure 82).^1027^ Here, the emission itself originates from octahedral Mn(II) complexes and is only a little dependent on the temperature. Moreover, the presence of bromido-bridged chains results in long-range magnetic ordering below 2.4 K. While changing the temperature, the presence of the structural phase transition can be observed at ca. 219 K as depicted by calorimetric and dielectric measurements. The reason for the latter is an order-disorder phase transition for organic cations placed in the supramolecular lattice. The high-temperature phase adopts the orthorhombic *C*mcm space group, which is paraelectric, but below the critical temperature, the transition to the polar *C*mc2_1_ space group induces ferroelectricity. As a result, this material shows the *P*(*E*) hysteresis loops below 219 K measured along the polar *c* axis, thus serving as a unique luminescent multiferroic.^1027^

### 7.4 Extended Multifunctionality in Optical Molecule-Based Magnetic Materials

**Figure 83:**
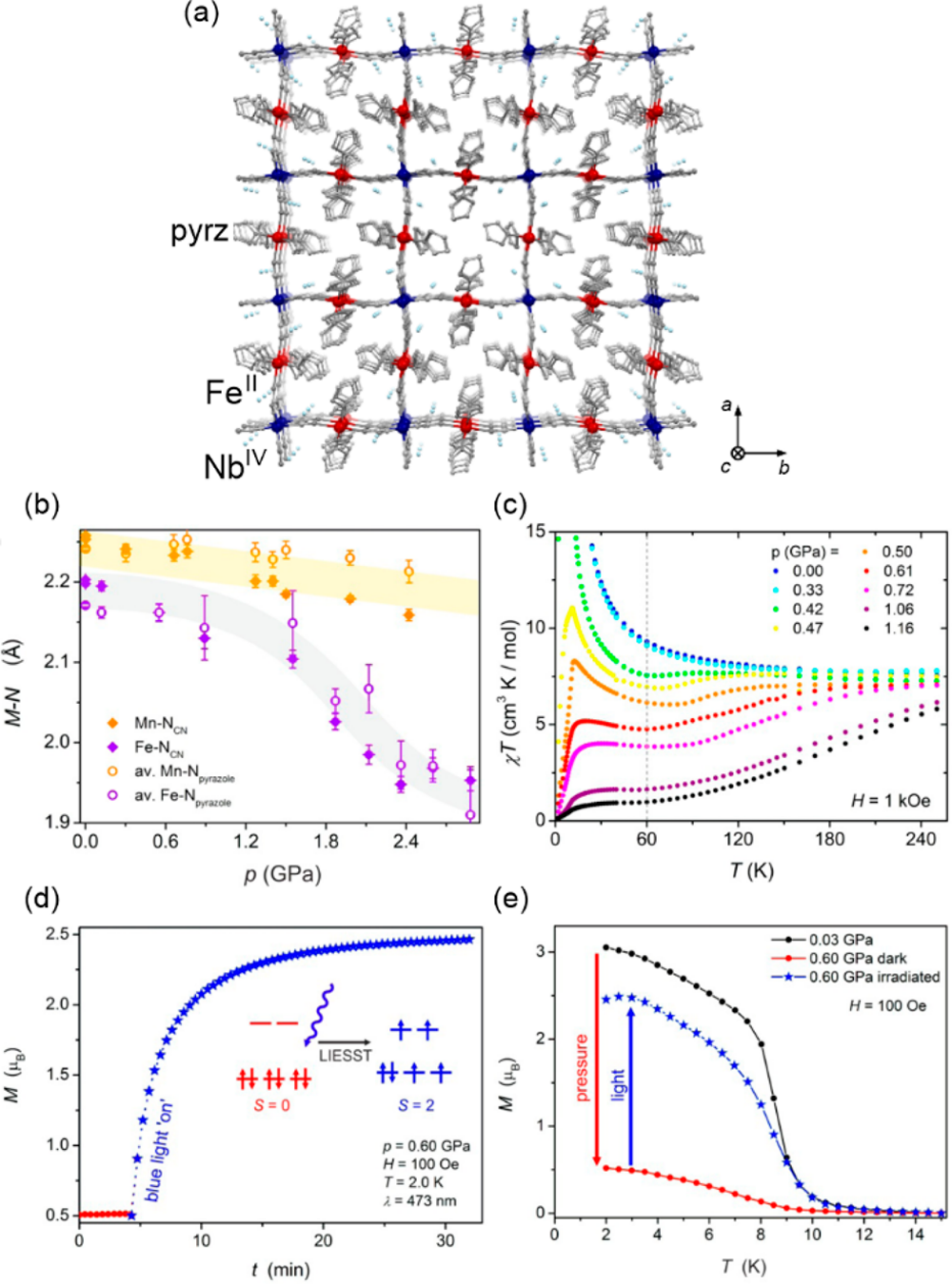
The structure of {[Fe^II^(pyrz)_4_]_2_[Nb^IV^(CN)_8_]}·4H_2_O (pyrz = pyrazole) coordination network (a),^144^ the pressure dependence of the averaged metal-nitrogen distances for this Fe(II)-based framework and its Mn(II) analog at room temperature (b), the pressure-variable temperature dependences of the *χ*_M_*T* product for the Fe(II)-based compound (c), its time evolution of magnetization upon the 473 nm irradiation at 2 K under 0.6 GPa (d), and the related field-cooled magnetization curves (e). Parts (b), (c), (d), and (e) were adapted with permission from ref (144). Copyright 2015 American Chemical Society.

In this last section, we will focus on systems that can be classified as molecule-based magnetic materials, and possess some intriguing optical properties but, moreover, reveal some physical features placing them among unique materials with super-extended multifunctionality. For instance, in 2015, D. Pinkowicz et al. reported a three-dimensional cyanido-bridged framework composed of paramagnetic high-spin Fe(II) and Nb(IV) complexes, {[Fe^II^(pyrazole)_4_]_2_[Nb^IV^(CN)_8_]}·4H_2_O, which, at low temperatures, behave as a ferrimagnet with *T*_c_ = 9.4 K (Figure 83).^144^ Initially, no thermal spin-crossover behavior was observed, however, this property appears upon applying the external pressure. At the pressure extending 1 GPa and while cooling below 80 K, a complete SCO transition on Fe(II) sites is achieved. Then using the conditions of 0.6 GPa external pressure, for which the ferromagnetic ordering is quenched, the light irradiation experiment was performed. It results in a strong photomagnetic response related to the Fe(II)-based LIESST effect. Then the HS centers, generated using a 473 nm laser source, magnetically couple with paramagnetic Nb(IV) complexes leading to a long-range magnetic ordering. While increasing the pressure above 1 GPa, the light response is also hampered, probably due to structural restraint of the Fe^II^ sites. However, under 0.6 GPa pressure, a large modulation of the *M*(*H*) hysteresis loop is observed. This behavior was compared with the properties of the Mn^II^-based analog which does not reveal pressure-induced spin-crossover behavior, as well as light-induced changes under applied external pressure. Therefore, the possibility of switching the magnetization by light was enabled by applying the additional external stimulus in the form of pressure, resulting in a multifunctional molecule-based magnetic material.^144^

**Figure 84:**
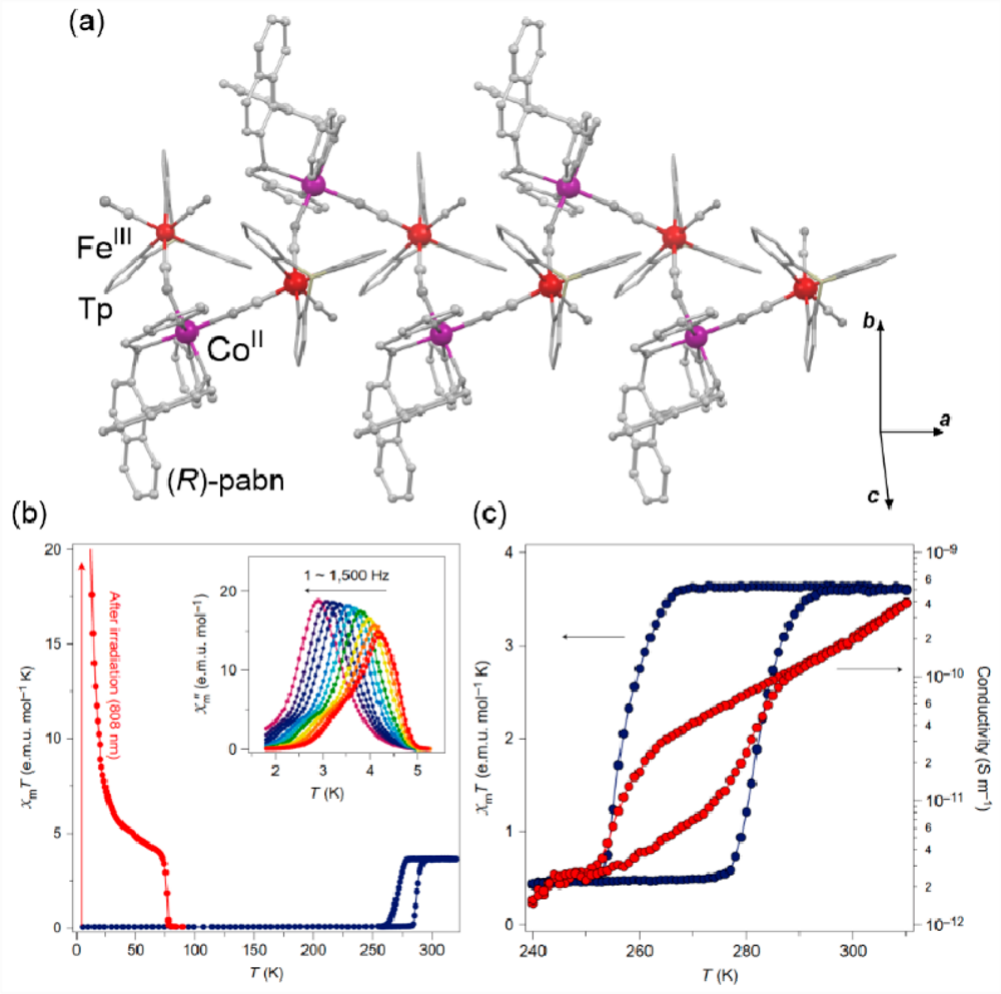
The structure of chiral {[Co^II^((*R*)-pabn)][Fe^III^(Tp)(CN)_3_]}(BF_4_)·MeOH·H_2_O ((*R*)-pabn = (*R*)-*N*(2),*N*(2′)-bis(pyridin-2-ylmethyl)-1,1′-binaphtyl-2,2′-diamine; Tp = hydrotris (pyrazol-1-yl)borate) coordination chain (a),^238^ the temperature dependence of the *χ*_M_*T* product for the hydrated phase of this compound before irradiation (blue points) and after the 808 nm irradiation (red points), shown together with the temperature dependences of the out-of-phase *ac* magnetic susceptibility for the indicated field frequencies (the inset) (b), and the temperature dependences of the *dc* electrical conductivity overlapped with the temperature dependence of the *χ*_M_*T* product showing the hysteretic ETCST effect (c). Parts (b) and (c) were reproduced with permission from ref (238) under terms of the CC-BY license. Copyright 2012 Springer Nature.

**Figure 85:**
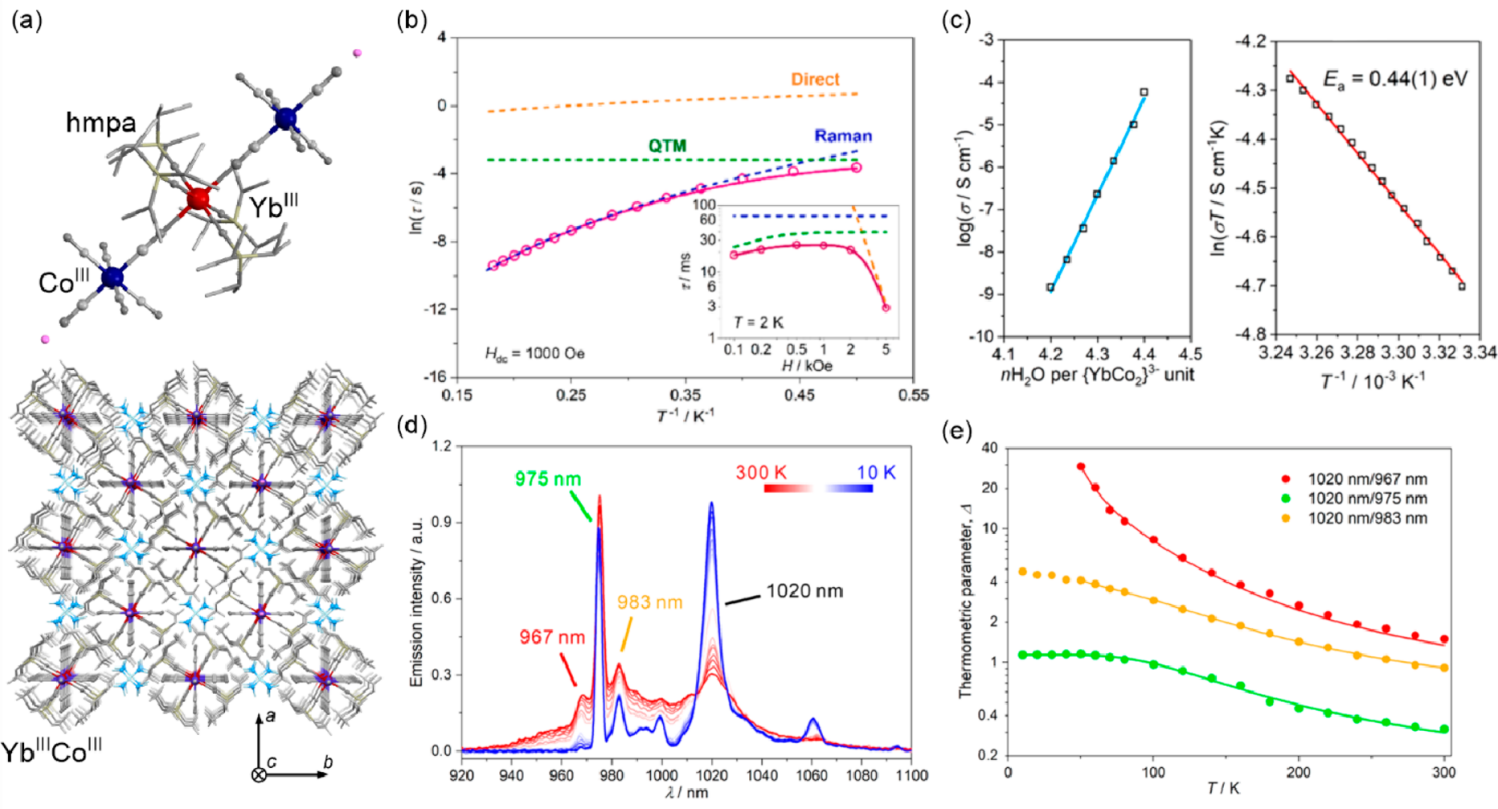
The representative structural views on (H_5_O_2_)_2_{(H)[Yb^III^(hmpa)_4_][Co^III^(CN)_6_]_2_}·0.2H_2_O (hmpa = hexamethylphosphoramide) molecular system (a),^239^ the temperature dependence of the magnetic relaxation time under the 1000 Oe *dc* field, shown together with the corresponding field dependence at 2 K (b), the correlation between observed proton conductivity and the overall number of water molecules per molecular unit (left) and the temperature dependence of proton conductivity at 90% of relative humidity (right) (c), temperature variable NIR emission spectra upon the 320 nm excitation, shown together with thermometric curves for three selected emission intensity ratios (d). Parts (b), (c), (d), and (e) were adapted with permission from ref (239). Copyright 2020 American Chemical Society.

In general, considering multifunctional magnetic materials, one should search for additional optical and electrical features combined. Among charge-transfer molecular systems, the introduction of photomagnetism and electron conductivity was achieved by H. Oshio and co-workers.^238^ The obtained {[Co^II^((*R*)-pabn)][Fe^III^(Tp)(CN)_3_]}(BF_4_)·MeOH·H_2_O ((*R*)-pabn = (*R*)-*N*(2),*N*(2′)-bis(pyridin-2-ylmethyl)-1,1′-binaphtyl-2,2′-diamine; Tp = hydrotris(pyrazol-1-yl)borate) system composed of zig-zag coordination chains not only reveals a chiral structure due to the presence of a supporting organic ligand but also undergo electron transfer-coupled spin transition (ETCST) with a thermal hysteresis loop between 290 and 320 K (Figure 84).^238^ Moreover, a partially desolvated sample containing only one water molecule of crystallization per formula unit shows decreased temperatures of ETCST, while for the as-synthesized system with two water molecules and one methanol molecule of crystallization, the thermal hysteresis loop appears between 278 and 296 K. At cryogenic temperatures, the partially desolvated system reveal light-induced magnetization after 808 nm irradiation, due to ETCST and ferromagnetic intrachain coupling between resulting paramagnetic centers. The photoinduced phase reveals partial SCM behavior and relaxes thermally at 78 K. The presence of electron transfer transition strongly affects electrical conductivity within the range of thermal hysteresis. The resulting hysteretic behavior for both *χ*_M_*T* and direct-current conductivity courses overlap, however, at increased frequencies, complex dielectric relaxation occurs.^238^ Lately, increased attention has been devoted to the introduction of proton conductivity for the systems built of luminescent lanthanide(III) molecular nanomagnets. Such multifunctionality was realized by linking Yb(III) centers with hmpa (hexamethylphosphoramide) ligands and hexacyanidocobaltate(III) anions into trinuclear molecular anions crystallizing with additional hydrogen and Zündel cations (Figure 85).^239^ The H^+^ cations stabilize this unusual type of ionic salt, being located between terminal cyanido ligands. On the other hand, structurally disordered Zündel cations serve as charge carriers for the observation of proton conductivity, which was found strongly humidity-dependent, reaching *σ* = 1.7·10^–4^ S·cm^–1^ at 97% relative humidity. Moreover, Yb(III) complexes within this (H_5_O_2_)_2_{(H)[Yb^III^(hmpa)_4_][Co^III^(CN)_6_]_2_}·0.2H_2_O system show field-induced SMM behavior, as well as NIR luminescence. Its emission features, that is the intensity ratios between the selected components of the NIR-emission band related to the Yb(III) ^2^F_5/2_ → ^2^F_7/2_ electronic transition, were utilized to construct a ratiometric optical thermometer. As a result, this material presents the conjunction of molecular nanomagnetism, optical thermometry, and humidity-variable proton conductivity, all achieved within a single-phase crystalline material.^239^

The work on this compound was followed by a considerable number of reports concerning the introduction of proton conductivity into molecule-based materials incorporating lanthanide(III) complexes.^1138−1141^ For instance, [Dy^III^_2_(phen)_4_(paa)_4_](ClO_4_)_2_ molecules with the 1,10-phenantroline (phen) ligands and 4-hydroxyphenylacetate anions (PAA) reveal the Dy(III)-centered emission in the visible range, as well as field-induced SMM behavior under *H*_dc_ = 2 kOe with the effective energy barrier of 86 K.^1138^ This system also shows proton conductivity reaching the *σ* value of 1.08·10^–5^ S cm^–1^ at 80°C and 100% RH. Next, some metal–organic frameworks composed of magnetically anisotropic Dy(III) centers were shown to reveal additional sensing features. The (Hdmbpy)[Dy^III^(H_2_dobdc)_2_(H_2_O)]·3H_2_O (H_4_dobdc = 2,5-dihydroxyterephthalic acid, dmbpy = 4,4′-dimethyl-2,2′-bipyridine) MOF reveals a high proton conduction of 1.2·10^–3^ S cm^–1^ at 100% RH and 343 K, accompanied by the ratiometric detection of pH within the broad luminescence of the H_2_dobdc^2–^ ligands.^1139^ Furthermore, the series of [Ln^III^_5_(L_3a4hb_)_6_(OH)_3_(dmf)_3_]·5H_2_O (H_2_L_3a4hb_ = 3-amino-4-hydroxybenzoic acid) frameworks shows various physical phenomena, such as field-induced SMM behavior for the Yb^3+^ analog or ratiometric luminescence thermometry for the mixed Gd^3+^–Eu^3+^–Tb^3+^ and Y^3+^–Eu^3+^–Tb^3+^ systems, which are accompanied by the CO_2_ adsorption properties.^1140^ Among MOF materials, [(CH_3_)_2_NH_2_][Dy^III^(bptc)]·2H_2_O (H_4_bptc = biphenyl-3,3′,5,5′-tetracarboxylic acid) framework reveals the combination of field-induced SMM behavior, visible light Dy^III^-based emission, and selective adsorption of Rhodamine B, while its Gd^III^-based analog exhibits the similar adsorption property but accompanied by the magnetocaloric effect and proton conduction.^1141^

Heterometallic Zn^II^–Ln^III^ complexes were also used for the construction of multifunctional molecule-based magnetic materials. One of them, based on [Zn^II^_2_Ln^III^(L_bmseae_)_2_(H_2_O)_2_](ClO_4_)_3_·3.5H_2_O (H_2_L_bmseae_ = 1,2-bis(3-methoxysalicylideneethylaminooxy)ethane) molecules, for the Dy^III^ case, exhibit field-induced slow magnetic relaxation and emission in the visible range, while for its Tb^III^-based analog, the conjunction of luminescence and proton conductivity was presented.^1142^ Exploting a similar approach, to combine SMM behavior, NIR emission, proton conductivity, and SHG activity, S.-J. Liu, C.-M. Liu, and co-workers obtained [Zn_2_Yb(*SS*/*RR*-L_chdabmbm_)_2_(H_2_O)_4_](ClO_4_)_3_·5H_2_O enantiomorphic systems (H_2_L_chdabmbm_ = cyclohexane-1,2-diylbis(azanediyl)-bis(methylene)-bis(2-methoxyphenol)).^1020^ At room temperature and 100% relative humidity, the *σ* value reaches 1.55·10^–4^ S cm^–1^ for this material. Moreover, these systems reveal chiroptical activity in the UV range as proven by CD spectra, the SHG effect with intensity reaching ca. 20% of the KDP reference, and a luminescent SMM character.

**Figure 86:**
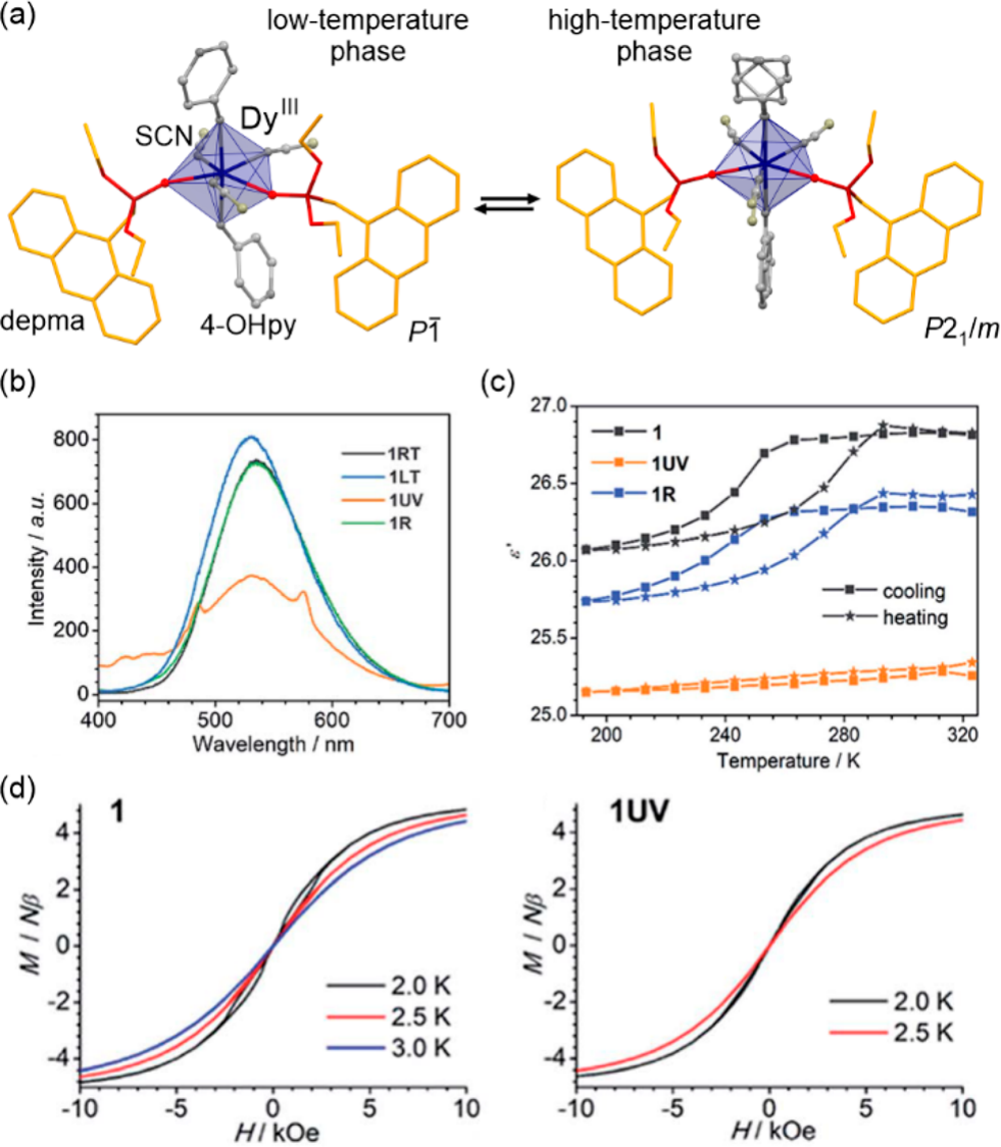
The structure of [Dy^III^(SCN)_3_(depma)_2_(4-OHpy)_2_] (depma = 9-diethylphosphonomethylanthracene; 4-OHpy = 4-hydroxypyridine) complex at 193 K (**LT**) and 300 K (**RT**) (a),^657^ emission spectra upon the 365 nm excitation of the pristine compound at 193 K (**1LT**), at 300 K before (**1RT**) and after the 365 nm light irradiation (**1UV**), and at 300 K after thermal relaxation at 393 K (**1R**) (b), the temperature dependences of dielectric constant for the pristine material (**1**), after its irradiation at 300 K with the 365 nm light (**1UV**), and after the thermal relaxation at 393 K (**1R**) (c), and magnetization versus field curves for **1** and **1UV** (d). Parts (b), (c), and (d) were reproduced from ref (657) with permission from the Royal Society of Chemistry.

Among photoswitchable single-molecule magnets with extended multifunctionality, the example reported by L.-M. Zheng and co-workers should be discussed.^657^ The [Dy^III^(SCN)_3_(depma)_2_(4-OHpy)_2_] (4-OHpy = 4-hydroxypyridine; depma = 9-diethylphosphonomethylanthracene) complex, which is built of highly magnetically anisotropic Dy(III) centers embedded in the geometry of a pentagonal bipyramid, undergo the photoinduced structural change leading to reversible modulation of SMM characteristics and the related *M*(*H*) hysteresis loop (Figure 86). Such behavior is accompanied by luminescent photochromism in the visible range as also presented for the other members of the family of lanthanide(III) complexes with depma ligands (see section 7.1. and Figure 75). However, the use of auxiliary 4-OHpy ligands introduces an additional feature into the final material which is the presence of an order-disorder phase transition appearing upon decreasing the temperature. The DSC curves show the transition to the ordered phase at ca. 240 K while the reverse transition upon heating appears at room temperature. This transition is accompanied by the change of the space group from the *P*2_1_/m at room temperature to the *P*1 one at 100 K. The related temperature-variable dielectric measurement reveals a hysteretic behavior of the dielectric constant for the non-irradiated phase. After UV light irradiation at room temperature, the dielectric characteristics of this system are modified to the point that the order-disorder transition disappears within the studied temperature range. Thermal annealing recovers the initial phase, thus the dielectric thermal hysteresis loop reappears. As a consequence, this unique system reveals the photoswitching ability not only of the magnetic and luminescent properties but also of the dielectric constant contributing to the related research on photoinduced dielectric constant observed, e.g., in hybrid perovskites.^657,1143^

**Figure 87:**
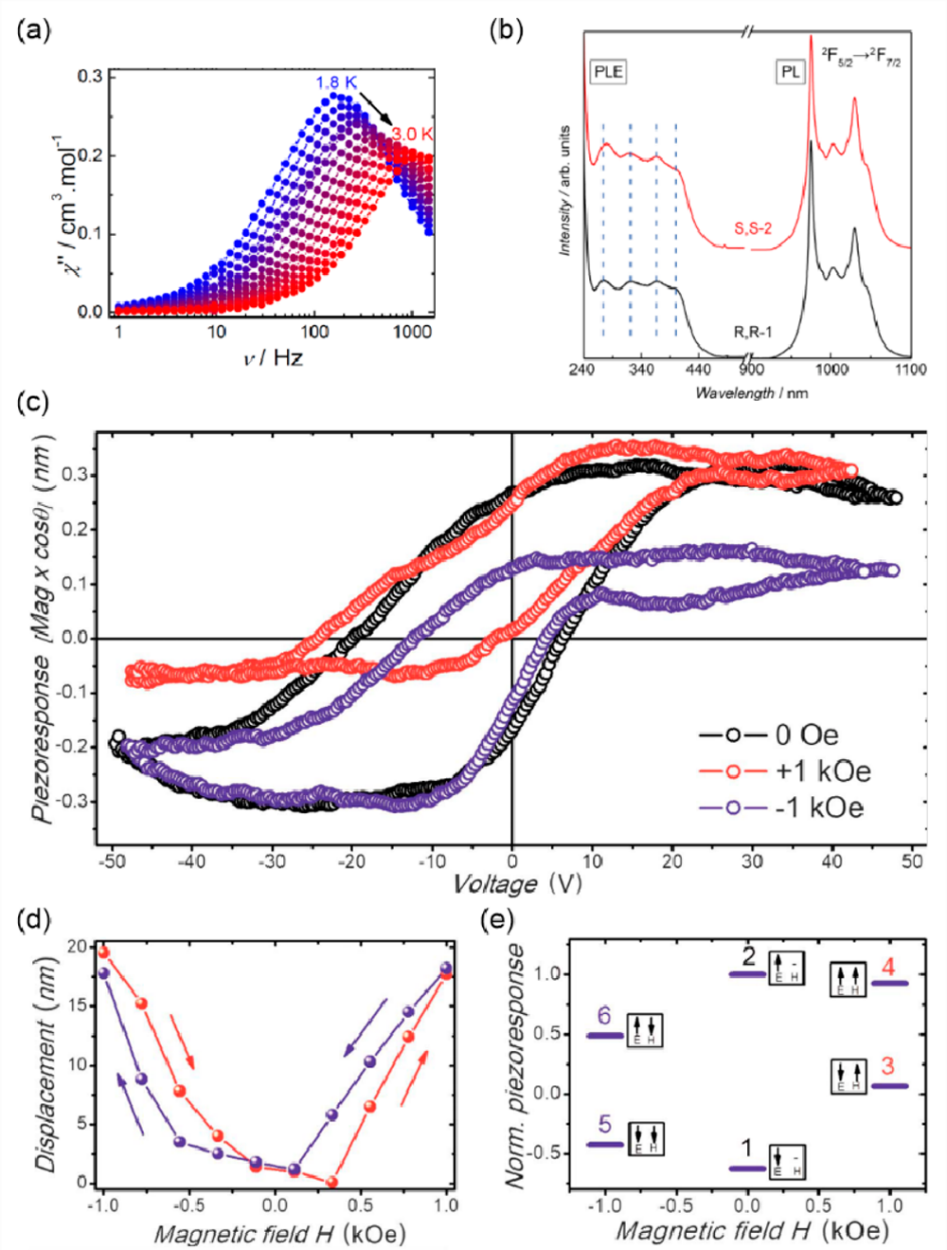
The optical, magnetic, electrical, and magneto-electric characteristics of chiral polar [Yb^III^Zn^II^((*R*,*R*)-debnbp)(OAc)(NO_3_)_2_] ((*R*,*R*)-debnbp = the (*R*,*R*) enantiomer of 2,2′[2,2-di-phenyl-1,2-ethanediyl]bis[(*E*)-nitrilomethylidyne]bis(6-methoxy)phenol ligand; OAc = acetate) molecule (the structure is analogous to shown in Figure 81 for the {Dy^III^Zn^II^} derivative), including temperature-variable frequency dependences of the *ac* magnetic susceptibility under the 600 Oe *dc* field (a), room-temperature excitation and emission spectra for both enantiomers (b), piezoresponse hysteresis loops obtained at zero and under applied magnetic fields of ±1 kOe for the single crystal (c), magnetic-field variable displacement representing the magnetostriction effect (d), and the magnetic-field dependence of normalized piezoresponse representing six remanent polarization states accessible by applying magnetic and electric fields (e). Reproduced with permission from ref (1028). Copyright 2020 American Association for the Advancement of Science.

In the field of multifunctional magnetic molecule-based systems, the ground-breaking results were recently presented by J. Long et al.^1028^ The enantiopure chiral Yb(III) analog of the system presented in section 7.3, that is [Yb^III^Zn^II^((*R*,*R*)-debnbp)(OAc)(NO_3_)_2_] ((*R*,*R*)-debnbp = the (*R*,*R*) enantiomer of 2,2′[2,2-diphenyl-1,2-ethanediyl]bis[(*E*)-nitrilomethylidyne]bis(6-methoxy)phenol ligand; OAc = acetate) dinuclear molecule, was found to exhibit the conjunction of NIR-centered luminescence, field-induced slow relaxation of magnetization, accompanied by room-temperature ferroelectricity as it was presented also for its Dy(III)-based derivative (Figure 87). Even up to 550 K, which is the temperature of the decomposition of the material, this system behaves as a molecular ferroelectric. The polar character of its single crystals was investigated by piezoresponse force microscopy (PFM), and the strongest responses for both in-phase and out-of-phase piezoelectric behavior were observed for the (011) plane. Upon applying the *dc* current bias voltage to different areas within the image, the uniform polarization states are visible for both responses. The inversion of bias voltage switches the phase by 180 degrees. Then the piezoresponse hysteresis loops could be collected using switching spectroscopy PFM, and the longitudinal piezoelectric coefficient of 73 pm·V^–1^ was determined. Moreover, the application of the external *dc* magnetic field of ca. 1 kOe along the (011) plane results in a redistribution of ferroelectric polarization as shown by PFM. The ferroelectric hysteresis loop measurements are also strongly affected by employing a magnetic field, especially in the context of height, asymmetry, and coercive field. As a result, a multilevel switchable system was obtained where each state is given by a proper combination of magnetic and electric fields. Strong room-temperature magneto-electric coupling was justified by magneto-elastic correlation related to paramagnetic Yb^III^ centers, as supported by X-ray diffraction measurements under an applied magnetic field. In this case, the emission property serves as an additional feature.^1028^

## 8 Summary, Conclusions, and Perspectives

The continuously developing family of molecule-based magnetic materials, which include magnetically ordered phases, spin transition materials, electron transfer systems, as well as molecular nanomagnets and spin qubit candidates, was found to be a great source of diverse optical phenomena. Some classes of optical properties, such as thermochromism occurring due to the spin crossover (SCO) effect, vis-to-NIR photoluminescence appearing often when lanthanide complexes are used for the construction of molecular magnetic systems, or natural optical activity observed when magnetic metal complexes are embedded in a chiral structure, are intrinsically related to the design of molecular magnets based on various metal complexes; thus, these optical effects were reported even at the earliest stages of the development of molecular magnetism field and their respective branches. However, in recent years, we witnessed a significantly increasing interest in further exploration of more advanced optical phenomena in molecule-based magnetic materials, partially due to the emerging research line of multifunctionality in materials science but also thanks to the promise of strong coupling between magnetism and optics in molecule-based materials. As a result, this review discussed a considerable amount of new reports, i.e., ca. 45% of 1153 references presented here were published within the last five years (2019–2023), underlying the broad scientific attention devoted very recently to the light-matter interactions in molecule-based magnetic materials.

In this regard, a few scientific directions were addressed the most. Among them, thermochromic effects were broadly recognized for SCO materials incorporating Fe(II) complexes. The gradual course of thermal spin transition was explored for sensing applications while the cooperative SCO giving the hysteretic behavior opened the pathway for the construction of memory devices. Of particular interest are the SCO compounds exhibiting close-to-room-temperature SCO which was achieved for a broad set of molecular systems, some of them were found to be processable into nanomaterials which is an important step toward applications. Diversity of accessible colors for thermochromism could be achieved by incorporating Fe(II) complexes into various types of coordination frameworks, ranging from porous Hofmann-type MOFs to nanosized polynuclear clusters. Relatively less attention was devoted to the exploration of other SCO-active metal centers; however, the pioneering works suggest that efficient thermochromism, e.g., between deep colors or connected with full discoloration at high temperatures, is accessible using Mn(III) or Cr(II) complexes, thus this direction is worth deeper investigation. It was presented that the optical absorption can be controlled through the SCO on single crystalline objects, and even the movement of the two-color interface can be controlled using temperature or light; however, the number of tested materials is small. An alternative mechanism of thermochromism is realized by electron transfer systems mainly based on heterometallic d–f cyanido-bridged assemblies, such as Co^II/III^–Fe^III/II^ and Co^II/III^–W^V/IV^ pairs. They offer a good transition cooperativity providing a large hysteresis loop even around room temperature. Therefore, they are promising candidates for optical memory effects while molecular materials revealing valence tautomerism present usually gradual electron transfer processes that might be exploited for optical sensing soon.

The usage of light for switching magnetic effects appears as one of the leading concepts in the last two decades of molecular magnetism. The dominant phenomenon is light-induced excited spin-state trapping (LIESST) which was broadly recognized for SCO-active Fe(II) complexes and explored in the contexts of memory devices and spintronics applications. The control of thermal relaxation of photoinduced state (generated usually at very low temperatures below 20 K) appears as the critical issue, and several studies were conducted to unveil the key structural factors such as supramolecular interactions or structural rearrangements occurring upon the related spin transition. The temperature of relaxation remained, however, rather low, usually not exceeding 100 K. The search for alternatives among other SCO-active metal ions resulted in the observation of the LIESST effect only for rare examples of Fe(III)-based molecular materials with moderate characteristics when compared with the best Fe(II) compounds. A new quality appeared when the photomagnetism of [M^IV^(CN)_8_]^4–^ (M = Mo, W) ions was discovered. First, it gives the pathway to relaxation of the photoinduced state at higher temperatures, much closer to the room temperature. Second, heterometallic systems using Fe(II) and [M^IV^(CN)_8_]^4–^ could be isolated opening the route for site-selective photoswitching that could be, however, also realized in the mixed-valence Fe(II)–Fe(III) molecules. The recent interest was devoted to the generation of a multi-step SCO effect toward the potential increase of information storage capacity; thus, the combination of such a course of spin transition with photoswitching ability was tested. This results in a few remarkable physical phenomena, such as reverse-LIESST, light-assisted spin-state annealing (LASSA), or temperature-assisted spin-state annealing (TASSA). Besides this, the LIESST can produce long-range magnetic ordering which was shown for Fe(II)–[Nb^IV^(CN)_8_]^4–^ and Mn(II)–[W^IV^(CN)_8_]^4–^ systems. These findings suggest that a room-temperature photomagnet could be achieved with this approach. The LIESST was also employed for photoswitching of molecular nanomagnets as demonstrated in pioneering works utilizing SCO-active Fe(II) complexes employed as a source of both the photoswitching effect and SMM/SCM behavior, or as a photoswitchable unit between SMMs based on other metal complexes; however, the challenge remains in employing high-performance lanthanide molecular nanomagnets in this regard. Recently, pioneering works on ultrafast photoswitching of metal complexes were reported which appears as an important step in the future employment of photoswitchable molecule-based magnetic materials in electronic devices.

The scope of photoswitchable phenomena in molecule-based magnetic materials was extended by playing with electron transfer processes. While the exploration of LMCT or linkage isomerism (e.g., of the nitroprusside ions) stays at a rather initial stage and should be investigated more, the photoswitching based on MMCT bands was fruitfully studied, mainly in heterometallic cyanido-bridged systems, e.g., Co^II/III^–Fe^III/II^, Co^II/III^–W^V/IV^, or Mn^II/III^–Fe^III/II^. In the latter case, ultrafast photoswitching was presented, providing the route toward the application in information processing. The pioneering works were performed on the extended coordination networks; however, recent years brought the miniaturization of the photomagnetic systems up to dinuclear molecules. The challenge remains in predicting the coordination environment to control an electron transfer and its photoswitching, especially for Co^II/III^–[W^V/IV^(CN)_8_]^4–/3–^ and other materials beyond Prussian blue analogs (PBAs). The photoinduced electron transfer could be employed for switching the molecular nanomagnetism; however, it is challenging as two metal centers should appear along with the source of SMM behavior, e.g., lanthanide ion. In this context, much better results in photoswitching of molecular nanomagnets were achieved using photochromic ligands. In general, a series of reports on using photochromic ligands to switch the spin state was presented. Among others, this employs various mechanisms, including light-induced coordination change, photoisomerization (difficult in the solid state), the mostly addressed photocyclization, or photocycloaddition. The latter was found realizable in the solid state when the proper arrangement of ligands is achieved which becomes a main issue in inducing and controlling the photoswitching effect using photochromic ligands. Nevertheless, remarkable examples of switchable lanthanide SMMs bearing photochromic units were presented. The alternative as encapsulation of Ln(III) molecular nanomagnets into photochromic MOFs was also provided. Thus, these two pathways are expected to be intensively explored.

In the context of studies of photoinduced changes in molecule-based magnetic systems, the risks concerning photoirradiation experiments are worth noting. As, usually, the molecule-based magnetic materials crystallize with additional solvent molecules and/or reveal sensitivity toward multiple external stimuli,^466,1144−1149^ the studies of photoinduced response should be performed with the appropriate awareness. For instance, volatile guest molecules, which are present in the crystal lattice, can be removed upon prolonged photo-irradiation, owing to the thermal treatment of the sample. Moreover, for a significant number of photomagnetic and photochromic materials, the reversibility of the photoinduced transformation is either limited or quenched completely, thus, this issue should be always precisely examined.^646,948,1150^ As the light-induced changes are often correlated with the reorganization of molecules within the crystal lattice, some attention should be given to determine if the impossibility of recovering the pristine state is not due to the partial removal of the non-coordinated solvent. An obvious solution to this problem requires performing studies on solvent-free systems,^485^ but often an additional effort necessary to characterize sensitive molecule-based materials in their solvated forms is sometimes worth a try. Although different methods of protecting the samples against solvent loss were reported both for photochromic and photomagnetic systems, it is always important to describe in detail how the reported compound was protected for the specific measurements.^466,1145^ Moreover, different methods of characterization for the light-induced phase can be employed, such as photocrystallography and X-ray absorption techniques.^464,1151^ Then the recovery of the pristine state should be proven by different experimental methods from the vast group of structural, spectroscopic, and magnetic studies, although even such simple basic characterization techniques as the elemental analysis or thermogravimetry, can serve as additional proof of the thermal or light-induced reversibility. Similar issues, as for photoswitchable materials, should be also addressed for luminescent and SHG-active molecule-based magnetic systems. In both cases, the intensity of the generated emission/SH-generation may vary due to the partial decomposition by either strong UV light or NIR laser irradiation, respectively. The basic physicochemical characterization before and after the related experiments should be performed to check the preservation of the integrity of the sample after the interaction with light. Moreover, for the case of luminescent molecule-based magnetic materials, additional information should be provided by carefully checking the time stability of the emission signal and the related characteristics, such as the emission lifetime or quantum yield, or even thermometric performance within several cycles of temperature change for optical thermometers.^795,1152,1153^

A broad range of optical phenomena are accessible when using luminescent metal complexes or organic molecules in the construction of molecule-based magnetic materials. However, it was found extremely difficult to build a luminescent molecule-based system showing long-range magnetic ordering with *T*_c_ above even 15 K as most transition metal ions, usually used for high-*T*_c_ molecular magnets, are not emissive. As a result, an expected correlation between spin ordering and luminescence was not well documented. On the contrary, luminescence was better incorporated into spin transition materials, and the coupling between a spin state and optical characteristics was presented. It was realized, first, for the composite materials incorporating separate SCO-active and emissive materials, but, more recently, a strictly molecular approach was also applied, and various luminescent SCO systems, from molecular to MOF systems, were reported. As the opto-magnetic correlation appears, the next step is to further explore the application of such materials for sensors and memory devices with optical output. The combination of luminescence with molecular nanomagnetism was the most widely studied especially as lanthanide complexes can efficiently combine strong photoluminescence with magnetic anisotropy resulting in the SMM behavior. This research line was exploited due to related magneto-optical correlations that were found useful as the optical insight into the mechanisms of the SMM effect. Moreover, the conjunction of luminescence and molecular nanomagnetism was proven to be an attractive route for multifunctionality realized both in homometallic complexes, mainly of lanthanide ions, as well as more advanced heterometallic systems. Among them, an emerging trend of linking luminescent thermometry with SMM property appeared as a route toward future SMM-based electromagnetic devices self-monitoring their temperature. The first attempts were also made to achieve the control of emission by magnetic field in molecule-based magnetic materials but the related effect was found rather weak for the reported cases. The solution could rely on exploring the interaction of magnetism with more subtle luminescent effects, such as up-conversion luminescence (UCL) and quantum cutting in the case of lanthanides, or charge transfer emission in the case of d-block metal complexes, that are expected to be more sensitive to external stimuli, thus, their correlation with a magnetic field can be stronger.

A different story is related to the usage of luminescence for addressing molecular qubits which appear now at the forefront of quantum information science. In this regard, transition metal complexes and their much rarely explored luminescence, related to d–d electronic transitions, were tested. As in the field of luminescence, the emission from transition metals becomes an emerging trend, it is expected that further exploration of this luminescence gives hope for new research lines in molecular magnetism. The efficient luminescence of transition metal ions together with their ability to form high-*T*_c_ molecule-based magnets may be a key for high-performance emissive magnets.

Incorporation of chirality and breaking the symmetry towards non-centrosymmetric space groups open another set of numerous optical phenomena, including circular dichroism (CD), circularly polarized luminescence (CPL), and nonlinear optical (NLO) effects, e.g., second harmonic generation (SHG), that can be coupled with the magnetism of molecule-based materials giving new physical cross-effects. Many strategies were employed to construct chiral and polar molecule-based magnetic materials. They usually rely on the usage of enantiopure organic ligands which were employed in the formation of unique families of chiral/polar magnetically ordered phases, spin transition materials, and molecular nanomagnets. Some synthetic challenges remain, e.g., the number of chiral high-performance lanthanide SMMs or electron transfer systems is very low as the ligand design is difficult, thus time-consuming organic synthesis is usually the only choice. It is, however, worth continuing this research quest as a series of impressive physical effects were presented for chiral and polar molecule-based magnetic materials. This is represented by magneto-chiral dichroism (MChD) appearing thanks to a combination of magnetic and natural optical activities which was recognized, e.g., as a possible tool for the optical read-out of the magnetic state for memory devices. The MChD was found to be strongly correlated with magnetism as shown in pioneering works on chiral spin-ordered magnets and SCMs. It was also presented for various SMMs and the currently discussed issue is the strategy of controlling the strength of MChD in such systems. The SHG property was found for a relatively large group of diverse molecule-based magnetic systems, and the strong coupling effect, named magnetization-induced SHG (MSHG), was detected but only at very low temperatures which is to be improved in the future. The other research lines, which were recently initialized, consist of the photoswitching of SHG and the correlation of SCO-related thermochromism with SHG for advanced optical sensing. The great challenge lies also in the generation of other NLO properties for molecule-based magnets and molecular nanomagnets.

Molecule-based magnetic materials are often responsive to various external stimuli, besides light and temperature, which was explored for switching their optical properties. In particular, chemical stimuli provide a great pathway for opto-magnetic switching in the contexts of both sensor applications as well as chemically-stimulated data storage and quantum computing. Chemical stimuli were used at room temperature to switch optical features, e.g., the sample’s color, together with a magnetic state, or as the post-synthetic treatment to achieve new properties as shown in desolvation-induced SMM behavior or even SMM-based optical thermometry. A smaller attention was devoted to the electric-field control over optical phenomena in molecule-based magnetic materials, only the single examples on high-*T*_c_ magnets and electron transfer systems were shown. This field is worth investigating more, especially for polar molecule-based magnets, which are expected to be more responsive to an electric field. The pressure was also broadly correlated with SCO properties in the related systems while little attention was paid to the pressure modulation of optical effects in other molecule-based magnetic materials. This challenge should be addressed in the future. The usage of mechanical force was demonstrated to be a non-trivial route of a post-synthetic modification of the structure and properties of molecular nanomagnets. An interesting future pathway can also rely on the usage of pressure to induce electron transfer processes in the uncommon metal pairs to unveil the unique opto-magnetic switching effects.

Last but not least, molecule-based magnetic materials were recognized as a great platform for multifunctionality including, e.g., a few different optical phenomena. The first group of such systems includes photoswitchable luminescent molecule-based magnetic materials which may offer an advanced emission-based optical output for the photomagnetic effect. This was proven by pioneering works on SCO-active Fe(II) complexes with photochromic luminescent ligands. Similar types of ligands could be also employed for the construction of lanthanide SMMs that additionally offer their characteristic emission. As a result, a photoswitchable emissive molecular nanomagnet was achieved and this pathway is expected to be explored much more soon thanks also to the great structural flexibility of lanthanide ions that enables the incorporation of expanded ligands adding the desired properties. The demonstration of further correlation of such photoswitching with the magnetic field effect remains a challenge. On the other hand, only very few reports discuss the synthesis and properties of photoswitchable chiral molecule-based magnets but the related unique systems reveal the great multifunctionality leading to, e.g., photoswitching of the SHG effect due to the photoinduced spin state change. More advanced effects, such as photoswitchable MChD, are expected for this class of materials but the limitations not only in the proper compounds’ candidates but also in the necessary instrumentation should be overcome. The conjunction of chirality and/or polarity with luminescence is an emerging research line in materials science due to the appearance of circularly polarized luminescence (CPL), having potential in multiple applications, e.g., CP-LEDs or advanced sensors, and the perspective of electric-field control over emission in molecular luminescent ferroelectrics. These concepts were recently found for molecule-based magnetic materials, especially lanthanide SMMs among which the CPL effect as well as the co-existence of ferroelectricity and luminescence were demonstrated. However, only very few examples without well-established design principles are known, and the exploration of coupling between the related optical and electro-optical effects with the magnetism of such systems is a future challenge that will demand improved instrumentation. Except for a single report on the Mn(II)-based molecular hybrid, the incorporation of chirality/polarity together with luminescence into magnetically ordered phases and spin transition systems is a future challenge.

Taking advantage of design principles towards diversely functionalized molecule-based magnetic materials, it was found achievable to generate even more extended multifunctionality as exemplified by pioneering works on pressure-induced photomagnet, simultaneous photoswitching of magnetism and electrical conductivity, the combination of SMM property with luminescent thermometry and super-ionic conductivity, as well as the set of photoswitchable emission, SMM, and dielectric constant characteristics, up to magneto-electric coupling found for a luminescent SMM. There are only a few very recent examples but much broader perspectives in (1) the photoswitching of multiple physical properties, (2) magnetic field control over various optical phenomena, (3) the magneto-electric coupling and its influence on optical properties, (4) the generation of more subtle optical phenomena, such as CPL, UCL, NLO, and the exploration of their interaction with magnetic field and other external stimuli in molecule-based magnetic materials, and (5) the investigation of optical phenomena exploiting the other (beyond UV–vis-NIR) parts of electromagnetic radiation, e.g., far-IR or THz range, which can be very sensitive to magnetic effects of molecular materials. These research lines are expected to be open in the near future.
